# On the Efficiency of the Density Functional Theory (DFT)-Based Computational Protocol for ^1^H and ^13^C Nuclear Magnetic Resonance (NMR) Chemical Shifts of Natural Products: Studying the Accuracy of the pecS-*n* (*n* = 1, 2) Basis Sets

**DOI:** 10.3390/ijms241914623

**Published:** 2023-09-27

**Authors:** Yuriy Yu. Rusakov, Valentin A. Semenov, Irina L. Rusakova

**Affiliations:** A. E. Favorsky Irkutsk Institute of Chemistry, Siberian Branch of the Russian Academy of Sciences, Favorsky St. 1, 664033 Irkutsk, Russia; rusakov82@mail.ru (Y.Y.R.); semenovval@inbox.ru (V.A.S.)

**Keywords:** pecS-1, pecS-2, ^1^H NMR, ^13^C NMR, ^15^N NMR, chemical shift, DFT, PEC, natural products

## Abstract

The basis set issue has always been one of the most important factors of accuracy in the quantum chemical calculations of NMR chemical shifts. In a previous paper, we developed new pecS-*n* (*n* = 1, 2) basis sets purposed for the calculations of the NMR chemical shifts of the nuclei of the most popular NMR-active isotopes of 1–2 row elements and successfully approbated these on the DFT calculations of chemical shifts in a limited series of small molecules. In this paper, we demonstrate the performance of the pecS-*n* (*n* = 1, 2) basis sets on the calculations of as much as 713 ^1^H and 767 ^13^C chemical shifts of 23 biologically active natural products with complicated stereochemical structures, carried out using the GIAO-DFT(PBE0) approach. We also proposed new alternative contraction schemes for our basis sets characterized by less contraction depth of the *p*-shell. New contraction coefficients have been optimized with the property-energy consistent (PEC) method. The accuracies of the pecS-*n* (*n* = 1, 2) basis sets of both the original and newly contracted forms were assessed on massive benchmark calculations of proton and carbon chemical shifts of a vast variety of natural products. It was found that less contracted pecS-*n* (*n* = 1, 2) basis sets provide no noticeable improvement in accuracy. These calculations represent the most austere test of our basis sets as applied to routine calculations of the NMR chemical shifts of real-life compounds.

## 1. Introduction

The ^1^H and ^13^C nuclear magnetic resonance (NMR) spectroscopy has become one of the most important means of studying the structure and dynamics of large naturally occurring compounds. The effectiveness and accuracy of the NMR analysis are enhanced via high-quality quantum chemical calculations, appending a great deal of validity to the assignments of the resonance signals of the NMR spectra. The accuracy of the quantum chemical calculations of NMR chemical shifts is determined using many factors, among which the most important ones are the method of calculation and the basis set used. 

Evidently, in modern quantum chemical NMR calculations of large natural molecules, the electron density functional theory (DFT) has become extremely popular [1,2]. Indeed, the DFT method takes into account the electron correlation effects via the exchange–correlation (XC) potential and has moderate computational requirements comparable to that of the Hartree–Fock method [3]. In this sense, one can hardly expect to find a more balanced approach than the DFT method. Today, a lot of work has been conducted to establish the most suitable XC functionals for the calculation of NMR chemical shifts [4,5,6,7,8,9,10]. However, regardless of what XC functional is employed, using unsuitable basis sets of little flexibility in the DFT calculations of NMR chemical shifts may give the results of poor quality or even erroneous results. In this respect, one should pay no less attention to the basis set issue than the choice of the XC functional used.

It is of prime importance to use the basis sets that give results, which are sufficiently close to the complete basis set (CBS) limit reached in a particular computational method. Early on, the NMR calculations of any compounds via the DFT approach were carried out using nonspecialized energy-optimized basis sets. Thus, the suitability of different families of energy-optimized basis sets to NMR chemical shift calculations was widely investigated [11], and it was found that in order to reach an acceptable convergence towards the CBS limit, one should resort to large quadruple-*ζ* quality basis sets. For example, Dunning’s cc-pVQZ [12] and Kutzelnigg’s IGLO-IV basis sets [13,14] of quadruple-*ζ* quality, consisting of, accordingly, 55 and 51 functions for the second-row atoms give the results close to the CBS limit [11]. However, this number of functions on each atom of the carbon skeleton presents an unsurmountable problem for large organic compounds. Such compounds may contain hundreds of carbon atoms, therefore, the calculations of their NMR chemical shifts using the DFT method with an otherwise favorable scaling factor of *N*^3^-*N*^4^ (with *N* being the size of basis set space) were practically unfeasible till the first decade of the 21st century.

The alleviation of the problem came with the appearance of the first specifically optimized basis sets, called the chemical shift-oriented (or, briefly, *δ*-oriented) basis sets. Frank Jensen was the first to make a suggestion that, in order to reduce the sizes of the basis sets used in the NMR shielding calculations, one should take usual energy-optimized moderate-sized basis sets, expand them with the least needed number of functions in the important exponential regions and optimize the added exponents with regard to the NMR shielding constants under interest. This allows dealing with the featured *δ*-oriented basis sets of moderate sizes in the NMR calculations, which, in turn, opens new avenues for accurate and fast predictions of the NMR chemical shifts of large natural products using the DFT method. 

Specifically, the famous Jensen’s (aug)-pcS-*n* (*n* = 0–4) basis sets [15] for the atoms of 1–3 periods (H-Ar) were created by adding an optimized tight *p*-function to the corresponding energy-optimized (aug)-pc-*n* (*n* = 0–4) basis sets [16,17,18,19]. Later on, more efficient segmented contracted basis sets of Jensen’s series, namely, the (aug)-pcSseg-*n* (*n* = 0–4) [20], were developed for the atoms of 1–4 periods by applying the P-orthogonalization procedure [21] to the generally contracted property-tuned basis sets. Remarkably, in our personal experience, the pcS-2 basis set of triple-*ζ* quality consisting of only 33 functions for carbon atom provides the accuracy comparable to that of a large nonspecialized cc-pVQZ basis set of quadruple-*ζ* quality consisting of as much as 55 functions. 

The development of the *δ*-oriented basis sets continues today. We also have introduced recently new effective *δ*-oriented basis sets, *viz.*, the pecS-*n* (*n* = 1, 2), purposed for the calculations of ^1^H, ^13^C, ^15^N, ^17^O, and ^31^P NMR chemical shifts [22,23], which were optimized using the property-energy consistent (PEC) method [24]. The pecS-1/pecS-2 basis sets consisting of only 5/14 and 18/34 functions for hydrogen and carbon atoms, respectively, demonstrated a very good accuracy against the experiment. Moreover, we also watched the pecS-*n* basis sets manifesting a remarkable robustness of the results in relation to the choice of the XC functional used. In all respects, our NMR-oriented basis sets [22,23,24,25,26] showed a fine balance between size and accuracy, which makes them a promising tool in demanding DFT calculations of the NMR parameters of large natural products. 

The first tests of the designed pecS-*n* (*n* = 1, 2) basis sets for the 1–2 row elements were performed on the example of 35 small and rigid molecules consisting of up to 15 atoms [22]. The performance of the pecS-*n* basis sets has never been studied on large real-life compounds. That is the gap that requires completion.

A matter of this present study is to demonstrate the performance of our pecS-*n* (*n* = 1, 2) basis sets on practice on the example of routine DFT calculations of 713 ^1^H and 767 ^13^C NMR chemical shifts of 23 large biologically active natural products of different classes. For this purpose, we have naturally chosen the PBE0 functional [27,28,29] because the pecS-*n* basis sets logically perform the best in combination with the PBE0 due to the fact that the latter was used for optimizing the original pecS-*n* basis sets. Thus, the pecS-*n* logically manifests the best performance only in combination with the PBE0 functional, and there is no point in discussing other functionals when calculating with the pecS-*n* basis sets. Thus, what is tested in this work is the performance of the developed basis sets only in combination with the PBE0 functional and no others.

Another important goal of this work is to study the changes in accuracy when applying our basis sets with less contracted *p*-shell. Indeed, we are witnessing rapidly developing computer technologies nowadays, which opens new perspectives for more computer-resource-demanding quantum chemical calculations. This allows one to slacken the pursuit of very small basis sets used in the NMR calculations of large molecules to some extent. In this respect, our pecS-1 and pecS-2 basis sets are indeed very compact due to a large contraction ratio, being of about 50 and 30% for the hydrogen pecS-1 and pecS-2 basis sets, respectively, and of about 30% for the rest of atoms. Thus, based on solid statistics, we will give the answer to the question of what benefits one can obtain in accuracy when lowering the contraction of the *p*-shell of our basis sets. This is an important question from a practical point of view since whatever computer power is accessible now, adding only one *p*-function leads to as many as three additional functions for each hydrogen and carbon atom, which results in a substantial increase in the overall basis set space in the case of large natural products.

## 2. Results and Discussion

### 2.1. Brief Notes on PEC-Generated Contraction Coefficients

The main idea of the PEC method [24] consists of the optimization of basis sets in relation to a certain molecular property provided that the least possible total molecular energy of fitting molecules is achieved. More specifically, the basis set exponents are randomly generated around the starting set via the Monte Carlo simulations. The generated arrays are verified whether they give the property under interest within a desired diapason or not. Of all sets that provide the property in the desired range, only one set is selected, namely, the one that gives the lowest energy. It should be mentioned that the optimization of the property under the energetic constraint represents a nonlinear problem with multiple solutions, which is not correctly solvable using standard optimization procedures based on the directed search, like numerical Newton-like methods [30]. In this sense, our PEC approach, based on Monte Carlo simulations, is more suitable for solving such optimization problems because it is not bound to find only a single extremum in the close vicinity of the starting point. 

The pecS-*n* (*n* = 1, 2) basis sets for hydrogen and carbon atoms presented in reference [22] were obtained via the PEC algorithm, viz., by minimizing the mean absolute deviations of the ^1^H and ^13^C NMR chemical shifts of several fitting molecules from their target values under the energetic constrain. Their original contraction schemes were chosen based on a minimally necessary number of functions capable of providing the near-zero contraction error. These contractions are very tight and close to that of Jensen’s contraction schemes of the pcS-*n* basis sets.

In this work, we have decided to release one *p*-function of both pecS-*n* (*n* = 1, 2) basis sets for hydrogen and carbon atoms and perform the optimization procedure for the whole set of the contraction coefficients for the *p*-shell via the PEC algorithm. This idea was inspired by constantly developing computer power and an observation made by Jensen [15], which consists of the *p*-shell that plays an important role in the paramagnetic term of shielding constants. It is worth noting that in the case of hydrogen atoms, releasing one *p*-function in both pecS-*n* (*n* = 1, 2) basis sets results in a fully uncontracted *p*-shell. Thus, further discussion of the optimization procedure is relevant to carbon atoms only.

New contraction coefficients for the *p*-shell were optimized using the DFT approach with the PBE0 exchange-correlation (XC) functional [27,28,29] in the gauge, including atomic orbitals (GIAO) formalism [31]. The PBE0 functional represents the model that has been used in our previous work [22] to generate both the exponents and the original contraction coefficients for the pecS-*n* basis sets. That is why it represents a reasonable choice in this work. 

The main procedure can briefly be described as the minimization of the contraction errors due to the *p*-shell contractions, provided the least possible molecular energy of the fitting molecules is achieved. The optimization of the contraction coefficients for the carbon basis sets of both levels was carried out using three fitting molecules, namely, acetylene (C_2_H_2_), ethylene (C_2_H_4_), and methane (CH_4_). The fitting molecules were chosen to be small in size and to provide a wide diversity of carbon shielding constants. 

The pecS-*n* basis set with varying *p*-shell contraction coefficients was set on the carbon atoms, while the originally contracted pecS-*n* basis set of the corresponding level was set on hydrogen atoms.

Thus, the optimization process of contraction coefficients can be represented as follows:

(1) $$\tilde{∆}=\frac{1}{3}\sum_{n=1}^{3}\left|{\tilde{\sigma }}_{n}-\sigma_{ideal,n}\right|\to min$$

(2) $$\sum_{n=1}^{3}{\tilde{E}}_{n}\to min$$

The ideal or target values of the carbon shielding constants ($\sigma_{ideal,n}$) of the fitting molecules were obtained using the pecS-*n* basis sets with fully uncontracted *p*-shells (and contracted *s*-shells), set on carbon atoms, and the original pecS-*n* basis sets on hydrogen atoms. The ideal values of *σ*(^13^C) are as follows: 108.06, 50.87, and 192.94 ppm for C_2_H_2_, C_2_H_4_, and CH_4_, respectively. The total molecular energy tolerance threshold was set to 10^−5^ Hartree. The final mean absolute error $\tilde{∆}$ of the carbon shielding constants of three fitting molecules relative to their ideal values did not exceed 0.01 ppm.

The pecS-*n* (*n* = 1, 2) basis sets for carbon atoms with newly optimized contraction coefficients of the *p*-shell are presented in the Supplementary Material. Detailed structures of modified and original pecS-*n* (*n* = 1, 2) basis sets together with their sizes and numerical estimations of the contraction depths and mean absolute and percentage contraction errors (MACEs, MAPCEs) are given in Table 1.

The mean absolute contraction errors (MACEs) were evaluated as the average absolute deviations of the ^1^H and ^13^C NMR shielding constants of three fitting molecules, calculated with either originally or newly contracted pecS-*n* basis sets, set on the NMR spectator atoms ($\sigma_{i,c}$, *i* = 1−3), from the values obtained with totally uncontracted (in all shells) pecS-*n* basis sets, set on the same atoms ($\sigma_{i,uc}$). The rest atoms were described in the originally contracted pecS-*n* basis sets. For the hydrogen shielding constants, $\sigma_{i,c}$ represent the values obtained with the pecS-*n* basis set with fully uncontracted *p*-shell. Thus, the MACE is expressed as follows:

(3) $$MACE=\frac{1}{3}\sum_{n=1}^{3}\left|\sigma_{i,c}-\sigma_{i,uc}\right|\;$$

The mean absolute percentage contraction errors (MAPCEs) were evaluated as the average relative deviations of the $\sigma_{i,c}$ from $\sigma_{i,uc}$, taken in an absolute value and expressed in the percentage terms:

(4) $$MAPCE=\frac{1}{3}\sum_{n=1}^{3}\left|\frac{\sigma_{i,c}-\sigma_{i,uc}}{\sigma_{i,uc}}\right|\times 100\%$$

As one can see from Table 1, releasing one *p*-function did not lead to a noticeable effect. To be more precise, the MACE of the pecS-1 basis set for hydrogen atoms does not change at all, keeping its value of ca. 0.03 ppm on going from the original to the newly contracted version. At the same time, the MACE provided by the pecS-2 basis set for hydrogen decreases only insignificantly, from ca. 0.03 to 0.01 ppm. In the case of carbon basis sets, releasing one *p*-function reduces the MACE almost in half (decreasing it from 0.39 to 0.18 ppm) for the pecS-1 basis set and almost negates the error (decreasing it from 0.45 to 0.05 ppm) for the pecS-2 basis set. However, the MACEs provided by the original carbon pecS-*n* basis sets are insignificant per se. Therefore, diminishing them next to nothing by releasing one *p*-function is expected to bring about only a minor effect on the overall accuracy. Moreover, the corresponding MAPCEs for the original and new contractions of carbon basis sets are also very small, being of, accordingly, 0.5 and 0.3% and 0.6 and 0.0% for the pecS-1 and pecS-2 basis sets, respectively. At this point, we expect only a small positive effect from less succinct contractions of the *p*-shell of the hydrogen and carbon pecS-*n* basis sets. However, this conclusion is to be validated on the extensive calculations of the ^1^H and ^13^C NMR chemical shifts.

### 2.2. Benchmark Calculations

To examine the performance of the pecS-*n* (*n* = 1, 2) basis sets, including both newly and originally contracted versions, we have carried out calculations of ^1^H and ^13^C NMR chemical shifts of 23 natural products at the GIAO-DFT level with PBE0 functional and compared the theoretical results with experiment. We have used the PBE0 functional, as it *hitherto* has been shown to be one of the best functionals for predicting the NMR chemical shifts of light nuclei, especially as applied to natural products [32]. Remarkably, Adamo and Barone [9] showed that the PBE0 functional is competitive with low-order perturbation post-HF techniques, such as the MP2 method, for well-behaved systems and gives significantly improved results in the presence of huge correlation effects. Now, PBE0 functional is one of the most popular functionals used in the calculations of NMR chemical shifts of a vast variety of molecules. Moreover, it is worth mentioning again that the PBE0 functional was used by us when generating the pecS-*n* basis sets for the atoms of 1–2 periods. That is why the PBE0 represents the best choice when calculating the proton and carbon NMR chemical shifts with the pecS-*n* basis sets, and there is no point in considering any other functionals. However, it could be assumed that the tendencies observed in this work shall repeat themselves because in the previous paper [22], we have shown that the MAEs for ^1^H and ^13^C chemical shifts, calculated using the PBE0, B97-2, B3LYP, HCTH, B3PW91, and OLYP functionals in combination with our basis sets, occurred to be not much of a difference, if any.

The considered natural products are produced by living organisms such as marine sponges, fungi, and plants. Practically all of them represent very important species with potential biological activity. The considered compounds include 10′-hydroxyusambarensine (**1**), 2-2-di(3-indolyl)-3-indolone (**2**), acantholactam (**3**), alasmontamine A (**4**), anabsinthin (**5**), asperlicin (**6**), chetomin (**7**), diosgenin-3-*O*-*β*-D-glucopyranoside (**8**), itoaic acid (**9**), jaspamide Q (**10**), korundamine A (**11**), limonin (**12**), matopensine (**13**), mulberrofuran G (**14**), 1*α*,8*β*,9*β*,14*α*,15*β*-pentaacetoxy-3*β*-benzoyloxy-7-oxojatropha-5,12-diene (**15**), pedunculagin (**16**), phyllaemblicin B (**17**), physalin D (**18**), procyanidin B2 (**19**), strychnine (**20**), strychnobaillonine (**21**), terrequinone A (**22**), and viomycin (**23**). 

In particular, 10′-hydroxyusambarensine (**1**) was isolated by Frédérich et al. [33] as a new derivative of usambarensine [34], a plant alkaloid extracted from the roots of *Strychnos usambarensis* growing in Central Africa. Compound (**1**) was presented as a new antiplasmodial bisindole alkaloid possessing potential antimalarial activity. The structure of compound **1** was deduced from UV spectra, IR spectrum, and, more importantly for us, from the analysis of ^1^H and ^13^C NMR spectra supported by the data obtained from 2D NMR experiments such as ^1^H-^1^H COSY, HMQC, HMBC, and NOESY [33].

2-2-di(3-indolyl)-3-indolone (**2**) was isolated by Kobayshi et al. [35] from the Okinawan marine sponge *Hyrtios altum*, though originally it was reported as the oxidation product of indole by Capdevielle and Maumy [36]. Compound **2** showed antibacterial activity against *Bacillus cereus* [37], an anaerobic, toxin-producing Gram-positive bacterium found in soil, vegetation, and food. Its structure was validated via ^1^H and ^13^C NMR spectroscopy together with liquid chromatography–mass spectrometry (LC-MS) and high-resolution mass spectrometry (HRMS) methods [37].

Acantholactam (**3**) represents a manzamine alkaloid isolated from the marine sponge *Acanthostrongylophora ingens* from Indonesian waters [38]. Compound **3** was shown to exhibit rather low cytotoxic activity, inhibitory activity against the proteasome, and inhibitory activity against the accumulation of cholesterol esters in macrophages compared to other products isolated from *Acanthostrongylophora ingens* [38]. Therefore, its possible application is yet to be found. The structure of compound **3** was confirmed via ^1^H and ^13^C NMR spectra, supported by the 2D NMR spectra, obtained via COSY, HOHAHA (or TOCSY), HSQC, and HMBC experiments [38].

Alasmontamine A (**4**) is a tetrakis monoterpene indole alkaloid, which was isolated from the leaves of *Tabernaemontana elegans* [39]*,* a tropical plant found in Indonesia. Compound **4** exhibits moderate cell growth inhibitory activity against HL-60 cells. Structural studies of Alasmontamine A were carried out using ^1^H and ^13^C NMR, COSY, HOHAHA, HSQC, and HMQC spectroscopy [39].

Anabsinthin (**5**) is sesquiterpene lactone that can be extracted from the aerial parts of *Artemisia absinthium* L., commonly known as wormwood, which is a yellow-flowering, perennial plant distributed throughout various parts of Europe and Siberia and used for the antiparasitic effects, as well as to treat anorexia and indigestion. Structural elucidation of anabsinthin was performed by Aberham et al. [40] based on High-Performance Liquid Chromatography (HPLC) in combination with ^1^H and ^13^C NMR studies.

Asperlicin (**6**) is a quinazoline alkaloid that was isolated from *Aspergillus alliaceus* [41], a species of fungus in the genus *Aspergillus*. Asperlicin is a competitive cholecystokinin (CCK) antagonist, which is highly selective for peripheral CCK receptors relative to brain CCK and gastrin receptors. High-quality ^1^H and ^13^C NMR structural studies for asperlicin were carried out by Sun et al. [42].

Chetomin (**7**) represents an antibiotic metabolite produced from the fungi species *Chaetomium cochliodes* of the family *Chaetomiaceae* [43,44]. The originally proposed isolation procedures [43,44] were modified by Brewer et al. [45], who also performed comprehensive high-resolution NMR studies of compound **7**.

Diosgenin-3-*O*-*β*-D-glucopyranoside (**8**) is a steroidal saponin that has been found in *Trillium tschonoskii Maxim Root* and has diverse biological activities, including the decrease in neuronal damage in a rat model of spinal cord injury, the decrease in serum levels of glucose, insulin, and triglycerides in diabetic mice, and many others [46,47,48,49,50]. The structure of diosgenin-3-*O*-*β*-D-glucopyranoside was thoroughly investigated with IR, Fast Atom Bombardment Mass Spectrometry (FABMS), and high-resolution ^1^H and ^13^C NMR spectroscopy by Feng et al. [51]. 

Itoaic acid or 2*β*,11*β*-dihydroxy-3,4-secofriedelolactone-27-oic acid (**9**) represents a rare naturally occurring triterpenoid of 3,4-seco-friedelolactone skeleton with potential anti-inflammatory activity against COX-2, which was isolated from *Flacouritaceae* plants. The isolation procedure and structural studies of compound **9**, carried out using MS, 1D, and 2D NMR techniques, were reported by Chai et al. [52]. 

Jaspamide Q (**10**) was isolated from the marine sponge *Jaspis splendens* collected in Kalimantan (Indonesia). The structure of compound **10** was unambiguously elucidated via the 1D and 2D NMR spectroscopy by Ebada et al. [53]. In addition, compound **10** was established to manifest significant inhibitory activity against the growth of the mouse lymphoma (L5178Y) cell line [53]. 

Korundamine A (**11**) is a unique heterodimeric naphthylisoquinoline alkaloid comprising two different monomeric biaryl halves. Hallock et al. [54] isolated Korundamine A from the Cameroonian tropical liana *Ancistrocladus korupensis* and carried out comprehensive structural studies using ^1^H and ^13^C NMR spectroscopy, including 2D HMQC and HMBC techniques. Korundamine A was shown to possess anticytopathic activity against HIV-l and antimalarial activity against *Plasmodium falciparum*. 

Limonin (**12**) is a natural tetracyclic triterpenoid compound that exists in *Euodia rutaecarpa* (Juss.) Benth., *Phellodendron chinense* Schneid., and *Coptis chinensis* Franch. Limonin has a wide spectrum of pharmacological effects, including anti-cancer, anti-inflammatory, analgesic, anti-bacterial and anti-virus, anti-oxidation, and liver protection properties [55]. The isolation procedure of limonin from the bark of *Phellodendron amurense*, together with its NMR study, was presented by Min et al. [56].

Matopensine (**13**) is a symmetrical bisindole alkaloid, which is extracted from the roots of *Strychnos matopensis* and *Strychnos kasengaensis*, plants from eastern Africa. Matopensine-type alkaloids were found to exhibit potent and selective activities against *Plasmodium* [57]. The structure of matopensine has been elucidated using NMR spectroscopy by Massiot et al. [58,59].

Mulberrofuran G (**14**) is a Diels–Alder-type adduct derived from mulberrofuran C, which can be isolated from the ethyl acetate extract of the root bark of cultivated mulberry tree. Intravenous injection of mulberrofuran G causes a marked depressor effect. The derivation of mulberrofuran G from mulberrofuran C, together with its NMR study, was presented by Fukai et al. [60].

1*α*,8*β*,9*β*,14*α*,15*β*-pentaacetoxy-3*β*-benzoyloxy-7-oxojatropha-5,12-diene (**15**) was isolated from the white latex of *Pedilanthus tithymaloides*. Compound **15** showed notable antiplasmodial activity and antimycobacterial activity against *Mycobacterium tuberculosis*. The isolation procedure and structural identification based on 2D NMR and MS investigations were reported on by Mongkolvisut and Sutthivaiyakit [61]. 

Pedunculagin (**16**) is a hydrolyzable tannin found in the pericarp of pomegranates (*Punica granatum*), in plants in the order Fagales such as walnuts (*Juglans regia*), in leaves of *Camellia pachyandra* Hu., and in some other species. Pedunculagin is an ellagitannin, and ellagitannins are known to exhibit antioxidant and anti-inflammatory bioactivity, facilitating the suppression of disease initiation and progression [62,63]. The first biomimetic synthesis of pedunculagin (**1**) was presented by Feldman et al. [64] and its isolation from the leaves of *Camellia pachyandra* Hu. supported by ^1^H, ^13^C, and 2D-NMR (including ^1^H-^1^H COSY, HSQC, and HMBC) studies was presented by Gao et al. [65]. 

Phyllaemblicin B (**17**) is a natural product found in *Phyllanthus emblica*, a shrub or tree growing in subtropical and tropical areas of the People’s Republic of China, India, Indonesia, and the Malay Peninsula. Phyllaemblicin B was found to inhibit *Coxsackie virus B3*-induced apoptosis and myocarditis [66]. The procedure of isolation of Phyllaemblicin B supported via ^1^H and ^13^C NMR measurements was reported on by Zhang et al. [67].

Physalin D (**18**) is a fraction from aerial parts of *Physalis angulate*, known in Brazil as *camapu*, being a branched annual shrub that belongs to the *Solanaceae* family. Extracts from this plant have been used in traditional folk medicine to treat tumors. Physalin D per se was found to exhibit an inhibitory activity against *Mycobacterium tuberculosis*. The procedure of isolation of Phyllaemblicin B supported via ^1^H and ^13^C NMR measurements was carried out by Januário et al. [68].

Procyanidin B2 (**19**) is a flavonoid that was extracted from hawthorn. Procyanidin B2 manifests an antioxidant and anti-inflammatory activity [69]. A heteronuclear NMR study of Procyanidin B2 was undertaken by Khan et al. [70] in order to reliably establish its structure.

Strychnine (**20**) is a highly toxic alkaloid that is found in *Strychnos nux-vomica* (*Loganiaceae*). It causes excitation of all parts of the central nervous system, with a characteristic motor pattern. Strychnine is a competitive antagonist at inhibitory neurotransmitter glycine receptors in the spinal cord, brain stem, and higher centers. It thus increases neuronal activity and excitability, leading to increased muscular activity [71]. Strychnine was used by Martin et al. [72] as a model compound in studying long-range correlations observed with the earlier reported ACCORD-HMBC pulse sequence using both static and accordion optimization of the long-range coupling delay.

Strychnobaillonine (**21**) is an unsymmetrical bisindole alkaloid found in the roots of liana *Strychnos Icaja*, mainly used by local populations of Africa as an arrow or ordeal poison and as a means of the treatment of skin diseases and chronic, persistent malaria. In particular, strychnobaillonine showed potent activity against the chloroquine-sensitive 3D7 strain of *Plasmodium falciparum.* Its structure was defined via detailed ^1^H, ^13^C NMR, HSQC, COSY, NOESY, HMBC, and HRESIMS spectroscopic analyses performed by Tchinda et al. [73].

Terrequinone A (**22**) is a cytotoxic metabolite isolated from extracts of *Aspergillus Terreus*. He et al. [74] reported on the isolation procedure and structure elucidation of Terrequinone A using ^1^H and ^13^C NMR experiments and investigated its cytotoxicity toward a panel of four human cancer cell lines.

Viomycin (**23**) is a cyclic peptide antibiotic that has been used in the treatment of tuberculosis. Hawkes et al. [75] carried out ^1^H, ^13^C, and ^15^N NMR analysis of viomycin, using it as a model compound in the investigation of intramolecular hydrogen bonding in peptides.

**Scheme 1:**
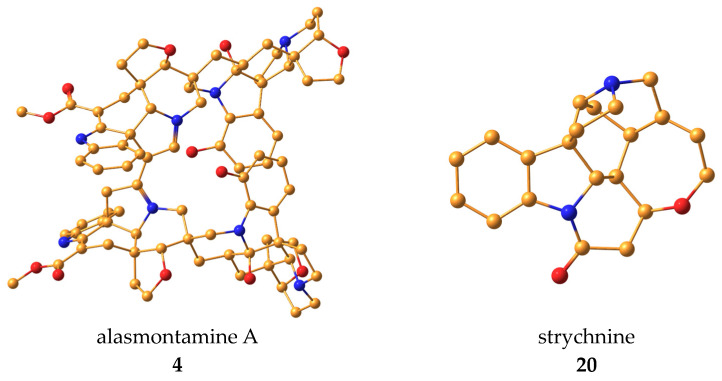
Alasmontamine A (**4**) and strychnine (**20**), as the representatives of the series of considered compounds.

Aside from bearing a pronounced bioactivity, compounds **1**–**23** also pose a challenging computational task due to their considerable sizes and complex electron structure. Given that NMR data for compounds **1**–**23** are available, these represent ideal candidates for rigorous benchmarking of our recently proposed pecS-*n* basis sets. Indeed, the considered compounds include only two moderate molecules, namely, strychnine (**20**) and 2-2-di(3-indolyl)-3-indolone (**2**) consisting of 47 and 45 atoms, respectively, while the rest are bulky molecules containing 60 to 195 atoms, with half of them consisting of more than 80 atoms. The largest one is alasmontamine A (**4**), which consists of as many as 195 atoms. For example, alasmontamine A (**4**) and strychnine (**20**) are presented in Scheme 1.

All considered compounds possess multiple degrees of conformational freedom, therefore, very accurate protocols for calculating their ^1^H and ^13^C NMR chemical shifts would inevitably involve a multistep procedure, which implies obtaining accurate Boltzmann conformer weights, followed by NMR calculations for the individual conformers. However, large natural products potentially have dozens of practically relevant conformers. Thus, an approach that involves the NMR calculations for all of them becomes very demanding. Therefore, it can be noticed that when dealing with large molecules, it is frequently the case that one calculates the NMR chemical shifts for the lowest-energy conformer only [76,77,78], while a proper conformational averaging is still the exception rather than the rule [1]. In this work, we also have performed the calculations in an ordinary manner, considering only the lowest-energy conformer in each case, as we indeed deal with as many as 23 natural products of considerable sizes, each of which presents a computational challenge per se. 

Thus, we have carried out six series of calculations of ^1^H and ^13^C NMR shielding constants of compounds **1**–**23** using different basis set schemes. In the first four series of calculations, Jensen’s pcS-1 and pcS-2 and our original pecS-1 and pecS-2 basis sets were used on all atoms. The last two series of calculations were performed with newly contracted pecS-1 and pecS-2 basis sets (pecS-1 mod and pecS-2 mod) used on hydrogen and carbon atoms, whereas the rest of atoms were presented, accordingly, in the original pecS-1 and pecS-2 basis sets. In view of the absence of pecS-*n* basis sets for sulfur, the corresponding pcS-*n* basis sets were used for the atoms of this type.

Calculated shielding constants were transformed to the chemical shifts scale via the linear regression models, derived from the mapping of the calculated shielding constants (*σ_calc_*) onto the experimental chemical shifts (*δ_exp_*): *δ_exp_* = *Aσ_calc_* + *B*, where *A* represents the slope (the tangent of the line angle), and *B* represents the intercept of the model with the *δ_exp_*-axis. This was followed by the evaluation of the scaled chemical shifts (*δ_scaled_*) that represent the values of chemical shifts restored from the *σ*_calc_ via the established linear models. 

The measure of accuracy in this work is the Corrected Mean Absolute Errors (CMAEs) calculated between *δ_scaled_* and the corresponding experimental values:

(5) $$CMAE=\frac{1}{N}\sum_{n=1}^{N}\left|\delta_{scaled,n}-\delta_{exp,n}\right|\;$$

where *N* is the total number of chemical shifts (overall, 713 ^1^H and 767 and ^13^C NMR chemical shifts were calculated).

**Figure 1:**
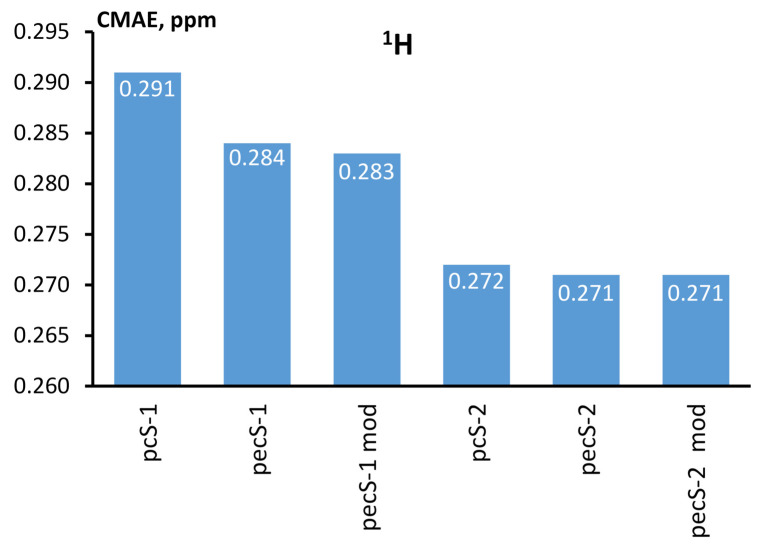
The CMAEs (in ppm) of scaled proton chemical shifts against experiment, evaluated over compounds **1**–**23**, depending on basis sets used in proton shielding calculations.

**Figure 2:**
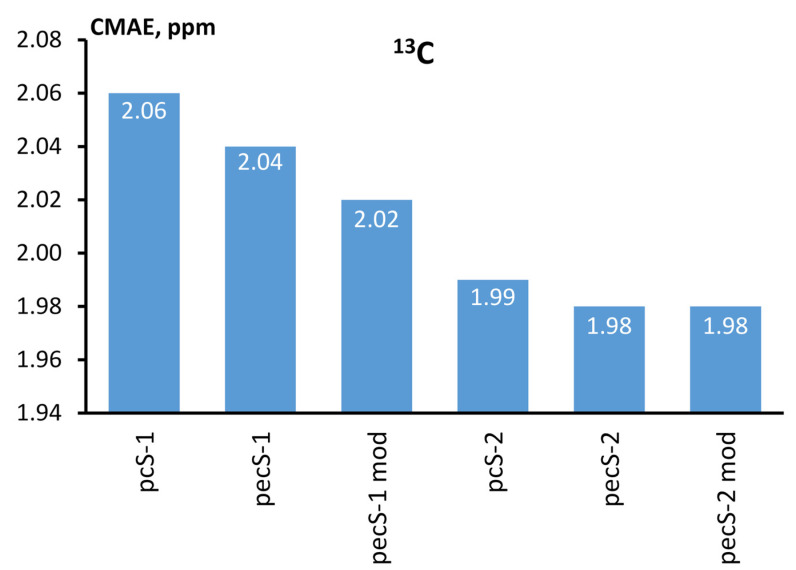
The CMAEs (in ppm) of scaled carbon chemical shifts against experiment, evaluated over compounds **1**–**23**, depending on basis sets used in carbon shielding calculations.

The CMAEs evaluated for scaled ^1^H and ^13^C NMR chemical shifts of the whole series of compounds **1**–**23**, obtained from the shielding constants calculated in each of the six basis set schemes described above, against the experiment are shown in Figure 1 and Figure 2. The parameters of each linear regression model are given below in Table 2. 

All calculated ^1^H and ^13^C shielding constants together with scaled chemical shifts retrieved from linear regression models and experimental chemical shifts are given in Tables S1–S4 of Supplementary Material.

Roughly speaking, all considered basis sets give accuracies around 0.28 and 2 ppm for proton and carbon NMR chemical shifts, respectively. In spite of sufficiently favorable accuracies, there are some examples of noticeably large deviations for scaled values of ^1^H and ^13^C chemical shifts from their experimental values (see, for example, the calculated scaled proton chemical shifts of 3.06, 3.09, and 3.11 ppm in compound 1 against its experimental value of 4.38 ppm in Table S2, or calculated scaled carbon chemical shifts of 140.4, 142.0, and 141.3 ppm of compound 1 against its experimental value of 135.2 ppm (Table S3), etc.). Apparently, such outliers can be explained, in the first place, with the omitted factor of vibrational corrections. On average, the rovibrational effects are known to provide as much of a value as the solvent effects do in the case of proton and carbon chemical shifts, and this factor cannot be said to be systematic; hence, it cannot be alleviated when applying linear regression analysis. For today, in the case of large real-life compounds consisting of 100–200 atoms, this factor cannot be taken into account within reasonable computational efforts, as it requires determining the effective (vibrationally averaged) geometry with the calculation of the cubic force field and the calculation of the second derivatives of the shielding constants using the numerical differentiation with regard to the normal coordinates at the effective geometry. Thus, one should bear in mind that the obtained favorable statistics was built on the data that is not deprived of noticeable outliers, and it is an encouraging fact that without abandoning such largely deviating values, the general CMAEs are very moderate.

Going into details, one can see from Figure 1 and Figure 2 that the pecS-*n* basis sets demonstrate a better accuracy in both cases compared to the commensurate Jensen’s basis sets, pcS-*n*. 

**Figure 3:**
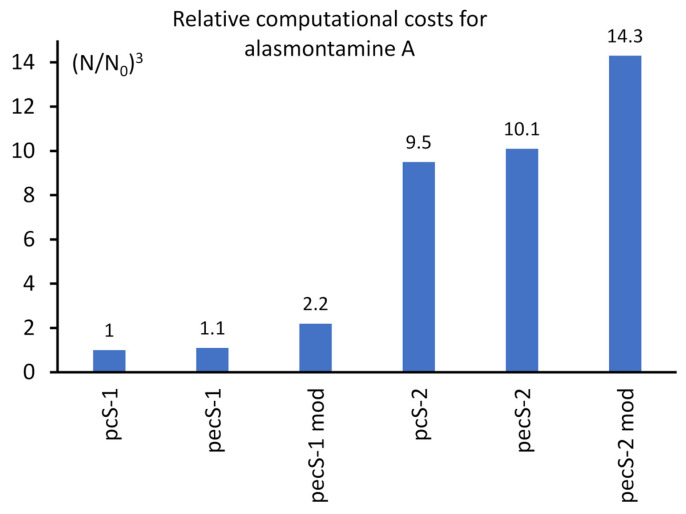
Relative computational DFT costs for alasmontamine A.

Now, we also can give the answer to one of the main questions posed in the beginning: is it worthwhile to apply a less succinct contraction of the *p*-shell to our pecS-*n* basis sets? From Figure 1 and Figure 2, it is clearly seen that releasing one *p*-function in the pecS-1 basis sets gives a benefit in accuracy of about 0.001 and 0.02 ppm in CMAEs for hydrogen and carbon chemical shifts, respectively, while the same action applied to the pecS-2 basis set for both atoms gives nothing at all. Thus, we confidently can say that a less succinct contraction of the *p*-shell of our pecS-*n* basis sets does not make sense. This fact indicates high-quality contraction of our original pecS-*n* basis sets, such that even releasing one *p*-function has no effect on accuracy. At that, the computational costs or the formal operational costs for modern implementations of the DFT method increase significantly on going from pecS-*n* to pecS-*n* mod version. The example for alasmontamine A is given below in Figure 3. Therein, N represents the total number of contracted basis set functions participating in the NMR calculation of alasmontamine A with different basis sets, with N_0_ being the total number of contracted basis set functions for the case of smallest pcS-1 basis set (this equals 2223). The computational costs of modern DFT methods can roughly be estimated as N^3^, with N being the total number of basis sets functions. Therefore, the ratio (N/N_0_)^3^ depicted in Figure 3 represents the estimation of the characteristic number of operations algorithmically executing via the DFT method when applying one of the considered basis sets in relation to the number of operations needed for the calculation with the pcS-1 basis set.

**Figure 4:**
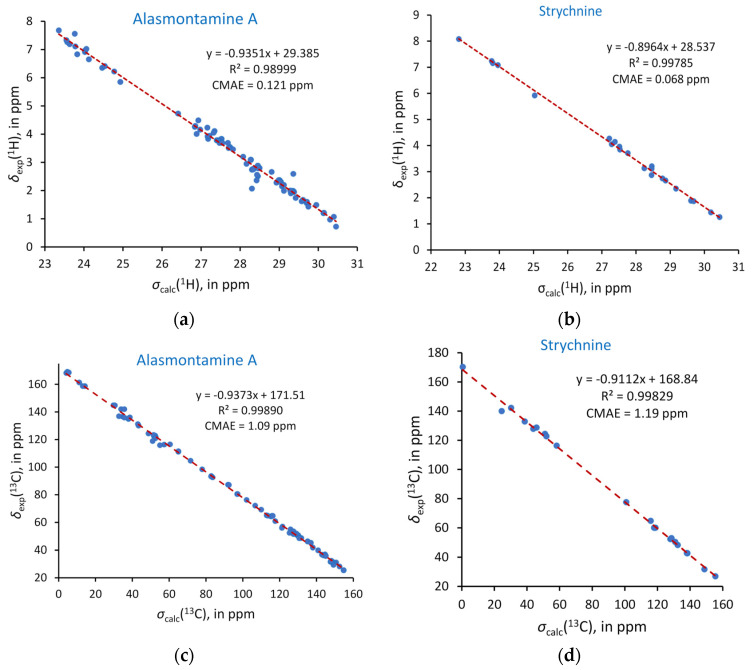
The correlation plots between the proton and carbon shielding constants calculated at the GIAO-DFT(PBE0)/pecS-2 level against the corresponding experimental chemical shifts: (**a**) plot for protons of alasmontamine A; (**b**) plot for protons of strychnine; (**c**) plot for carbons of alasmontamine A; (**d**) plot for carbons of strychnine.

Overall, we can conclude that our pecS-*n* basis sets have successfully passed the first austere test on real-life molecules and demonstrated very good accuracy in the calculations of proton and carbon chemical shifts of a vast variety of bulky natural products, carried out using the GIAO-DFT method, even without accounting for Boltzmann averaging. To demonstrate the accuracy provided by the pecS-2 basis set, we showed in Figure 4 the correlation plots between the proton and carbon shielding constants calculated at the GIAO-DFT(PBE0) level with the pecS-2 basis set against the corresponding experimental chemical shifts for two representative compounds, alasmontamine A (**4**) and strychnine (**20**), depicted on Scheme 1 (*vide supra*).

## 3. Materials and Methods

Geometry optimizations of three fitting molecules (used for the optimizations of contraction coefficients) were performed in the gas phase at the DFT(PBE0) [27,28,29] level of theory using the pc-3 basis set [16,17] on all atoms via the Dalton program [79]. The optimizations of contraction coefficients were performed using the PEC algorithm [24]. The calculations of shielding constants of three fitting molecules, involved in the PEC optimization process or in the estimations of contraction errors, were performed at the DFT(PBE0) level of theory in the gauge, including atomic orbitals (GIAO) formalism [31] in the Dalton program. 

The initial conformational search of compounds **1**–**23** was carried out using the OPLS3 force field in the liquid phase of a specific solvent, employing the MacroModel module implemented in the Schrödinger Maestro 11.5 package [80]. The first-step geometries of the lowest-energy conformers of each compound were then reoptimized at the DFT level of theory with the Minnesota M06-2X exchange-correlation functional [81] using the cc-pVTZ basis set for hydrogen and carbon atoms [12] and aug-cc-pVTZ for nitrogen, oxygen and sulfur atoms [12,82,83], using the Gaussian 09 code [84]. The optimizations were performed taking into account the solvent effects using the integral equation formalism polarizable continuum model (IEF-PCM) [85,86], parametrized for a particular solvent mentioned in the article with the corresponding experimental data. The equilibrium geometries of compounds **1**–**23** are available in the Supplementary Material. 

All benchmark calculations of shielding constants were carried out at the GIAO-DFT theory with exchange–correlation functional PBE0 via the IEF-PCM using the pcS-1, pcS-2 [15], pecS-1, and pecS-2 [22] basis sets, including the modified versions of the latter two, in the Gaussian 09 program.

## 4. Conclusions

In this paper, we have performed the first challenging test of our previously proposed pecS-*n* (*n* = 1, 2) basis sets on the example of 23 real-life biologically active natural products. The pecS-*n* basis sets have successfully passed this austere test, demonstrating very good accuracy in the calculations of 713 ^1^H and 767 ^13^C chemical shifts, carried out at the GIAO-DFT(PBE0) level of theory. The accuracy reached can be expressed in terms of the corrected mean absolute errors (CMAEs) as 0.284 and 0.271 ppm for hydrogen chemical shifts and as 2.04 and 1.98 ppm for carbon chemical shifts, for the pecS-1 and pecS-2 basis sets, respectively. 

In this paper, we also have proposed new alternative contraction schemes for our pecS-*n* (*n* = 1, 2) basis sets by releasing one function in their *p*-shells and reoptimizing the contraction coefficients for the whole *p*-shell using the property-energy consistent (PEC) method. The performance of the pecS-*n* (*n* = 1, 2) basis sets with new contraction of the *p*-shell was also tested on the calculations of proton and carbon chemical shifts of all 23 natural products. It was found that the new contraction of the pecS-1 basis sets gives the benefit in accuracy of only 0.001 and 0.02 ppm in CMAE terms for hydrogen and carbon chemical shifts, respectively, while the new contraction of the pecS-2 basis set for both atoms gives no advantage in accuracy at all. Thus, a less succinct contraction of the *p*-shell of our pecS-*n* basis sets does not make sense. This points to the fact that the original contraction of the pecS-*n* basis sets is of high quality. Therefore, it does not need any corrections, and the pecS-*n* basis sets as they are now can be of great practical use in the large-scale calculations of ^1^H and ^13^C NMR chemical shifts of real-life organic compounds.

## Figures and Tables

**Table 1:** Comparative analysis of the original and new contraction schemes for the *p*-shell of the pecS-*n* (*n* = 1, 2) basis sets.

Atom	Basis Set	[Uncontracted| Contracted] Composition	Detailed Contraction of the *p*-Shell	Number of Uncontracted Basis Set Functions, *N*_uc_	Number of Contracted Basis Set Functions, *N*_c_	Cont. Depth (*N*_uc_ − *N*_c_)/*N*_uc_, %	MACE, in ppm	MAPCE, in %
H	original pecS-1	[4*s*2*p*|2*s*1*p*]	2*p* → (2)	10	5	50	0.03	0.1
	modified pecS-1	[4*s*2*p*|2*s*2*p*]	2*p* → (1, 1)	10	8	20	0.03	0.1
	original pecS-2	[6*s*3*p*1*d*|3*s*2*p*1*d*]	3*p* → (2, 1)	20	14	30	0.03	0.1
	modified pecS-2	[6*s*3*p*1*d*|3*s*3*p*1*d*]	3*p* → (1, 1, 1)	20	17	15	0.01	0.0
C	original pecS-1	[7*s*5*p*1*d*|4*s*3*p*1*d*]	5*p* → (3, 1, 1)	27	18	33	0.39	0.5
	modified pecS-1	[7*s*5*p*1*d*|4*s*4*p*1*d*]	5*p* → (2, 1, 1, 1)	27	21	22	0.18	0.3
	original pecS-2	[10*s*7*p*2*d*1*f*|5*s*4*p*2*d*1*f*]	7*p* → (4, 1, 1, 1)	48	34	29	0.45	0.6
	modified pecS-2	[10*s*7*p*2*d*1*f*|5*s*5*p*2*d*1*f*]	7*p* → (3, 1, 1, 1, 1)	48	37	23	0.05	0.0

**Table 2:** The parameters of linear regression models *δ_exp_* = *Aσ_calc_* + *B* obtained by mapping the shielding constants calculated at the GIAO-DFT(PBE0) level with different basis sets (*σ_calc_*) onto the experimental values (*δ_exp_*) taken from different sources ^1.^.

	^1^H NMR			^13^C NMR		
Basis Set	*A*	*B*	R^2^	*A*	*B*	R^2^
pcS-1	−0.92079	29.32047	0.96457	−0.95991	179.6763	0.99657
pecS-1	−0.94085	30.03978	0.96750	−0.93108	173.1887	0.99660
pecS-1 mod	−0.93398	29.88718	0.96765	−0.94531	175.7673	0.99663
pcS-2	−0.90872	28.87741	0.96807	−0.93833	173.0559	0.99674
pecS-2	−0.91012	28.87941	0.96850	−0.93670	172.2379	0.99676
pecS-2 mod	−0.91125	28.94657	0.96860	−0.94024	172.9650	0.99676

^1^ For the experimental values, see the references: 1 [33], 2 [37], 3 [38], 4 [39], 5 [40], 6 [42], 7 [45], 8 [51], 9 [52], 10 [53], 11 [54], 12 [56], 13 [58,59], 14 [60], 15 [61], 16 [65], 17 [67], 18 [68], 19 [70], 20 [72], 21 [73], 22 [74], and 23 [75].

## Data Availability

Data sharing is not applicable.

## Supplementary Materials

The following supporting information can be downloaded at: https://www.mdpi.com/article/10.3390/ijms241914623/s1.
