# Supplementary material for: On the Efficiency of the Density Functional Theory (DFT)-Based Computational Protocol for 1H and 13C Nuclear Magnetic Resonance (NMR) Chemical Shifts of Natural Products: Studying the Accuracy of the pecS-n (n = 1, 2) Basis Sets
Source: Int J Mol Sci. 2023 Sep 27;24(19):14623. doi: 10.3390/ijms241914623 (PMC10572908; doi:10.3390/ijms241914623)
Supplement: Supplementary file 1 [file ijms-24-14623-s001.zip › ijms-2612933-supplementary.pdf]

# *Supplementary Material to Article*

## On the efficiency of the DFT-based computational protocol for $^{1}H$ and $^{13}C$ NMR chemical shifts of natural products: studying the accuracy of the pecS-*n* (*n* = 1, 2) basis sets

*Authors: Yuriy Yu. Rusakov, Valentin A. Semenov and Irina L. Rusakova*

**Contents**

**Basis sets**

The pecS-1 basis set for carbon atom with newly optimized contraction coefficients of the p-shell (Dalton format) ........ 3

The pecS-1 basis set for carbon atom with newly optimized contraction coefficients of the p-shell (Gaussian format)........ 4

The pecS-2 basis set for carbon atom with newly optimized contraction coefficients of the p-shell (Dalton format) ........ 5

The pecS-2 basis set for carbon atom with newly optimized contraction coefficients of the p-shell (Gaussian format)........ 6

**Equilibrium Geometries**

10’-Hydroxyusambarensine (**1**) ........ 7

2-2-Di(3-indolyl)-3-indolone (**2**) ........ 8

Acantholactam (**3**)........ 9

Alasmontamine A (**4**) ........ 11

Anabsinthin (**5**)........ 16

Asperlicin (**6**)........ 18

Chetomin (**7**)........ 19

Diosgen-in-3-O-β-D-glucopyranoside (**8**) ........ 21

Itoaic acid (**9**) ... 24
Jaspamide Q (**10**) ... 26
Korundamine A (**11**) ... 28
Limonin (**12**) ... 30
Matopensine (**13**) ... 32
Mulberrofuran G (**14**) ... 35
1α,8β,9β,14α,15β-Pentaacetoxy-3β-benzoyloxy-7-oxojatropha-5,12-diene (**15**) 36
Pedunculagin (**16**) ... 39
Phyllaemblicin B (**17**) ... 41
Physalin D (**18**) ... 43
Procyanidin B2 (**19**) ... 45
Strychnine (**20**) ... 47
Strychnobaillonine (**21**) ... 48
Terrequinone A (**22**) ... 50
Viomycin (**23**) ... 52
**Table S1.** Calculated $^{1}$H NMR shielding constants, together with scaled and experimental chemical shifts – the GIAO-DFT(PBE0) calculations with the first-level basis sets. ... 54
**Table S2.** Calculated $^{1}$H NMR shielding constants, together with scaled and experimental chemical shifts – the GIAO-DFT(PBE0) calculations with the second-level basis sets ... 68
**Table S3.** Calculated $^{13}$C NMR shielding constants, together with scaled and experimental chemical shifts – the GIAO-DFT(PBE0) calculations with the first-level basis sets ... 83
**Table S4.** Calculated $^{13}$C NMR shielding constants, together with scaled and experimental chemical shifts – the GIAO-DFT(PBE0) calculations with the second-level basis sets ... 99

**The pecS-1 basis set for carbon atom with newly optimized contraction coefficients of the *p*-shell (Dalton format)**

```
$ C
a 6
$ s functions
  7   4   0
 1.158704E+03  6.1043E-03  1.0143E-03  0.00000000  0.00000000
 1.744210E+02  4.4530E-02  8.1051E-03  0.00000000  0.00000000
 4.064338E+01  1.8738E-01  3.1628E-02  0.00000000  0.00000000
 1.141525E+01  4.7151E-01  1.1437E-01  0.00000000  0.00000000
 3.439092E+00  4.3067E-01  1.1469E-01  0.00000000  0.00000000
 3.002996E-01  0.00000000  0.00000000  1.00000000  0.00000000
 5.973462E-02  0.00000000  0.00000000  0.00000000  1.00000000
$ p functions
  5   4   0
 3.880432E+01  3.8015E-03  0.00000000  0.00000000  0.00000000
 6.201621E+00  4.3304E-02  0.00000000  0.00000000  0.00000000
 1.332637E+00  0.00000000  1.00000000  0.00000000  0.00000000
 3.106991E-01  0.00000000  0.00000000  1.00000000  0.00000000
 8.352061E-02  0.00000000  0.00000000  0.00000000  1.00000000
$ d functions
  1   1   0
 4.584126E-01  1.00000000
```

**The pecS-1 basis set for carbon atom with newly optimized contraction coefficients of the *p*-shell (Gaussian format)**

```
C     0
S    5   1.00
1.158704E+03          6.1043E-03
1.744210E+02          4.4530E-02
4.064338E+01          1.8738E-01
1.141525E+01          4.7151E-01
3.439092E+00          4.3067E-01
S    5   1.00
1.158704E+03          1.0143E-03
1.744210E+02          8.1051E-03
4.064338E+01          3.1628E-02
1.141525E+01          1.1437E-01
3.439092E+00          1.1469E-01
S    1   1.00
3.002996E-01         0.1000E+01
S    1   1.00
5.973462E-02         0.1000E+01
P    2   1.00
3.880432E+01          3.8015E-03
6.201621E+00          4.3304E-02
P    1   1.00
1.332637E+00          0.1000E+01
P    1   1.00
3.106991E-01         0.1000E+01
P    1   1.00
8.352061E-02         0.1000E+01
D    1   1.00
4.584126E-01         0.1000E+01
****
```

**The pecS-2 basis set for carbon atom with newly optimized contraction coefficients of the *p*-shell (Dalton format)**

```
$ C
a 6
$ s functions
  10    5    0
  7.811885E+03  5.7075E-04  1.0361E-04  0.00000000  0.00000000  0.00000000
  1.168486E+03  4.3473E-03  7.7592E-04  0.00000000  0.00000000  0.00000000
  2.735860E+02  2.1213E-02  3.9293E-03  0.00000000  0.00000000  0.00000000
  7.954210E+01  7.9825E-02  1.4569E-02  0.00000000  0.00000000  0.00000000
  2.613539E+01  2.2806E-01  4.6979E-02  0.00000000  0.00000000  0.00000000
  9.217097E+00  4.3136E-01  9.9114E-02  0.00000000  0.00000000  0.00000000
  3.390089E+00  3.5137E-01  1.5321E-01  0.00000000  0.00000000  0.00000000
  1.045814E+00  0.00000000  0.00000000  1.00000000  0.00000000  0.00000000
  3.150044E-01  0.00000000  0.00000000  0.00000000  1.00000000  0.00000000
  1.155406E-01  0.00000000  0.00000000  0.00000000  0.00000000  1.00000000
$ p functions
   7    5    0
  1.844063E+02  2.5812E-04  0.00000000  0.00000000  0.00000000  0.00000000
  3.098346E+01  3.5919E-03  0.00000000  0.00000000  0.00000000  0.00000000
  7.830746E+00  2.4201E-02  0.00000000  0.00000000  0.00000000  0.00000000
  2.308071E+00  0.00000000  1.00000000  0.00000000  0.00000000  0.00000000
  7.525987E-01  0.00000000  0.00000000  1.00000000  0.00000000  0.00000000
  2.530275E-01  0.00000000  0.00000000  0.00000000  1.00000000  0.00000000
  8.574535E-02  0.00000000  0.00000000  0.00000000  0.00000000  1.00000000
$ d functions
   2    2    0
  1.566238E+00  1.00000000  0.00000000
  3.508371E-01  0.00000000  1.00000000
$ f functions
   1    1    0
  1.148503E+00  1.00000000
```

**The pecS-2 basis set for carbon atom with newly optimized contraction coefficients of the *p*-shell (Gaussian format)**

```
C 0
S 7 1.00
7.811885E+03 5.7075E-04
1.168486E+03 4.3473E-03
2.735860E+02 2.1213E-02
7.954210E+01 7.9825E-02
2.613539E+01 2.2806E-01
9.217097E+00 4.3136E-01
3.390089E+00 3.5137E-01
S 7 1.00
7.811885E+03 1.0361E-04
1.168486E+03 7.7592E-04
2.735860E+02 3.9293E-03
7.954210E+01 1.4569E-02
2.613539E+01 4.6979E-02
9.217097E+00 9.9114E-02
3.390089E+00 1.5321E-01
S 1 1.00
1.045814E+00 0.1000E+01
S 1 1.00
3.150044E-01 0.1000E+01
S 1 1.00
1.155406E-01 0.1000E+01
P 3 1.00
1.844063E+02 2.5812E-04
3.098346E+01 3.5919E-03
7.830746E+00 2.4201E-02
P 1 1.00
2.308071E+00 0.1000E+01
P 1 1.00
7.525987E-01 0.1000E+01
P 1 1.00
2.530275E-01 0.1000E+01
P 1 1.00
8.574535E-02 0.1000E+01
D 1 1.00
1.566238E+00 0.1000E+01
D 1 1.00
3.508371E-01 0.1000E+01
F 1 1.00
1.148503E+00 0.1000E+01
****
```

Equilibrium geometry of 10'-hydroxyusambarensine (**1**)

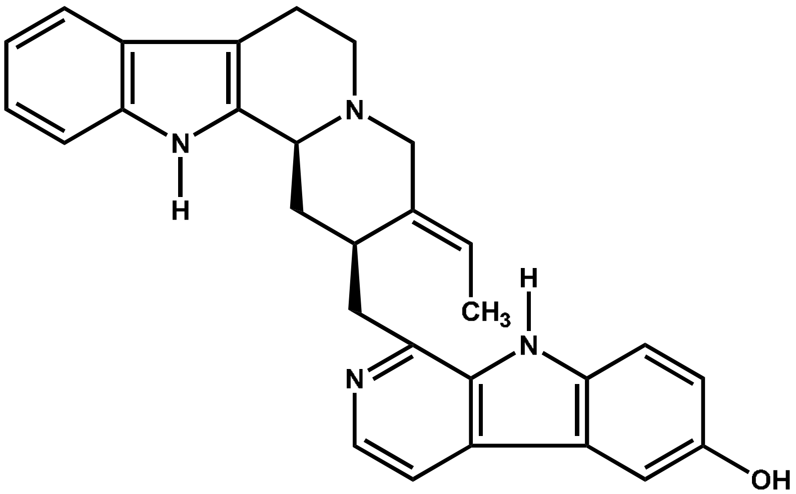


| | | | |
|---|---|---|---|
| O | -6.394331 | 3.027550 | 0.537892 |
| N | 3.301681 | -1.346042 | 0.086243 |
| N | 1.494289 | 1.775900 | -0.740848 |
| N | -2.058264 | -2.998707 | -1.752602 |
| N | -2.427560 | -0.724545 | 1.109050 |
| C | 2.227833 | -0.657599 | -0.630014 |
| C | 0.871484 | -1.119192 | -0.110473 |
| C | 0.708798 | -2.636136 | -0.266311 |
| C | 2.443630 | 0.813684 | -0.486650 |
| C | 3.206613 | -2.788501 | -0.131247 |
| C | 4.617293 | -0.834178 | -0.291364 |
| C | 1.892856 | -3.321182 | 0.379537 |
| C | 3.610171 | 1.397999 | -0.100066 |
| C | 4.822825 | 0.588499 | 0.223173 |
| C | -0.659313 | -3.113844 | 0.225724 |
| C | 3.385501 | 2.814317 | -0.102680 |
| C | 2.046188 | 3.013085 | -0.513413 |
| C | 1.783415 | -4.268274 | 1.308078 |
| C | -1.786383 | -2.498329 | -0.554958 |
| C | 4.177324 | 3.929157 | 0.194415 |
| C | -2.526690 | -1.413465 | -0.076400 |
| C | 1.488309 | 4.283766 | -0.629902 |
| C | 2.878703 | -5.003666 | 2.017410 |
| C | -3.557534 | -0.848762 | -0.850733 |
| C | 3.627028 | 5.190819 | 0.079053 |
| C | 2.294451 | 5.365318 | -0.329122 |
| C | -4.106760 | 0.237831 | -0.077812 |
| C | -3.377722 | 0.276895 | 1.123561 |
| C | -3.819119 | -1.397720 | -2.103348 |
| C | -3.040339 | -2.465816 | -2.499184 |
| C | -5.135337 | 1.155202 | -0.308814 |
| C | -3.658578 | 1.223335 | 2.107007 |
| C | -5.409392 | 2.089335 | 0.668121 |
| C | -4.675609 | 2.121401 | 1.865367 |
| H | 2.278733 | -0.915783 | -1.705107 |

| | | | |
|---|---|---|---|
| H | 0.079218 | -0.608053 | -0.659976 |
| H | 0.784221 | -0.842591 | 0.943658 |
| H | 0.757882 | -2.852632 | -1.340524 |
| H | 3.294726 | -3.018964 | -1.207776 |
| H | 4.048147 | -3.258338 | 0.367420 |
| H | 4.736178 | -0.851792 | -1.386466 |
| H | 5.370766 | -1.497791 | 0.128891 |
| H | 5.001382 | 0.568023 | 1.300490 |
| H | 5.714138 | 1.016168 | -0.238005 |
| H | -0.764930 | -2.894498 | 1.290278 |
| H | -0.727170 | -4.194633 | 0.109504 |
| H | 0.551093 | 1.608350 | -1.044305 |
| H | 0.787190 | -4.571906 | 1.607418 |
| H | 5.204377 | 3.802867 | 0.509866 |
| H | 0.462819 | 4.417758 | -0.944170 |
| H | 3.874897 | -4.674550 | 1.740167 |
| H | 2.772524 | -4.885610 | 3.096135 |
| H | 2.808968 | -6.072813 | 1.813542 |
| H | 4.227366 | 6.060546 | 0.305176 |
| H | 1.891666 | 6.364872 | -0.409689 |
| H | -1.775853 | -0.912896 | 1.849813 |
| H | -4.596315 | -1.012706 | -2.746903 |
| H | -3.203940 | -2.929417 | -3.462797 |
| H | -5.704604 | 1.135177 | -1.229115 |
| H | -3.100089 | 1.255387 | 3.030893 |
| H | -4.928830 | 2.872321 | 2.599773 |
| H | -6.838818 | 2.922974 | -0.307951 |

Equilibrium geometry of 2-2-di(3-indolyl)-3-indolone (**2**)

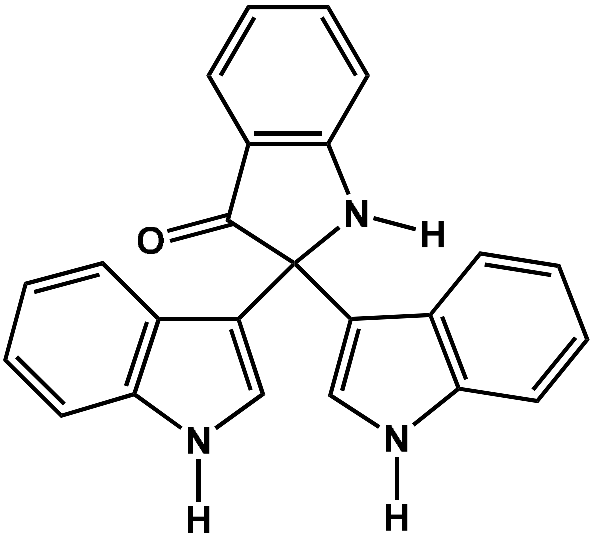


| | | | |
|---|---|---|---|
| C | -1.193205 | -3.837036 | -1.284155 |
| C | -0.576609 | -2.537599 | -1.244483 |
| C | -1.589002 | -1.628975 | -1.130284 |
| C | -2.585644 | -3.631839 | -1.191500 |
| N | -2.795024 | -2.278982 | -1.097471 |

| | | | |
|---|---|---|---|
| C | -3.494560 | -4.688643 | -1.197118 |
| C | -2.983784 | -5.966307 | -1.302311 |
| C | -1.600130 | -6.194393 | -1.392531 |
| C | -0.702827 | -5.146363 | -1.380177 |
| C | 0.900259 | -2.271252 | -1.361728 |
| C | 1.684685 | -2.823542 | -0.210940 |
| C | 3.116328 | -2.815573 | -0.079180 |
| C | 3.413137 | -3.376708 | 1.180834 |
| N | 2.219403 | -3.701344 | 1.776990 |
| C | 1.188488 | -3.362721 | 0.939141 |
| C | 4.168585 | -2.379541 | -0.894066 |
| C | 5.464006 | -2.517508 | -0.439155 |
| C | 5.736906 | -3.086601 | 0.816259 |
| C | 4.720324 | -3.522367 | 1.641584 |
| C | 1.118815 | -0.736906 | -1.489947 |
| C | 1.520299 | -0.483834 | -2.868911 |
| C | 1.615440 | -1.716317 | -3.520016 |
| N | 1.413305 | -2.768697 | -2.657161 |
| C | 1.769820 | 0.714126 | -3.531990 |
| C | 2.096505 | 0.668631 | -4.872113 |
| C | 2.165452 | -0.568677 | -5.530044 |
| C | 1.930949 | -1.765628 | -4.879910 |
| O | 0.949821 | 0.049619 | -0.588446 |
| H | -1.540031 | -0.555648 | -1.056759 |
| H | -3.690505 | -1.830233 | -1.015051 |
| H | -4.558754 | -4.511315 | -1.120227 |
| H | -3.660900 | -6.809533 | -1.309241 |
| H | -1.235717 | -7.209955 | -1.464692 |
| H | 0.363218 | -5.333736 | -1.424606 |
| H | 2.112934 | -4.121115 | 2.683600 |
| H | 0.164125 | -3.533164 | 1.225583 |
| H | 3.970218 | -1.941493 | -1.864142 |
| H | 6.286811 | -2.183872 | -1.056611 |
| H | 6.763669 | -3.182243 | 1.142323 |
| H | 4.928023 | -3.956672 | 2.610372 |
| H | 1.002891 | -3.615748 | -3.021297 |
| H | 1.697137 | 1.651779 | -2.996644 |
| H | 2.294644 | 1.578287 | -5.420194 |
| H | 2.413828 | -0.588523 | -6.583270 |
| H | 1.997671 | -2.709842 | -5.402319 |

Equilibrium geometry of acantholactam (**3**)

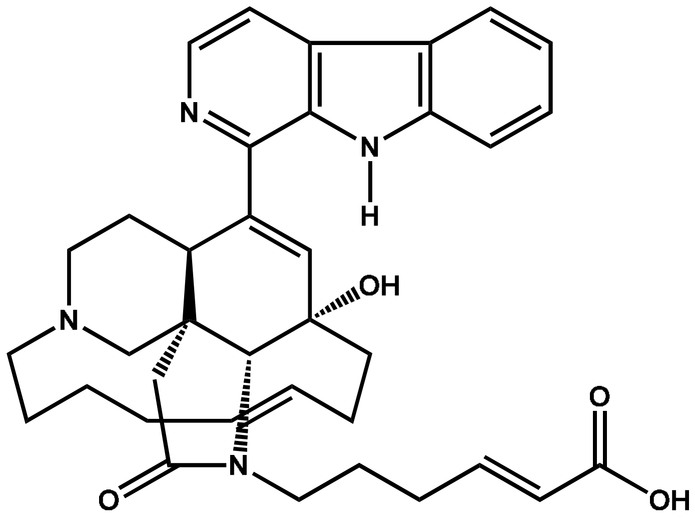


| | | | |
|---|---|---|---|
| O | 0.011522 | -1.314907 | -2.910697 |
| O | 1.917915 | -4.289869 | -2.082897 |
| O | 6.613205 | 4.071861 | -0.234977 |
| O | 7.366689 | 2.255039 | 0.820447 |
| N | 1.823244 | -2.126410 | -1.332294 |
| N | 1.238886 | -1.432266 | 2.332325 |
| N | -4.084552 | -2.274963 | 0.444987 |
| N | -3.913678 | 1.292539 | -0.448048 |
| C | 0.165299 | -2.282027 | 0.285820 |
| C | 0.854346 | -1.272549 | -0.657724 |
| C | -1.192472 | -1.829581 | 0.826271 |
| C | -0.078433 | -0.567875 | -1.692056 |
| C | 1.101968 | -2.582006 | 1.467418 |
| C | 0.116903 | -3.524169 | -0.609334 |
| C | -1.089556 | -0.837020 | 2.005735 |
| C | -2.048429 | -1.171843 | -0.220926 |
| C | 1.381359 | -3.399615 | -1.441476 |
| C | -1.528683 | -0.564533 | -1.284024 |
| C | -0.017941 | -1.195801 | 3.018841 |
| C | 0.418672 | 0.861327 | -1.963788 |
| C | 2.960710 | -1.617194 | -2.067401 |
| C | 2.383963 | -1.478129 | 3.227208 |
| C | -3.509412 | -1.148260 | 0.038687 |
| C | 0.368781 | 1.800748 | -0.739554 |
| C | 3.853550 | -0.759829 | -1.183548 |
| C | 2.868837 | -0.087975 | 3.634750 |
| C | -4.278843 | 0.011435 | -0.104986 |
| C | 3.215984 | 0.794756 | 2.430699 |
| C | 1.692242 | 2.447382 | -0.447344 |
| C | 5.021474 | -0.146294 | -1.959275 |
| C | -5.662216 | -0.014645 | 0.155716 |
| C | 2.050506 | 1.665897 | 1.948735 |
| C | 2.389796 | 2.396873 | 0.684725 |
| C | -5.403324 | -2.308482 | 0.680157 |
| C | -6.145449 | 1.330166 | -0.046182 |
| C | -6.240088 | -1.216509 | 0.548987 |
| C | -5.025111 | 2.104230 | -0.408647 |
| C | 5.855169 | 0.681473 | -1.042117 |

| | | | |
|---|---|---|---|
| C | -7.400730 | 1.930114 | 0.055235 |
| C | -5.133630 | 3.468767 | -0.669944 |
| C | 5.913917 | 2.008376 | -1.065447 |
| C | -7.513256 | 3.281566 | -0.203635 |
| C | -6.386935 | 4.038577 | -0.561500 |
| C | 6.706840 | 2.744382 | -0.063366 |
| H | 1.364452 | -0.514012 | -0.064163 |
| H | -1.714000 | -2.720573 | 1.184132 |
| H | 0.698975 | -3.453449 | 2.011919 |
| H | 2.085052 | -2.860116 | 1.089072 |
| H | -0.739362 | -3.502754 | -1.286100 |
| H | 0.102669 | -4.463678 | -0.060533 |
| H | -2.062803 | -0.769564 | 2.493039 |
| H | -0.868286 | 0.155716 | 1.612852 |
| H | -2.186591 | -0.073443 | -1.997256 |
| H | 0.100614 | -0.373307 | 3.723445 |
| H | -0.312971 | -2.086100 | 3.598646 |
| H | -0.177174 | 1.269456 | -2.781879 |
| H | 1.442193 | 0.784432 | -2.334998 |
| H | 2.617144 | -1.046645 | -2.934022 |
| H | 3.506524 | -2.481466 | -2.440879 |
| H | 3.197438 | -1.974102 | 2.695470 |
| H | 2.170064 | -2.083183 | 4.121199 |
| H | -0.615606 | -0.942491 | -3.538154 |
| H | 0.014869 | 1.257571 | 0.134028 |
| H | -0.369002 | 2.584030 | -0.921593 |
| H | 3.275252 | 0.057273 | -0.749536 |
| H | 4.226796 | -1.364371 | -0.353743 |
| H | 3.746272 | -0.224526 | 4.267395 |
| H | 2.123188 | 0.412623 | 4.256600 |
| H | 3.547923 | 0.156478 | 1.607165 |
| H | 4.057189 | 1.447218 | 2.672333 |
| H | 2.114719 | 3.011100 | -1.274067 |
| H | 5.629870 | -0.941826 | -2.394025 |
| H | 4.638338 | 0.467093 | -2.775588 |
| H | 1.816668 | 2.390244 | 2.734881 |
| H | 1.168942 | 1.042605 | 1.820122 |
| H | -2.972774 | 1.597685 | -0.627575 |
| H | 3.338284 | 2.927638 | 0.705753 |
| H | -5.802192 | -3.263121 | 0.995865 |
| H | -7.295588 | -1.302123 | 0.760769 |
| H | 6.409443 | 0.158090 | -0.269239 |
| H | -8.267458 | 1.346163 | 0.331884 |
| H | -4.270306 | 4.056051 | -0.946623 |
| H | 5.378571 | 2.594991 | -1.799223 |
| H | -8.475745 | 3.765975 | -0.131074 |
| H | -6.503052 | 5.094898 | -0.757898 |
| H | 7.144651 | 4.503842 | 0.447538 |

Equilibrium geometry of alasmontamine A (**4**)

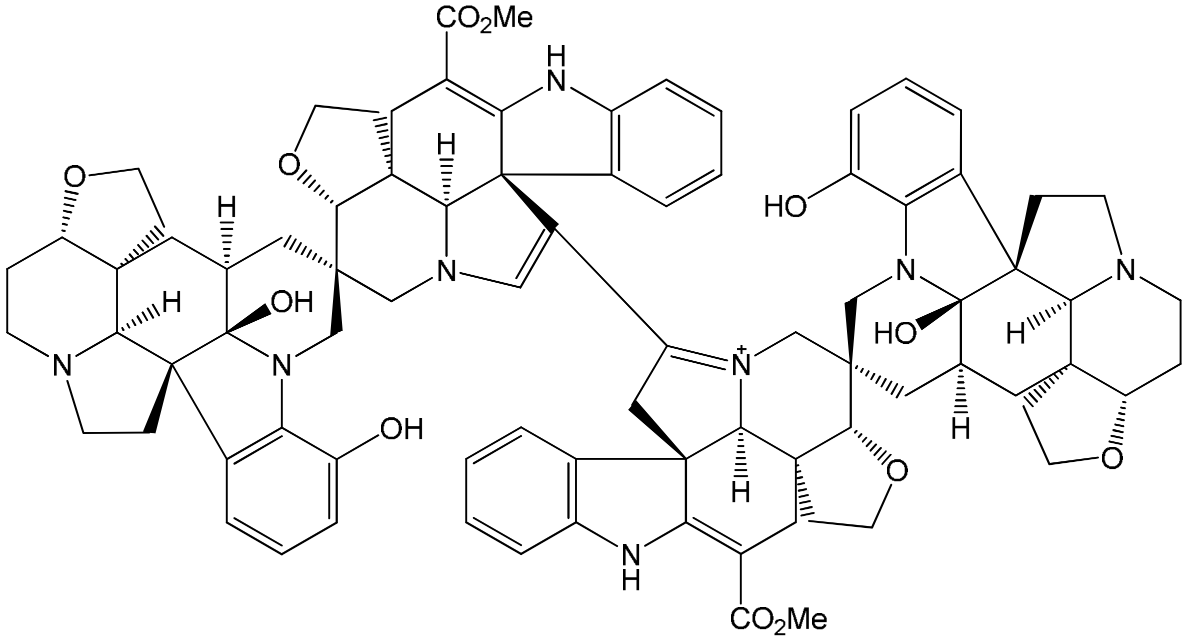


| | | | |
|---|---|---|---|
| N | -6.469236 | -0.974674 | 2.483841 |
| C | -5.993793 | -0.541149 | 1.279537 |
| C | -1.767896 | 1.264250 | -0.604795 |
| N | -2.648759 | 0.327001 | 0.094932 |
| C | -2.574431 | -0.993994 | -0.000847 |
| C | -3.817697 | -1.614627 | 0.569250 |
| C | -4.478135 | -0.462855 | 1.374619 |
| C | -4.239007 | -0.636317 | 2.856495 |
| C | -3.077322 | -0.572441 | 3.610159 |
| C | -3.155101 | -0.844527 | 4.982488 |
| C | -4.377154 | -1.183550 | 5.566640 |
| C | -5.552922 | -1.260039 | 4.811748 |
| C | -5.456906 | -0.979425 | 3.454591 |
| C | -2.510989 | 2.534253 | -1.072824 |
| C | -3.950993 | 2.125250 | -1.450755 |
| C | -6.620978 | -0.183074 | 0.126588 |
| C | -5.755026 | 0.440004 | -0.938723 |
| C | -5.810148 | 3.512329 | -1.038860 |
| C | -5.577631 | 2.682279 | 0.217835 |
| C | -4.787689 | 1.479767 | -0.323530 |
| C | -3.884009 | 0.816329 | 0.735610 |
| C | -8.065118 | -0.238254 | -0.070252 |
| C | -10.148213 | -0.858520 | 0.838669 |
| N | -0.585709 | 3.708290 | -2.251081 |
| C | -0.606098 | 4.825985 | -1.298859 |
| C | 0.902731 | 8.581462 | 0.343870 |
| N | 0.586288 | 7.472008 | -0.546542 |
| C | 1.159255 | 7.633013 | -1.885924 |
| C | 1.214904 | 6.205985 | -2.456628 |
| C | 0.871916 | 5.277372 | -1.255018 |
| C | 1.562594 | 3.941604 | -1.427529 |

| | | | |
|---|---|---|---|
| C | 2.885049 | 3.584149 | -1.212455 |
| C | 3.317246 | 2.322201 | -1.632380 |
| C | 2.432165 | 1.459776 | -2.275748 |
| C | 1.100062 | 1.822550 | -2.507449 |
| C | 0.654388 | 3.073788 | -2.067658 |
| C | 0.270747 | 8.350272 | 1.708270 |
| C | 0.640490 | 6.992195 | 2.279427 |
| C | -1.062003 | 4.288936 | 0.072670 |
| C | -0.949626 | 5.374390 | 1.133153 |
| C | 2.500999 | 5.636432 | 2.640024 |
| C | 1.395179 | 4.780600 | 2.001959 |
| C | 0.505205 | 5.816204 | 1.297996 |
| C | 1.137148 | 6.201877 | -0.051819 |
| C | -2.456684 | 3.672337 | -0.033681 |
| C | -1.869186 | 3.037665 | -2.390562 |
| N | -3.678023 | -4.307303 | -1.052576 |
| C | -2.422541 | -4.175071 | -0.526811 |
| C | 2.004207 | -2.242752 | -1.384805 |
| N | 0.557777 | -2.255524 | -1.256573 |
| C | -0.199619 | -1.330688 | -0.699243 |
| C | -1.526445 | -1.727650 | -0.572032 |
| C | -1.693215 | -3.074686 | -1.305375 |
| C | -2.637998 | -2.852000 | -2.473532 |
| C | -2.541846 | -2.003135 | -3.566257 |
| C | -3.635661 | -1.901079 | -4.434624 |
| C | -4.805243 | -2.623975 | -4.184275 |
| C | -4.920002 | -3.465618 | -3.073152 |
| C | -3.816263 | -3.566494 | -2.232893 |
| C | 2.643174 | -3.420529 | -0.624732 |
| C | 1.856796 | -4.704485 | -0.929868 |
| C | -1.787453 | -4.884753 | 0.442804 |
| C | -0.297271 | -4.668833 | 0.556771 |
| C | 1.150958 | -6.090808 | -2.634559 |
| C | -0.017648 | -5.952633 | -1.643056 |
| C | 0.313270 | -4.669664 | -0.856135 |
| C | -0.207108 | -3.450743 | -1.638565 |
| C | -2.413096 | -5.944022 | 1.228849 |
| C | -4.400829 | -7.097082 | 1.746049 |
| N | 3.630242 | -2.254913 | 1.409745 |
| C | 5.002806 | -2.488867 | 0.944275 |
| C | 9.349836 | -2.103978 | 1.194086 |
| N | 7.930568 | -2.178373 | 1.516332 |
| C | 7.658204 | -1.982483 | 2.943208 |
| C | 6.191685 | -1.524367 | 2.998491 |
| C | 5.786112 | -1.283099 | 1.514196 |
| C | 4.717452 | -0.214260 | 1.462546 |
| C | 4.818781 | 1.162265 | 1.589997 |
| C | 3.651571 | 1.918855 | 1.743186 |
| C | 2.415380 | 1.280122 | 1.817493 |
| C | 2.310300 | -0.109133 | 1.717481 |
| C | 3.466650 | -0.866509 | 1.493606 |

| | | | |
|---|---|---|---|
| C | 9.559219 | -2.322937 | -0.297551 |
| C | 8.690857 | -1.400372 | -1.134597 |
| C | 4.999326 | -2.479116 | -0.596298 |
| C | 6.418564 | -2.537106 | -1.140570 |
| C | 8.098885 | 0.837564 | -1.351248 |
| C | 6.812065 | -0.004620 | -1.390097 |
| C | 7.219826 | -1.313921 | -0.693991 |
| C | 7.158048 | -1.130119 | 0.832069 |
| C | 4.095722 | -3.597958 | -1.102694 |
| C | 2.627814 | -3.186887 | 0.906116 |
| H | -7.459165 | -1.053624 | 2.675490 |
| H | -0.914149 | 1.520225 | 0.042139 |
| H | -1.380028 | 0.726734 | -1.483079 |
| H | -3.588129 | -2.481442 | 1.200993 |
| H | -4.464119 | -1.957383 | -0.252140 |
| H | -2.121504 | -0.312537 | 3.148143 |
| H | -2.256319 | -0.793909 | 5.596605 |
| H | -4.421034 | -1.393494 | 6.635907 |
| H | -6.505914 | -1.523123 | 5.270771 |
| H | -3.871031 | 1.415720 | -2.294781 |
| H | -6.396218 | 0.933730 | -1.680972 |
| H | -5.170155 | -0.311089 | -1.499228 |
| H | -5.849540 | 4.591027 | -0.837044 |
| H | -6.737220 | 3.216656 | -1.555100 |
| H | -4.981559 | 3.243443 | 0.949694 |
| H | -6.517675 | 2.384220 | 0.700389 |
| H | -3.616912 | 1.535608 | 1.520064 |
| H | -10.512564 | -1.314759 | 1.763735 |
| H | -10.412108 | -1.479456 | -0.025848 |
| H | -10.573067 | 0.144502 | 0.711691 |
| H | 2.003616 | 8.683597 | 0.443820 |
| H | 0.519688 | 9.511658 | -0.099943 |
| H | 2.179820 | 8.055563 | -1.813601 |
| H | 0.545875 | 8.322685 | -2.482638 |
| H | 2.223428 | 5.973238 | -2.818939 |
| H | 0.507950 | 6.047062 | -3.279198 |
| H | 3.576905 | 4.277712 | -0.730092 |
| H | 4.347000 | 2.008072 | -1.464048 |
| H | 2.780381 | 0.490292 | -2.640181 |
| H | 0.580536 | 9.136425 | 2.411404 |
| H | -0.824712 | 8.401968 | 1.618128 |
| H | 0.012643 | 6.778631 | 3.165221 |
| H | -0.345616 | 3.495977 | 0.348729 |
| H | -1.325969 | 5.000534 | 2.098286 |
| H | -1.581349 | 6.233653 | 0.854080 |
| H | 3.430658 | 5.624345 | 2.050237 |
| H | 2.733953 | 5.307921 | 3.663524 |
| H | 1.800605 | 4.033259 | 1.306897 |
| H | 0.812089 | 4.257269 | 2.774785 |
| H | 2.237138 | 6.304283 | 0.083170 |
| H | -2.750022 | 3.315951 | 0.965077 |

| | | | |
|---|---|---|---|
| H | -3.172376 | 4.454712 | -0.329749 |
| H | -2.558137 | 3.778031 | -2.817285 |
| H | -1.771022 | 2.212137 | -3.104195 |
| H | -4.326221 | -5.013225 | -0.729122 |
| H | 2.256601 | -2.317803 | -2.454753 |
| H | 2.365094 | -1.277489 | -1.003449 |
| H | 0.258305 | -0.408786 | -0.343300 |
| H | -1.633108 | -1.423106 | -3.743969 |
| H | -3.577671 | -1.249591 | -5.306251 |
| H | -5.651429 | -2.529337 | -4.865692 |
| H | -5.836199 | -4.021253 | -2.873879 |
| H | 2.214984 | -5.498538 | -0.245498 |
| H | 0.141308 | -5.473514 | 1.161396 |
| H | -0.086128 | -3.718387 | 1.070294 |
| H | 0.856627 | -5.905686 | -3.675986 |
| H | 1.614914 | -7.086126 | -2.569828 |
| H | -0.988239 | -5.895018 | -2.153420 |
| H | -0.055603 | -6.804926 | -0.950045 |
| H | -0.086529 | -3.607751 | -2.720380 |
| H | -4.334117 | -6.874228 | 2.817968 |
| H | -3.947672 | -8.075671 | 1.547137 |
| H | -5.443221 | -7.081077 | 1.415160 |
| H | 9.754119 | -1.115575 | 1.497730 |
| H | 9.883907 | -2.874050 | 1.769300 |
| H | 8.319525 | -1.194737 | 3.352541 |
| H | 7.851644 | -2.911353 | 3.498375 |
| H | 6.103402 | -0.582328 | 3.552789 |
| H | 5.527660 | -2.261485 | 3.464781 |
| H | 5.797273 | 1.647622 | 1.579782 |
| H | 3.711060 | 3.004254 | 1.825416 |
| H | 1.494595 | 1.842595 | 1.982050 |
| H | 10.614762 | -2.165574 | -0.561354 |
| H | 9.307763 | -3.363077 | -0.554429 |
| H | 8.717529 | -1.725559 | -2.192151 |
| H | 4.573136 | -1.507086 | -0.902319 |
| H | 6.397886 | -2.576973 | -2.240871 |
| H | 6.909982 | -3.462157 | -0.796129 |
| H | 8.292818 | 1.327579 | -2.316676 |
| H | 8.070062 | 1.609447 | -0.567807 |
| H | 6.514744 | -0.216994 | -2.427706 |
| H | 5.976824 | 0.502186 | -0.889076 |
| H | 7.575586 | -0.131692 | 1.091317 |
| H | 4.117069 | -3.633565 | -2.200747 |
| H | 4.477252 | -4.564587 | -0.737872 |
| H | 1.648108 | -2.829358 | 1.229826 |
| H | 2.811839 | -4.153788 | 1.397870 |
| H | -1.215286 | 6.645663 | -1.339545 |
| H | 0.720655 | 0.274047 | -3.580442 |
| H | 6.418206 | -3.706002 | 1.394286 |
| H | 1.023901 | -1.209556 | 2.655127 |
| O | -3.737376 | -6.077741 | 0.987656 |

| | | | |
|---|---|---|---|
| O | 5.445555 | -3.733053 | 1.439041 |
| O | 9.172803 | -0.060152 | -1.039655 |
| O | 1.054558 | -0.690449 | 1.839878 |
| O | -8.650081 | 0.156418 | -1.064625 |
| O | -4.692911 | 3.254161 | -1.898791 |
| O | -1.491034 | 5.816241 | -1.769934 |
| O | 2.016509 | 6.986108 | 2.663244 |
| O | -8.724308 | -0.781886 | 0.978978 |
| O | 0.224959 | 0.995703 | -3.173329 |
| O | 2.109813 | -5.088918 | -2.276277 |
| O | -1.830740 | -6.649323 | 2.034400 |

Equilibrium geometry of anabsinthin (**5**)

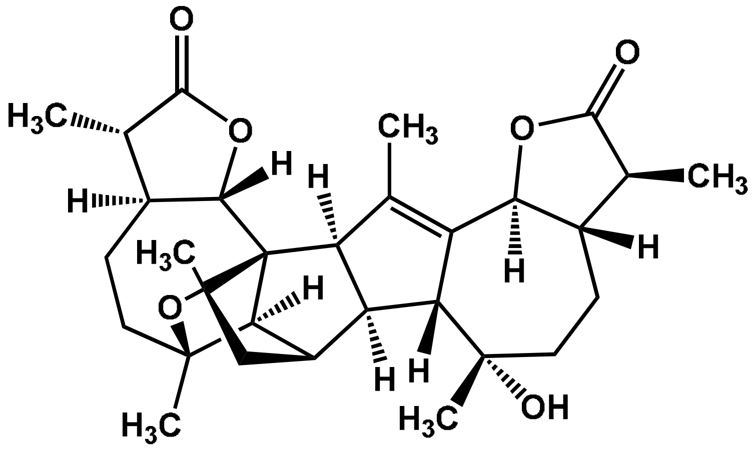


| | | | |
|---|---|---|---|
| O | -2.352482 | 1.078412 | -2.272179 |
| O | -2.552358 | -1.936139 | 0.706542 |
| O | 2.213547 | 0.406871 | 3.076285 |
| O | 3.089752 | -1.673862 | -1.182207 |
| O | -3.734299 | -3.335790 | 1.966951 |
| O | 4.598536 | -2.247297 | -2.714927 |
| C | -1.648074 | 0.145249 | -0.264228 |
| C | -1.979303 | 1.652662 | -0.054392 |
| C | -0.505952 | 2.110477 | -0.165333 |
| C | -1.182604 | 0.431992 | -1.726658 |
| C | -0.515706 | -0.100270 | 0.765705 |
| C | 0.147845 | 1.285635 | 0.949266 |
| C | -2.822210 | 2.028670 | -1.285452 |
| C | -0.108125 | 1.514567 | -1.533576 |
| C | -2.811690 | -0.846564 | -0.217402 |
| C | 1.677773 | 1.090254 | 0.847225 |
| C | 0.624029 | -1.010605 | 0.404030 |
| C | -4.174455 | -0.318123 | 0.219375 |
| C | -4.335191 | 1.866783 | -1.082082 |
| C | 1.791746 | -0.364376 | 0.420992 |
| C | -0.778154 | -0.685694 | -2.651669 |
| C | -4.875177 | 0.456707 | -0.879750 |

| | | | |
|---|---|---|---|
| C | 2.466439 | 1.452444 | 2.135438 |
| C | -2.594620 | 3.460431 | -1.767657 |
| C | -4.866435 | -1.608095 | 0.653437 |
| C | 3.101059 | -1.020747 | 0.122730 |
| C | 0.410926 | -2.469978 | 0.167909 |
| C | 3.980913 | 1.599605 | 1.896545 |
| C | -3.706331 | -2.409053 | 1.201824 |
| C | 4.315528 | -0.111264 | 0.049653 |
| C | 4.764631 | 0.386110 | 1.410305 |
| C | 1.987264 | 2.793265 | 2.699573 |
| C | -6.017774 | -1.469936 | 1.630079 |
| C | 5.287001 | -1.000410 | -0.722384 |
| C | 4.342456 | -1.715692 | -1.668055 |
| C | 6.432402 | -0.308584 | -1.434437 |
| H | -2.439307 | 1.913726 | 0.897284 |
| H | -0.336514 | 3.181123 | -0.069967 |
| H | -0.979876 | -0.453956 | 1.688392 |
| H | -0.139643 | 1.704111 | 1.908691 |
| H | 0.892665 | 1.089163 | -1.537009 |
| H | -0.166793 | 2.224289 | -2.355837 |
| H | -2.933946 | -1.308618 | -1.199995 |
| H | 2.078642 | 1.757070 | 0.075167 |
| H | -4.046914 | 0.310573 | 1.107710 |
| H | -4.836896 | 2.303808 | -1.947947 |
| H | -4.607822 | 2.485109 | -0.222645 |
| H | 0.143076 | -1.148641 | -2.300930 |
| H | -1.549496 | -1.449293 | -2.745722 |
| H | -0.593151 | -0.271278 | -3.643652 |
| H | -5.939230 | 0.532040 | -0.647304 |
| H | -4.795365 | -0.111359 | -1.809917 |
| H | -3.123368 | 3.613231 | -2.708404 |
| H | -2.996074 | 4.162303 | -1.034956 |
| H | -1.546213 | 3.694837 | -1.924022 |
| H | -5.197636 | -2.145539 | -0.244232 |
| H | 3.295494 | -1.811151 | 0.856833 |
| H | -0.311880 | -2.639861 | -0.632188 |
| H | 1.333590 | -2.986746 | -0.083482 |
| H | -0.020208 | -2.919397 | 1.064659 |
| H | 4.417919 | 1.927101 | 2.843326 |
| H | 4.126669 | 2.423434 | 1.191964 |
| H | 4.070164 | 0.743222 | -0.592091 |
| H | 4.693524 | -0.427740 | 2.136798 |
| H | 5.817817 | 0.669477 | 1.359789 |
| H | 0.996401 | 2.711707 | 3.137900 |
| H | 1.961862 | 3.552985 | 1.916713 |
| H | 2.669510 | 3.135428 | 3.478949 |
| H | -5.695519 | -0.943348 | 2.528787 |
| H | -6.394708 | -2.448363 | 1.922334 |
| H | -6.834205 | -0.909343 | 1.176777 |
| H | 2.598598 | 0.659290 | 3.922050 |
| H | 5.674336 | -1.769725 | -0.042673 |

| | | | |
|---|---|---|---|
| H | 6.053601 | 0.440444 | -2.130228 |
| H | 7.026837 | -1.027368 | -1.995404 |
| H | 7.083210 | 0.186916 | -0.715295 |

Equilibrium geometry of asperlicin (**6**)

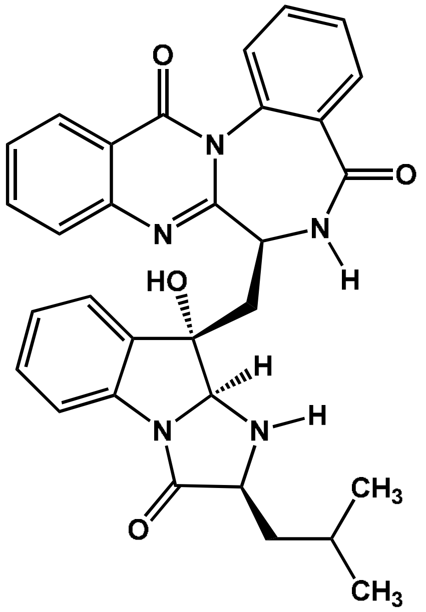


| | | | |
|---|---|---|---|
| C | 6.394614 | 0.062583 | -1.378436 |
| C | 6.598687 | -0.596205 | -0.159305 |
| C | 5.523966 | -1.107573 | 0.533308 |
| C | 4.235090 | -0.955293 | 0.015636 |
| C | 4.029038 | -0.311228 | -1.203357 |
| C | 5.128361 | 0.200240 | -1.903172 |
| C | 3.079455 | -1.431078 | 0.768222 |
| N | 1.821263 | -1.029988 | 0.236496 |
| C | 1.745759 | -0.580628 | -1.070988 |
| N | 2.768719 | -0.233234 | -1.762529 |
| C | 0.722837 | -1.006347 | 1.166489 |
| C | -0.525470 | -1.548277 | 0.890987 |
| C | -0.896834 | -2.188862 | -0.396305 |
| N | -0.573234 | -1.554431 | -1.555653 |
| C | 0.455555 | -0.575103 | -1.892902 |
| C | 0.971341 | -0.473042 | 2.426775 |
| C | -0.009792 | -0.477592 | 3.398095 |
| C | -1.259612 | -1.016716 | 3.128777 |
| C | -1.507108 | -1.554635 | 1.882375 |
| O | -1.561352 | -3.210482 | -0.416592 |
| O | 3.148547 | -2.073908 | 1.790402 |
| C | -0.002458 | 0.822222 | -2.357610 |
| C | -0.515169 | 1.966764 | -1.466429 |
| C | 0.071664 | 2.115553 | -0.071733 |
| C | -0.938178 | 2.151125 | 0.892219 |
| N | -2.168014 | 1.913104 | 0.275541 |
| C | -2.038523 | 1.986183 | -1.186114 |

| | | | |
|---|---|---|---|
| C | 1.381961 | 2.364330 | 0.291904 |
| C | 1.662641 | 2.679155 | 1.619608 |
| C | 0.640963 | 2.735403 | 2.560449 |
| C | -0.678388 | 2.465352 | 2.213993 |
| N | -2.929464 | 0.966974 | -1.697110 |
| C | -4.040416 | 1.035443 | -0.721950 |
| C | -3.331560 | 1.303863 | 0.627415 |
| O | -3.724939 | 1.041871 | 1.743504 |
| C | -4.987319 | -0.147512 | -0.687063 |
| C | -4.285230 | -1.509770 | -0.678291 |
| C | -5.032641 | -2.517681 | 0.186252 |
| C | -4.104768 | -2.058144 | -2.092684 |
| O | -0.303328 | 3.170156 | -2.211288 |
| H | 7.243365 | 0.459785 | -1.918573 |
| H | 7.599165 | -0.702300 | 0.235398 |
| H | 5.649098 | -1.617036 | 1.478512 |
| H | 4.958607 | 0.686308 | -2.853841 |
| H | -0.886500 | -2.090232 | -2.354760 |
| H | 0.852753 | -0.967941 | -2.827571 |
| H | 1.943238 | -0.059651 | 2.646501 |
| H | 0.201850 | -0.045410 | 4.366572 |
| H | -2.034322 | -1.016818 | 3.881937 |
| H | -2.463772 | -2.004161 | 1.656194 |
| H | 0.881380 | 1.217673 | -2.860054 |
| H | -0.746366 | 0.670642 | -3.142602 |
| H | -2.405759 | 2.958055 | -1.522739 |
| H | 2.178738 | 2.342858 | -0.443676 |
| H | 2.680027 | 2.892899 | 1.916804 |
| H | 0.875006 | 2.989387 | 3.585713 |
| H | -1.477581 | 2.505329 | 2.939817 |
| H | -2.474841 | 0.060786 | -1.594473 |
| H | -4.606609 | 1.946652 | -0.945491 |
| H | -5.584477 | -0.027375 | 0.219113 |
| H | -5.673604 | -0.089416 | -1.534085 |
| H | -3.297694 | -1.373770 | -0.230189 |
| H | -4.513751 | -3.477077 | 0.192020 |
| H | -5.118999 | -2.163193 | 1.214564 |
| H | -6.041509 | -2.676942 | -0.201968 |
| H | -3.508239 | -2.969756 | -2.075809 |
| H | -5.082230 | -2.293270 | -2.520977 |
| H | -3.627189 | -1.337682 | -2.758756 |
| H | 0.643109 | 3.328399 | -2.289995 |

Equilibrium geometry of chetomin (**7**)

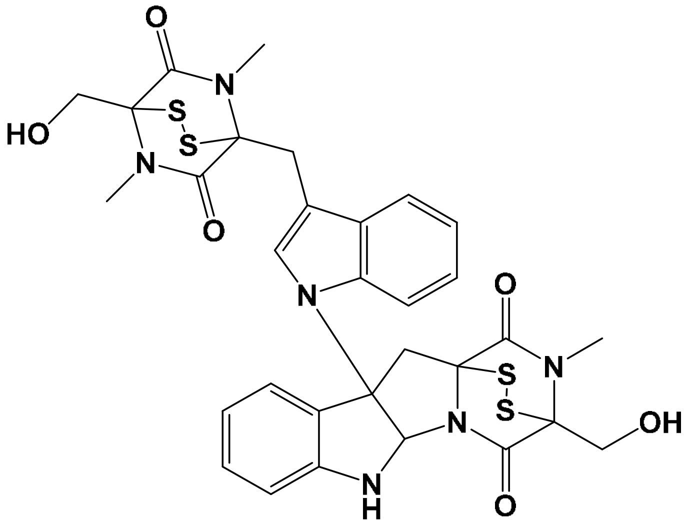


| | | | |
|---|---|---|---|
| S | 5.174918 | 0.690405 | -0.420283 |
| S | 6.000094 | -1.038296 | 0.412444 |
| S | -2.867353 | -1.100449 | 0.232448 |
| S | -4.285768 | -2.621712 | 0.069442 |
| O | 3.026147 | -2.813002 | 1.244671 |
| O | 3.729484 | -0.555354 | -3.267270 |
| O | 5.839832 | -3.675966 | 1.262674 |
| O | -4.399662 | 1.487021 | -1.535818 |
| O | -6.915653 | -1.314590 | 1.854258 |
| O | -7.082400 | -3.503944 | 0.205577 |
| N | 2.945435 | -0.860616 | 0.098320 |
| N | 0.287532 | 1.513081 | -0.161448 |
| N | 1.902710 | -0.065754 | 2.147177 |
| N | 4.570871 | -2.055087 | -1.755640 |
| N | -5.085481 | 0.002519 | 1.506467 |
| N | -5.518517 | -0.468029 | -1.199920 |
| C | 1.672586 | 1.123773 | 0.082144 |
| C | 1.753873 | -0.296626 | 0.730669 |
| C | 3.530358 | -0.058705 | -0.926578 |
| C | 2.499756 | 1.020994 | -1.211925 |
| C | 2.317521 | 1.968256 | 1.164242 |
| C | 4.798247 | -2.285489 | -0.349190 |
| C | 3.497472 | -2.049311 | 0.429321 |
| C | 3.928837 | -0.904857 | -2.123644 |
| C | 2.454323 | 1.209151 | 2.322631 |
| C | -0.182674 | 2.496582 | -1.015852 |
| C | -0.752783 | 1.131153 | 0.657022 |
| C | 2.726074 | 3.285855 | 1.122116 |
| C | 5.371531 | -3.667546 | -0.064694 |
| C | -1.909107 | 1.758929 | 0.302623 |
| C | -1.565094 | 2.655770 | -0.764482 |
| C | 3.032396 | 1.749265 | 3.460801 |
| C | 5.278728 | -2.816355 | -2.780554 |
| C | -3.226783 | 1.559702 | 0.989183 |

| | | | |
|---|---|---|---|
| C | 3.301342 | 3.842519 | 2.260572 |
| C | 0.473839 | 3.280177 | -1.969578 |
| C | -4.057031 | 0.342819 | 0.551155 |
| C | 3.455410 | 3.073053 | 3.408877 |
| C | -2.293384 | 3.606953 | -1.487543 |
| C | -5.775030 | -1.504390 | -0.248998 |
| C | -0.270457 | 4.212090 | -2.663526 |
| C | -4.677011 | 0.541585 | -0.831617 |
| C | -1.643112 | 4.377317 | -2.426463 |
| C | -6.007679 | -0.921784 | 1.138621 |
| C | -4.987329 | 0.371509 | 2.916107 |
| C | -6.940564 | -2.408942 | -0.657226 |
| C | -5.865124 | -0.632364 | -2.607549 |
| H | 0.899393 | -0.935056 | 0.513369 |
| H | 1.876367 | 0.701563 | -2.046939 |
| H | 2.980233 | 1.957937 | -1.478002 |
| H | 2.278513 | -0.838448 | 2.678979 |
| H | -0.584291 | 0.430292 | 1.459328 |
| H | 2.609503 | 3.873313 | 0.218982 |
| H | 6.187414 | -3.868007 | -0.758361 |
| H | 4.573531 | -4.399251 | -0.219819 |
| H | 3.152169 | 1.159909 | 4.359532 |
| H | 6.355863 | -2.671782 | -2.689200 |
| H | 4.948239 | -2.440206 | -3.742132 |
| H | 5.038618 | -3.874297 | -2.709472 |
| H | -3.859859 | 2.438905 | 0.855902 |
| H | -3.029147 | 1.453833 | 2.052184 |
| H | 3.635509 | 4.870093 | 2.250513 |
| H | 1.528383 | 3.186976 | -2.171375 |
| H | 3.913645 | 3.512058 | 4.284934 |
| H | -3.352557 | 3.722988 | -1.310198 |
| H | 6.162312 | -4.555955 | 1.473752 |
| H | 0.220720 | 4.829118 | -3.403496 |
| H | -2.193216 | 5.119015 | -2.989116 |
| H | -4.060002 | -0.013769 | 3.340632 |
| H | -5.829270 | -0.082396 | 3.425736 |
| H | -5.030758 | 1.450239 | 3.039187 |
| H | -6.758015 | -2.809079 | -1.651344 |
| H | -7.842977 | -1.789753 | -0.684654 |
| H | -5.383988 | -1.524846 | -3.009631 |
| H | -5.499357 | 0.241703 | -3.134299 |
| H | -6.942406 | -0.704240 | -2.737800 |
| H | -7.279464 | -3.142613 | 1.080596 |

Equilibrium geometry of diosgenin-3-*O*-*β*-D-glucopyranoside (**8**)

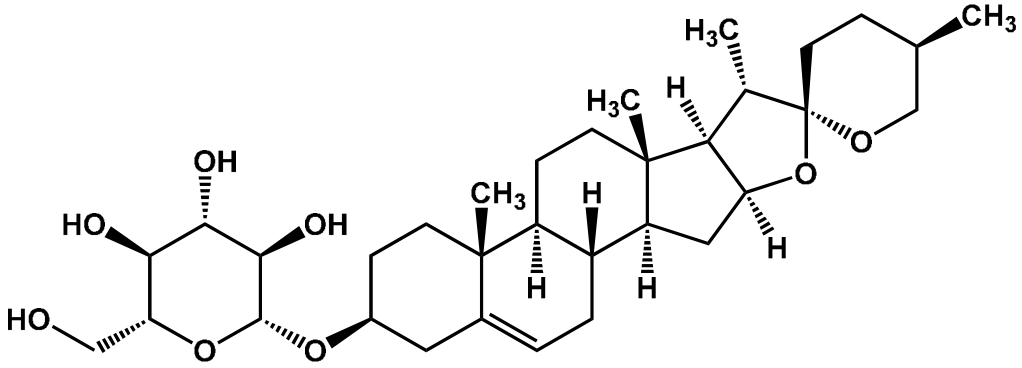

| | | | |
|---|---|---|---|
| O | 5.946016 | 0.808737 | -0.381183 |
| O | 7.256757 | 0.555770 | 1.515506 |
| O | -5.295839 | -0.659263 | -1.530560 |
| O | -6.236308 | 1.100233 | -0.445365 |
| O | -6.732577 | -2.460162 | 0.224094 |
| O | -7.843321 | -1.211757 | 2.529288 |
| O | -8.805617 | 1.386401 | 2.146078 |
| O | -7.156870 | 3.746367 | -0.943125 |
| C | 3.247813 | -1.014505 | 0.118926 |
| C | 2.463994 | 0.297676 | -0.062407 |
| C | 4.462695 | -0.494106 | 0.921765 |
| C | 1.165204 | 0.162128 | -0.842041 |
| C | 4.723215 | 0.924008 | 0.346538 |
| C | 0.275671 | -0.905893 | -0.178579 |
| C | 3.527266 | 1.289491 | -0.539288 |
| C | 2.348248 | -1.993123 | 0.867537 |
| C | 5.819276 | -1.206547 | 0.799226 |
| C | 1.036112 | -2.210962 | 0.108188 |
| C | -1.042782 | -1.140530 | -0.963708 |
| C | 6.768540 | -0.081140 | 0.348043 |
| C | 3.680431 | -1.608008 | -1.228768 |
| C | 0.424849 | 1.494986 | -0.889858 |
| C | -1.654182 | 0.188445 | -1.390078 |
| C | -2.052287 | -1.850008 | -0.031844 |
| C | 6.259522 | -1.947343 | 2.051765 |
| C | 7.913249 | -0.527269 | -0.544763 |
| C | -0.999402 | 1.344750 | -1.324031 |
| C | -0.804145 | -2.009484 | -2.212020 |
| C | -3.077436 | 0.146533 | -1.890883 |
| C | -3.463184 | -1.953829 | -0.602306 |
| C | -4.002245 | -0.571407 | -0.918963 |
| C | 8.865979 | 0.623522 | -0.846948 |
| C | 9.324959 | 1.290032 | 0.446241 |
| C | 8.097722 | 1.674039 | 1.257166 |
| C | 10.195622 | 2.509791 | 0.184396 |
| C | -6.379163 | -0.269340 | -0.767152 |
| C | -6.572470 | -1.089661 | 0.515231 |
| C | -7.749492 | -0.531589 | 1.297482 |

| | | | |
|---|---|---|---|
| C | -7.378516 | 1.663443 | 0.175761 |
| C | -7.606387 | 0.966050 | 1.516224 |
| C | -7.134003 | 3.143094 | 0.337591 |
| H | 2.175613 | 0.610191 | 0.952223 |
| H | 4.172629 | -0.420216 | 1.972264 |
| H | 1.384149 | -0.137099 | -1.871966 |
| H | 4.859028 | 1.654775 | 1.146428 |
| H | -0.015763 | -0.482100 | 0.792975 |
| H | 3.774040 | 1.122240 | -1.589341 |
| H | 3.226575 | 2.329511 | -0.425076 |
| H | 2.132828 | -1.585033 | 1.860365 |
| H | 2.854332 | -2.950994 | 1.016098 |
| H | 5.791414 | -1.914987 | -0.028203 |
| H | 1.255825 | -2.721284 | -0.830131 |
| H | 0.400592 | -2.885894 | 0.682697 |
| H | 2.850859 | -1.664740 | -1.930889 |
| H | 4.470790 | -1.019374 | -1.695446 |
| H | 4.058320 | -2.622710 | -1.093266 |
| H | 0.940020 | 2.185493 | -1.562383 |
| H | 0.459236 | 1.966158 | 0.100826 |
| H | -2.096679 | -1.295553 | 0.911261 |
| H | -1.686231 | -2.849282 | 0.204932 |
| H | 5.572487 | -2.768453 | 2.259593 |
| H | 7.259871 | -2.366829 | 1.934528 |
| H | 6.269155 | -1.281243 | 2.912638 |
| H | 7.494318 | -0.948141 | -1.459265 |
| H | 8.445380 | -1.326275 | -0.022028 |
| H | -1.521252 | 2.251704 | -1.614645 |
| H | -1.671884 | -2.013410 | -2.869129 |
| H | 0.042073 | -1.637714 | -2.791441 |
| H | -0.601193 | -3.044141 | -1.934053 |
| H | -3.444047 | 1.158091 | -2.065801 |
| H | -3.134284 | -0.388244 | -2.844276 |
| H | -3.471766 | -2.546811 | -1.520573 |
| H | -4.120704 | -2.459800 | 0.105252 |
| H | -4.071808 | 0.020398 | -0.001600 |
| H | 9.726245 | 0.266157 | -1.415546 |
| H | 8.355243 | 1.367524 | -1.465381 |
| H | 9.892739 | 0.558540 | 1.030655 |
| H | 8.379681 | 2.067641 | 2.233548 |
| H | 7.532414 | 2.447168 | 0.723882 |
| H | 10.534662 | 2.965242 | 1.115782 |
| H | 11.075137 | 2.239396 | -0.400462 |
| H | 9.638447 | 3.262941 | -0.377144 |
| H | -7.258530 | -0.403366 | -1.411617 |
| H | -5.676324 | -1.012031 | 1.134779 |
| H | -8.666954 | -0.690346 | 0.709679 |
| H | -8.262917 | 1.514390 | -0.459003 |
| H | -6.743037 | 1.146432 | 2.166036 |
| H | -7.556367 | -2.589648 | -0.258041 |
| H | -6.163900 | 3.284953 | 0.823788 |

| | | | |
|---|---|---|---|
| H | -7.914578 | 3.564456 | 0.976596 |
| H | -8.542469 | -0.784040 | 3.036776 |
| H | -8.632618 | 2.139426 | 2.716489 |
| H | -6.936762 | 4.676866 | -0.851899 |

Equilibrium geometry of itoaic acid (**9**)

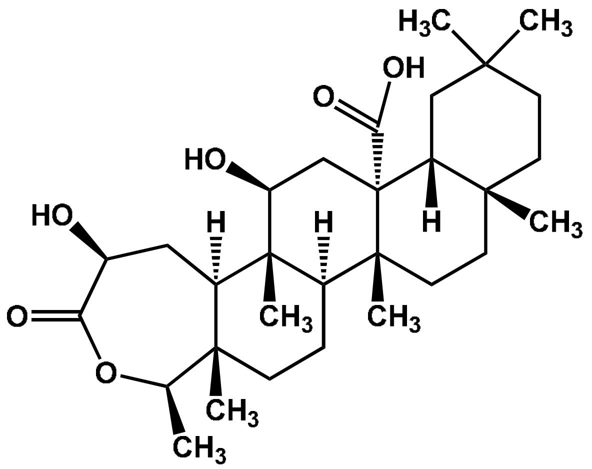


| | | | |
|---|---|---|---|
| C | -3.407752 | -1.105963 | -0.224295 |
| C | -2.544170 | 0.167520 | 0.056563 |
| C | -1.247516 | -0.085103 | 0.920009 |
| C | -0.415959 | -1.139573 | 0.107067 |
| C | -1.215906 | -2.431605 | -0.035039 |
| C | -2.496011 | -2.189054 | -0.820115 |
| C | -0.412654 | 1.219180 | 0.992315 |
| C | 1.070128 | 1.018467 | 1.320820 |
| C | 1.790694 | 0.039619 | 0.381811 |
| C | 1.068151 | -1.346848 | 0.526498 |
| C | 3.308724 | -0.090404 | 0.776018 |
| C | 4.079641 | -1.253570 | 0.100199 |
| C | 3.253460 | -2.552442 | 0.008172 |
| C | 1.775316 | -2.403157 | -0.349397 |
| C | 4.141068 | 1.221370 | 0.745008 |
| C | 5.043947 | 1.527703 | -0.490426 |
| C | 4.710036 | 0.606339 | -1.666235 |
| C | 4.586138 | -0.884832 | -1.322712 |
| C | -3.355835 | 1.372962 | 0.546364 |
| C | -4.424609 | 1.915123 | -0.411072 |
| C | -5.691312 | 1.088779 | -0.336505 |
| O | -5.654647 | -0.170741 | -0.737374 |
| C | -4.468828 | -0.775162 | -1.311549 |
| O | -4.738790 | 3.243107 | -0.071383 |
| O | -6.713564 | 1.548853 | 0.112807 |
| C | -5.027426 | -1.962191 | -2.078442 |
| C | -4.138870 | -1.665394 | 1.002964 |
| C | -1.598193 | -0.504716 | 2.363444 |
| O | -0.953806 | 2.080853 | 1.993650 |
| C | 1.144997 | -1.888592 | 1.968674 |

| | | | |
|---|---|---|---|
| C | 5.270194 | -1.590115 | 1.014943 |
| C | 6.540230 | 1.398939 | -0.163919 |
| C | 4.809209 | 2.980089 | -0.917325 |
| C | 1.684920 | 0.694441 | -1.000523 |
| O | 1.604066 | -0.116492 | -2.066306 |
| O | 1.698316 | 1.891270 | -1.156459 |
| H | -2.156206 | 0.458722 | -0.929439 |
| H | -0.363960 | -0.733074 | -0.908088 |
| H | -1.440404 | -2.872288 | 0.937271 |
| H | -0.637734 | -3.174967 | -0.579709 |
| H | -3.052516 | -3.123265 | -0.905301 |
| H | -2.214356 | -1.892963 | -1.836697 |
| H | -0.489835 | 1.713159 | 0.017064 |
| H | 1.165794 | 0.678110 | 2.350465 |
| H | 1.533266 | 2.002142 | 1.269542 |
| H | 3.238834 | -0.349722 | 1.834415 |
| H | 3.329935 | -3.084212 | 0.956763 |
| H | 3.736070 | -3.204046 | -0.725326 |
| H | 1.311308 | -3.374290 | -0.177271 |
| H | 1.649343 | -2.178684 | -1.404743 |
| H | 4.778075 | 1.215047 | 1.630737 |
| H | 3.465238 | 2.063074 | 0.884403 |
| H | 5.479096 | 0.732946 | -2.432963 |
| H | 3.789477 | 0.960975 | -2.123991 |
| H | 5.554404 | -1.373485 | -1.455440 |
| H | 3.921380 | -1.338230 | -2.059528 |
| H | -2.683929 | 2.211417 | 0.690391 |
| H | -3.820199 | 1.194846 | 1.518816 |
| H | -4.043736 | 1.901003 | -1.436526 |
| H | -4.038077 | -0.077442 | -2.032945 |
| H | -5.656829 | 3.244668 | 0.233913 |
| H | -4.271383 | -2.412970 | -2.714939 |
| H | -5.421165 | -2.721526 | -1.404028 |
| H | -5.842314 | -1.613677 | -2.709830 |
| H | -4.628190 | -0.888257 | 1.588297 |
| H | -4.912550 | -2.364729 | 0.685129 |
| H | -3.463439 | -2.205921 | 1.659546 |
| H | -1.618708 | -1.582171 | 2.497834 |
| H | -0.885497 | -0.095153 | 3.074380 |
| H | -2.570270 | -0.114967 | 2.655916 |
| H | -0.462042 | 2.907075 | 1.975306 |
| H | 0.726523 | -1.228307 | 2.715621 |
| H | 0.588453 | -2.826256 | 2.013667 |
| H | 2.163479 | -2.112009 | 2.271408 |
| H | 4.909357 | -1.917323 | 1.992235 |
| H | 5.853958 | -2.405490 | 0.583177 |
| H | 5.934760 | -0.747384 | 1.177320 |
| H | 6.872282 | 0.365275 | -0.091004 |
| H | 7.124870 | 1.865949 | -0.958980 |
| H | 6.784684 | 1.905415 | 0.771787 |
| H | 5.099643 | 3.664549 | -0.116639 |

| | | | |
|---|---|---|---|
| H | 5.406266 | 3.222574 | -1.799275 |
| H | 3.758771 | 3.149750 | -1.150500 |
| H | 1.574625 | 0.450444 | -2.851617 |

Equilibrium geometry of jaspamide Q (**10**)

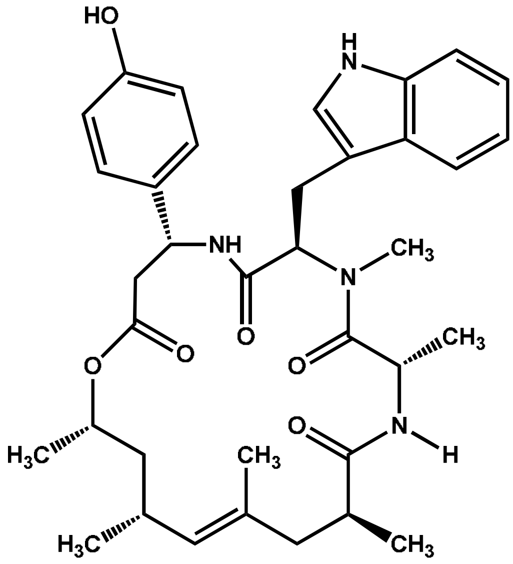


| | | | |
|---|---|---|---|
| H | -4.571376 | -0.713897 | -3.879176 |
| O | 4.185064 | 0.291683 | -0.973848 |
| O | -0.794533 | 2.543485 | -2.418770 |
| O | -1.441747 | 0.026352 | 1.386564 |
| O | 0.313452 | -4.377678 | 1.196744 |
| O | 2.610094 | 0.921698 | -2.439061 |
| O | 0.521335 | 7.050226 | 2.860218 |
| N | -1.747424 | -0.403372 | -0.809692 |
| N | 0.176665 | 1.704484 | -0.545181 |
| N | -0.601063 | -2.436721 | 1.893837 |
| N | -5.772499 | -1.471786 | -2.290232 |
| C | -2.075158 | 0.998500 | -1.069889 |
| C | -3.208307 | 1.172693 | -2.084825 |
| C | 4.759529 | -2.729754 | -0.711548 |
| C | 5.626454 | -1.598678 | -1.283090 |
| C | 1.327802 | 2.598285 | -0.552786 |
| C | -4.314520 | 0.186316 | -1.880361 |
| C | -0.833398 | 1.826508 | -1.434684 |
| C | 4.868866 | -0.486036 | -1.983095 |
| C | 1.543619 | -3.066011 | 2.810062 |
| C | 2.329087 | -1.859045 | 2.224934 |
| C | -1.513732 | -2.273825 | 0.782353 |
| C | -1.551091 | -0.782849 | 0.463916 |
| C | 2.828340 | -2.052178 | 0.812643 |
| C | 4.099719 | -2.379987 | 0.595013 |
| C | 2.600890 | 1.833468 | -0.207389 |
| C | -4.986777 | -0.152274 | -0.653538 |
| C | 0.363690 | -3.384582 | 1.907286 |

| | | | |
|---|---|---|---|
| C | 5.619115 | -3.979279 | -0.501239 |
| C | -1.591652 | -1.321600 | -1.935101 |
| C | 1.111662 | 3.776110 | 0.375300 |
| C | -4.826398 | -0.641991 | -2.835326 |
| C | -5.886644 | -1.201180 | -0.952553 |
| C | 5.781762 | 0.437976 | -2.766224 |
| C | 2.430685 | -4.280783 | 3.017093 |
| C | 3.100938 | 0.970766 | -1.339789 |
| C | -2.937403 | -2.737495 | 1.094708 |
| C | 1.831778 | -1.843130 | -0.299336 |
| C | -4.912191 | 0.311815 | 0.667639 |
| C | -6.688627 | -1.797997 | 0.019262 |
| C | 1.110144 | 5.074919 | -0.119273 |
| C | 0.907384 | 3.587053 | 1.739297 |
| C | -5.703898 | -0.276633 | 1.632129 |
| C | -6.581557 | -1.326028 | 1.311470 |
| C | 0.913721 | 6.162412 | 0.717949 |
| C | 0.707518 | 4.662573 | 2.588580 |
| C | 0.711715 | 5.955707 | 2.075550 |
| H | -2.402645 | 1.393593 | -0.110690 |
| H | -2.825352 | 1.093351 | -3.100785 |
| H | -3.563387 | 2.198571 | -1.978776 |
| H | 3.995928 | -2.971707 | -1.452358 |
| H | 6.254633 | -1.169057 | -0.498095 |
| H | 6.301712 | -2.027146 | -2.026333 |
| H | 1.414257 | 2.967734 | -1.571116 |
| H | 4.112332 | -0.909431 | -2.643833 |
| H | 1.143401 | -2.739625 | 3.772145 |
| H | 3.175574 | -1.681508 | 2.887855 |
| H | 1.694090 | -0.970368 | 2.269985 |
| H | -1.119746 | -2.853972 | -0.047294 |
| H | 4.755508 | -2.456082 | 1.460402 |
| H | -0.048056 | 1.228985 | 0.321412 |
| H | 2.474190 | 1.208205 | 0.679325 |
| H | 3.400058 | 2.535617 | 0.031401 |
| H | 5.027777 | -4.794081 | -0.085812 |
| H | 6.055742 | -4.313720 | -1.441132 |
| H | 6.432848 | -3.766364 | 0.193815 |
| H | -0.483946 | -1.614463 | 2.467586 |
| H | -1.683035 | -0.756359 | -2.855995 |
| H | -2.361047 | -2.091362 | -1.933470 |
| H | -0.605725 | -1.781623 | -1.921499 |
| H | 6.271320 | -0.113520 | -3.566191 |
| H | 5.214882 | 1.254523 | -3.207830 |
| H | 6.546993 | 0.852001 | -2.110628 |
| H | 1.865641 | -5.108749 | 3.439897 |
| H | 2.859579 | -4.609944 | 2.073139 |
| H | 3.244882 | -4.034193 | 3.696730 |
| H | -6.290943 | -2.170350 | -2.792276 |
| H | -2.926294 | -3.798583 | 1.332098 |
| H | -3.331660 | -2.183501 | 1.944864 |

| | | | |
|---|---|---|---|
| H | -3.597843 | -2.567223 | 0.243446 |
| H | 1.147527 | -2.687277 | -0.392485 |
| H | 2.312939 | -1.705018 | -1.263812 |
| H | 1.226710 | -0.951968 | -0.103700 |
| H | -4.248545 | 1.121067 | 0.937885 |
| H | -7.365772 | -2.601802 | -0.232714 |
| H | 1.263093 | 5.242119 | -1.177296 |
| H | 0.900131 | 2.587468 | 2.157312 |
| H | -5.649650 | 0.072378 | 2.653444 |
| H | -7.186321 | -1.768142 | 2.090263 |
| H | 0.911538 | 7.171256 | 0.331364 |
| H | 0.549130 | 4.502916 | 3.646984 |
| H | 0.391465 | 6.784271 | 3.775197 |

Equilibrium geometry of korundamine A (**11**)

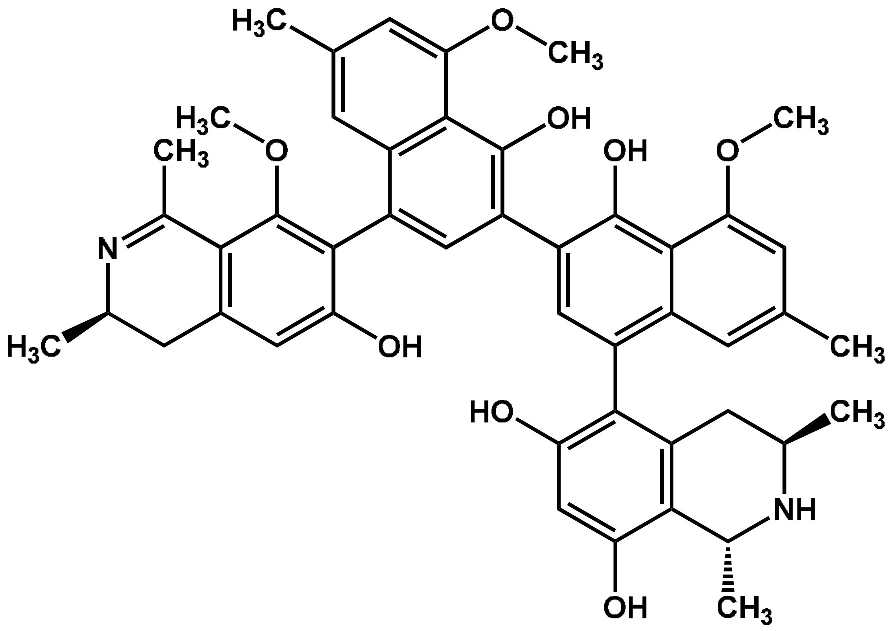


| | | | |
|---|---|---|---|
| C | 6.711090 | -4.269977 | 0.873019 |
| N | 6.633041 | -3.452999 | 2.088490 |
| C | 5.760378 | -2.523900 | 2.143585 |
| C | 4.792621 | -2.271343 | 1.040323 |
| C | 4.539675 | -3.326252 | 0.154453 |
| C | 5.316183 | -4.594082 | 0.351886 |
| C | 4.108574 | -1.064660 | 0.849438 |
| C | 3.165399 | -0.904542 | -0.171863 |
| C | 2.909083 | -1.997378 | -1.002254 |
| C | 3.599510 | -3.194553 | -0.847899 |
| C | 2.475162 | 0.390649 | -0.405946 |
| C | 1.129265 | 0.485923 | -0.188584 |
| C | 0.405822 | 1.687910 | -0.341801 |
| C | 1.100600 | 2.812302 | -0.738171 |
| C | 2.501148 | 2.769030 | -0.999640 |
| C | 3.194552 | 1.539323 | -0.840774 |

| | | | |
|---|---|---|---|
| C | 4.582982 | 1.478198 | -1.122961 |
| C | 5.277162 | 2.575556 | -1.541992 |
| C | 4.597096 | 3.807744 | -1.694996 |
| C | 3.259824 | 3.903603 | -1.435986 |
| C | -1.062880 | 1.651659 | -0.117753 |
| C | -1.778632 | 0.593888 | -0.719178 |
| C | -3.111991 | 0.390380 | -0.513255 |
| C | -3.821618 | 1.273923 | 0.349017 |
| C | -3.135622 | 2.351208 | 0.978657 |
| C | -1.742435 | 2.531836 | 0.709537 |
| C | -3.905060 | 3.196033 | 1.855709 |
| C | -5.245933 | 2.970382 | 2.042589 |
| C | -5.916861 | 1.909173 | 1.395561 |
| C | -5.209717 | 1.083893 | 0.572093 |
| C | -3.801675 | -0.748174 | -1.182154 |
| C | -4.213340 | -0.622164 | -2.507143 |
| C | -4.876970 | -1.655160 | -3.150708 |
| C | -5.129047 | -2.830302 | -2.464179 |
| C | -4.725315 | -3.003177 | -1.137478 |
| C | -4.064641 | -1.946532 | -0.506167 |
| C | -3.661408 | -2.070782 | 0.944175 |
| C | -3.682007 | -3.504677 | 1.461525 |
| N | -4.888758 | -4.216925 | 1.037663 |
| C | -5.012322 | -4.312360 | -0.416343 |
| C | 7.589629 | -3.578588 | -0.166740 |
| C | 5.692459 | -1.715780 | 3.412337 |
| O | 1.993833 | -1.833058 | -1.990862 |
| O | 4.447955 | -0.004663 | 1.634894 |
| C | 3.426986 | 0.619655 | 2.412605 |
| C | 6.745343 | 2.516623 | -1.849156 |
| O | 2.567078 | 5.072040 | -1.575165 |
| C | 3.260038 | 6.232535 | -2.014932 |
| O | 0.383201 | 3.962364 | -0.881537 |
| O | -1.086960 | 3.553393 | 1.313093 |
| O | -3.255813 | 4.201537 | 2.483664 |
| C | -3.996177 | 5.035259 | 3.354421 |
| C | -7.386491 | 1.721723 | 1.639675 |
| O | -3.957720 | 0.556248 | -3.142922 |
| O | -5.801364 | -3.793780 | -3.159834 |
| C | -4.126811 | -5.448305 | -0.939428 |
| C | -3.573738 | -3.543262 | 2.975415 |
| H | -5.695197 | -3.720694 | 1.405815 |
| H | 7.197548 | -5.202599 | 1.158268 |
| H | 5.370612 | -5.160184 | -0.578019 |
| H | 4.800806 | -5.220364 | 1.086730 |
| H | 3.406733 | -4.018315 | -1.524887 |
| H | 0.590134 | -0.392882 | 0.143237 |
| H | 5.095308 | 0.533309 | -1.005766 |
| H | 5.156518 | 4.670960 | -2.023633 |
| H | -1.240170 | -0.079880 | -1.375338 |
| H | -5.706873 | 0.263726 | 0.071176 |

| | | | |
|---|---|---|---|
| H | -5.818139 | 3.609256 | 2.697994 |
| H | -5.210240 | -1.554846 | -4.175681 |
| H | -4.348122 | -1.465716 | 1.548982 |
| H | -2.671016 | -1.635434 | 1.088414 |
| H | -2.829462 | -4.039065 | 1.036985 |
| H | -6.056329 | -4.586655 | -0.600398 |
| H | 7.722500 | -4.221179 | -1.037129 |
| H | 7.140734 | -2.639565 | -0.492963 |
| H | 8.569491 | -3.360644 | 0.255936 |
| H | 6.111886 | -0.722277 | 3.264899 |
| H | 4.659908 | -1.584068 | 3.736105 |
| H | 6.255489 | -2.234445 | 4.183652 |
| H | 1.927787 | -2.640160 | -2.512352 |
| H | 2.577828 | -0.050828 | 2.545168 |
| H | 3.866006 | 0.845831 | 3.382788 |
| H | 3.090594 | 1.540638 | 1.937779 |
| H | 7.142124 | 1.517647 | -1.682421 |
| H | 6.932780 | 2.795497 | -2.886992 |
| H | 7.297752 | 3.218090 | -1.222547 |
| H | 4.048100 | 6.495152 | -1.309714 |
| H | 3.684776 | 6.069386 | -3.005083 |
| H | 2.520480 | 7.024756 | -2.055261 |
| H | 0.980800 | 4.676697 | -1.168726 |
| H | -0.411419 | 3.884032 | 0.698817 |
| H | -3.289009 | 5.759563 | 3.746207 |
| H | -4.793255 | 5.555317 | 2.820078 |
| H | -4.426357 | 4.462328 | 4.177933 |
| H | -7.942908 | 2.611997 | 1.343003 |
| H | -7.773085 | 0.871269 | 1.082149 |
| H | -7.583260 | 1.558840 | 2.700328 |
| H | -4.300541 | 0.522119 | -4.041800 |
| H | -5.987980 | -4.554245 | -2.602069 |
| H | -4.417383 | -6.389172 | -0.474745 |
| H | -4.202055 | -5.553764 | -2.021951 |
| H | -3.079855 | -5.253348 | -0.711099 |
| H | -4.450104 | -3.071609 | 3.425863 |
| H | -3.514935 | -4.570326 | 3.332224 |
| H | -2.689998 | -3.002076 | 3.313097 |

Equilibrium geometry of limonin (**12**)

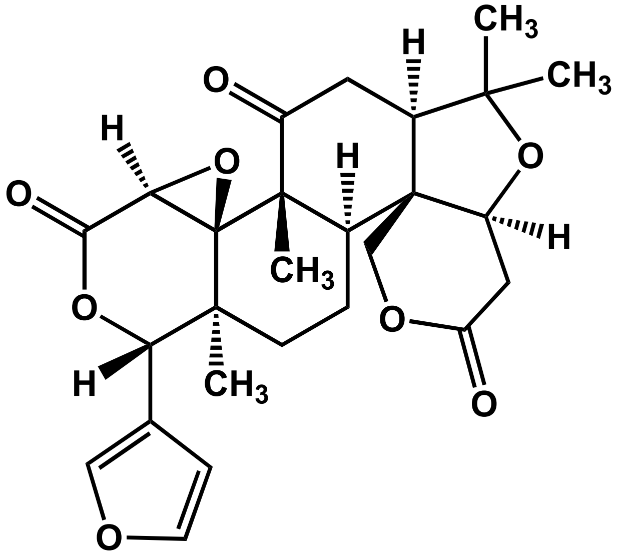


| | | | |
|---|---|---|---|
| O | -1.973063 | -1.231342 | -2.049429 |
| O | 3.984992 | 0.948083 | 1.322761 |
| O | -4.222653 | -1.048697 | -0.223655 |
| O | 4.263902 | 0.724640 | -1.564344 |
| O | -0.183371 | -2.898039 | 0.951474 |
| O | -4.177136 | -3.249158 | -0.210668 |
| O | 5.451520 | 2.567432 | -1.443803 |
| O | -5.469064 | 3.156413 | 0.535721 |
| C | -1.402377 | -0.998736 | -0.762088 |
| C | 0.132844 | -1.141237 | -0.676736 |
| C | 0.716731 | 0.255105 | -0.261419 |
| C | 2.248909 | 0.244229 | -0.098157 |
| C | -2.070106 | 0.167015 | -0.040273 |
| C | -2.234736 | -2.178448 | -1.028657 |
| C | 2.504290 | -0.797926 | 1.008195 |
| C | 0.009782 | 1.333037 | -1.075309 |
| C | -1.410783 | 1.498350 | -0.504793 |
| C | 2.842733 | 1.458464 | 0.628551 |
| C | 0.586405 | -2.180329 | 0.364317 |
| C | 2.087891 | -2.215236 | 0.645541 |
| C | 3.895914 | -0.479673 | 1.552966 |
| C | -3.552696 | 0.204521 | -0.487327 |
| C | 0.621612 | -1.669696 | -2.040234 |
| C | 2.990278 | 0.075447 | -1.441527 |
| C | -2.006504 | -0.061760 | 1.477181 |
| C | -3.622156 | -2.216719 | -0.470294 |
| C | 3.342874 | 2.550916 | -0.296204 |
| C | 5.036511 | -1.191513 | 0.841726 |
| C | 3.984806 | -0.723662 | 3.051243 |
| C | -4.368789 | 1.254770 | 0.189295 |
| C | 4.443053 | 1.987321 | -1.149839 |
| C | -4.927226 | 1.234153 | 1.513010 |
| C | -4.742048 | 2.439083 | -0.348457 |

| | | | |
|---|---|---|---|
| C | -5.577718 | 2.407187 | 1.660751 |
| H | 0.380851 | 0.431749 | 0.759863 |
| H | -1.790160 | -3.156098 | -1.157602 |
| H | 1.830807 | -0.493153 | 1.818247 |
| H | -0.030035 | 1.072647 | -2.134503 |
| H | 0.523837 | 2.290134 | -1.004394 |
| H | -1.377269 | 2.177145 | 0.348387 |
| H | -2.040177 | 1.976967 | -1.255302 |
| H | 2.115496 | 1.862212 | 1.340876 |
| H | 2.252035 | -2.908223 | 1.469025 |
| H | 2.634902 | -2.587681 | -0.222327 |
| H | -3.576879 | 0.374885 | -1.561396 |
| H | 0.494964 | -0.925963 | -2.823125 |
| H | 1.656816 | -1.982603 | -2.015928 |
| H | 0.035032 | -2.540906 | -2.324613 |
| H | 3.226707 | -0.961184 | -1.655662 |
| H | 2.357877 | 0.448539 | -2.248889 |
| H | -2.302221 | 0.841766 | 2.007590 |
| H | -2.680026 | -0.867961 | 1.770767 |
| H | -1.016595 | -0.346491 | 1.822861 |
| H | 3.745287 | 3.394169 | 0.258371 |
| H | 2.541118 | 2.916699 | -0.939910 |
| H | 5.001204 | -1.068709 | -0.237491 |
| H | 5.985617 | -0.788758 | 1.194632 |
| H | 5.010155 | -2.256567 | 1.076808 |
| H | 3.228012 | -0.141715 | 3.576746 |
| H | 3.832980 | -1.780521 | 3.275328 |
| H | 4.967569 | -0.432553 | 3.420599 |
| H | -4.861660 | 0.439831 | 2.235800 |
| H | -4.578785 | 2.889180 | -1.312359 |
| H | -6.145423 | 2.840523 | 2.464357 |

Equilibrium geometry of matopensine (**13**)

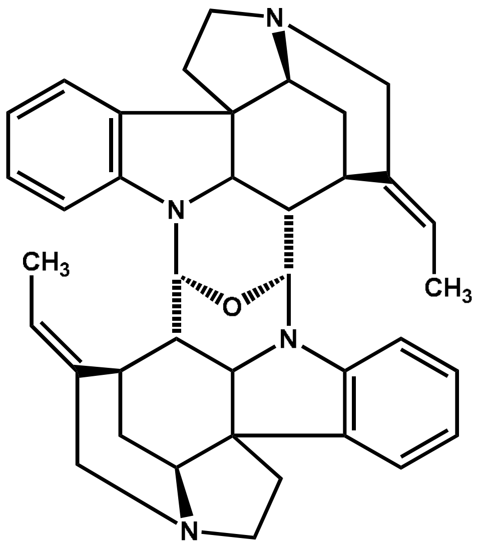


| | | | |
|---|---|---|---|
| O | 0.000000 | 0.000000 | 2.415838 |
| N | 0.546855 | 1.412037 | 0.641547 |
| N | 4.247457 | 0.364652 | -1.715275 |
| C | 1.879052 | 0.852221 | 0.956094 |
| C | 2.799674 | 1.501048 | -0.087960 |
| C | 1.859927 | -0.673920 | 1.053734 |
| C | -0.476838 | 1.081532 | 1.627294 |
| C | 2.841561 | 0.732739 | -1.468123 |
| C | 2.303187 | -1.370187 | -0.253873 |
| C | 1.950367 | -0.499241 | -1.463950 |
| C | 4.269070 | 1.553663 | 0.354113 |
| C | 2.119369 | 2.845074 | -0.208717 |
| C | 0.805144 | 2.738349 | 0.268427 |
| C | 5.040300 | 1.317412 | -0.944930 |
| C | 3.804224 | -1.652086 | -0.268249 |
| C | 4.542106 | -1.045264 | -1.439338 |
| C | 2.605494 | 4.050023 | -0.663790 |
| C | 0.000000 | 3.864512 | 0.354665 |
| C | 4.440341 | -2.415482 | 0.615797 |
| C | 1.785799 | 5.180175 | -0.620776 |
| C | 0.505448 | 5.081727 | -0.103897 |
| C | 3.837563 | -3.175576 | 1.757148 |
| H | 2.181287 | 1.251929 | 1.932946 |
| H | 2.575675 | -0.931796 | 1.831854 |
| H | -0.596729 | 1.915794 | 2.327130 |
| H | 2.524848 | 1.428037 | -2.246944 |
| H | 1.778277 | -2.316938 | -0.350937 |
| H | 2.084497 | -1.054191 | -2.391835 |
| H | 0.909952 | -0.202015 | -1.408751 |
| H | 4.472234 | 0.742463 | 1.055450 |
| H | 4.516037 | 2.494141 | 0.843009 |
| H | 5.110431 | 2.249952 | -1.510523 |
| H | 6.051591 | 0.946670 | -0.793746 |
| H | 4.282730 | -1.619586 | -2.332876 |

| | | | |
|---|---|---|---|
| H | 5.616942 | -1.153340 | -1.307126 |
| H | 3.621135 | 4.128114 | -1.030121 |
| H | -0.993146 | 3.826173 | 0.774801 |
| H | 5.514014 | -2.528033 | 0.500626 |
| H | 2.157480 | 6.132431 | -0.969505 |
| H | -0.117881 | 5.962617 | -0.043121 |
| H | 2.751226 | -3.136534 | 1.760808 |
| H | 4.133354 | -4.223873 | 1.712614 |
| H | 4.186895 | -2.790351 | 2.716985 |
| N | -0.546855 | -1.412037 | 0.641547 |
| N | -4.247457 | -0.364652 | -1.715275 |
| C | -1.879052 | -0.852221 | 0.956094 |
| C | -2.799674 | -1.501048 | -0.087960 |
| C | -1.859927 | 0.673920 | 1.053734 |
| C | 0.476838 | -1.081532 | 1.627294 |
| C | -2.841561 | -0.732739 | -1.468123 |
| C | -2.303187 | 1.370187 | -0.253873 |
| C | -1.950367 | 0.499241 | -1.463950 |
| C | -4.269070 | -1.553663 | 0.354113 |
| C | -2.119369 | -2.845074 | -0.208717 |
| C | -0.805144 | -2.738349 | 0.268427 |
| C | -5.040300 | -1.317412 | -0.944930 |
| C | -3.804224 | 1.652086 | -0.268249 |
| C | -4.542106 | 1.045264 | -1.439338 |
| C | -2.605494 | -4.050023 | -0.663790 |
| C | -0.000000 | -3.864512 | 0.354665 |
| C | -4.440341 | 2.415482 | 0.615797 |
| C | -1.785799 | -5.180175 | -0.620776 |
| C | -0.505448 | -5.081727 | -0.103897 |
| C | -3.837563 | 3.175576 | 1.757148 |
| H | -2.181287 | -1.251929 | 1.932946 |
| H | -2.575675 | 0.931796 | 1.831854 |
| H | 0.596729 | -1.915794 | 2.327130 |
| H | -2.524848 | -1.428037 | -2.246944 |
| H | -1.778277 | 2.316938 | -0.350937 |
| H | -2.084497 | 1.054191 | -2.391835 |
| H | -0.909952 | 0.202015 | -1.408751 |
| H | -4.472234 | -0.742463 | 1.055450 |
| H | -4.516037 | -2.494141 | 0.843009 |
| H | -5.110431 | -2.249952 | -1.510523 |
| H | -6.051591 | -0.946670 | -0.793746 |
| H | -4.282730 | 1.619586 | -2.332876 |
| H | -5.616942 | 1.153340 | -1.307126 |
| H | -3.621135 | -4.128114 | -1.030121 |
| H | 0.993146 | -3.826173 | 0.774801 |
| H | -5.514014 | 2.528033 | 0.500626 |
| H | -2.157480 | -6.132431 | -0.969505 |
| H | 0.117881 | -5.962617 | -0.043121 |
| H | -2.751226 | 3.136534 | 1.760808 |
| H | -4.133354 | 4.223873 | 1.712614 |
| H | -4.186895 | 2.790351 | 2.716985 |

Equilibrium geometry of mulberrofuran G (**14**)

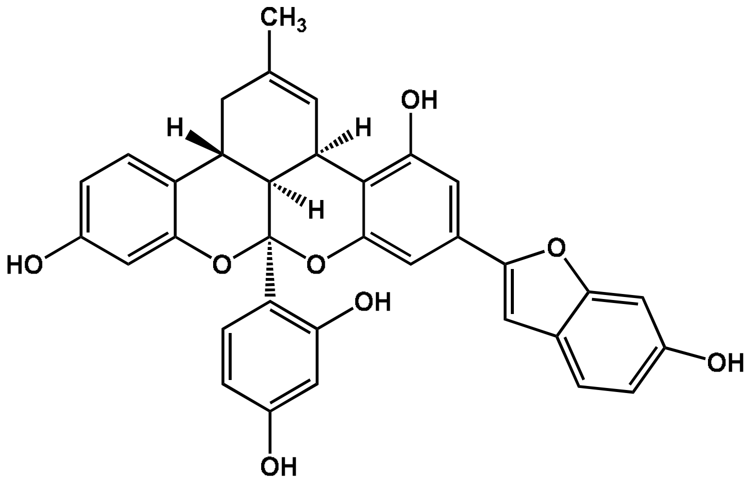


| | | | |
|---|---|---|---|
| O | -3.167348 | 0.347493 | -1.355271 |
| O | -1.054098 | -0.147690 | -0.756672 |
| O | 0.923959 | 0.691685 | 3.547273 |
| O | -0.594341 | 1.964002 | -2.281951 |
| O | -5.922384 | -2.490449 | -3.848427 |
| O | 4.774994 | -0.265103 | 0.642972 |
| O | -1.468443 | 6.208455 | -0.275245 |
| O | 9.504759 | -0.809774 | -0.154773 |
| C | -2.644899 | -0.023646 | 0.993124 |
| C | -2.845861 | -1.532871 | 0.822177 |
| C | -1.582552 | 0.259587 | 2.065746 |
| C | -2.215317 | 0.573471 | -0.346602 |
| C | -3.428336 | -2.106211 | 2.106569 |
| C | -3.676832 | -1.822157 | -0.401980 |
| C | -0.185117 | 0.159255 | 1.469000 |
| C | -1.796354 | -0.643441 | 3.264892 |
| C | -2.645041 | -1.666229 | 3.310813 |
| C | -1.969520 | 2.074439 | -0.336852 |
| C | -3.777459 | -0.878210 | -1.420655 |
| C | 0.001246 | -0.091682 | 0.105326 |
| C | 0.972294 | 0.322635 | 2.234918 |
| C | -4.357778 | -3.022584 | -0.585057 |
| C | -2.873886 | -2.458366 | 4.562758 |
| C | 1.254385 | -0.300558 | -0.445877 |
| C | -4.529572 | -1.105149 | -2.564147 |
| C | -1.176557 | 2.703538 | -1.305803 |
| C | -2.606208 | 2.893575 | 0.593020 |
| C | 2.237269 | 0.114675 | 1.708088 |
| C | 2.381454 | -0.225417 | 0.366533 |
| C | -5.110571 | -3.280697 | -1.718018 |
| C | -5.196751 | -2.309483 | -2.709765 |
| C | -0.993954 | 4.083168 | -1.286378 |
| C | -2.447434 | 4.266013 | 0.618179 |
| C | -1.621426 | 4.860889 | -0.326817 |
| C | 3.703304 | -0.469864 | -0.196001 |

| | | | |
|---|---|---|---|
| C | 4.114963 | -0.885380 | -1.416676 |
| C | 5.550090 | -0.954011 | -1.362703 |
| C | 5.891041 | -0.559735 | -0.068398 |
| C | 6.576851 | -1.301947 | -2.244083 |
| C | 7.189551 | -0.487021 | 0.405879 |
| C | 7.882700 | -1.240832 | -1.802750 |
| C | 8.184528 | -0.837041 | -0.493029 |
| H | -3.598689 | 0.412467 | 1.296316 |
| H | -1.858712 | -1.986352 | 0.686704 |
| H | -1.717949 | 1.288762 | 2.416273 |
| H | -3.424005 | -3.197423 | 2.074354 |
| H | -4.477293 | -1.808209 | 2.215547 |
| H | -1.250600 | -0.407082 | 4.170524 |
| H | -4.299578 | -3.784831 | 0.180226 |
| H | -3.933814 | -2.448826 | 4.827406 |
| H | -2.302969 | -2.064426 | 5.401412 |
| H | -2.596962 | -3.503481 | 4.409102 |
| H | 1.326440 | -0.496352 | -1.505955 |
| H | -4.589738 | -0.346447 | -3.331649 |
| H | -3.264111 | 2.451681 | 1.327571 |
| H | 3.096223 | 0.231806 | 2.351871 |
| H | -5.627593 | -4.224451 | -1.833245 |
| H | -0.365438 | 4.547096 | -2.037828 |
| H | -2.950454 | 4.877212 | 1.352916 |
| H | 0.064585 | 1.063562 | 3.765670 |
| H | -0.102523 | 2.541083 | -2.875459 |
| H | -6.336596 | -3.359271 | -3.838817 |
| H | 3.482061 | -1.125806 | -2.253454 |
| H | -0.881823 | 6.508525 | -0.977380 |
| H | 6.357782 | -1.614038 | -3.255933 |
| H | 7.412617 | -0.177742 | 1.417948 |
| H | 8.703427 | -1.502551 | -2.455820 |
| H | 9.609413 | -0.516138 | 0.755875 |

Equilibrium geometry of 1$\alpha$,8$\beta$,9$\beta$,14$\alpha$,15$\beta$-pentaacetoxy-3$\beta$-benzoyloxy-7-oxojatropha-5,12-diene (**15**)

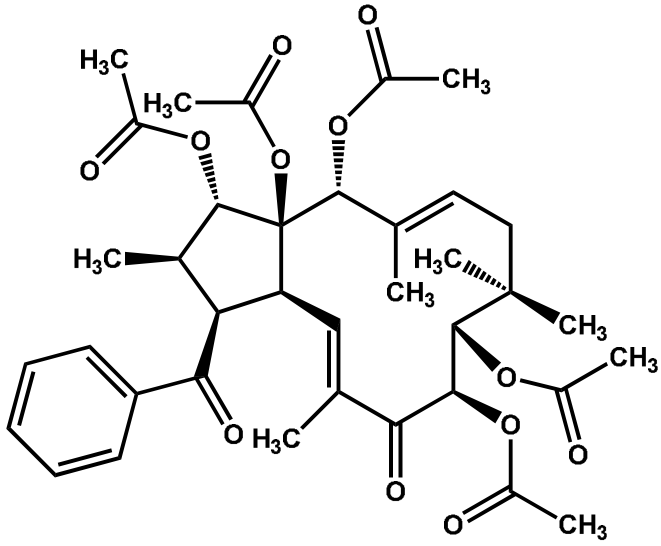

| | | | |
|---|---|---|---|
| C | 2.975370 | 0.820288 | -0.836379 |
| C | 3.275052 | -0.352941 | 0.102683 |
| C | 1.901057 | -0.812702 | 0.709248 |
| C | 0.891778 | 0.253588 | 0.211829 |
| C | 1.757576 | 1.456967 | -0.172784 |
| C | 4.148682 | 1.766222 | -1.028125 |
| O | 2.149733 | 2.132976 | 1.031543 |
| C | 2.437976 | 3.449153 | 1.064568 |
| O | 3.270409 | 3.841679 | 1.834816 |
| C | 1.659956 | 4.377146 | 0.192231 |
| C | 2.327701 | 5.457142 | -0.374509 |
| C | 1.627396 | 6.379596 | -1.135199 |
| C | 0.256276 | 6.240108 | -1.304103 |
| C | -0.414311 | 5.173556 | -0.719985 |
| C | 0.286528 | 4.233619 | 0.018975 |
| O | 4.096083 | -1.422722 | -0.341328 |
| C | 4.204528 | -2.024529 | -1.544010 |
| O | 4.553853 | -3.174927 | -1.540750 |
| C | 3.977093 | -1.275413 | -2.826441 |
| O | 1.932449 | -0.692973 | 2.137064 |
| C | 2.832435 | -1.359577 | 2.885592 |
| O | 3.615701 | -2.149056 | 2.437821 |
| C | 2.712372 | -0.963548 | 4.325346 |
| C | 1.492602 | -2.247263 | 0.309544 |
| C | 0.185144 | -2.722896 | 0.916646 |
| C | 0.313953 | -3.282311 | 2.310673 |
| C | -0.996496 | -2.745460 | 0.299815 |
| O | 1.467740 | -2.230509 | -1.120077 |
| C | 1.531788 | -3.376366 | -1.841642 |
| O | 1.421819 | -3.279192 | -3.032424 |
| C | 1.749434 | -4.669110 | -1.114733 |
| C | -1.376214 | -2.287195 | -1.085998 |

| | | | |
|---|---|---|---|
| C | -2.882851 | -2.179805 | -1.401700 |
| C | -3.655992 | -1.129150 | -0.582371 |
| C | -3.569685 | -3.528448 | -1.158462 |
| C | -3.013941 | -1.831037 | -2.888504 |
| O | -3.457446 | -1.360890 | 0.805922 |
| C | -4.448156 | -1.605190 | 1.688963 |
| O | -4.135759 | -1.762669 | 2.836197 |
| C | -5.866047 | -1.670942 | 1.196755 |
| C | -3.375768 | 0.353104 | -0.859705 |
| C | -1.956906 | 0.848839 | -0.524028 |
| C | -1.476884 | 0.928930 | 0.884209 |
| O | -1.253256 | 1.164284 | -1.460146 |
| O | -4.363983 | 1.047723 | -0.102977 |
| C | -4.766123 | 2.304678 | -0.412968 |
| O | -5.548267 | 2.831466 | 0.323395 |
| C | -4.227192 | 2.935524 | -1.664477 |
| C | -0.205106 | 0.618281 | 1.159810 |
| C | -2.406512 | 1.378228 | 1.980763 |
| H | 2.609415 | 0.459611 | -1.794160 |
| H | 3.838973 | 0.054863 | 0.940655 |
| H | 0.495307 | -0.106304 | -0.732267 |
| H | 1.229919 | 2.130617 | -0.839949 |
| H | 5.003114 | 1.257986 | -1.470355 |
| H | 3.876627 | 2.597190 | -1.678467 |
| H | 4.475440 | 2.168201 | -0.069756 |
| H | 3.390891 | 5.564151 | -0.212961 |
| H | 2.147810 | 7.211068 | -1.587346 |
| H | -0.291575 | 6.966071 | -1.887431 |
| H | -1.483443 | 5.076262 | -0.839691 |
| H | -0.230460 | 3.398120 | 0.473375 |
| H | 2.295799 | -2.904483 | 0.641395 |
| H | 0.424285 | -2.489296 | 3.049907 |
| H | -0.571198 | -3.853733 | 2.576725 |
| H | 1.186878 | -3.931794 | 2.395602 |
| H | -1.799646 | -3.187260 | 0.874649 |
| H | -0.997294 | -3.012320 | -1.812408 |
| H | -0.884241 | -1.355601 | -1.350630 |
| H | -4.709039 | -1.250452 | -0.836817 |
| H | -3.623386 | -3.783646 | -0.101196 |
| H | -4.585218 | -3.516536 | -1.555949 |
| H | -3.020942 | -4.321099 | -1.665650 |
| H | -2.667360 | -2.669002 | -3.491136 |
| H | -4.051875 | -1.631960 | -3.159107 |
| H | -2.411489 | -0.962440 | -3.153228 |
| H | -3.514153 | 0.532047 | -1.923276 |
| H | 0.091754 | 0.671985 | 2.198816 |
| H | -3.227212 | 0.686050 | 2.138702 |
| H | -1.847913 | 1.468206 | 2.908613 |
| H | -2.842018 | 2.351856 | 1.752760 |
| H | 4.426261 | -1.859337 | -3.621805 |
| H | 2.908794 | -1.202358 | -3.019455 |

| | | | |
|---|---|---|---|
| H | 4.410962 | -0.282114 | -2.803762 |
| H | 3.361337 | -1.586311 | 4.929094 |
| H | 2.997838 | 0.082049 | 4.427611 |
| H | 1.679800 | -1.056872 | 4.654112 |
| H | 2.716495 | -4.646348 | -0.615253 |
| H | 0.974102 | -4.828005 | -0.368104 |
| H | 1.738099 | -5.471052 | -1.842670 |
| H | -6.501862 | -1.883461 | 2.047237 |
| H | -6.156006 | -0.723144 | 0.748019 |
| H | -5.982739 | -2.455775 | 0.451220 |
| H | -4.580659 | 3.958332 | -1.705717 |
| H | -3.140186 | 2.913630 | -1.694630 |
| H | -4.591653 | 2.394357 | -2.536919 |

Equilibrium geometry of pedunculagin (**16**)

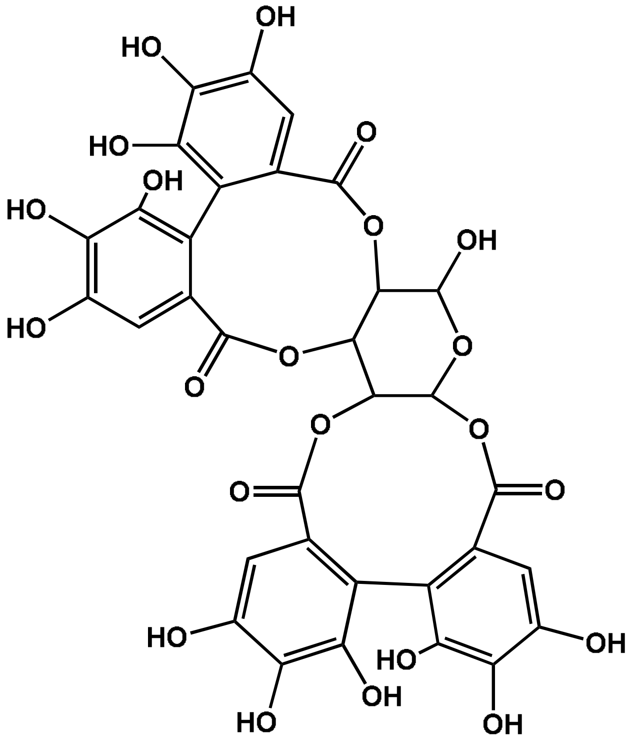


| | | | |
|---|---|---|---|
| O | -3.109212 | -2.666988 | -0.936707 |
| C | -4.103065 | -2.063602 | -0.257714 |
| C | -4.650483 | -0.894947 | -1.006124 |
| O | -4.494237 | -2.421603 | 0.821285 |
| C | -2.374676 | -3.689140 | -0.250169 |
| C | -0.640452 | -1.812589 | -0.179639 |
| C | -0.901401 | -3.318478 | -0.119155 |
| O | -0.171945 | -3.935345 | -1.179194 |
| C | 1.216644 | -3.818180 | -1.067534 |
| C | 1.604392 | -2.341258 | -1.040349 |
| C | 0.837089 | -1.591533 | 0.048396 |
| H | 1.631347 | -4.296636 | -1.955915 |
| O | 1.631323 | -4.478527 | 0.099721 |

| | | | |
|---|---|---|---|
| O | 2.994479 | -2.235349 | -0.742386 |
| C | 3.676528 | -1.185299 | -1.274375 |
| O | 3.314139 | -0.594384 | -2.252274 |
| C | 4.873889 | -0.861211 | -0.451788 |
| C | 5.718370 | -1.904255 | -0.096870 |
| C | 6.849105 | -1.651533 | 0.656868 |
| C | 7.113098 | -0.351256 | 1.064496 |
| C | 6.250188 | 0.685944 | 0.730530 |
| C | 5.098435 | 0.457677 | -0.026725 |
| O | 1.159808 | -0.208431 | -0.083712 |
| C | 2.205162 | 0.260220 | 0.637092 |
| O | 2.630031 | -0.274554 | 1.623400 |
| C | 2.766514 | 1.487856 | 0.004417 |
| C | 4.138176 | 1.569214 | -0.277082 |
| C | 4.581685 | 2.749016 | -0.872431 |
| C | 3.715968 | 3.791427 | -1.170774 |
| C | 2.361752 | 3.671471 | -0.898002 |
| C | 1.883603 | 2.508659 | -0.321494 |
| O | 5.904167 | 2.893964 | -1.175596 |
| O | 4.250095 | 4.899294 | -1.743440 |
| O | 1.602866 | 4.745085 | -1.236824 |
| O | 6.595365 | 1.900617 | 1.250547 |
| O | 8.230314 | -0.134644 | 1.800691 |
| O | 7.691762 | -2.660344 | 0.984529 |
| O | -1.427352 | -1.182547 | 0.826379 |
| C | -2.102348 | -0.068015 | 0.461598 |
| C | -3.130680 | 0.302777 | 1.471999 |
| O | -1.905855 | 0.515693 | -0.569156 |
| C | -2.750761 | 0.386472 | 2.803627 |
| C | -3.670863 | 0.789276 | 3.755180 |
| C | -4.966422 | 1.097655 | 3.361274 |
| C | -5.347889 | 0.985174 | 2.029350 |
| C | -4.443603 | 0.565141 | 1.054719 |
| C | -4.887407 | 0.317501 | -0.342338 |
| C | -5.492019 | 1.332251 | -1.078013 |
| C | -5.829947 | 1.163448 | -2.413534 |
| C | -5.551073 | -0.034314 | -3.054611 |
| C | -4.952954 | -1.064721 | -2.350457 |
| O | -3.305009 | 0.898652 | 5.055401 |
| O | -5.838153 | 1.500361 | 4.318752 |
| O | -6.662704 | 1.257399 | 1.783783 |
| O | -5.757031 | 2.528044 | -0.477552 |
| O | -6.417251 | 2.209773 | -3.045881 |
| O | -5.913910 | -0.090802 | -4.362497 |
| H | -2.458503 | -4.607659 | -0.826742 |
| H | -2.812739 | -3.834020 | 0.731424 |
| H | -0.931708 | -1.428886 | -1.156237 |
| H | -0.540768 | -3.712046 | 0.833318 |
| H | 1.395723 | -1.896494 | -2.012393 |
| H | 1.128245 | -1.943099 | 1.037815 |
| H | 2.559087 | -4.272941 | 0.265018 |

| | | | |
|---|---|---|---|
| H | 5.512928 | -2.915959 | -0.416713 |
| H | 0.826506 | 2.400720 | -0.115840 |
| H | 6.037525 | 3.742829 | -1.619659 |
| H | 3.549665 | 5.537331 | -1.930695 |
| H | 0.675356 | 4.592633 | -1.027450 |
| H | 6.528679 | 2.572308 | 0.553083 |
| H | 8.288207 | 0.810793 | 1.996084 |
| H | 8.422107 | -2.304691 | 1.506462 |
| H | -1.738162 | 0.160848 | 3.107088 |
| H | -4.746226 | -2.006286 | -2.841280 |
| H | -4.065501 | 1.191928 | 5.573233 |
| H | -6.685642 | 1.699119 | 3.897984 |
| H | -6.729264 | 1.800826 | 0.983221 |
| H | -6.145029 | 3.131936 | -1.125595 |
| H | -6.583880 | 1.977768 | -3.968542 |
| H | -5.698815 | -0.948312 | -4.744010 |

Equilibrium geometry of phyllaemblicin B (**17**)

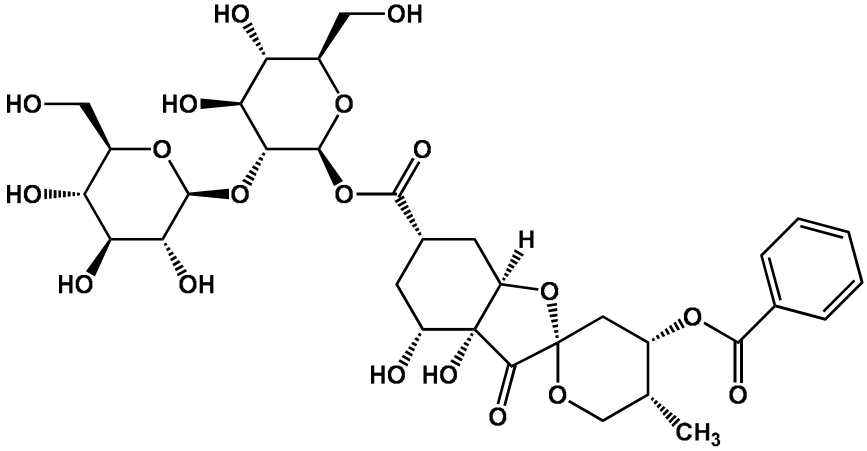


| | | | |
|---|---|---|---|
| C | 5.952244 | -2.751371 | -0.697872 |
| C | 4.773768 | -3.099797 | -1.597719 |
| O | 3.563632 | -2.830077 | -0.898154 |
| C | 3.418332 | -1.475716 | -0.577265 |
| C | 4.507550 | -1.051135 | 0.403427 |
| C | 5.867931 | -1.298572 | -0.250431 |
| O | 4.298900 | 0.277948 | 0.859859 |
| C | 4.679863 | 1.312113 | 0.019796 |
| O | 6.044114 | 1.583679 | 0.247160 |
| C | 6.561631 | 2.593951 | -0.605742 |
| C | 5.830694 | 3.902892 | -0.327926 |
| C | 4.331446 | 3.700781 | -0.483956 |
| C | 3.855383 | 2.538725 | 0.367438 |
| O | 2.171766 | -1.329791 | 0.060043 |
| C | 1.079243 | -1.299727 | -0.739613 |
| C | -0.192042 | -1.228895 | 0.061521 |
| O | 1.145617 | -1.335864 | -1.937619 |
| C | -0.391865 | -2.522047 | 0.860732 |

| | | | |
|---|---|---|---|
| C | -1.750839 | -2.508800 | 1.542279 |
| C | -1.911768 | -1.286637 | 2.469655 |
| C | -1.462192 | 0.034016 | 1.834740 |
| C | -0.193870 | -0.031895 | 1.013869 |
| O | -2.569037 | 0.391167 | 0.999115 |
| C | -3.766332 | 0.146783 | 1.701007 |
| C | -3.403045 | -1.018246 | 2.634439 |
| O | -4.129301 | -1.568583 | 3.409721 |
| C | -4.919043 | -0.118254 | 0.732066 |
| C | -5.911285 | 1.037127 | 0.787863 |
| C | -5.203022 | 2.384526 | 0.733795 |
| C | -4.258924 | 2.461971 | 1.952773 |
| O | -4.061449 | 1.194992 | 2.589374 |
| C | -4.465186 | 2.618940 | -0.582339 |
| O | -6.811538 | 0.979527 | -0.329347 |
| C | -7.819343 | 0.105174 | -0.270888 |
| O | -7.991108 | -0.651545 | 0.654395 |
| C | -8.703990 | 0.176746 | -1.465885 |
| C | -9.765147 | -0.719517 | -1.552504 |
| C | -10.617741 | -0.678638 | -2.643655 |
| C | -10.412440 | 0.259065 | -3.648772 |
| C | -9.353488 | 1.153949 | -3.563685 |
| C | -8.496606 | 1.114007 | -2.474485 |
| C | 8.041058 | 2.674847 | -0.322400 |
| O | 8.578882 | 1.383745 | -0.563820 |
| C | 4.762594 | -4.547798 | -2.025668 |
| O | 4.833967 | -5.372676 | -0.875594 |
| O | 2.489134 | 2.322366 | 0.089665 |
| O | 3.710239 | 4.916829 | -0.119443 |
| O | 6.304901 | 4.876871 | -1.231913 |
| O | 6.948620 | -1.066903 | 0.623331 |
| O | 7.142540 | -2.972208 | -1.421984 |
| H | -0.331171 | -3.395814 | 0.209416 |
| O | -1.251518 | -1.492866 | 3.687238 |
| H | 5.915553 | -3.391109 | 0.189976 |
| H | 4.821206 | -2.481585 | -2.503437 |
| H | 3.430273 | -0.869633 | -1.489401 |
| H | 4.420473 | -1.675098 | 1.294147 |
| H | 5.970420 | -0.669908 | -1.143991 |
| H | 4.526172 | 1.056398 | -1.038550 |
| H | 6.406725 | 2.306788 | -1.654388 |
| H | 6.032629 | 4.204831 | 0.707389 |
| H | 4.120089 | 3.470630 | -1.536052 |
| H | 4.012578 | 2.767295 | 1.427160 |
| H | -0.997950 | -1.126224 | -0.663206 |
| H | 0.388048 | -2.617327 | 1.618480 |
| O | -2.813285 | -2.464194 | 0.599601 |
| H | -1.855352 | -3.390984 | 2.181067 |
| H | -1.347620 | 0.773039 | 2.632611 |
| H | -0.094083 | 0.906841 | 0.465510 |
| H | 0.646886 | -0.116518 | 1.706077 |

| | | | |
|---|---|---|---|
| H | -5.417772 | -1.048240 | 0.998302 |
| H | -4.517300 | -0.224755 | -0.273677 |
| H | -6.501466 | 0.969471 | 1.700992 |
| H | -5.964750 | 3.156898 | 0.844088 |
| H | -4.674649 | 3.094103 | 2.733063 |
| H | -3.290648 | 2.867773 | 1.652918 |
| H | -4.026725 | 3.616693 | -0.583007 |
| H | -3.656526 | 1.901344 | -0.718845 |
| H | -5.144287 | 2.546960 | -1.430515 |
| H | -9.910368 | -1.441100 | -0.760758 |
| H | -11.441255 | -1.375728 | -2.711888 |
| H | -11.078753 | 0.291644 | -4.500107 |
| H | -9.195982 | 1.883690 | -4.345653 |
| H | -7.671639 | 1.807726 | -2.401561 |
| H | 8.490840 | 3.426331 | -0.972808 |
| H | 8.190339 | 2.968320 | 0.720911 |
| H | 9.525110 | 1.402576 | -0.397570 |
| H | 3.843339 | -4.734653 | -2.585889 |
| H | 5.617327 | -4.716562 | -2.684092 |
| H | 4.786936 | -6.290806 | -1.153828 |
| H | 2.183431 | 1.605935 | 0.657286 |
| H | 2.764188 | 4.838611 | -0.280401 |
| H | 5.752061 | 5.660455 | -1.135177 |
| H | 7.194864 | -0.133340 | 0.547843 |
| H | 7.863354 | -2.598530 | -0.901606 |
| H | -1.752521 | -2.133696 | 4.207419 |
| H | -2.854052 | -3.303475 | 0.131165 |

Equilibrium geometry of physalin D (**18**)

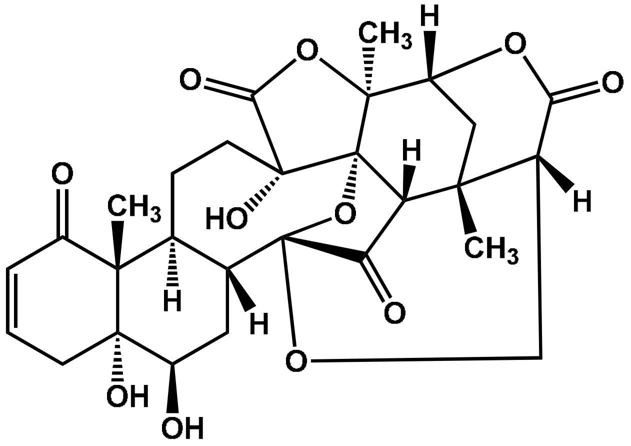


| | | | |
|---|---|---|---|
| C | -5.942961 | 0.451842 | -0.844269 |
| C | -5.339127 | -0.918064 | -0.866466 |
| C | -3.816483 | -0.873677 | -0.762625 |
| C | -3.361891 | 0.087839 | 0.379363 |
| C | -3.842978 | 1.457936 | -0.101098 |
| C | -5.265122 | 1.544774 | -0.490017 |
| C | -3.216241 | -2.271138 | -0.621847 |

| | | | |
|---|---|---|---|
| C | -1.699852 | -2.199479 | -0.548163 |
| C | -1.212279 | -1.299199 | 0.588303 |
| C | -1.824672 | 0.114374 | 0.501054 |
| O | -3.108913 | 2.425727 | -0.205480 |
| C | 0.307331 | -1.182450 | 0.515089 |
| C | 1.010103 | -0.636587 | 1.759106 |
| C | 2.111234 | 0.237686 | 1.238901 |
| C | 1.473145 | 0.755046 | -0.057834 |
| C | 0.592769 | 2.006121 | 0.206053 |
| C | -0.218495 | 2.048952 | 1.491462 |
| C | -1.394387 | 1.042271 | 1.670167 |
| O | 0.775126 | -0.948768 | 2.892455 |
| O | 0.704964 | -0.334051 | -0.541344 |
| O | 0.910469 | -2.458193 | 0.368455 |
| C | 3.417448 | -0.576228 | 1.037266 |
| C | 3.377784 | -1.831015 | 0.088419 |
| C | 2.058099 | -2.505648 | -0.448132 |
| C | 4.391922 | 0.433374 | 0.433784 |
| C | 3.947984 | 0.736883 | -0.975393 |
| C | 2.508288 | 1.284712 | -1.089467 |
| C | 1.671218 | 3.091202 | 0.135514 |
| O | 2.665883 | 2.668850 | -0.665479 |
| O | 1.667217 | 4.177573 | 0.633586 |
| C | 4.122298 | -1.648507 | -1.219826 |
| O | 4.133349 | -0.433468 | -1.787566 |
| C | 2.041556 | 1.224099 | -2.530865 |
| O | 4.584877 | -2.578460 | -1.826564 |
| C | 3.913177 | -1.028380 | 2.410796 |
| O | -3.270989 | -0.401122 | -1.999480 |
| O | -3.774689 | -2.886881 | 0.528807 |
| C | -4.044088 | -0.223055 | 1.728073 |
| O | -0.225352 | 2.278280 | -0.920153 |
| H | -6.973659 | 0.543801 | -1.165427 |
| H | -5.608622 | -1.422175 | -1.797909 |
| H | -5.762334 | -1.523057 | -0.062548 |
| H | -5.699553 | 2.534065 | -0.544118 |
| H | -3.507035 | -2.825950 | -1.519677 |
| H | -1.323072 | -1.820918 | -1.499921 |
| H | -1.304987 | -3.206901 | -0.420771 |
| H | -1.449819 | -1.759686 | 1.551760 |
| H | -1.473576 | 0.511478 | -0.443733 |
| H | 2.344312 | 1.026828 | 1.951589 |
| H | -0.624647 | 3.061818 | 1.522635 |
| H | 0.450319 | 1.987466 | 2.349074 |
| H | -2.242577 | 1.657419 | 1.945889 |
| H | -1.195903 | 0.422713 | 2.542867 |
| H | 3.916452 | -2.615315 | 0.615105 |
| H | 2.281338 | -3.561973 | -0.564387 |
| H | 1.815303 | -2.107128 | -1.431663 |
| H | 4.414784 | 1.346028 | 1.028946 |
| H | 5.406569 | 0.030938 | 0.406906 |

| | | | |
|---|---|---|---|
| H | 4.584476 | 1.489640 | -1.437268 |
| H | 2.823989 | 1.620019 | -3.177618 |
| H | 1.849995 | 0.190548 | -2.811914 |
| H | 1.131541 | 1.798149 | -2.662976 |
| H | 3.976079 | -0.181612 | 3.094802 |
| H | 3.248504 | -1.773846 | 2.846705 |
| H | 4.906120 | -1.467743 | 2.316418 |
| H | -3.860321 | 0.255550 | -2.385161 |
| H | -3.442960 | -3.788049 | 0.581918 |
| H | -3.609107 | -1.115058 | 2.170414 |
| H | -3.919211 | 0.604119 | 2.423748 |
| H | -5.111667 | -0.389961 | 1.616300 |
| H | -1.130928 | 2.460779 | -0.621948 |

Equilibrium geometry of procyanidin B2 (**19**)

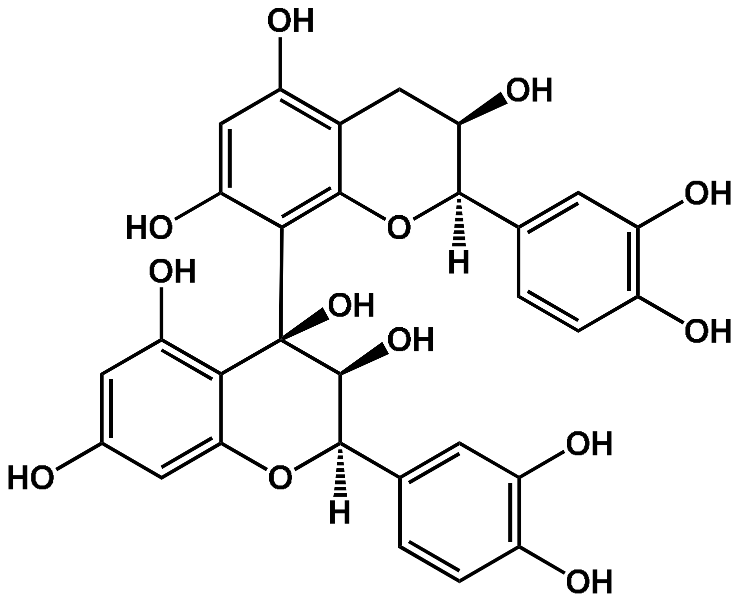


| | | | |
|---|---|---|---|
| C | 1.259532 | 3.094442 | 0.552592 |
| C | 0.671982 | 4.344187 | 0.683580 |
| C | -0.702691 | 4.458426 | 0.569712 |
| C | -1.511641 | 3.352246 | 0.304461 |
| C | -0.882536 | 2.113959 | 0.197607 |
| C | 0.499785 | 1.941846 | 0.343059 |
| C | -2.999874 | 3.495927 | 0.112038 |
| C | -3.596386 | 2.240910 | -0.514988 |
| C | -3.005355 | 1.028455 | 0.190522 |
| O | -1.595967 | 0.989463 | -0.094446 |
| O | -1.225256 | 5.702966 | 0.712051 |
| O | 2.610516 | 2.945403 | 0.631377 |
| C | -3.561560 | -0.316830 | -0.201850 |
| C | -4.664621 | -0.460068 | -1.037043 |
| C | -2.950588 | -1.451199 | 0.319261 |
| C | -3.426620 | -2.716775 | 0.010366 |
| C | -4.526032 | -2.853517 | -0.819066 |

| | | | |
|---|---|---|---|
| C | -5.148074 | -1.720726 | -1.341483 |
| O | -6.227025 | -1.847847 | -2.161537 |
| O | -5.076169 | -4.047936 | -1.186807 |
| C | 1.182313 | 0.595443 | 0.208047 |
| C | 1.245163 | 0.194185 | -1.265893 |
| C | 0.610074 | -0.552279 | 1.021478 |
| C | 0.608657 | -1.846424 | 0.501223 |
| O | 1.095986 | -2.151337 | -0.736666 |
| C | 1.891409 | -1.188330 | -1.418160 |
| C | 0.127912 | -0.407333 | 2.330680 |
| C | -0.369265 | -1.478254 | 3.058407 |
| C | -0.373962 | -2.742215 | 2.488779 |
| C | 0.119694 | -2.938993 | 1.211024 |
| C | 3.342558 | -1.230777 | -0.992926 |
| C | 3.747658 | -1.730180 | 0.238066 |
| C | 4.305128 | -0.730450 | -1.871486 |
| C | 5.640506 | -0.709609 | -1.517409 |
| C | 5.092168 | -1.715697 | 0.595322 |
| C | 6.036189 | -1.202697 | -0.272330 |
| O | 7.375996 | -1.144107 | -0.018248 |
| O | 6.564528 | -0.220947 | -2.385548 |
| H | -2.184964 | 5.683120 | 0.639349 |
| H | 3.026275 | 3.795059 | 0.810677 |
| H | 1.269191 | 5.227048 | 0.868382 |
| H | -3.227713 | 4.334006 | -0.550823 |
| H | -3.495087 | 3.688469 | 1.067881 |
| H | -3.117548 | 1.169487 | 1.271645 |
| H | -6.422599 | -2.786442 | -2.272321 |
| H | -4.584097 | -4.781535 | -0.804946 |
| H | 2.217131 | 0.739453 | 0.534391 |
| H | 0.147370 | -3.919550 | 0.755209 |
| H | 1.829022 | -1.464442 | -2.470584 |
| H | 4.023987 | -0.362847 | -2.850137 |
| H | 3.028849 | -2.145680 | 0.929273 |
| H | 5.408089 | -2.108637 | 1.553521 |
| H | 7.437979 | -0.290261 | -1.980098 |
| H | 7.575448 | -1.504009 | 0.851939 |
| H | -2.944783 | -3.598729 | 0.416090 |
| H | -2.092388 | -1.345830 | 0.968455 |
| H | -5.159031 | 0.393005 | -1.479868 |
| O | -3.349142 | 2.187549 | -1.907932 |
| H | -2.451817 | 1.864877 | -2.051935 |
| H | -4.677686 | 2.253240 | -0.388026 |
| O | 1.908005 | 1.163701 | -2.045327 |
| H | 2.727891 | 1.415863 | -1.603821 |
| H | -0.744603 | -1.321568 | 4.058981 |
| O | -0.876265 | -3.762372 | 3.236811 |
| H | -0.821051 | -4.589314 | 2.747113 |
| O | 0.130621 | 0.788464 | 2.978158 |
| H | 0.345097 | 1.504586 | 2.365299 |
| H | 0.231275 | 0.120580 | -1.662277 |

Equilibrium geometry of strychnine (**20**)

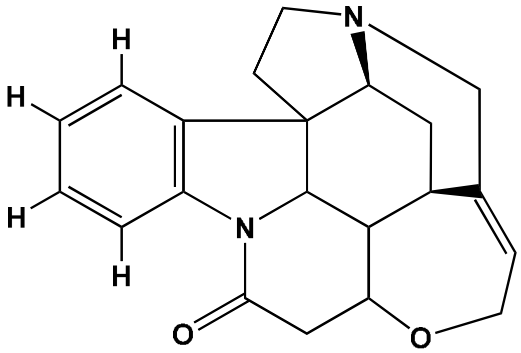


| | | | |
|---|---|---|---|
| O | -0.433610 | 6.038797 | -0.009786 |
| O | 4.162485 | 6.001808 | 0.994590 |
| N | 2.533037 | 4.897923 | 2.166640 |
| N | -1.182535 | 2.443235 | 3.222202 |
| C | 0.189887 | 2.081242 | 2.770606 |
| C | 0.232166 | 1.747976 | 1.287865 |
| C | -0.223225 | 2.975923 | 0.497402 |
| C | 0.881655 | 4.023668 | 0.660817 |
| C | 1.134892 | 4.409428 | 2.116061 |
| C | 3.338655 | 3.961534 | 2.841997 |
| C | 4.725223 | 3.944259 | 2.986813 |
| C | 5.287136 | 2.877452 | 3.693057 |
| C | 4.492928 | 1.868911 | 4.241361 |
| C | 3.103674 | 1.909748 | 4.098095 |
| C | 2.530735 | 2.960597 | 3.392051 |
| C | 1.083047 | 3.272531 | 3.166100 |
| C | 0.365431 | 3.755082 | 4.436022 |
| C | -1.095176 | 3.680074 | 4.019419 |
| C | -2.142351 | 2.566483 | 2.116735 |
| C | -1.585913 | 3.406475 | 1.002781 |
| C | -2.213573 | 4.486199 | 0.515471 |
| C | -1.580067 | 5.353261 | -0.528550 |
| C | 0.791555 | 5.323477 | -0.145598 |
| C | 1.859095 | 6.322318 | 0.343125 |
| C | 2.974146 | 5.733260 | 1.173485 |
| H | 0.528954 | 1.215432 | 3.347011 |
| H | -0.402002 | 0.885077 | 1.081933 |
| H | 1.251190 | 1.470764 | 1.009118 |
| H | -0.298540 | 2.728641 | -0.567707 |
| H | 1.800771 | 3.535868 | 0.308249 |
| H | 0.453314 | 5.217219 | 2.395731 |
| H | 5.334142 | 4.724921 | 2.562209 |
| H | 6.361323 | 2.838028 | 3.814471 |
| H | 4.954744 | 1.055862 | 4.784347 |
| H | 2.485285 | 1.135557 | 4.534824 |
| H | 0.572158 | 3.058375 | 5.250661 |
| H | 0.680491 | 4.753756 | 4.739421 |
| H | -1.777927 | 3.637917 | 4.867963 |

| | | | |
|---|---|---|---|
| H | -1.361424 | 4.558083 | 3.415356 |
| H | -3.061135 | 3.003377 | 2.509436 |
| H | -2.391182 | 1.566830 | 1.754289 |
| H | -3.182228 | 4.770032 | 0.910912 |
| H | -1.284477 | 4.765429 | -1.405329 |
| H | -2.266070 | 6.130165 | -0.858329 |
| H | 0.942686 | 5.089714 | -1.205493 |
| H | 2.305773 | 6.873105 | -0.479305 |
| H | 1.329257 | 7.038595 | 0.976108 |

Equilibrium geometry of strychnobaillonine (**21**)

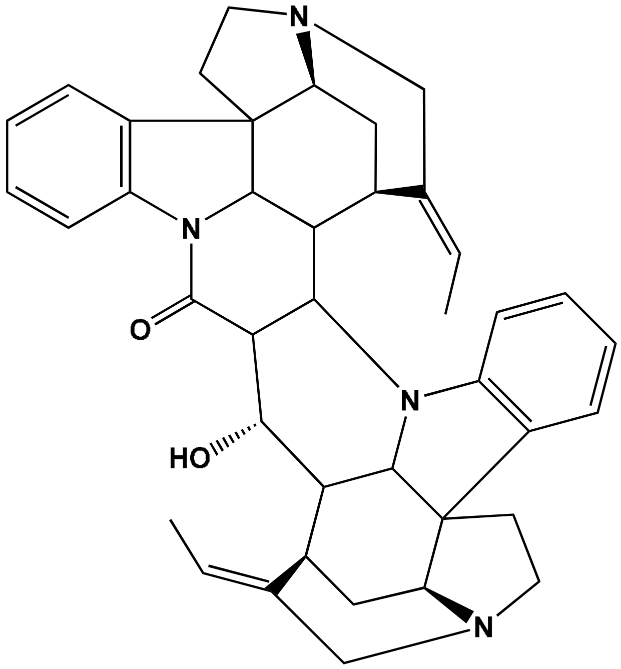


| | | | |
|---|---|---|---|
| O | 1.226924 | 2.680840 | 1.849197 |
| O | -1.111705 | 3.429238 | 0.772890 |
| N | -2.304949 | 1.611534 | 0.132811 |
| N | -4.644151 | -2.384122 | -0.240140 |
| N | 1.116773 | -0.748764 | 0.319312 |
| N | 5.200814 | -0.129625 | -1.459283 |
| C | -5.376803 | 3.624259 | 0.823201 |
| C | -6.304697 | 2.692637 | 0.378494 |
| C | -5.880006 | 1.459546 | -0.108275 |
| C | -4.526833 | 1.180701 | -0.152370 |
| C | -3.608298 | 2.128467 | 0.289286 |
| C | -4.010975 | 3.360334 | 0.786520 |
| C | -3.829325 | -0.089099 | -0.577905 |
| C | -2.363007 | 0.391194 | -0.706021 |
| C | -1.150415 | 2.281897 | 0.341456 |
| C | 0.093869 | 1.477757 | 0.017406 |
| C | -0.117571 | 0.017778 | 0.466097 |
| C | -1.227792 | -0.615691 | -0.401591 |
| C | -4.359219 | -0.691615 | -1.891234 |

| | | | |
|---|---|---|---|
| C | -5.286079 | -1.812756 | -1.420431 |
| C | -4.082899 | -1.233159 | 0.485834 |
| C | -2.817518 | -1.616475 | 1.238822 |
| C | -1.724052 | -1.936551 | 0.215392 |
| C | -0.054069 | -3.224850 | -2.034742 |
| C | -1.512349 | -3.464317 | -1.780683 |
| C | -2.247860 | -2.939588 | -0.803330 |
| C | -3.652928 | -3.424568 | -0.537388 |
| C | 1.371128 | 2.164501 | 0.538485 |
| C | 2.539206 | 1.184338 | 0.596433 |
| C | 2.276272 | -0.050177 | -0.261501 |
| C | 1.621093 | -1.604807 | 1.296787 |
| C | 3.421113 | -1.088763 | -0.171414 |
| C | 2.975578 | -1.871328 | 1.042013 |
| C | 0.987287 | -2.167614 | 2.396364 |
| C | 1.719472 | -3.029978 | 3.212910 |
| C | 3.047812 | -3.321723 | 2.948340 |
| C | 3.683190 | -2.730555 | 1.853140 |
| C | 3.923675 | 1.807155 | 0.371221 |
| C | 4.947173 | 0.767331 | 0.822881 |
| C | 4.827853 | -0.440883 | -0.078108 |
| C | 4.829801 | -1.357710 | -2.169933 |
| C | 3.554117 | -1.919776 | -1.472586 |
| C | 4.498725 | 1.037643 | -2.027307 |
| C | 4.230866 | 2.178632 | -1.061158 |
| C | 4.271078 | 3.430875 | -1.515081 |
| C | 3.945191 | 4.692538 | -0.776115 |
| H | -3.290344 | 4.084825 | 1.125464 |
| H | -2.511872 | -0.789588 | 1.883580 |
| H | -4.840528 | -0.868685 | 1.181464 |
| H | -6.256180 | -1.404449 | -1.126631 |
| H | -4.851376 | 0.055665 | -2.512391 |
| H | -3.608945 | -4.094528 | 0.327206 |
| H | -6.604148 | 0.731046 | -0.450069 |
| H | -7.360391 | 2.923887 | 0.411036 |
| H | -5.716182 | 4.578517 | 1.202646 |
| H | 0.168609 | 1.466858 | -1.077589 |
| H | -0.414970 | 0.021376 | 1.520569 |
| H | 0.438057 | -2.730997 | -1.197606 |
| H | 0.452575 | -4.173905 | -2.216108 |
| H | -2.242302 | 0.735720 | -1.737498 |
| H | -0.869272 | -2.399292 | 0.708932 |
| H | -4.014998 | -4.024112 | -1.371704 |
| H | -1.997096 | -4.173853 | -2.445923 |
| H | -3.530653 | -1.120635 | -2.458634 |
| H | -5.464276 | -2.577616 | -2.173676 |
| H | -3.021746 | -2.467774 | 1.888720 |
| H | -0.747099 | -0.837510 | -1.354152 |
| H | 0.102492 | -2.611288 | -2.926976 |
| H | 1.602147 | 2.981166 | -0.156270 |
| H | 0.498019 | 3.315017 | 1.813535 |

| | | | |
|---|---|---|---|
| H | 3.612591 | 4.518639 | 0.244429 |
| H | 4.811888 | 5.355830 | -0.746144 |
| H | 3.153132 | 5.236292 | -1.295061 |
| H | 4.560735 | 3.573583 | -2.553338 |
| H | 5.093683 | 1.412971 | -2.860662 |
| H | 3.540603 | 0.724756 | -2.463666 |
| H | 4.759548 | 0.480683 | 1.859035 |
| H | 5.961837 | 1.161606 | 0.757668 |
| H | 3.996525 | 2.689303 | 1.004626 |
| H | 2.542296 | 0.821535 | 1.630193 |
| H | 2.057075 | 0.213751 | -1.294702 |
| H | -0.044561 | -1.945668 | 2.633399 |
| H | 1.234965 | -3.473439 | 4.072613 |
| H | 3.595366 | -3.993753 | 3.593973 |
| H | 4.726534 | -2.942102 | 1.651459 |
| H | 3.676385 | -2.977873 | -1.245198 |
| H | 5.645577 | -2.074754 | -2.074225 |
| H | 2.661229 | -1.820954 | -2.090236 |
| H | 4.685803 | -1.156359 | -3.230042 |
| H | 5.517286 | -1.221272 | 0.258906 |

Equilibrium geometry of terrequinone A (**22**)

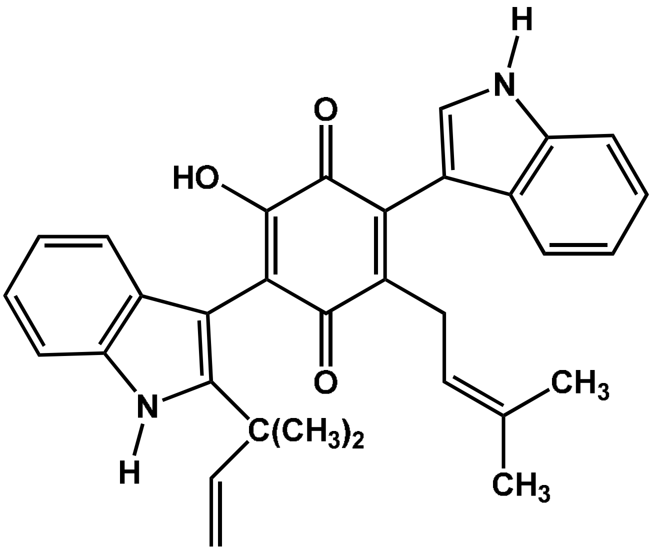


| | | | |
|---|---|---|---|
| O | -1.109757 | 0.842247 | -1.908403 |
| O | -1.679172 | -2.033836 | 1.747446 |
| O | 0.841069 | -2.659500 | 1.499915 |
| N | -4.856105 | 1.001519 | 0.279797 |
| N | 4.743419 | -2.486347 | -0.678133 |
| C | -2.847011 | 2.417670 | 0.752199 |
| C | -3.486392 | 1.102770 | 0.364293 |
| C | -2.938415 | -0.118597 | 0.052502 |
| C | -4.029026 | -1.014084 | -0.224397 |
| C | -1.523822 | -0.500073 | -0.029227 |
| C | -5.216920 | -0.273438 | -0.076796 |

| | | | |
|---|---|---|---|
| C | -2.841815 | 3.360743 | -0.455788 |
| C | -3.653816 | 3.067827 | 1.894578 |
| C | -1.459960 | 2.164472 | 1.297480 |
| C | -0.662144 | 0.070644 | -1.084003 |
| C | 0.796418 | -0.307860 | -1.160900 |
| C | 1.321022 | -1.203393 | -0.309952 |
| C | -0.988850 | -1.428294 | 0.779594 |
| C | -4.102455 | -2.362518 | -0.588493 |
| C | -6.474594 | -0.838712 | -0.278740 |
| C | 2.752104 | -1.549397 | -0.275116 |
| C | 0.447010 | -1.832424 | 0.700486 |
| C | 1.612418 | 0.412194 | -2.197019 |
| C | 3.737272 | -0.898413 | 0.541838 |
| C | -5.345836 | -2.928677 | -0.790175 |
| C | -6.520672 | -2.172690 | -0.635807 |
| C | 4.975089 | -1.511486 | 0.258457 |
| C | 3.409773 | -2.504631 | -0.994639 |
| C | -0.340709 | 2.748049 | 0.895844 |
| C | 2.099864 | 1.718402 | -1.625158 |
| C | 3.687680 | 0.169651 | 1.444649 |
| C | 6.162970 | -1.090339 | 0.856784 |
| C | 3.361174 | 2.083967 | -1.400495 |
| C | 4.860786 | 0.592719 | 2.033910 |
| C | 6.086970 | -0.034319 | 1.742160 |
| C | 3.667819 | 3.411466 | -0.766122 |
| C | 4.578969 | 1.271119 | -1.729670 |
| H | -5.502521 | 1.744359 | 0.484223 |
| H | -2.234051 | 2.953620 | -1.261866 |
| H | -2.449420 | 4.337128 | -0.169520 |
| H | -3.858744 | 3.500022 | -0.822681 |
| H | -4.656289 | 3.347459 | 1.569179 |
| H | -3.148299 | 3.977880 | 2.215379 |
| H | -3.738540 | 2.396073 | 2.748738 |
| H | -1.425967 | 1.466701 | 2.130159 |
| H | -3.198500 | -2.947208 | -0.706038 |
| H | -7.377229 | -0.254591 | -0.160993 |
| H | 2.438943 | -0.219238 | -2.506045 |
| H | 0.978781 | 0.605730 | -3.062028 |
| H | -5.422083 | -3.970411 | -1.070520 |
| H | -7.479732 | -2.644711 | -0.799905 |
| H | 3.026143 | -3.202957 | -1.719359 |
| H | 0.603323 | 2.538943 | 1.382195 |
| H | -0.321982 | 3.450536 | 0.072535 |
| H | 5.439348 | -3.092170 | -1.078299 |
| H | 1.309176 | 2.408300 | -1.346334 |
| H | -1.068460 | -2.641310 | 2.198707 |
| H | 2.746734 | 0.660703 | 1.659840 |
| H | 7.105875 | -1.567600 | 0.627146 |
| H | 4.844604 | 1.420394 | 2.729878 |
| H | 6.989081 | 0.321297 | 2.220972 |
| H | 4.199162 | 3.256367 | 0.176968 |

| | | | |
|---|---|---|---|
| H | 2.764145 | 3.985151 | -0.566544 |
| H | 4.325745 | 4.004298 | -1.405750 |
| H | 4.352483 | 0.274160 | -2.100443 |
| H | 5.178792 | 1.793464 | -2.479310 |
| H | 5.201761 | 1.166040 | -0.837259 |

Equilibrium geometry of viomycin (**23**)

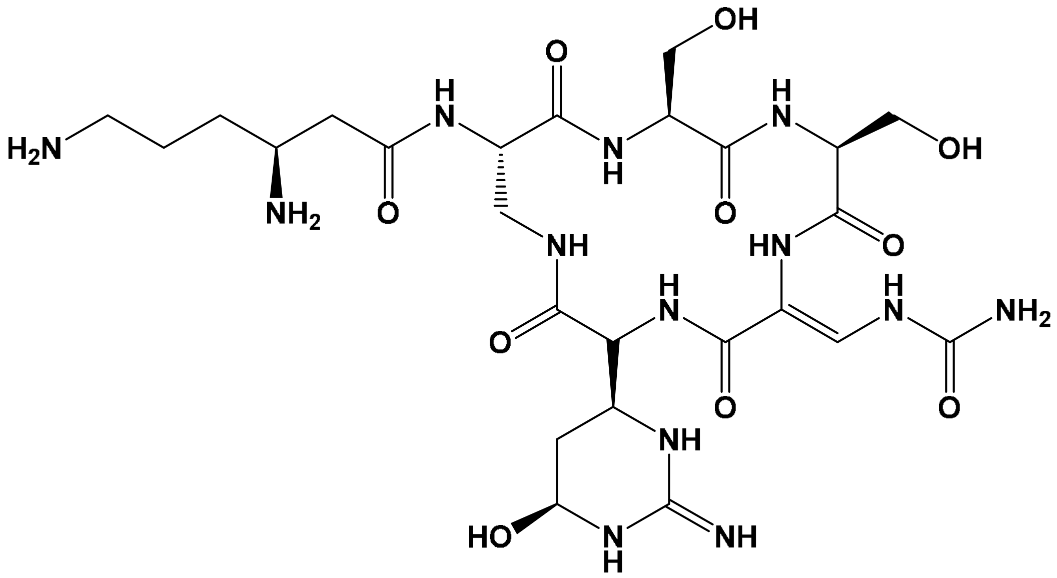


| | | | |
|---|---|---|---|
| O | 6.441226 | -3.610173 | 0.960422 |
| O | 2.531476 | -3.420217 | -0.033416 |
| O | -1.206663 | -1.198041 | -2.592761 |
| O | 2.619322 | -0.259604 | 1.613239 |
| O | -2.095408 | -2.561678 | 2.313012 |
| O | -5.270592 | 1.983455 | -2.829879 |
| O | -2.865375 | 2.681514 | -1.308139 |
| O | -0.212447 | 0.891800 | -0.464688 |
| O | 0.861491 | 4.626766 | -2.166782 |
| O | 2.547402 | 3.922850 | 4.240783 |
| N | 5.503255 | -0.380676 | -0.361011 |
| N | 2.797459 | 0.053045 | -0.617970 |
| N | 7.401281 | -1.769552 | -0.194214 |
| N | 0.979817 | -1.971947 | -0.803966 |
| N | -2.516316 | -2.851721 | 0.114892 |
| N | -2.967050 | -0.480421 | -1.366091 |
| N | 7.625750 | 0.532694 | -0.259465 |
| N | -6.486193 | -3.835167 | 1.581042 |
| N | -1.336432 | 1.772068 | -2.723610 |
| N | 1.409077 | 2.460447 | -0.393242 |
| N | -7.510256 | 1.431374 | 1.152819 |
| N | 1.705283 | 3.800649 | 2.124305 |
| N | 1.390854 | 5.685505 | 3.407879 |
| C | 4.677119 | -1.575126 | -0.256333 |
| C | 3.301864 | -1.279413 | -0.880073 |
| C | 5.344520 | -2.757806 | -0.953228 |

| | | | |
|---|---|---|---|
| C | 6.689321 | -3.016010 | -0.305545 |
| C | 2.239161 | -2.329963 | -0.505782 |
| C | -1.412233 | -2.034028 | -0.336379 |
| C | -0.154728 | -2.849757 | -0.612925 |
| C | 6.769822 | -0.557851 | -0.290779 |
| C | -5.305314 | -3.048711 | 1.223402 |
| C | -4.063969 | -3.807308 | 1.710607 |
| C | -5.391298 | -1.661897 | 1.849197 |
| C | -1.842607 | -1.203895 | -1.549895 |
| C | 2.511322 | 0.472042 | 0.636331 |
| C | -2.794579 | -3.037104 | 1.431781 |
| C | -3.394562 | 0.551992 | -2.296504 |
| C | -6.520152 | -0.810122 | 1.281793 |
| C | 2.024108 | 1.872958 | 0.741548 |
| C | -0.191562 | 2.516317 | -2.246596 |
| C | -2.515454 | 1.787096 | -2.063271 |
| C | -6.446811 | 0.629953 | 1.761805 |
| C | -4.859603 | 0.883772 | -2.048886 |
| C | 0.313268 | 1.882659 | -0.943075 |
| C | -0.426370 | 4.022786 | -2.124494 |
| C | 2.156695 | 2.529617 | 1.898437 |
| C | 1.921830 | 4.448160 | 3.334898 |
| H | 4.521047 | -1.826399 | 0.795372 |
| H | 3.410180 | -1.343200 | -1.966258 |
| H | 4.734737 | -3.653377 | -0.882387 |
| H | 5.501889 | -2.513753 | -2.006599 |
| H | 7.293659 | -3.695313 | -0.907966 |
| H | 2.901646 | 0.748643 | -1.337630 |
| H | 8.404998 | -1.787424 | -0.125002 |
| H | -1.190745 | -1.324182 | 0.463910 |
| H | 0.040356 | -3.484745 | 0.249309 |
| H | -0.309759 | -3.490730 | -1.484314 |
| H | 0.808840 | -1.046740 | -1.170149 |
| H | -5.222889 | -2.917216 | 0.135492 |
| H | -4.017044 | -4.782180 | 1.219934 |
| H | -4.130324 | -3.964533 | 2.785562 |
| H | -3.078634 | -3.330827 | -0.571034 |
| H | -5.501600 | -1.772874 | 2.931873 |
| H | -4.443245 | -1.139486 | 1.684686 |
| H | 7.283484 | -3.798919 | 1.385639 |
| H | -3.255777 | 0.181588 | -3.311585 |
| H | -7.492116 | -1.224646 | 1.564890 |
| H | -6.475524 | -0.819895 | 0.188297 |
| H | -3.352260 | -0.449895 | -0.432761 |
| H | 7.163439 | 1.386907 | -0.531455 |
| H | 8.509706 | 0.406706 | -0.730100 |
| H | 0.600309 | 2.392527 | -2.987863 |
| H | -6.438282 | -4.749389 | 1.144687 |
| H | -7.321571 | -3.393696 | 1.214741 |
| H | -6.472775 | 0.644293 | 2.857990 |
| H | -5.487360 | 1.059454 | 1.463133 |

| | | | |
|---|---|---|---|
| H | -5.013953 | 1.083467 | -0.983893 |
| H | -5.471579 | 0.028495 | -2.329912 |
| H | -1.115298 | 0.888601 | -3.165903 |
| H | 1.777205 | 3.297828 | -0.830311 |
| H | -0.940924 | 4.266252 | -1.196644 |
| H | -1.040920 | 4.345867 | -2.966389 |
| H | 2.661158 | 2.063230 | 2.731489 |
| H | -7.450371 | 2.390614 | 1.472701 |
| H | -8.413379 | 1.088285 | 1.461298 |
| H | -4.857543 | 2.769880 | -2.455071 |
| H | 1.167589 | 4.260711 | 1.404836 |
| H | 0.780464 | 5.554058 | -1.926269 |
| H | 0.866760 | 6.104959 | 2.660523 |
| H | 1.508526 | 6.199439 | 4.263021 |

**Table S1.** Calculated $^{1}$H NMR shielding constants, together with scaled and experimental chemical shifts – the GIAO-DFT(PBE0) calculations with the first-level basis sets.

| Mol. | pcS-1 | | pecS-1 | | pecS-1 mod | | $\delta_{exp}$[b] |
|---|---|---|---|---|---|---|---|
| | $\sigma$ | $\delta_{scaled}$[a] | $\sigma$ | $\delta_{scaled}$[a] | $\sigma$ | $\delta_{scaled}$[a] | |
| **1** | 28.71 | 2.88 | 28.3 | 3.39 | 28.4 | 3.36 | 4.38 |
| | 30.19 | 1.52 | 30.1 | 1.74 | 30.1 | 1.75 | 2.51 |
| | 30.60 | 1.15 | 30.5 | 1.36 | 30.6 | 1.34 | 2.31 |
| | 29.09 | 2.53 | 28.9 | 2.89 | 28.9 | 2.88 | 3.53 |
| | 28.92 | 2.69 | 28.9 | 2.85 | 29.0 | 2.84 | 3.21 |
| | 27.69 | 3.82 | 27.8 | 3.91 | 27.8 | 3.96 | 4.03 |
| | 29.15 | 2.48 | 29.0 | 2.71 | 29.1 | 2.72 | 3.18 |
| | 28.63 | 2.96 | 28.7 | 3.02 | 28.8 | 3.01 | 3.34 |
| | 28.66 | 2.93 | 28.9 | 2.87 | 28.9 | 2.88 | 3.24 |
| | 28.98 | 2.64 | 29.1 | 2.63 | 29.1 | 2.66 | 2.80 |
| | 28.67 | 2.92 | 28.6 | 3.11 | 28.7 | 3.09 | 3.00 |
| | 28.31 | 3.25 | 28.3 | 3.37 | 28.4 | 3.37 | 3.27 |
| | 25.71 | 5.64 | 25.9 | 5.71 | 25.9 | 5.69 | 5.42 |
| | 23.79 | 7.42 | 24.2 | 7.31 | 24.2 | 7.30 | 7.45 |
| | 24.38 | 6.87 | 24.7 | 6.76 | 24.8 | 6.76 | 7.25 |
| | 29.33 | 2.31 | 29.6 | 2.15 | 29.7 | 2.17 | 0.99 |
| | 29.71 | 1.96 | 30.2 | 1.67 | 30.2 | 1.69 | 0.99 |
| | 29.75 | 1.93 | 30.2 | 1.65 | 30.2 | 1.67 | 0.99 |
| | 24.26 | 6.98 | 24.5 | 6.94 | 24.6 | 6.93 | 7.01 |
| | 24.29 | 6.96 | 24.6 | 6.92 | 24.6 | 6.90 | 7.05 |
| | 23.37 | 7.80 | 23.7 | 7.72 | 23.7 | 7.71 | 7.81 |
| | 22.81 | 8.32 | 23.1 | 8.26 | 23.2 | 8.22 | 8.11 |
| | 23.60 | 7.59 | 24.1 | 7.34 | 24.1 | 7.37 | 7.47 |
| | 23.81 | 7.39 | 24.3 | 7.21 | 24.3 | 7.20 | 7.40 |
| | 24.06 | 7.17 | 24.5 | 7.02 | 24.5 | 7.02 | 7.10 |
| **2** | 24.33 | 6.92 | 24.53 | 6.96 | 24.57 | 6.94 | 6.94 |

| | | | | | | | |
|---|---|---|---|---|---|---|---|
| | 23.84 | 7.37 | 24.22 | 7.25 | 24.23 | 7.26 | 7.62 |
| | 24.00 | 7.23 | 24.28 | 7.20 | 24.31 | 7.18 | 7.09 |
| | 24.12 | 7.11 | 24.40 | 7.08 | 24.44 | 7.06 | 7.19 |
| | 23.68 | 7.52 | 23.81 | 7.64 | 23.81 | 7.65 | 7.36 |
| | 24.91 | 6.38 | 24.94 | 6.58 | 24.95 | 6.58 | 6.94 |
| | 23.97 | 7.25 | 24.15 | 7.32 | 24.19 | 7.29 | 7.36 |
| | 24.31 | 6.94 | 24.60 | 6.90 | 24.64 | 6.87 | 7.19 |
| | 24.12 | 7.11 | 24.40 | 7.09 | 24.43 | 7.07 | 7.09 |
| | 23.98 | 7.24 | 24.36 | 7.12 | 24.38 | 7.12 | 7.62 |
| | 23.40 | 7.78 | 23.70 | 7.75 | 23.72 | 7.73 | 7.73 |
| | 24.30 | 6.95 | 24.64 | 6.86 | 24.66 | 6.86 | 6.89 |
| | 23.70 | 7.50 | 24.00 | 7.46 | 24.03 | 7.44 | 7.51 |
| | 24.34 | 6.91 | 24.71 | 6.79 | 24.70 | 6.81 | 6.91 |
| **3** | 27.33 | 4.16 | 26.94 | 4.69 | 27.00 | 4.67 | 4.18 |
| | 28.38 | 3.19 | 28.25 | 3.46 | 28.32 | 3.44 | 2.88 |
| | 29.55 | 2.11 | 29.52 | 2.26 | 29.63 | 2.21 | 2.20 |
| | 28.77 | 2.83 | 28.80 | 2.94 | 28.81 | 2.98 | 2.60 |
| | 28.88 | 2.72 | 28.91 | 2.84 | 28.97 | 2.83 | 2.35 |
| | 29.87 | 1.82 | 29.90 | 1.91 | 29.97 | 1.89 | 1.81 |
| | 30.17 | 1.54 | 30.23 | 1.59 | 30.30 | 1.59 | 1.51 |
| | 30.05 | 1.65 | 30.03 | 1.78 | 30.09 | 1.79 | 2.34 |
| | 25.38 | 5.95 | 25.43 | 6.12 | 25.43 | 6.14 | 6.40 |
| | 28.82 | 2.79 | 28.92 | 2.83 | 28.93 | 2.87 | 2.80 |
| | 29.94 | 1.76 | 29.98 | 1.83 | 30.06 | 1.81 | 1.97 |
| | 29.48 | 2.18 | 29.73 | 2.07 | 29.79 | 2.06 | 1.94 |
| | 29.84 | 1.84 | 29.90 | 1.91 | 29.96 | 1.90 | 1.57 |
| | 28.57 | 3.02 | 28.67 | 3.07 | 28.72 | 3.06 | 3.24 |
| | 27.63 | 3.88 | 27.65 | 4.03 | 27.66 | 4.06 | 3.80 |
| | 29.40 | 2.25 | 29.47 | 2.31 | 29.51 | 2.32 | 2.44 |
| | 29.16 | 2.47 | 29.34 | 2.43 | 29.40 | 2.43 | 2.54 |
| | 29.24 | 2.40 | 29.20 | 2.57 | 29.22 | 2.60 | 2.07 |
| | 29.24 | 2.40 | 29.39 | 2.39 | 29.45 | 2.38 | 2.31 |
| | 29.33 | 2.31 | 29.43 | 2.35 | 29.45 | 2.38 | 1.70 |
| | 30.22 | 1.49 | 30.27 | 1.56 | 30.36 | 1.53 | 1.61 |
| | 30.24 | 1.47 | 30.46 | 1.38 | 30.51 | 1.39 | 1.47 |
| | 30.31 | 1.41 | 30.46 | 1.38 | 30.51 | 1.39 | 1.47 |
| | 30.54 | 1.20 | 30.46 | 1.38 | 30.53 | 1.37 | 1.38 |
| | 30.13 | 1.58 | 30.29 | 1.54 | 30.36 | 1.53 | 1.38 |
| | 25.75 | 5.61 | 25.91 | 5.66 | 25.97 | 5.64 | 5.58 |
| | 29.22 | 2.41 | 29.46 | 2.32 | 29.50 | 2.33 | 2.56 |
| | 29.33 | 2.32 | 29.45 | 2.33 | 29.51 | 2.33 | 2.58 |
| | 29.90 | 1.79 | 30.06 | 1.75 | 30.14 | 1.74 | 1.86 |
| | 28.56 | 3.03 | 28.55 | 3.17 | 28.59 | 3.18 | 2.43 |
| | 26.02 | 5.37 | 26.17 | 5.42 | 26.24 | 5.38 | 5.47 |
| | 22.77 | 8.36 | 23.12 | 8.29 | 23.16 | 8.25 | 8.33 |
| | 23.27 | 7.89 | 23.58 | 7.85 | 23.61 | 7.84 | 8.02 |
| | 23.98 | 7.24 | 24.00 | 7.46 | 24.03 | 7.45 | 6.24 |
| | 22.84 | 8.29 | 23.28 | 8.14 | 23.30 | 8.13 | 8.23 |
| | 23.66 | 7.54 | 24.02 | 7.44 | 24.04 | 7.44 | 7.64 |
| | 25.40 | 5.93 | 25.61 | 5.95 | 25.64 | 5.94 | 5.75 |

| | | | | | | | |
|---|---|---|---|---|---|---|---|
| | 23.84 | 7.37 | 24.19 | 7.28 | 24.21 | 7.27 | 7.25 |
| | 23.58 | 7.61 | 23.89 | 7.57 | 23.92 | 7.55 | 7.56 |
| **4** | 27.62 | 3.89 | 27.55 | 4.12 | 27.62 | 4.09 | 4.11 |
| | 27.16 | 4.32 | 26.74 | 4.88 | 26.84 | 4.81 | 4.26 |
| | 28.61 | 2.97 | 28.39 | 3.33 | 28.48 | 3.29 | 2.76 |
| | 28.56 | 3.02 | 28.23 | 3.48 | 28.31 | 3.45 | 2.95 |
| | 24.02 | 7.20 | 24.12 | 7.34 | 24.15 | 7.34 | 7.56 |
| | 24.18 | 7.05 | 24.48 | 7.01 | 24.50 | 7.00 | 6.91 |
| | 23.77 | 7.44 | 24.06 | 7.40 | 24.10 | 7.38 | 7.19 |
| | 24.12 | 7.12 | 24.53 | 6.96 | 24.54 | 6.97 | 6.96 |
| | 28.54 | 3.04 | 28.39 | 3.33 | 28.48 | 3.29 | 2.79 |
| | 28.71 | 2.88 | 28.59 | 3.14 | 28.67 | 3.11 | 2.51 |
| | 30.68 | 1.07 | 30.53 | 1.32 | 30.65 | 1.26 | 0.72 |
| | 27.21 | 4.26 | 27.49 | 4.18 | 27.53 | 4.17 | 3.83 |
| | 27.37 | 4.12 | 27.58 | 4.09 | 27.60 | 4.11 | 4.05 |
| | 29.97 | 1.72 | 29.79 | 2.01 | 29.90 | 1.96 | 1.56 |
| | 29.77 | 1.91 | 29.77 | 2.03 | 29.82 | 2.03 | 1.62 |
| | 27.33 | 4.15 | 27.10 | 4.54 | 27.13 | 4.55 | 4.49 |
| | 27.35 | 4.14 | 27.84 | 3.85 | 27.85 | 3.88 | 3.78 |
| | 27.36 | 4.12 | 27.80 | 3.88 | 27.81 | 3.91 | 3.78 |
| | 27.40 | 4.09 | 27.81 | 3.88 | 27.82 | 3.90 | 3.78 |
| | 29.16 | 2.47 | 29.12 | 2.64 | 29.17 | 2.64 | 2.37 |
| | 28.59 | 3.00 | 28.76 | 2.98 | 28.78 | 3.01 | 2.88 |
| | 29.20 | 2.43 | 29.26 | 2.51 | 29.30 | 2.52 | 2.31 |
| | 28.35 | 3.22 | 28.54 | 3.19 | 28.58 | 3.20 | 3.09 |
| | 30.27 | 1.45 | 30.31 | 1.52 | 30.38 | 1.51 | 1.20 |
| | 28.44 | 3.13 | 28.57 | 3.16 | 28.62 | 3.16 | 2.74 |
| | 24.41 | 6.84 | 24.64 | 6.86 | 24.63 | 6.88 | 6.65 |
| | 25.13 | 6.18 | 25.44 | 6.11 | 25.44 | 6.13 | 5.85 |
| | 24.91 | 6.38 | 25.27 | 6.26 | 25.26 | 6.29 | 6.22 |
| | 29.45 | 2.21 | 29.67 | 2.12 | 29.73 | 2.12 | 1.95 |
| | 29.48 | 2.18 | 29.69 | 2.10 | 29.72 | 2.13 | 1.99 |
| | 27.90 | 3.63 | 27.97 | 3.73 | 28.04 | 3.70 | 3.54 |
| | 29.42 | 2.23 | 29.09 | 2.67 | 29.14 | 2.67 | 2.28 |
| | 30.63 | 1.12 | 30.60 | 1.25 | 30.69 | 1.22 | 1.07 |
| | 29.54 | 2.12 | 29.49 | 2.29 | 29.54 | 2.30 | 1.90 |
| | 27.01 | 4.45 | 27.37 | 4.29 | 27.37 | 4.32 | 4.01 |
| | 27.15 | 4.32 | 27.58 | 4.09 | 27.59 | 4.12 | 4.23 |
| | 28.61 | 2.98 | 28.74 | 3.00 | 28.77 | 3.02 | 2.07 |
| | 29.56 | 2.10 | 29.61 | 2.18 | 29.70 | 2.15 | 2.59 |
| | 28.95 | 2.66 | 28.50 | 3.23 | 28.59 | 3.19 | 2.80 |
| | 30.21 | 1.50 | 30.16 | 1.66 | 30.23 | 1.65 | 1.49 |
| | 29.20 | 2.43 | 29.06 | 2.70 | 29.21 | 2.61 | 2.15 |
| | 27.67 | 3.85 | 27.61 | 4.06 | 27.68 | 4.03 | 3.71 |
| | 27.63 | 3.87 | 27.58 | 4.09 | 27.68 | 4.04 | 3.83 |
| | 27.57 | 3.94 | 27.65 | 4.02 | 27.70 | 4.02 | 3.82 |
| | 27.42 | 4.07 | 27.44 | 4.22 | 27.50 | 4.20 | 3.92 |
| | 23.64 | 7.55 | 23.69 | 7.75 | 23.76 | 7.70 | 7.68 |
| | 23.74 | 7.46 | 23.92 | 7.53 | 23.96 | 7.51 | 7.26 |
| | 23.95 | 7.27 | 24.19 | 7.28 | 24.23 | 7.26 | 7.11 |

| | | | | | | | |
|---|---|---|---|---|---|---|---|
| | 23.70 | 7.50 | 23.95 | 7.51 | 23.99 | 7.48 | 7.33 |
| | 24.17 | 7.06 | 24.55 | 6.94 | 24.54 | 6.96 | 7.02 |
| | 28.05 | 3.50 | 27.97 | 3.73 | 28.06 | 3.68 | 3.46 |
| | 28.74 | 2.86 | 28.66 | 3.07 | 28.72 | 3.06 | 2.36 |
| | 29.05 | 2.57 | 29.01 | 2.75 | 29.07 | 2.73 | 2.66 |
| | 27.49 | 4.01 | 27.84 | 3.84 | 27.86 | 3.87 | 3.75 |
| | 27.43 | 4.06 | 27.72 | 3.96 | 27.76 | 3.96 | 3.75 |
| | 29.99 | 1.71 | 29.99 | 1.83 | 30.05 | 1.82 | 1.43 |
| | 29.84 | 1.84 | 29.86 | 1.94 | 29.93 | 1.93 | 1.60 |
| | 27.21 | 4.26 | 26.88 | 4.75 | 26.91 | 4.75 | 4.29 |
| | 27.64 | 3.87 | 28.06 | 3.64 | 28.08 | 3.66 | 3.69 |
| | 27.47 | 4.03 | 27.89 | 3.80 | 27.90 | 3.83 | 3.69 |
| | 27.50 | 4.00 | 27.98 | 3.72 | 27.98 | 3.75 | 3.69 |
| | 29.12 | 2.50 | 29.13 | 2.63 | 29.17 | 2.64 | 2.37 |
| | 28.57 | 3.02 | 28.77 | 2.98 | 28.77 | 3.01 | 2.88 |
| | 29.18 | 2.45 | 29.27 | 2.50 | 29.30 | 2.52 | 2.31 |
| | 28.34 | 3.23 | 28.56 | 3.17 | 28.58 | 3.19 | 3.09 |
| | 30.29 | 1.43 | 30.36 | 1.48 | 30.41 | 1.48 | 1.21 |
| | 28.47 | 3.10 | 28.64 | 3.09 | 28.68 | 3.10 | 2.75 |
| | 24.10 | 7.13 | 24.33 | 7.14 | 24.31 | 7.18 | 6.83 |
| | 24.74 | 6.54 | 25.00 | 6.52 | 25.00 | 6.54 | 6.35 |
| | 24.66 | 6.61 | 25.00 | 6.52 | 25.05 | 6.49 | 6.41 |
| | 29.42 | 2.23 | 29.67 | 2.12 | 29.72 | 2.13 | 1.95 |
| | 29.42 | 2.23 | 29.65 | 2.14 | 29.67 | 2.18 | 1.99 |
| | 27.88 | 3.65 | 27.94 | 3.75 | 28.00 | 3.73 | 3.51 |
| | 29.57 | 2.09 | 29.35 | 2.43 | 29.37 | 2.45 | 1.99 |
| | 30.51 | 1.22 | 30.55 | 1.29 | 30.62 | 1.29 | 0.97 |
| | 29.50 | 2.15 | 29.56 | 2.23 | 29.58 | 2.26 | 2.00 |
| | 27.21 | 4.26 | 27.60 | 4.08 | 27.61 | 4.10 | 3.94 |
| | 27.09 | 4.38 | 27.42 | 4.24 | 27.42 | 4.28 | 4.16 |
| | 29.58 | 2.09 | 29.69 | 2.11 | 29.75 | 2.10 | 1.74 |
| | 28.69 | 2.91 | 28.93 | 2.82 | 28.94 | 2.85 | 2.56 |
| | 28.89 | 2.72 | 28.45 | 3.28 | 28.52 | 3.25 | 2.79 |
| | 29.75 | 1.93 | 29.86 | 1.94 | 29.92 | 1.95 | 1.67 |
| | 29.18 | 2.46 | 29.24 | 2.52 | 29.33 | 2.49 | 2.20 |
| | 26.74 | 4.70 | 26.79 | 4.84 | 26.80 | 4.86 | 4.73 |
| | 28.26 | 3.30 | 28.25 | 3.46 | 28.30 | 3.46 | 3.20 |
| **5** | 29.35 | 2.30 | 29.27 | 2.50 | 29.37 | 2.46 | 2.48 |
| | 29.52 | 2.14 | 29.60 | 2.19 | 29.68 | 2.16 | 2.23 |
| | 28.16 | 3.39 | 28.07 | 3.63 | 28.16 | 3.58 | 3.41 |
| | 28.84 | 2.77 | 28.87 | 2.88 | 28.96 | 2.84 | 2.75 |
| | 30.09 | 1.61 | 30.15 | 1.67 | 30.19 | 1.69 | 1.67 |
| | 29.96 | 1.74 | 30.19 | 1.64 | 30.28 | 1.61 | 1.55 |
| | 27.52 | 3.98 | 27.46 | 4.20 | 27.52 | 4.19 | 4.23 |
| | 29.20 | 2.43 | 29.05 | 2.71 | 29.14 | 2.67 | 2.57 |
| | 30.00 | 1.69 | 29.71 | 2.08 | 29.79 | 2.06 | 1.95 |
| | 29.96 | 1.73 | 30.11 | 1.71 | 30.17 | 1.71 | 1.76 |
| | 30.20 | 1.51 | 30.33 | 1.51 | 30.42 | 1.48 | 1.56 |
| | 30.50 | 1.24 | 30.74 | 1.12 | 30.81 | 1.11 | 1.15 |
| | 30.25 | 1.47 | 30.59 | 1.26 | 30.66 | 1.25 | 1.15 |

| | | | | | | | |
|---|---|---|---|---|---|---|---|
| | 30.30 | 1.42 | 30.66 | 1.19 | 30.74 | 1.17 | 1.15 |
| | 29.96 | 1.73 | 30.04 | 1.78 | 30.11 | 1.76 | 1.84 |
| | 30.18 | 1.53 | 30.29 | 1.54 | 30.37 | 1.52 | 1.64 |
| | 30.43 | 1.30 | 30.78 | 1.08 | 30.83 | 1.09 | 1.25 |
| | 30.45 | 1.28 | 30.70 | 1.16 | 30.75 | 1.17 | 1.25 |
| | 30.11 | 1.60 | 30.49 | 1.35 | 30.52 | 1.38 | 1.25 |
| | 29.57 | 2.09 | 29.47 | 2.32 | 29.52 | 2.32 | 2.29 |
| | 26.90 | 4.55 | 26.84 | 4.79 | 26.93 | 4.73 | 4.74 |
| | 29.72 | 1.95 | 29.90 | 1.90 | 29.99 | 1.87 | 1.89 |
| | 29.18 | 2.45 | 29.40 | 2.38 | 29.46 | 2.37 | 1.89 |
| | 29.70 | 1.97 | 29.99 | 1.83 | 30.06 | 1.81 | 1.89 |
| | 30.02 | 1.68 | 30.18 | 1.64 | 30.24 | 1.65 | 1.63 |
| | 30.02 | 1.68 | 30.18 | 1.64 | 30.26 | 1.62 | 1.78 |
| | 29.94 | 1.75 | 29.70 | 2.10 | 29.79 | 2.07 | 1.98 |
| | 29.99 | 1.71 | 30.07 | 1.74 | 30.16 | 1.71 | 1.64 |
| | 29.92 | 1.77 | 30.08 | 1.74 | 30.15 | 1.73 | 1.84 |
| | 30.02 | 1.68 | 30.49 | 1.36 | 30.52 | 1.38 | 1.22 |
| | 30.33 | 1.40 | 30.61 | 1.24 | 30.67 | 1.25 | 1.22 |
| | 30.51 | 1.22 | 30.89 | 0.97 | 30.93 | 0.99 | 1.22 |
| | 30.86 | 0.91 | 31.13 | 0.75 | 31.20 | 0.75 | 1.16 |
| | 30.03 | 1.67 | 30.34 | 1.50 | 30.40 | 1.49 | 1.16 |
| | 30.31 | 1.41 | 30.69 | 1.17 | 30.75 | 1.17 | 1.16 |
| | 29.51 | 2.14 | 29.48 | 2.31 | 29.54 | 2.30 | 2.31 |
| | 30.87 | 0.90 | 31.18 | 0.70 | 31.25 | 0.70 | 1.18 |
| | 30.01 | 1.69 | 30.37 | 1.47 | 30.44 | 1.46 | 1.18 |
| | 30.28 | 1.44 | 30.69 | 1.16 | 30.75 | 1.17 | 1.18 |
| **6** | 23.35 | 7.82 | 23.72 | 7.73 | 23.73 | 7.72 | 7.82 |
| | 23.63 | 7.57 | 24.00 | 7.46 | 24.03 | 7.44 | 7.55 |
| | 22.92 | 8.21 | 23.29 | 8.13 | 23.30 | 8.13 | 8.27 |
| | 23.51 | 7.67 | 24.01 | 7.45 | 24.01 | 7.47 | 7.67 |
| | 26.56 | 4.86 | 26.52 | 5.09 | 26.54 | 5.10 | 4.56 |
| | 25.05 | 6.26 | 25.19 | 6.34 | 25.20 | 6.35 | 7.58 |
| | 24.19 | 7.04 | 24.44 | 7.04 | 24.46 | 7.04 | 7.69 |
| | 23.89 | 7.32 | 24.16 | 7.31 | 24.20 | 7.29 | 7.66 |
| | 23.80 | 7.41 | 23.92 | 7.53 | 23.93 | 7.53 | 7.96 |
| | 28.32 | 3.25 | 28.41 | 3.31 | 28.43 | 3.34 | 2.58 |
| | 28.32 | 3.24 | 28.44 | 3.28 | 28.54 | 3.23 | 2.71 |
| | 26.74 | 4.70 | 26.68 | 4.94 | 26.72 | 4.93 | 5.49 |
| | 24.60 | 6.67 | 24.88 | 6.63 | 24.86 | 6.67 | 7.23 |
| | 24.67 | 6.60 | 24.98 | 6.54 | 24.99 | 6.54 | 7.09 |
| | 24.20 | 7.04 | 24.44 | 7.05 | 24.47 | 7.04 | 7.27 |
| | 24.42 | 6.84 | 24.67 | 6.83 | 24.69 | 6.83 | 7.48 |
| | 28.13 | 3.42 | 28.10 | 3.60 | 28.19 | 3.56 | 4.25 |
| | 29.62 | 2.05 | 29.58 | 2.20 | 29.63 | 2.21 | 1.73 |
| | 30.10 | 1.60 | 30.17 | 1.66 | 30.26 | 1.63 | 1.51 |
| | 30.52 | 1.22 | 30.22 | 1.61 | 30.25 | 1.63 | 1.84 |
| | 30.27 | 1.45 | 30.57 | 1.28 | 30.61 | 1.30 | 0.95 |
| | 30.27 | 1.45 | 30.59 | 1.26 | 30.65 | 1.26 | 0.95 |
| | 30.62 | 1.13 | 30.88 | 0.99 | 30.96 | 0.97 | 0.95 |
| | 30.01 | 1.69 | 30.33 | 1.50 | 30.36 | 1.53 | 0.90 |

| | | | | | | | |
|---|---|---|---|---|---|---|---|
| | 30.92 | 0.85 | 31.11 | 0.77 | 31.21 | 0.74 | 0.90 |
| | 30.23 | 1.48 | 30.52 | 1.32 | 30.56 | 1.34 | 0.90 |
| **7** | 25.48 | 5.86 | 25.42 | 6.12 | 25.46 | 6.11 | 6.17 |
| | 27.47 | 4.02 | 27.39 | 4.27 | 27.46 | 4.24 | 4.49 |
| | 27.96 | 3.58 | 28.04 | 3.66 | 28.10 | 3.64 | 3.09 |
| | 23.78 | 7.42 | 23.86 | 7.59 | 23.86 | 7.60 | 7.34 |
| | 23.74 | 7.46 | 24.00 | 7.46 | 24.00 | 7.47 | 7.22 |
| | 27.17 | 4.30 | 27.47 | 4.20 | 27.51 | 4.19 | 4.72 |
| | 26.82 | 4.62 | 27.06 | 4.58 | 27.11 | 4.57 | 4.72 |
| | 24.44 | 6.82 | 24.86 | 6.65 | 24.87 | 6.66 | 6.75 |
| | 28.99 | 2.63 | 29.37 | 2.41 | 29.42 | 2.41 | 2.93 |
| | 27.63 | 3.88 | 27.96 | 3.73 | 27.95 | 3.79 | 2.93 |
| | 29.42 | 2.23 | 29.74 | 2.06 | 29.77 | 2.08 | 2.93 |
| | 27.79 | 3.73 | 27.78 | 3.90 | 27.82 | 3.91 | 2.62 |
| | 28.18 | 3.37 | 28.11 | 3.59 | 28.15 | 3.60 | 2.62 |
| | 24.47 | 6.79 | 24.81 | 6.70 | 24.84 | 6.69 | 6.90 |
| | 23.53 | 7.66 | 23.74 | 7.71 | 23.73 | 7.72 | 7.29 |
| | 24.00 | 7.22 | 24.31 | 7.16 | 24.35 | 7.14 | 7.23 |
| | 22.93 | 8.21 | 23.36 | 8.06 | 23.36 | 8.07 | 7.64 |
| | 23.91 | 7.30 | 24.25 | 7.22 | 24.29 | 7.20 | 7.19 |
| | 24.01 | 7.22 | 24.38 | 7.10 | 24.40 | 7.10 | 7.19 |
| | 28.90 | 2.71 | 29.23 | 2.54 | 29.27 | 2.55 | 3.14 |
| | 27.49 | 4.01 | 27.81 | 3.87 | 27.78 | 3.94 | 3.14 |
| | 28.99 | 2.63 | 29.33 | 2.44 | 29.35 | 2.47 | 3.14 |
| | 27.53 | 3.97 | 27.90 | 3.79 | 27.95 | 3.78 | 4.72 |
| | 27.04 | 4.42 | 27.32 | 4.33 | 27.37 | 4.32 | 4.72 |
| | 29.24 | 2.39 | 29.67 | 2.13 | 29.71 | 2.14 | 3.14 |
| | 27.85 | 3.67 | 28.19 | 3.52 | 28.18 | 3.57 | 3.14 |
| | 29.41 | 2.24 | 29.78 | 2.02 | 29.82 | 2.03 | 3.14 |
| **8** | 30.32 | 1.40 | 30.56 | 1.29 | 30.57 | 1.33 | 0.82 |
| | 30.74 | 1.01 | 30.85 | 1.01 | 30.89 | 1.04 | 0.82 |
| | 31.18 | 0.61 | 31.40 | 0.50 | 31.45 | 0.51 | 0.82 |
| | 30.55 | 1.19 | 30.86 | 1.01 | 30.95 | 0.98 | 1.13 |
| | 30.66 | 1.09 | 30.99 | 0.88 | 31.05 | 0.89 | 1.13 |
| | 30.52 | 1.22 | 30.83 | 1.04 | 30.89 | 1.03 | 1.13 |
| | 25.77 | 5.59 | 26.07 | 5.51 | 26.10 | 5.51 | 5.42 |
| | 30.01 | 1.68 | 30.22 | 1.61 | 30.28 | 1.61 | 1.05 |
| | 30.71 | 1.04 | 30.96 | 0.91 | 31.02 | 0.92 | 1.05 |
| | 30.81 | 0.95 | 30.98 | 0.89 | 31.05 | 0.89 | 1.05 |
| | 30.65 | 1.10 | 30.98 | 0.90 | 31.05 | 0.88 | 0.68 |
| | 30.61 | 1.13 | 30.94 | 0.93 | 31.00 | 0.94 | 0.68 |
| | 31.08 | 0.70 | 31.35 | 0.55 | 31.41 | 0.55 | 0.68 |
| **9** | 30.67 | 1.08 | 30.43 | 1.41 | 30.60 | 1.31 | 1.44 |
| | 30.28 | 1.44 | 30.04 | 1.78 | 30.18 | 1.70 | 1.69 |
| | 30.04 | 1.66 | 30.27 | 1.56 | 30.34 | 1.55 | 1.69 |
| | 29.97 | 1.72 | 30.09 | 1.73 | 30.16 | 1.72 | 1.59 |
| | 30.25 | 1.47 | 30.25 | 1.58 | 30.34 | 1.55 | 1.52 |
| | 30.75 | 1.01 | 30.73 | 1.12 | 30.85 | 1.07 | 1.52 |
| | 28.05 | 3.50 | 28.07 | 3.63 | 28.16 | 3.59 | 3.64 |
| | 30.19 | 1.52 | 30.37 | 1.47 | 30.44 | 1.46 | 2.21 |

| | | | | | | | |
|---|---|---|---|---|---|---|---|
| | 29.24 | 2.39 | 29.45 | 2.33 | 29.47 | 2.36 | 1.52 |
| | 29.83 | 1.85 | 29.70 | 2.09 | 29.77 | 2.08 | 1.77 |
| | 29.96 | 1.73 | 30.07 | 1.75 | 30.12 | 1.75 | 1.52 |
| | 30.23 | 1.48 | 30.36 | 1.47 | 30.46 | 1.44 | 1.52 |
| | 30.44 | 1.29 | 30.48 | 1.36 | 30.58 | 1.33 | 1.98 |
| | 29.00 | 2.61 | 29.12 | 2.64 | 29.23 | 2.59 | 1.28 |
| | 29.93 | 1.76 | 30.04 | 1.78 | 30.13 | 1.74 | 1.31 |
| | 29.68 | 1.99 | 29.76 | 2.04 | 29.77 | 2.08 | 1.77 |
| | 30.48 | 1.26 | 30.57 | 1.28 | 30.69 | 1.23 | 1.48 |
| | 30.44 | 1.29 | 30.53 | 1.31 | 30.57 | 1.34 | 1.49 |
| | 30.20 | 1.51 | 30.27 | 1.56 | 30.37 | 1.52 | 1.81 |
| | 29.76 | 1.92 | 29.88 | 1.92 | 30.00 | 1.87 | 1.28 |
| | 28.52 | 3.06 | 28.61 | 3.12 | 28.69 | 3.09 | 3.10 |
| | 29.92 | 1.77 | 30.16 | 1.67 | 30.25 | 1.63 | 1.74 |
| | 27.34 | 4.15 | 27.52 | 4.15 | 27.59 | 4.12 | 4.43 |
| | 27.73 | 3.79 | 27.64 | 4.04 | 27.73 | 3.99 | 4.26 |
| | 30.51 | 1.23 | 30.76 | 1.10 | 30.82 | 1.10 | 1.21 |
| | 30.08 | 1.62 | 30.56 | 1.29 | 30.60 | 1.31 | 1.21 |
| | 30.49 | 1.24 | 30.94 | 0.93 | 31.00 | 0.93 | 1.21 |
| | 30.79 | 0.97 | 31.03 | 0.84 | 31.10 | 0.84 | 0.88 |
| | 30.79 | 0.97 | 30.97 | 0.90 | 31.05 | 0.89 | 0.88 |
| | 30.69 | 1.06 | 30.91 | 0.96 | 30.97 | 0.96 | 0.88 |
| | 30.75 | 1.01 | 30.98 | 0.89 | 31.05 | 0.89 | 0.95 |
| | 30.41 | 1.32 | 30.58 | 1.27 | 30.69 | 1.23 | 0.95 |
| | 30.69 | 1.06 | 30.83 | 1.04 | 30.92 | 1.01 | 0.95 |
| | 30.43 | 1.30 | 30.60 | 1.25 | 30.66 | 1.25 | 1.13 |
| | 31.06 | 0.72 | 31.17 | 0.71 | 31.27 | 0.68 | 1.13 |
| | 30.22 | 1.50 | 30.39 | 1.45 | 30.42 | 1.48 | 1.13 |
| | 30.89 | 0.88 | 31.19 | 0.70 | 31.26 | 0.69 | 1.22 |
| | 30.70 | 1.05 | 30.94 | 0.93 | 31.01 | 0.92 | 1.22 |
| | 29.47 | 2.18 | 29.70 | 2.10 | 29.75 | 2.10 | 1.22 |
| | 29.63 | 2.03 | 29.98 | 1.84 | 30.03 | 1.84 | 0.99 |
| | 30.94 | 0.83 | 31.18 | 0.70 | 31.26 | 0.69 | 0.99 |
| | 30.77 | 0.99 | 31.06 | 0.82 | 31.14 | 0.80 | 0.99 |
| | 30.46 | 1.27 | 30.76 | 1.10 | 30.82 | 1.10 | 0.96 |
| | 31.01 | 0.77 | 31.27 | 0.62 | 31.35 | 0.61 | 0.96 |
| | 30.29 | 1.43 | 30.66 | 1.19 | 30.71 | 1.20 | 0.96 |
| **10** | 26.09 | 5.29 | 25.90 | 5.67 | 25.91 | 5.69 | 5.63 |
| | 28.19 | 3.36 | 28.27 | 3.44 | 28.29 | 3.47 | 3.43 |
| | 28.49 | 3.09 | 28.66 | 3.08 | 28.68 | 3.10 | 3.17 |
| | 29.33 | 2.31 | 29.18 | 2.59 | 29.22 | 2.60 | 2.23 |
| | 30.14 | 1.57 | 30.25 | 1.58 | 30.35 | 1.54 | 1.32 |
| | 30.32 | 1.40 | 30.29 | 1.54 | 30.34 | 1.55 | 1.10 |
| | 26.48 | 4.94 | 26.19 | 5.40 | 26.19 | 5.43 | 5.25 |
| | 26.70 | 4.73 | 26.69 | 4.93 | 26.72 | 4.93 | 4.64 |
| | 29.30 | 2.34 | 29.25 | 2.52 | 29.29 | 2.53 | 2.51 |
| | 29.48 | 2.18 | 29.44 | 2.34 | 29.53 | 2.31 | 1.90 |
| | 29.03 | 2.59 | 29.15 | 2.61 | 29.20 | 2.62 | 2.37 |
| | 27.68 | 3.83 | 27.47 | 4.19 | 27.49 | 4.21 | 4.77 |
| | 26.32 | 5.09 | 26.42 | 5.19 | 26.48 | 5.15 | 4.79 |

| | | | | | | | |
|---|---|---|---|---|---|---|---|
| | 28.96 | 2.65 | 29.11 | 2.66 | 29.17 | 2.64 | 2.67 |
| | 29.32 | 2.32 | 29.45 | 2.33 | 29.49 | 2.34 | 2.61 |
| | 30.64 | 1.11 | 31.00 | 0.87 | 31.06 | 0.88 | 0.83 |
| | 30.52 | 1.22 | 30.90 | 0.97 | 30.95 | 0.98 | 0.83 |
| | 30.60 | 1.14 | 31.00 | 0.87 | 31.05 | 0.89 | 0.83 |
| | 28.95 | 2.67 | 29.16 | 2.60 | 29.16 | 2.65 | 2.97 |
| | 29.68 | 1.99 | 29.86 | 1.94 | 29.90 | 1.96 | 2.97 |
| | 29.16 | 2.47 | 29.40 | 2.38 | 29.43 | 2.40 | 2.97 |
| | 30.63 | 1.12 | 30.93 | 0.94 | 30.99 | 0.94 | 1.06 |
| | 30.14 | 1.56 | 30.54 | 1.31 | 30.60 | 1.31 | 1.06 |
| | 30.59 | 1.15 | 30.97 | 0.90 | 31.04 | 0.89 | 1.06 |
| | 30.72 | 1.03 | 31.04 | 0.83 | 31.10 | 0.84 | 1.15 |
| | 30.24 | 1.47 | 30.47 | 1.37 | 30.53 | 1.37 | 1.15 |
| | 31.15 | 0.64 | 31.40 | 0.50 | 31.48 | 0.48 | 1.15 |
| | 31.11 | 0.67 | 31.47 | 0.43 | 31.55 | 0.42 | 1.04 |
| | 31.63 | 0.19 | 31.97 | -0.04 | 32.04 | -0.04 | 1.04 |
| | 32.88 | -0.95 | 33.05 | -1.05 | 33.10 | -1.02 | 1.04 |
| | 29.97 | 1.73 | 30.13 | 1.69 | 30.19 | 1.69 | 1.59 |
| | 30.05 | 1.65 | 30.09 | 1.73 | 30.16 | 1.71 | 1.59 |
| | 30.89 | 0.88 | 30.97 | 0.90 | 31.05 | 0.88 | 1.59 |
| | 23.81 | 7.40 | 24.04 | 7.43 | 24.07 | 7.41 | 7.61 |
| | 23.87 | 7.34 | 24.28 | 7.19 | 24.30 | 7.19 | 7.35 |
| | 24.01 | 7.21 | 24.30 | 7.18 | 24.33 | 7.17 | 6.90 |
| | 24.00 | 7.22 | 24.25 | 7.22 | 24.24 | 7.25 | 6.90 |
| | 24.27 | 6.97 | 24.54 | 6.95 | 24.56 | 6.94 | 7.11 |
| | 24.12 | 7.11 | 24.40 | 7.08 | 24.44 | 7.06 | 7.18 |
| | 24.44 | 6.82 | 24.86 | 6.65 | 24.89 | 6.64 | 6.69 |
| | 24.39 | 6.87 | 24.81 | 6.70 | 24.78 | 6.74 | 6.69 |
| **11** | 27.53 | 3.97 | 27.63 | 4.05 | 27.68 | 4.03 | 3.78 |
| | 29.14 | 2.49 | 29.17 | 2.59 | 29.24 | 2.58 | 2.97 |
| | 28.69 | 2.91 | 28.83 | 2.91 | 28.88 | 2.91 | 2.77 |
| | 24.78 | 6.51 | 25.32 | 6.21 | 25.28 | 6.27 | 6.45 |
| | 23.84 | 7.37 | 23.96 | 7.49 | 24.01 | 7.46 | 7.35 |
| | 24.36 | 6.89 | 24.61 | 6.88 | 24.62 | 6.89 | 6.97 |
| | 24.43 | 6.83 | 24.72 | 6.78 | 24.72 | 6.80 | 6.84 |
| | 24.02 | 7.20 | 24.18 | 7.29 | 24.24 | 7.24 | 7.30 |
| | 24.72 | 6.55 | 24.93 | 6.59 | 24.94 | 6.59 | 6.73 |
| | 24.68 | 6.60 | 24.88 | 6.63 | 24.90 | 6.63 | 6.84 |
| | 24.95 | 6.34 | 25.57 | 5.99 | 25.54 | 6.03 | 6.44 |
| | 30.00 | 1.70 | 29.90 | 1.91 | 30.00 | 1.87 | 2.10 |
| | 29.47 | 2.18 | 29.34 | 2.43 | 29.40 | 2.43 | 2.75 |
| | 28.77 | 2.83 | 28.67 | 3.06 | 28.71 | 3.07 | 3.65 |
| | 27.65 | 3.86 | 27.68 | 4.00 | 27.67 | 4.05 | 4.76 |
| | 30.88 | 0.89 | 31.11 | 0.77 | 31.17 | 0.78 | 1.45 |
| | 30.54 | 1.20 | 30.84 | 1.03 | 30.85 | 1.07 | 1.45 |
| | 30.38 | 1.35 | 30.76 | 1.10 | 30.82 | 1.10 | 1.45 |
| | 28.59 | 3.00 | 28.96 | 2.79 | 29.01 | 2.79 | 2.44 |
| | 29.54 | 2.12 | 29.64 | 2.15 | 29.68 | 2.17 | 2.44 |
| | 29.59 | 2.08 | 29.82 | 1.98 | 29.88 | 1.98 | 2.44 |
| | 28.96 | 2.65 | 28.96 | 2.79 | 28.98 | 2.82 | 3.28 |

| | | | | | | | |
|---|---|---|---|---|---|---|---|
| | 28.06 | 3.49 | 28.37 | 3.34 | 28.42 | 3.34 | 3.28 |
| | 29.26 | 2.38 | 29.45 | 2.33 | 29.46 | 2.37 | 3.28 |
| | 29.49 | 2.16 | 29.69 | 2.10 | 29.73 | 2.12 | 2.38 |
| | 28.86 | 2.74 | 29.15 | 2.61 | 29.21 | 2.61 | 2.38 |
| | 28.83 | 2.78 | 29.10 | 2.66 | 29.15 | 2.66 | 2.38 |
| | 27.40 | 4.09 | 27.79 | 3.89 | 27.80 | 3.92 | 4.09 |
| | 27.47 | 4.02 | 27.86 | 3.83 | 27.87 | 3.86 | 4.09 |
| | 26.96 | 4.50 | 27.38 | 4.28 | 27.40 | 4.30 | 4.09 |
| | 27.11 | 4.35 | 27.54 | 4.12 | 27.60 | 4.11 | 4.08 |
| | 27.65 | 3.86 | 27.99 | 3.71 | 28.04 | 3.70 | 4.08 |
| | 27.74 | 3.78 | 28.06 | 3.64 | 28.11 | 3.63 | 4.08 |
| | 28.92 | 2.69 | 29.19 | 2.58 | 29.25 | 2.56 | 2.33 |
| | 29.61 | 2.06 | 29.79 | 2.02 | 29.83 | 2.02 | 2.33 |
| | 28.91 | 2.70 | 29.16 | 2.60 | 29.23 | 2.59 | 2.33 |
| | 30.30 | 1.42 | 30.50 | 1.34 | 30.57 | 1.34 | 1.62 |
| | 30.36 | 1.37 | 30.49 | 1.35 | 30.50 | 1.40 | 1.62 |
| | 30.08 | 1.63 | 30.38 | 1.46 | 30.40 | 1.50 | 1.62 |
| | 31.01 | 0.77 | 31.42 | 0.47 | 31.48 | 0.48 | 1.10 |
| | 30.41 | 1.31 | 30.79 | 1.07 | 30.87 | 1.06 | 1.10 |
| | 30.68 | 1.07 | 31.02 | 0.85 | 31.08 | 0.86 | 1.10 |
| **12** | 29.58 | 2.09 | 29.25 | 2.52 | 29.25 | 2.56 | 2.56 |
| | 28.32 | 3.24 | 28.42 | 3.30 | 28.45 | 3.31 | 4.04 |
| | 29.64 | 2.03 | 29.46 | 2.32 | 29.48 | 2.35 | 2.23 |
| | 29.31 | 2.33 | 29.39 | 2.39 | 29.45 | 2.38 | 2.47 |
| | 28.65 | 2.94 | 28.85 | 2.90 | 28.90 | 2.90 | 2.85 |
| | 30.34 | 1.39 | 30.56 | 1.29 | 30.60 | 1.31 | 1.07 |
| | 30.92 | 0.85 | 31.09 | 0.79 | 31.14 | 0.80 | 1.07 |
| | 31.13 | 0.66 | 31.24 | 0.64 | 31.31 | 0.65 | 1.07 |
| | 26.80 | 4.64 | 27.12 | 4.52 | 27.13 | 4.55 | 4.76 |
| | 27.30 | 4.18 | 27.34 | 4.31 | 27.38 | 4.32 | 4.46 |
| | 30.63 | 1.11 | 30.93 | 0.94 | 30.99 | 0.95 | 1.17 |
| | 30.24 | 1.48 | 30.42 | 1.42 | 30.46 | 1.44 | 1.17 |
| | 30.48 | 1.26 | 30.81 | 1.06 | 30.86 | 1.06 | 1.17 |
| | 28.80 | 2.80 | 29.01 | 2.75 | 29.07 | 2.73 | 2.98 |
| | 29.06 | 2.56 | 29.27 | 2.50 | 29.30 | 2.52 | 2.68 |
| | 29.87 | 1.82 | 30.21 | 1.62 | 30.23 | 1.66 | 1.18 |
| | 30.39 | 1.33 | 30.73 | 1.13 | 30.80 | 1.12 | 1.18 |
| | 30.87 | 0.90 | 31.10 | 0.78 | 31.16 | 0.79 | 1.18 |
| | 30.52 | 1.22 | 30.98 | 0.89 | 31.03 | 0.91 | 1.29 |
| | 30.25 | 1.47 | 30.58 | 1.27 | 30.63 | 1.28 | 1.29 |
| | 30.07 | 1.63 | 30.45 | 1.39 | 30.51 | 1.39 | 1.29 |
| | 24.77 | 6.51 | 25.17 | 6.36 | 25.16 | 6.39 | 6.34 |
| | 24.04 | 7.18 | 24.38 | 7.10 | 24.41 | 7.09 | 7.41 |
| | 23.97 | 7.25 | 24.44 | 7.05 | 24.42 | 7.08 | 7.40 |
| **13** | 27.86 | 3.67 | 27.59 | 4.08 | 27.65 | 4.06 | 4.11 |
| | 28.95 | 2.66 | 28.68 | 3.06 | 28.74 | 3.04 | 1.84 |
| | 28.28 | 3.28 | 28.26 | 3.45 | 28.28 | 3.47 | 3.14 |
| | 28.51 | 3.07 | 28.26 | 3.45 | 28.28 | 3.47 | 2.98 |
| | 29.68 | 2.00 | 29.80 | 2.00 | 29.87 | 1.98 | 2.16 |
| | 28.83 | 2.77 | 28.94 | 2.81 | 28.98 | 2.82 | 2.16 |

| | | | | | | | |
|---|---|---|---|---|---|---|---|
| | 29.55 | 2.11 | 29.49 | 2.30 | 29.52 | 2.31 | 2.48 |
| | 29.89 | 1.80 | 29.89 | 1.92 | 29.95 | 1.92 | 2.48 |
| | 28.21 | 3.34 | 28.39 | 3.33 | 28.41 | 3.35 | 3.12 |
| | 28.69 | 2.90 | 28.82 | 2.92 | 28.86 | 2.93 | 3.12 |
| | 27.72 | 3.80 | 27.85 | 3.84 | 27.85 | 3.87 | 3.55 |
| | 28.89 | 2.72 | 28.94 | 2.81 | 28.97 | 2.83 | 3.55 |
| | 24.11 | 7.12 | 24.38 | 7.10 | 24.40 | 7.10 | 7.14 |
| | 24.53 | 6.73 | 24.69 | 6.81 | 24.70 | 6.81 | 6.20 |
| | 25.99 | 5.39 | 26.13 | 5.46 | 26.16 | 5.45 | 5.35 |
| | 24.46 | 6.80 | 24.82 | 6.69 | 24.84 | 6.68 | 6.66 |
| | 24.27 | 6.97 | 24.52 | 6.97 | 24.56 | 6.95 | 7.12 |
| | 29.47 | 2.18 | 29.59 | 2.20 | 29.64 | 2.20 | 1.83 |
| | 29.79 | 1.89 | 30.12 | 1.70 | 30.16 | 1.71 | 1.83 |
| | 29.75 | 1.93 | 30.08 | 1.74 | 30.12 | 1.76 | 1.83 |
| | 26.30 | 5.10 | 26.28 | 5.31 | 26.30 | 5.32 | 5.30 |
| | 29.55 | 2.11 | 29.49 | 2.30 | 29.52 | 2.31 | 2.48 |
| | 29.89 | 1.80 | 29.89 | 1.92 | 29.95 | 1.92 | 2.48 |
| | 28.21 | 3.34 | 28.39 | 3.33 | 28.41 | 3.35 | 2.90 |
| | 28.69 | 2.90 | 28.82 | 2.92 | 28.86 | 2.93 | 2.90 |
| | 27.72 | 3.80 | 27.85 | 3.84 | 27.85 | 3.87 | 3.13 |
| | 28.89 | 2.72 | 28.94 | 2.81 | 28.97 | 2.83 | 3.13 |
| **14** | 29.25 | 2.39 | 28.81 | 2.94 | 28.89 | 2.90 | 2.99 |
| | 28.72 | 2.88 | 28.36 | 3.36 | 28.43 | 3.33 | 3.36 |
| | 27.94 | 3.59 | 27.87 | 3.82 | 27.89 | 3.83 | 3.50 |
| | 28.92 | 2.69 | 28.95 | 2.80 | 29.01 | 2.79 | 2.72 |
| | 29.60 | 2.06 | 29.62 | 2.17 | 29.69 | 2.16 | 2.04 |
| | 25.30 | 6.03 | 25.48 | 6.07 | 25.46 | 6.10 | 6.38 |
| | 24.08 | 7.15 | 24.38 | 7.10 | 24.38 | 7.12 | 7.14 |
| | 29.49 | 2.16 | 29.87 | 1.94 | 29.92 | 1.95 | 1.78 |
| | 29.64 | 2.03 | 29.93 | 1.88 | 29.97 | 1.89 | 1.78 |
| | 29.61 | 2.06 | 29.97 | 1.84 | 30.02 | 1.85 | 1.78 |
| | 24.34 | 6.91 | 24.75 | 6.76 | 24.76 | 6.76 | 6.94 |
| | 24.83 | 6.46 | 25.30 | 6.23 | 25.32 | 6.24 | 6.42 |
| | 23.91 | 7.30 | 24.13 | 7.34 | 24.14 | 7.34 | 7.24 |
| | 24.26 | 6.99 | 24.74 | 6.76 | 24.74 | 6.78 | 6.98 |
| | 24.84 | 6.45 | 25.34 | 6.20 | 25.31 | 6.25 | 6.51 |
| | 25.37 | 5.96 | 25.87 | 5.70 | 25.80 | 5.79 | 6.23 |
| | 24.90 | 6.39 | 25.34 | 6.20 | 25.35 | 6.21 | 6.46 |
| | 24.25 | 6.99 | 24.69 | 6.81 | 24.70 | 6.81 | 7.05 |
| | 23.65 | 7.54 | 24.14 | 7.33 | 24.18 | 7.30 | 7.41 |
| | 24.40 | 6.86 | 24.87 | 6.64 | 24.86 | 6.67 | 6.97 |
| | 24.37 | 6.89 | 24.78 | 6.72 | 24.81 | 6.72 | 6.81 |
| **15** | 29.12 | 2.51 | 28.95 | 2.80 | 28.96 | 2.84 | 2.23 |
| | 26.81 | 4.63 | 26.82 | 4.81 | 26.85 | 4.81 | 5.41 |
| | 27.97 | 3.57 | 27.55 | 4.12 | 27.55 | 4.16 | 3.58 |
| | 26.55 | 4.87 | 26.27 | 5.32 | 26.28 | 5.34 | 5.68 |
| | 30.25 | 1.47 | 30.64 | 1.21 | 30.71 | 1.20 | 1.02 |
| | 30.68 | 1.07 | 30.98 | 0.90 | 31.06 | 0.88 | 1.02 |
| | 30.26 | 1.46 | 30.58 | 1.27 | 30.63 | 1.28 | 1.02 |
| | 23.50 | 7.68 | 23.82 | 7.63 | 23.81 | 7.65 | 8.01 |

| | | | | | | | |
|---|---|---|---|---|---|---|---|
| | 23.68 | 7.51 | 24.07 | 7.40 | 24.08 | 7.39 | 7.42 |
| | 23.73 | 7.47 | 24.02 | 7.44 | 24.04 | 7.43 | 7.54 |
| | 24.07 | 7.16 | 24.42 | 7.07 | 24.43 | 7.07 | 7.42 |
| | 24.05 | 7.18 | 24.28 | 7.19 | 24.27 | 7.22 | 8.01 |
| | 25.82 | 5.54 | 25.71 | 5.85 | 25.73 | 5.85 | 6.36 |
| | 29.47 | 2.18 | 29.81 | 2.00 | 29.86 | 2.00 | 1.68 |
| | 29.81 | 1.87 | 30.08 | 1.74 | 30.12 | 1.76 | 1.68 |
| | 29.95 | 1.74 | 30.26 | 1.57 | 30.29 | 1.60 | 1.68 |
| | 25.38 | 5.95 | 25.50 | 6.05 | 25.49 | 6.08 | 5.39 |
| | 29.88 | 1.81 | 29.74 | 2.06 | 29.78 | 2.07 | 1.99 |
| | 29.22 | 2.41 | 29.20 | 2.57 | 29.24 | 2.58 | 2.37 |
| | 27.33 | 4.15 | 27.11 | 4.53 | 27.17 | 4.51 | 5.48 |
| | 29.85 | 1.84 | 30.22 | 1.60 | 30.25 | 1.63 | 0.97 |
| | 30.71 | 1.04 | 30.92 | 0.95 | 31.02 | 0.92 | 0.97 |
| | 30.93 | 0.84 | 31.14 | 0.74 | 31.21 | 0.73 | 0.97 |
| | 30.58 | 1.16 | 30.79 | 1.07 | 30.85 | 1.08 | 1.02 |
| | 30.74 | 1.02 | 30.89 | 0.98 | 30.97 | 0.96 | 1.02 |
| | 30.47 | 1.26 | 30.62 | 1.23 | 30.68 | 1.23 | 1.02 |
| | 26.88 | 4.57 | 26.66 | 4.96 | 26.70 | 4.95 | 5.39 |
| | 24.49 | 6.77 | 24.65 | 6.85 | 24.67 | 6.84 | 6.75 |
| | 28.51 | 3.07 | 28.85 | 2.89 | 28.90 | 2.89 | 1.73 |
| | 29.60 | 2.07 | 29.85 | 1.96 | 29.90 | 1.96 | 1.73 |
| | 29.60 | 2.07 | 29.93 | 1.88 | 29.99 | 1.88 | 1.73 |
| | 29.60 | 2.07 | 29.83 | 1.98 | 29.88 | 1.98 | 2.05 |
| | 28.79 | 2.81 | 29.17 | 2.60 | 29.18 | 2.63 | 2.05 |
| | 29.76 | 1.92 | 30.06 | 1.76 | 30.08 | 1.80 | 2.05 |
| | 29.60 | 2.07 | 29.90 | 1.91 | 29.95 | 1.92 | 2.27 |
| | 28.94 | 2.67 | 29.37 | 2.40 | 29.40 | 2.43 | 2.27 |
| | 29.12 | 2.50 | 29.57 | 2.22 | 29.59 | 2.25 | 2.27 |
| | 28.91 | 2.70 | 29.31 | 2.46 | 29.32 | 2.51 | 2.18 |
| | 29.60 | 2.07 | 29.91 | 1.90 | 29.90 | 1.96 | 2.18 |
| | 29.81 | 1.87 | 30.00 | 1.81 | 30.09 | 1.78 | 2.18 |
| | 29.43 | 2.22 | 29.68 | 2.12 | 29.73 | 2.12 | 2.02 |
| | 28.88 | 2.73 | 29.24 | 2.53 | 29.27 | 2.55 | 2.02 |
| | 29.40 | 2.25 | 29.74 | 2.06 | 29.78 | 2.07 | 2.02 |
| | 29.38 | 2.27 | 29.65 | 2.14 | 29.71 | 2.13 | 2.00 |
| | 29.31 | 2.33 | 29.73 | 2.07 | 29.72 | 2.13 | 2.00 |
| | 29.18 | 2.45 | 29.56 | 2.23 | 29.61 | 2.23 | 2.00 |
| **16** | 27.50 | 4.00 | 28.01 | 3.68 | 28.04 | 3.70 | 3.75 |
| | 25.84 | 5.53 | 26.13 | 5.45 | 26.10 | 5.51 | 5.21 |
| | 26.37 | 5.04 | 26.38 | 5.22 | 26.39 | 5.24 | 5.04 |
| | 26.84 | 4.60 | 26.83 | 4.80 | 26.90 | 4.76 | 4.57 |
| | 26.41 | 5.00 | 26.46 | 5.14 | 26.48 | 5.16 | 5.03 |
| | 25.87 | 5.50 | 25.95 | 5.62 | 25.98 | 5.62 | 5.44 |
| | 24.37 | 6.88 | 24.93 | 6.58 | 24.93 | 6.60 | 6.62 |
| | 24.81 | 6.48 | 25.42 | 6.12 | 25.39 | 6.17 | 6.30 |
| | 24.42 | 6.83 | 25.00 | 6.52 | 25.02 | 6.52 | 6.58 |
| | 24.47 | 6.79 | 25.10 | 6.43 | 25.06 | 6.49 | 6.49 |
| **17** | 29.69 | 1.99 | 29.99 | 1.83 | 30.06 | 1.82 | 1.77 |
| | 27.66 | 3.85 | 27.73 | 3.95 | 27.84 | 3.89 | 3.46 |

| | | | | | | | |
|---|---|---|---|---|---|---|---|
| | 28.33 | 3.23 | 28.21 | 3.50 | 28.31 | 3.45 | 3.39 |
| | 26.00 | 5.38 | 25.99 | 5.59 | 26.05 | 5.55 | 5.59 |
| | 27.96 | 3.57 | 28.14 | 3.57 | 28.22 | 3.53 | 4.08 |
| | 27.94 | 3.60 | 27.94 | 3.76 | 28.03 | 3.71 | 3.62 |
| | 27.28 | 4.20 | 27.14 | 4.50 | 27.23 | 4.46 | 4.18 |
| | 28.05 | 3.49 | 27.96 | 3.73 | 28.05 | 3.69 | 2.76 |
| | 28.17 | 3.38 | 28.33 | 3.38 | 28.43 | 3.33 | 3.25 |
| | 27.98 | 3.56 | 28.25 | 3.46 | 28.37 | 3.39 | 3.23 |
| | 28.24 | 3.32 | 28.50 | 3.22 | 28.59 | 3.19 | 3.11 |
| | 28.45 | 3.12 | 28.34 | 3.37 | 28.40 | 3.36 | 2.94 |
| | 29.54 | 2.12 | 29.63 | 2.16 | 29.69 | 2.16 | 2.04 |
| | 27.52 | 3.98 | 27.89 | 3.80 | 27.93 | 3.80 | 3.93 |
| | 27.32 | 4.17 | 27.40 | 4.26 | 27.47 | 4.23 | 4.29 |
| | 29.42 | 2.23 | 29.66 | 2.13 | 29.69 | 2.16 | 2.36 |
| | 29.48 | 2.18 | 29.54 | 2.25 | 29.57 | 2.27 | 1.90 |
| | 28.71 | 2.88 | 28.72 | 3.02 | 28.77 | 3.02 | 2.28 |
| | 29.72 | 1.95 | 29.85 | 1.96 | 29.94 | 1.92 | 2.01 |
| | 26.52 | 4.90 | 26.48 | 5.13 | 26.51 | 5.13 | 5.36 |
| | 29.27 | 2.36 | 29.33 | 2.45 | 29.38 | 2.45 | 2.18 |
| | 27.28 | 4.20 | 27.67 | 4.01 | 27.68 | 4.03 | 4.03 |
| | 27.72 | 3.79 | 27.93 | 3.76 | 27.93 | 3.80 | 3.58 |
| | 30.58 | 1.17 | 30.82 | 1.04 | 30.90 | 1.02 | 0.89 |
| | 29.75 | 1.93 | 29.92 | 1.89 | 29.98 | 1.89 | 0.89 |
| | 30.02 | 1.68 | 30.34 | 1.49 | 30.40 | 1.49 | 0.89 |
| | 23.12 | 8.03 | 23.36 | 8.06 | 23.35 | 8.08 | 8.16 |
| | 23.66 | 7.54 | 24.01 | 7.45 | 24.02 | 7.45 | 7.57 |
| | 23.58 | 7.61 | 23.83 | 7.62 | 23.86 | 7.60 | 7.66 |
| | 23.69 | 7.50 | 24.04 | 7.42 | 24.06 | 7.42 | 7.57 |
| | 23.06 | 8.09 | 23.32 | 8.10 | 23.30 | 8.12 | 8.16 |
| | 27.24 | 4.24 | 27.69 | 3.99 | 27.72 | 4.00 | 3.62 |
| | 27.72 | 3.80 | 28.18 | 3.53 | 28.21 | 3.54 | 3.56 |
| | 27.50 | 4.00 | 27.98 | 3.71 | 28.03 | 3.71 | 3.75 |
| | 27.38 | 4.11 | 27.68 | 3.99 | 27.74 | 3.98 | 3.89 |
| **18** | 24.20 | 7.04 | 24.57 | 6.93 | 24.58 | 6.93 | 6.62 |
| | 29.27 | 2.37 | 29.41 | 2.37 | 29.49 | 2.34 | 1.98 |
| | 27.85 | 3.67 | 28.03 | 3.67 | 28.08 | 3.66 | 3.11 |
| | 25.24 | 6.08 | 25.50 | 6.05 | 25.52 | 6.05 | 5.70 |
| | 27.71 | 3.80 | 27.90 | 3.79 | 28.00 | 3.73 | 3.49 |
| | 29.02 | 2.60 | 28.98 | 2.77 | 29.07 | 2.74 | 2.20 |
| | 28.51 | 3.07 | 28.22 | 3.49 | 28.26 | 3.49 | 3.11 |
| | 29.60 | 2.06 | 29.37 | 2.41 | 29.46 | 2.37 | 2.77 |
| | 29.29 | 2.35 | 29.49 | 2.29 | 29.55 | 2.29 | 2.11 |
| | 30.26 | 1.46 | 30.40 | 1.44 | 30.44 | 1.45 | 0.94 |
| | 28.76 | 2.84 | 28.60 | 3.13 | 28.66 | 3.11 | 2.87 |
| | 27.20 | 4.27 | 27.66 | 4.02 | 27.70 | 4.01 | 3.58 |
| | 27.67 | 3.84 | 27.89 | 3.80 | 27.90 | 3.83 | 4.25 |
| | 29.78 | 1.90 | 29.88 | 1.93 | 29.94 | 1.93 | 1.93 |
| | 29.65 | 2.02 | 29.76 | 2.04 | 29.84 | 2.02 | 2.10 |
| | 27.05 | 4.42 | 27.23 | 4.42 | 27.28 | 4.41 | 4.56 |
| | 29.96 | 1.73 | 30.24 | 1.58 | 30.32 | 1.57 | 1.81 |

| | | | | | | | |
|---|---|---|---|---|---|---|---|
| | 29.64 | 2.03 | 29.91 | 1.90 | 29.97 | 1.90 | 1.81 |
| | 29.00 | 2.61 | 29.32 | 2.45 | 29.36 | 2.47 | 1.81 |
| | 30.48 | 1.25 | 30.85 | 1.02 | 30.91 | 1.02 | 1.17 |
| | 29.96 | 1.73 | 30.35 | 1.48 | 30.39 | 1.50 | 1.17 |
| | 30.37 | 1.36 | 30.80 | 1.06 | 30.85 | 1.07 | 1.17 |
| | 29.71 | 1.97 | 29.83 | 1.97 | 29.89 | 1.97 | 1.11 |
| | 30.63 | 1.12 | 30.80 | 1.06 | 30.87 | 1.05 | 1.11 |
| | 30.41 | 1.32 | 30.52 | 1.32 | 30.59 | 1.32 | 1.11 |
| **19** | 25.14 | 6.17 | 25.82 | 5.74 | 25.77 | 5.82 | 5.97 |
| | 28.93 | 2.68 | 29.23 | 2.53 | 29.26 | 2.56 | 2.91 |
| | 28.84 | 2.77 | 29.12 | 2.64 | 29.14 | 2.67 | 2.74 |
| | 27.16 | 4.31 | 26.88 | 4.75 | 26.94 | 4.73 | 4.97 |
| | 27.93 | 3.60 | 27.77 | 3.91 | 27.80 | 3.92 | 4.74 |
| | 25.14 | 6.17 | 25.76 | 5.80 | 25.72 | 5.87 | 6.00 |
| | 26.47 | 4.95 | 26.53 | 5.07 | 26.60 | 5.04 | 5.11 |
| | 24.48 | 6.78 | 24.73 | 6.77 | 24.76 | 6.76 | 6.99 |
| | 24.70 | 6.58 | 24.97 | 6.54 | 24.96 | 6.58 | 6.99 |
| | 24.57 | 6.70 | 25.09 | 6.43 | 25.08 | 6.46 | 6.73 |
| | 24.62 | 6.65 | 25.18 | 6.35 | 25.12 | 6.42 | 6.71 |
| | 26.40 | 5.01 | 26.35 | 5.25 | 26.38 | 5.25 | 6.99 |
| | 24.15 | 7.08 | 24.47 | 7.01 | 24.50 | 7.01 | 6.99 |
| | 27.07 | 4.39 | 27.19 | 4.45 | 27.25 | 4.44 | 4.31 |
| | 25.26 | 6.06 | 25.76 | 5.80 | 25.78 | 5.81 | 6.01 |
| | 27.54 | 3.96 | 27.42 | 4.24 | 27.44 | 4.25 | 4.02 |
| **20** | 27.80 | 3.72 | 27.69 | 3.99 | 27.74 | 3.98 | 3.96 |
| | 29.36 | 2.29 | 29.43 | 2.35 | 29.50 | 2.34 | 2.35 |
| | 30.31 | 1.41 | 30.38 | 1.46 | 30.41 | 1.48 | 1.44 |
| | 28.65 | 2.94 | 28.43 | 3.29 | 28.50 | 3.27 | 3.13 |
| | 31.02 | 0.75 | 30.48 | 1.37 | 30.53 | 1.38 | 1.26 |
| | 28.01 | 3.53 | 27.57 | 4.10 | 27.60 | 4.11 | 3.85 |
| | 23.00 | 8.14 | 23.37 | 8.05 | 23.36 | 8.07 | 8.08 |
| | 23.97 | 7.25 | 24.25 | 7.22 | 24.30 | 7.19 | 7.24 |
| | 24.12 | 7.11 | 24.42 | 7.06 | 24.45 | 7.06 | 7.08 |
| | 24.02 | 7.21 | 24.31 | 7.17 | 24.31 | 7.18 | 7.16 |
| | 29.73 | 1.95 | 29.80 | 2.00 | 29.86 | 2.00 | 1.89 |
| | 29.89 | 1.80 | 29.96 | 1.85 | 30.01 | 1.86 | 1.87 |
| | 28.53 | 3.05 | 28.76 | 2.98 | 28.81 | 2.98 | 3.21 |
| | 28.75 | 2.85 | 28.68 | 3.05 | 28.69 | 3.09 | 2.87 |
| | 28.94 | 2.67 | 29.03 | 2.72 | 29.08 | 2.72 | 2.74 |
| | 27.86 | 3.67 | 28.06 | 3.64 | 28.08 | 3.66 | 3.71 |
| | 25.24 | 6.08 | 25.50 | 6.05 | 25.50 | 6.07 | 5.92 |
| | 27.55 | 3.95 | 27.62 | 4.06 | 27.63 | 4.08 | 4.05 |
| | 27.43 | 4.06 | 27.78 | 3.91 | 27.81 | 3.91 | 4.14 |
| | 27.45 | 4.05 | 27.51 | 4.16 | 27.56 | 4.14 | 4.27 |
| | 28.63 | 2.96 | 28.73 | 3.01 | 28.77 | 3.01 | 3.11 |
| | 28.97 | 2.64 | 29.22 | 2.55 | 29.26 | 2.56 | 2.66 |
| **21** | 22.91 | 8.22 | 23.33 | 8.09 | 23.28 | 8.14 | 8.23 |
| | 29.74 | 1.94 | 29.77 | 2.03 | 29.83 | 2.03 | 1.72 |
| | 28.32 | 3.25 | 28.23 | 3.48 | 28.24 | 3.51 | 3.24 |
| | 28.12 | 3.43 | 28.27 | 3.44 | 28.30 | 3.45 | 3.18 |

| | | | | | | | |
|---|---|---|---|---|---|---|---|
| | 29.69 | 1.99 | 29.69 | 2.11 | 29.74 | 2.11 | 2.46 |
| | 27.70 | 3.81 | 27.79 | 3.90 | 27.80 | 3.92 | 3.62 |
| | 23.90 | 7.31 | 24.20 | 7.27 | 24.19 | 7.29 | 7.26 |
| | 24.10 | 7.13 | 24.42 | 7.06 | 24.45 | 7.05 | 7.18 |
| | 24.02 | 7.20 | 24.35 | 7.13 | 24.38 | 7.12 | 7.29 |
| | 29.32 | 2.32 | 29.05 | 2.71 | 29.11 | 2.70 | 2.88 |
| | 27.96 | 3.57 | 27.68 | 4.00 | 27.73 | 3.99 | 3.86 |
| | 28.98 | 2.64 | 29.13 | 2.63 | 29.12 | 2.69 | 1.78 |
| | 30.32 | 1.40 | 30.60 | 1.25 | 30.65 | 1.26 | 1.78 |
| | 27.37 | 4.12 | 27.23 | 4.42 | 27.28 | 4.41 | 4.45 |
| | 28.43 | 3.14 | 28.14 | 3.57 | 28.18 | 3.57 | 2.74 |
| | 28.81 | 2.79 | 28.88 | 2.87 | 28.91 | 2.89 | 3.36 |
| | 26.00 | 5.38 | 26.18 | 5.41 | 26.21 | 5.41 | 5.43 |
| | 29.43 | 2.22 | 29.37 | 2.41 | 29.40 | 2.43 | 2.14 |
| | 28.59 | 2.99 | 28.73 | 3.01 | 28.78 | 3.01 | 3.03 |
| | 29.43 | 2.22 | 29.54 | 2.25 | 29.63 | 2.22 | 1.73 |
| | 28.88 | 2.73 | 28.60 | 3.13 | 28.69 | 3.09 | 2.57 |
| | 30.02 | 1.68 | 30.29 | 1.54 | 30.35 | 1.54 | 1.78 |
| | 27.39 | 4.10 | 27.37 | 4.29 | 27.44 | 4.26 | 4.62 |
| | 29.38 | 2.27 | 29.61 | 2.18 | 29.63 | 2.21 | 1.72 |
| | 29.86 | 1.83 | 30.24 | 1.59 | 30.28 | 1.61 | 1.72 |
| | 29.95 | 1.74 | 30.35 | 1.49 | 30.39 | 1.50 | 1.72 |
| | 25.78 | 5.58 | 25.99 | 5.58 | 26.02 | 5.59 | 5.62 |
| | 28.07 | 3.47 | 28.16 | 3.54 | 28.20 | 3.55 | 3.59 |
| | 28.66 | 2.93 | 28.45 | 3.27 | 28.49 | 3.28 | 2.91 |
| | 29.93 | 1.76 | 30.04 | 1.78 | 30.06 | 1.81 | 1.62 |
| | 29.70 | 1.97 | 29.79 | 2.01 | 29.86 | 2.00 | 2.04 |
| | 28.74 | 2.86 | 28.58 | 3.15 | 28.61 | 3.16 | 3.03 |
| | 30.20 | 1.51 | 29.77 | 2.03 | 29.83 | 2.02 | 1.61 |
| | 28.41 | 3.16 | 28.17 | 3.54 | 28.25 | 3.51 | 3.06 |
| | 24.78 | 6.51 | 24.95 | 6.57 | 24.95 | 6.58 | 6.52 |
| | 24.23 | 7.01 | 24.46 | 7.03 | 24.49 | 7.01 | 7.17 |
| | 24.53 | 6.73 | 24.84 | 6.67 | 24.87 | 6.66 | 6.85 |
| | 24.22 | 7.02 | 24.44 | 7.04 | 24.46 | 7.04 | 7.09 |
| | 28.50 | 3.08 | 28.73 | 3.01 | 28.76 | 3.03 | 3.18 |
| | 28.89 | 2.72 | 29.12 | 2.64 | 29.14 | 2.67 | 2.71 |
| | 28.37 | 3.20 | 28.17 | 3.53 | 28.23 | 3.52 | 3.51 |
| **22** | 29.49 | 2.16 | 29.59 | 2.20 | 29.65 | 2.20 | 1.52 |
| | 30.15 | 1.55 | 30.26 | 1.57 | 30.33 | 1.56 | 1.52 |
| | 30.09 | 1.62 | 30.28 | 1.56 | 30.35 | 1.54 | 1.52 |
| | 29.69 | 1.98 | 29.97 | 1.85 | 30.01 | 1.86 | 1.52 |
| | 30.40 | 1.33 | 30.64 | 1.21 | 30.71 | 1.20 | 1.52 |
| | 30.06 | 1.64 | 30.29 | 1.54 | 30.37 | 1.53 | 1.52 |
| | 24.90 | 6.40 | 25.02 | 6.50 | 25.06 | 6.48 | 6.16 |
| | 23.86 | 7.35 | 24.20 | 7.28 | 24.23 | 7.25 | 7.21 |
| | 23.80 | 7.40 | 24.17 | 7.30 | 24.19 | 7.30 | 7.32 |
| | 28.60 | 2.99 | 28.50 | 3.22 | 28.51 | 3.26 | 3.27 |
| | 28.36 | 3.21 | 28.47 | 3.26 | 28.50 | 3.27 | 3.35 |
| | 24.09 | 7.14 | 24.39 | 7.09 | 24.44 | 7.06 | 6.93 |
| | 24.01 | 7.21 | 24.30 | 7.17 | 24.35 | 7.15 | 7.03 |

| | | | | | | | |
|---|---|---|---|---|---|---|---|
| | 24.19 | 7.05 | 24.44 | 7.05 | 24.48 | 7.02 | 7.48 |
| | 25.88 | 5.49 | 26.24 | 5.35 | 26.30 | 5.32 | 5.10 |
| | 25.91 | 5.46 | 26.20 | 5.39 | 26.25 | 5.37 | 4.99 |
| | 23.99 | 7.23 | 24.24 | 7.24 | 24.26 | 7.23 | 7.39 |
| | 23.73 | 7.47 | 24.15 | 7.32 | 24.16 | 7.32 | 7.49 |
| | 24.11 | 7.12 | 24.42 | 7.07 | 24.45 | 7.05 | 7.10 |
| | 23.95 | 7.27 | 24.24 | 7.23 | 24.28 | 7.21 | 7.19 |
| | 30.15 | 1.56 | 30.47 | 1.37 | 30.51 | 1.39 | 1.57 |
| | 30.02 | 1.67 | 30.27 | 1.56 | 30.32 | 1.56 | 1.57 |
| | 30.18 | 1.54 | 30.47 | 1.37 | 30.50 | 1.40 | 1.57 |
| | 31.66 | 0.17 | 31.61 | 0.30 | 31.67 | 0.31 | 1.29 |
| | 31.18 | 0.61 | 31.40 | 0.50 | 31.47 | 0.50 | 1.29 |
| | 33.19 | -1.24 | 33.17 | -1.17 | 33.22 | -1.14 | 1.29 |
| **23** | 27.33 | 4.16 | 27.14 | 4.50 | 27.19 | 4.49 | 4.62 |
| | 28.68 | 2.91 | 28.59 | 3.14 | 28.67 | 3.11 | 5.01 |
| | 28.88 | 2.73 | 28.80 | 2.94 | 28.86 | 2.93 | 2.05 |
| | 31.02 | 0.76 | 30.83 | 1.04 | 30.92 | 1.01 | 1.60 |
| | 26.57 | 4.86 | 26.70 | 4.92 | 26.74 | 4.91 | 5.58 |
| | 26.83 | 4.61 | 26.67 | 4.94 | 26.73 | 4.93 | 4.68 |
| | 28.07 | 3.47 | 28.21 | 3.49 | 28.26 | 3.49 | 3.90 |
| | 28.45 | 3.12 | 28.62 | 3.11 | 28.70 | 3.08 | 3.09 |
| | 28.59 | 2.99 | 28.56 | 3.17 | 28.61 | 3.16 | 3.70 |
| | 29.04 | 2.58 | 29.32 | 2.45 | 29.37 | 2.45 | 2.80 |
| | 29.70 | 1.97 | 29.82 | 1.98 | 29.89 | 1.97 | 2.71 |
| | 30.53 | 1.21 | 30.50 | 1.35 | 30.60 | 1.31 | 1.81 |
| | 30.58 | 1.16 | 30.61 | 1.24 | 30.66 | 1.25 | 1.81 |
| | 27.25 | 4.23 | 27.15 | 4.50 | 27.21 | 4.47 | 4.85 |
| | 30.11 | 1.59 | 30.25 | 1.58 | 30.31 | 1.58 | 1.81 |
| | 30.37 | 1.35 | 30.46 | 1.38 | 30.56 | 1.35 | 1.81 |
| | 28.02 | 3.52 | 27.93 | 3.76 | 27.98 | 3.75 | 4.32 |
| | 28.63 | 2.96 | 28.89 | 2.85 | 28.95 | 2.85 | 3.06 |
| | 29.09 | 2.54 | 29.41 | 2.37 | 29.45 | 2.38 | 3.06 |
| | 27.51 | 3.99 | 27.89 | 3.80 | 27.96 | 3.78 | 3.95 |
| | 27.63 | 3.88 | 28.13 | 3.58 | 28.21 | 3.54 | 3.92 |
| | 26.64 | 4.79 | 26.98 | 4.66 | 26.98 | 4.69 | 4.13 |
| | 27.31 | 4.18 | 27.68 | 4.00 | 27.72 | 4.00 | 3.85 |
| | 23.83 | 7.37 | 23.84 | 7.61 | 23.85 | 7.61 | 8.03 |

[a] Parameters of linear regression models are presented in Table 2 in main text.
[b] For the references to the experimental works, please, see the main text.

**Table S2**. Calculated $^{1}H$ NMR shielding constants, together with scaled and experimental chemical shifts – the GIAO-DFT(PBE0) calculations with the second-level basis sets.

| Mol. | pcS-2 | | pecS-2 | | pecS-2 mod | | $\delta_{exp}$[b] |
|---|---|---|---|---|---|---|---|
| | $\sigma$ | $\delta_{scaled}$[a] | $\sigma$ | $\delta_{scaled}$[a] | $\sigma$ | $\delta_{scaled}$[a] | |
| **1** | 28.41 | 3.06 | 28.34 | 3.09 | 28.36 | 3.11 | 4.38 |
| | 29.89 | 1.72 | 29.80 | 1.76 | 29.84 | 1.76 | 2.51 |

| | | | | | | | |
|---|---|---|---|---|---|---|---|
| | 30.39 | 1.26 | 30.32 | 1.28 | 30.36 | 1.28 | 2.31 |
| | 28.81 | 2.69 | 28.77 | 2.69 | 28.80 | 2.70 | 3.53 |
| | 28.79 | 2.71 | 28.75 | 2.71 | 28.77 | 2.73 | 3.21 |
| | 27.43 | 3.95 | 27.37 | 3.97 | 27.42 | 3.96 | 4.03 |
| | 28.95 | 2.57 | 28.91 | 2.57 | 28.93 | 2.58 | 3.18 |
| | 28.40 | 3.07 | 28.41 | 3.02 | 28.44 | 3.03 | 3.34 |
| | 28.62 | 2.87 | 28.60 | 2.85 | 28.64 | 2.85 | 3.24 |
| | 28.82 | 2.68 | 28.81 | 2.66 | 28.85 | 2.66 | 2.80 |
| | 28.40 | 3.07 | 28.33 | 3.10 | 28.38 | 3.09 | 3.00 |
| | 28.13 | 3.32 | 28.05 | 3.35 | 28.10 | 3.34 | 3.27 |
| | 25.47 | 5.73 | 25.43 | 5.73 | 25.48 | 5.73 | 5.42 |
| | 23.67 | 7.37 | 23.66 | 7.35 | 23.71 | 7.34 | 7.45 |
| | 24.24 | 6.85 | 24.20 | 6.86 | 24.23 | 6.86 | 7.25 |
| | 29.27 | 2.28 | 29.21 | 2.29 | 29.26 | 2.28 | 0.99 |
| | 29.79 | 1.81 | 29.79 | 1.77 | 29.82 | 1.77 | 0.99 |
| | 29.81 | 1.79 | 29.81 | 1.75 | 29.84 | 1.75 | 0.99 |
| | 24.10 | 6.98 | 24.11 | 6.93 | 24.15 | 6.94 | 7.01 |
| | 24.13 | 6.95 | 24.13 | 6.92 | 24.17 | 6.92 | 7.05 |
| | 23.21 | 7.78 | 23.18 | 7.78 | 23.23 | 7.78 | 7.81 |
| | 22.70 | 8.25 | 22.66 | 8.25 | 22.70 | 8.26 | 8.11 |
| | 23.55 | 7.48 | 23.52 | 7.47 | 23.58 | 7.46 | 7.47 |
| | 23.73 | 7.32 | 23.71 | 7.30 | 23.75 | 7.31 | 7.40 |
| | 23.93 | 7.14 | 23.92 | 7.11 | 23.97 | 7.10 | 7.10 |
| **2** | 24.09 | 6.99 | 24.09 | 6.95 | 24.14 | 6.95 | 6.94 |
| | 23.70 | 7.34 | 23.66 | 7.34 | 23.70 | 7.35 | 7.62 |
| | 23.83 | 7.22 | 23.82 | 7.20 | 23.86 | 7.20 | 7.09 |
| | 23.97 | 7.10 | 23.96 | 7.07 | 24.01 | 7.07 | 7.19 |
| | 23.46 | 7.56 | 23.44 | 7.54 | 23.50 | 7.54 | 7.36 |
| | 24.62 | 6.51 | 24.58 | 6.51 | 24.65 | 6.49 | 6.94 |
| | 23.87 | 7.19 | 23.85 | 7.17 | 23.90 | 7.17 | 7.36 |
| | 24.18 | 6.90 | 24.18 | 6.87 | 24.22 | 6.88 | 7.19 |
| | 23.96 | 7.10 | 23.95 | 7.08 | 23.99 | 7.08 | 7.09 |
| | 23.84 | 7.22 | 23.80 | 7.22 | 23.84 | 7.23 | 7.62 |
| | 23.27 | 7.73 | 23.24 | 7.72 | 23.29 | 7.72 | 7.73 |
| | 24.17 | 6.91 | 24.17 | 6.88 | 24.21 | 6.89 | 6.89 |
| | 23.53 | 7.49 | 23.52 | 7.47 | 23.57 | 7.47 | 7.51 |
| | 24.25 | 6.84 | 24.23 | 6.82 | 24.27 | 6.83 | 6.91 |
| **3** | 27.04 | 4.30 | 26.88 | 4.41 | 26.94 | 4.40 | 4.18 |
| | 28.13 | 3.32 | 28.05 | 3.35 | 28.10 | 3.34 | 2.88 |
| | 29.44 | 2.13 | 29.39 | 2.13 | 29.41 | 2.15 | 2.20 |
| | 28.66 | 2.84 | 28.58 | 2.87 | 28.62 | 2.87 | 2.60 |
| | 28.82 | 2.69 | 28.77 | 2.70 | 28.80 | 2.70 | 2.35 |
| | 29.76 | 1.83 | 29.69 | 1.86 | 29.73 | 1.86 | 1.81 |
| | 30.04 | 1.58 | 30.00 | 1.58 | 30.03 | 1.58 | 1.51 |
| | 29.88 | 1.72 | 29.80 | 1.76 | 29.85 | 1.75 | 2.34 |
| | 25.11 | 6.06 | 25.03 | 6.10 | 25.08 | 6.09 | 6.40 |
| | 28.63 | 2.86 | 28.59 | 2.86 | 28.62 | 2.86 | 2.80 |
| | 29.84 | 1.76 | 29.80 | 1.76 | 29.82 | 1.77 | 1.97 |
| | 29.40 | 2.16 | 29.40 | 2.12 | 29.42 | 2.14 | 1.94 |
| | 29.77 | 1.83 | 29.73 | 1.82 | 29.77 | 1.82 | 1.57 |

| | | | | | | | |
|---|---|---|---|---|---|---|---|
| | 28.58 | 2.91 | 28.54 | 2.90 | 28.58 | 2.90 | 3.24 |
| | 27.46 | 3.92 | 27.42 | 3.92 | 27.46 | 3.92 | 3.80 |
| | 29.26 | 2.29 | 29.25 | 2.26 | 29.27 | 2.27 | 2.44 |
| | 29.05 | 2.48 | 29.03 | 2.46 | 29.06 | 2.47 | 2.54 |
| | 28.94 | 2.58 | 28.83 | 2.64 | 28.89 | 2.62 | 2.07 |
| | 29.10 | 2.44 | 29.07 | 2.42 | 29.10 | 2.43 | 2.31 |
| | 29.27 | 2.28 | 29.14 | 2.36 | 29.19 | 2.35 | 1.70 |
| | 30.14 | 1.49 | 30.08 | 1.50 | 30.12 | 1.50 | 1.61 |
| | 30.15 | 1.48 | 30.14 | 1.45 | 30.16 | 1.46 | 1.47 |
| | 30.21 | 1.43 | 30.17 | 1.42 | 30.20 | 1.42 | 1.47 |
| | 30.44 | 1.22 | 30.37 | 1.24 | 30.41 | 1.24 | 1.38 |
| | 30.01 | 1.61 | 29.98 | 1.59 | 30.02 | 1.59 | 1.38 |
| | 25.52 | 5.68 | 25.52 | 5.66 | 25.55 | 5.66 | 5.58 |
| | 29.17 | 2.37 | 29.16 | 2.34 | 29.19 | 2.35 | 2.56 |
| | 29.19 | 2.35 | 29.16 | 2.34 | 29.20 | 2.34 | 2.58 |
| | 29.85 | 1.76 | 29.83 | 1.73 | 29.85 | 1.74 | 1.86 |
| | 28.44 | 3.04 | 28.31 | 3.11 | 28.37 | 3.09 | 2.43 |
| | 25.84 | 5.40 | 25.81 | 5.39 | 25.85 | 5.39 | 5.47 |
| | 22.66 | 8.29 | 22.62 | 8.29 | 22.66 | 8.30 | 8.33 |
| | 23.12 | 7.87 | 23.10 | 7.86 | 23.14 | 7.86 | 8.02 |
| | 23.67 | 7.37 | 23.60 | 7.40 | 23.65 | 7.40 | 6.24 |
| | 22.74 | 8.21 | 22.75 | 8.17 | 22.80 | 8.17 | 8.23 |
| | 23.52 | 7.50 | 23.50 | 7.49 | 23.54 | 7.50 | 7.64 |
| | 25.09 | 6.08 | 25.02 | 6.11 | 25.07 | 6.10 | 5.75 |
| | 23.73 | 7.32 | 23.73 | 7.28 | 23.77 | 7.29 | 7.25 |
| | 23.42 | 7.59 | 23.42 | 7.56 | 23.46 | 7.56 | 7.56 |
| **4** | 27.43 | 3.95 | 27.35 | 3.99 | 27.39 | 3.99 | 4.11 |
| | 26.94 | 4.40 | 26.84 | 4.45 | 26.89 | 4.45 | 4.26 |
| | 28.38 | 3.09 | 28.32 | 3.10 | 28.37 | 3.09 | 2.76 |
| | 28.29 | 3.17 | 28.17 | 3.25 | 28.24 | 3.21 | 2.95 |
| | 23.80 | 7.25 | 23.77 | 7.25 | 23.82 | 7.24 | 7.56 |
| | 24.05 | 7.02 | 24.03 | 7.01 | 24.06 | 7.02 | 6.91 |
| | 23.64 | 7.39 | 23.64 | 7.37 | 23.68 | 7.37 | 7.19 |
| | 24.05 | 7.02 | 24.03 | 7.01 | 24.06 | 7.02 | 6.96 |
| | 28.40 | 3.07 | 28.37 | 3.06 | 28.41 | 3.06 | 2.79 |
| | 28.52 | 2.96 | 28.45 | 2.98 | 28.51 | 2.97 | 2.51 |
| | 30.54 | 1.12 | 30.45 | 1.16 | 30.49 | 1.16 | 0.72 |
| | 27.20 | 4.16 | 27.18 | 4.14 | 27.21 | 4.15 | 3.83 |
| | 27.31 | 4.06 | 27.32 | 4.01 | 27.34 | 4.03 | 4.05 |
| | 29.77 | 1.82 | 29.70 | 1.85 | 29.74 | 1.84 | 1.56 |
| | 29.62 | 1.96 | 29.57 | 1.96 | 29.63 | 1.95 | 1.62 |
| | 27.01 | 4.33 | 26.93 | 4.37 | 26.98 | 4.37 | 4.49 |
| | 27.41 | 3.97 | 27.40 | 3.94 | 27.44 | 3.94 | 3.78 |
| | 27.45 | 3.94 | 27.41 | 3.93 | 27.45 | 3.94 | 3.78 |
| | 27.47 | 3.91 | 27.43 | 3.92 | 27.47 | 3.92 | 3.78 |
| | 29.04 | 2.49 | 29.01 | 2.48 | 29.04 | 2.49 | 2.37 |
| | 28.44 | 3.04 | 28.45 | 2.98 | 28.48 | 2.99 | 2.88 |
| | 29.07 | 2.46 | 29.05 | 2.44 | 29.07 | 2.45 | 2.31 |
| | 28.25 | 3.20 | 28.28 | 3.14 | 28.30 | 3.16 | 3.09 |
| | 30.17 | 1.46 | 30.13 | 1.45 | 30.17 | 1.45 | 1.20 |

| | | | | | | | |
|---|---|---|---|---|---|---|---|
| | 28.35 | 3.12 | 28.30 | 3.12 | 28.35 | 3.11 | 2.74 |
| | 24.19 | 6.90 | 24.13 | 6.92 | 24.17 | 6.92 | 6.65 |
| | 24.97 | 6.18 | 24.93 | 6.19 | 24.98 | 6.18 | 5.85 |
| | 24.85 | 6.29 | 24.78 | 6.33 | 24.83 | 6.32 | 6.22 |
| | 29.39 | 2.17 | 29.38 | 2.14 | 29.42 | 2.14 | 1.95 |
| | 29.38 | 2.18 | 29.36 | 2.16 | 29.38 | 2.17 | 1.99 |
| | 27.77 | 3.64 | 27.74 | 3.63 | 27.76 | 3.65 | 3.54 |
| | 29.05 | 2.48 | 28.93 | 2.55 | 28.98 | 2.54 | 2.28 |
| | 30.49 | 1.17 | 30.40 | 1.21 | 30.43 | 1.21 | 1.07 |
| | 29.34 | 2.21 | 29.30 | 2.21 | 29.32 | 2.23 | 1.90 |
| | 26.92 | 4.41 | 26.89 | 4.41 | 26.92 | 4.42 | 4.01 |
| | 27.17 | 4.19 | 27.16 | 4.16 | 27.19 | 4.17 | 4.23 |
| | 28.40 | 3.07 | 28.30 | 3.12 | 28.35 | 3.11 | 2.07 |
| | 29.43 | 2.13 | 29.36 | 2.16 | 29.40 | 2.16 | 2.59 |
| | 28.60 | 2.88 | 28.50 | 2.94 | 28.54 | 2.94 | 2.80 |
| | 30.02 | 1.60 | 29.95 | 1.62 | 29.98 | 1.63 | 1.49 |
| | 29.14 | 2.40 | 29.07 | 2.42 | 29.10 | 2.43 | 2.15 |
| | 27.54 | 3.85 | 27.53 | 3.82 | 27.56 | 3.84 | 3.71 |
| | 27.56 | 3.83 | 27.53 | 3.83 | 27.57 | 3.83 | 3.83 |
| | 27.55 | 3.84 | 27.51 | 3.84 | 27.54 | 3.85 | 3.82 |
| | 27.28 | 4.09 | 27.17 | 4.15 | 27.23 | 4.14 | 3.92 |
| | 23.47 | 7.55 | 23.36 | 7.62 | 23.42 | 7.60 | 7.68 |
| | 23.60 | 7.43 | 23.58 | 7.42 | 23.63 | 7.42 | 7.26 |
| | 23.80 | 7.25 | 23.78 | 7.24 | 23.82 | 7.24 | 7.11 |
| | 23.56 | 7.47 | 23.55 | 7.44 | 23.60 | 7.45 | 7.33 |
| | 24.09 | 6.98 | 24.06 | 6.98 | 24.10 | 6.98 | 7.02 |
| | 27.87 | 3.55 | 27.82 | 3.56 | 27.84 | 3.58 | 3.46 |
| | 28.52 | 2.96 | 28.42 | 3.01 | 28.48 | 2.99 | 2.36 |
| | 28.86 | 2.65 | 28.81 | 2.66 | 28.84 | 2.66 | 2.66 |
| | 27.48 | 3.90 | 27.48 | 3.87 | 27.50 | 3.88 | 3.75 |
| | 27.47 | 3.91 | 27.46 | 3.88 | 27.49 | 3.90 | 3.75 |
| | 29.79 | 1.80 | 29.75 | 1.81 | 29.79 | 1.80 | 1.43 |
| | 29.74 | 1.85 | 29.71 | 1.84 | 29.73 | 1.85 | 1.60 |
| | 26.94 | 4.40 | 26.85 | 4.44 | 26.90 | 4.43 | 4.29 |
| | 27.73 | 3.68 | 27.69 | 3.68 | 27.72 | 3.69 | 3.69 |
| | 27.52 | 3.87 | 27.47 | 3.88 | 27.51 | 3.88 | 3.69 |
| | 27.56 | 3.83 | 27.55 | 3.81 | 27.59 | 3.81 | 3.69 |
| | 29.01 | 2.51 | 28.99 | 2.50 | 29.01 | 2.51 | 2.37 |
| | 28.43 | 3.04 | 28.45 | 2.99 | 28.47 | 3.00 | 2.88 |
| | 29.05 | 2.48 | 29.03 | 2.46 | 29.06 | 2.47 | 2.31 |
| | 28.25 | 3.21 | 28.27 | 3.15 | 28.29 | 3.17 | 3.09 |
| | 30.18 | 1.45 | 30.15 | 1.44 | 30.19 | 1.44 | 1.21 |
| | 28.38 | 3.09 | 28.33 | 3.09 | 28.38 | 3.08 | 2.75 |
| | 23.86 | 7.19 | 23.83 | 7.19 | 23.87 | 7.19 | 6.83 |
| | 24.52 | 6.59 | 24.47 | 6.61 | 24.51 | 6.61 | 6.35 |
| | 24.63 | 6.50 | 24.54 | 6.55 | 24.59 | 6.54 | 6.41 |
| | 29.36 | 2.20 | 29.35 | 2.16 | 29.38 | 2.17 | 1.95 |
| | 29.32 | 2.24 | 29.31 | 2.20 | 29.34 | 2.21 | 1.99 |
| | 27.74 | 3.67 | 27.71 | 3.66 | 27.74 | 3.67 | 3.51 |
| | 29.23 | 2.32 | 29.12 | 2.38 | 29.20 | 2.34 | 1.99 |

| | | | | | | | |
|---|---|---|---|---|---|---|---|
| | 30.38 | 1.27 | 30.30 | 1.30 | 30.34 | 1.30 | 0.97 |
| | 29.32 | 2.23 | 29.28 | 2.23 | 29.30 | 2.25 | 2.00 |
| | 27.22 | 4.14 | 27.21 | 4.11 | 27.23 | 4.13 | 3.94 |
| | 26.99 | 4.35 | 26.98 | 4.33 | 27.01 | 4.34 | 4.16 |
| | 29.48 | 2.09 | 29.42 | 2.10 | 29.45 | 2.11 | 1.74 |
| | 28.51 | 2.97 | 28.43 | 3.00 | 28.48 | 2.99 | 2.56 |
| | 28.53 | 2.95 | 28.46 | 2.98 | 28.48 | 2.99 | 2.79 |
| | 29.66 | 1.93 | 29.61 | 1.93 | 29.64 | 1.93 | 1.67 |
| | 29.14 | 2.40 | 29.12 | 2.37 | 29.15 | 2.39 | 2.20 |
| | 26.50 | 4.80 | 26.41 | 4.84 | 26.46 | 4.84 | 4.73 |
| | 28.11 | 3.34 | 28.08 | 3.33 | 28.11 | 3.33 | 3.20 |
| **5** | 29.11 | 2.42 | 29.04 | 2.45 | 29.08 | 2.45 | 2.48 |
| | 29.36 | 2.20 | 29.31 | 2.20 | 29.35 | 2.20 | 2.23 |
| | 28.09 | 3.35 | 28.05 | 3.35 | 28.08 | 3.36 | 3.41 |
| | 28.71 | 2.79 | 28.65 | 2.80 | 28.69 | 2.80 | 2.75 |
| | 30.00 | 1.62 | 29.92 | 1.65 | 29.98 | 1.63 | 1.67 |
| | 29.98 | 1.63 | 29.94 | 1.63 | 29.97 | 1.64 | 1.55 |
| | 27.34 | 4.03 | 27.26 | 4.07 | 27.29 | 4.08 | 4.23 |
| | 28.92 | 2.60 | 28.82 | 2.65 | 28.87 | 2.64 | 2.57 |
| | 29.71 | 1.88 | 29.59 | 1.95 | 29.64 | 1.94 | 1.95 |
| | 29.88 | 1.72 | 29.85 | 1.72 | 29.88 | 1.72 | 1.76 |
| | 30.10 | 1.52 | 30.07 | 1.51 | 30.09 | 1.52 | 1.56 |
| | 30.53 | 1.13 | 30.48 | 1.14 | 30.53 | 1.12 | 1.15 |
| | 30.30 | 1.34 | 30.27 | 1.33 | 30.31 | 1.33 | 1.15 |
| | 30.43 | 1.23 | 30.42 | 1.19 | 30.46 | 1.19 | 1.15 |
| | 29.78 | 1.81 | 29.76 | 1.80 | 29.79 | 1.80 | 1.84 |
| | 30.10 | 1.52 | 30.07 | 1.52 | 30.09 | 1.52 | 1.64 |
| | 30.50 | 1.17 | 30.50 | 1.12 | 30.52 | 1.13 | 1.25 |
| | 30.41 | 1.24 | 30.39 | 1.22 | 30.43 | 1.21 | 1.25 |
| | 30.13 | 1.49 | 30.10 | 1.49 | 30.14 | 1.48 | 1.25 |
| | 29.32 | 2.23 | 29.26 | 2.25 | 29.28 | 2.26 | 2.29 |
| | 26.73 | 4.59 | 26.66 | 4.61 | 26.69 | 4.63 | 4.74 |
| | 29.79 | 1.81 | 29.74 | 1.81 | 29.79 | 1.80 | 1.89 |
| | 29.19 | 2.35 | 29.16 | 2.34 | 29.21 | 2.33 | 1.89 |
| | 29.80 | 1.79 | 29.79 | 1.77 | 29.82 | 1.77 | 1.89 |
| | 29.88 | 1.73 | 29.85 | 1.71 | 29.88 | 1.72 | 1.63 |
| | 29.83 | 1.77 | 29.79 | 1.77 | 29.82 | 1.77 | 1.78 |
| | 29.65 | 1.94 | 29.54 | 2.00 | 29.58 | 1.99 | 1.98 |
| | 29.91 | 1.70 | 29.89 | 1.68 | 29.92 | 1.69 | 1.64 |
| | 29.77 | 1.83 | 29.74 | 1.82 | 29.77 | 1.82 | 1.84 |
| | 30.03 | 1.59 | 30.01 | 1.57 | 30.05 | 1.57 | 1.22 |
| | 30.26 | 1.38 | 30.24 | 1.35 | 30.28 | 1.35 | 1.22 |
| | 30.53 | 1.13 | 30.52 | 1.10 | 30.56 | 1.10 | 1.22 |
| | 30.87 | 0.82 | 30.84 | 0.81 | 30.88 | 0.81 | 1.16 |
| | 30.05 | 1.57 | 30.03 | 1.55 | 30.06 | 1.55 | 1.16 |
| | 30.31 | 1.34 | 30.30 | 1.30 | 30.33 | 1.31 | 1.16 |
| | 29.31 | 2.24 | 29.25 | 2.26 | 29.27 | 2.27 | 2.31 |
| | 30.91 | 0.79 | 30.87 | 0.78 | 30.91 | 0.78 | 1.18 |
| | 30.05 | 1.57 | 30.03 | 1.55 | 30.06 | 1.55 | 1.18 |
| | 30.29 | 1.35 | 30.28 | 1.32 | 30.31 | 1.33 | 1.18 |

| | | | | | | | |
|---|---|---|---|---|---|---|---|
| **6** | 23.19 | 7.80 | 23.18 | 7.78 | 23.22 | 7.79 | 7.82 |
| | 23.53 | 7.50 | 23.53 | 7.46 | 23.57 | 7.47 | 7.55 |
| | 22.78 | 8.18 | 22.76 | 8.17 | 22.80 | 8.17 | 8.27 |
| | 23.37 | 7.64 | 23.35 | 7.63 | 23.40 | 7.62 | 7.67 |
| | 26.27 | 5.01 | 26.23 | 5.01 | 26.26 | 5.02 | 4.56 |
| | 24.86 | 6.29 | 24.77 | 6.34 | 24.83 | 6.32 | 7.58 |
| | 24.10 | 6.98 | 24.06 | 6.99 | 24.10 | 6.99 | 7.69 |
| | 23.80 | 7.25 | 23.79 | 7.23 | 23.82 | 7.24 | 7.66 |
| | 23.62 | 7.42 | 23.53 | 7.46 | 23.60 | 7.44 | 7.96 |
| | 28.06 | 3.38 | 28.03 | 3.37 | 28.06 | 3.38 | 2.58 |
| | 28.24 | 3.22 | 28.19 | 3.23 | 28.23 | 3.22 | 2.71 |
| | 26.56 | 4.74 | 26.52 | 4.74 | 26.54 | 4.76 | 5.49 |
| | 24.45 | 6.66 | 24.43 | 6.64 | 24.47 | 6.64 | 7.23 |
| | 24.55 | 6.57 | 24.54 | 6.55 | 24.58 | 6.55 | 7.09 |
| | 24.06 | 7.01 | 24.02 | 7.02 | 24.06 | 7.02 | 7.27 |
| | 24.34 | 6.76 | 24.28 | 6.78 | 24.32 | 6.78 | 7.48 |
| | 27.95 | 3.48 | 27.90 | 3.48 | 27.92 | 3.51 | 4.25 |
| | 29.48 | 2.09 | 29.42 | 2.10 | 29.46 | 2.11 | 1.73 |
| | 30.02 | 1.60 | 29.98 | 1.59 | 30.01 | 1.60 | 1.51 |
| | 30.24 | 1.40 | 30.09 | 1.49 | 30.14 | 1.48 | 1.84 |
| | 30.26 | 1.38 | 30.26 | 1.34 | 30.29 | 1.34 | 0.95 |
| | 30.28 | 1.36 | 30.26 | 1.34 | 30.29 | 1.35 | 0.95 |
| | 30.64 | 1.04 | 30.64 | 0.99 | 30.67 | 1.00 | 0.95 |
| | 30.03 | 1.59 | 30.01 | 1.56 | 30.05 | 1.56 | 0.90 |
| | 30.94 | 0.76 | 30.93 | 0.73 | 30.96 | 0.73 | 0.90 |
| | 30.23 | 1.41 | 30.18 | 1.41 | 30.22 | 1.41 | 0.90 |
| **7** | 25.20 | 5.98 | 25.14 | 6.00 | 25.18 | 6.00 | 6.17 |
| | 27.21 | 4.15 | 27.12 | 4.20 | 27.18 | 4.17 | 4.49 |
| | 27.64 | 3.76 | 27.61 | 3.75 | 27.65 | 3.75 | 3.09 |
| | 23.55 | 7.48 | 23.49 | 7.50 | 23.54 | 7.50 | 7.34 |
| | 23.61 | 7.42 | 23.59 | 7.41 | 23.64 | 7.41 | 7.22 |
| | 27.06 | 4.29 | 26.99 | 4.32 | 27.03 | 4.32 | 4.72 |
| | 26.75 | 4.57 | 26.69 | 4.58 | 26.73 | 4.59 | 4.72 |
| | 24.39 | 6.71 | 24.36 | 6.71 | 24.40 | 6.71 | 6.75 |
| | 29.01 | 2.52 | 28.96 | 2.52 | 29.00 | 2.52 | 2.93 |
| | 27.49 | 3.90 | 27.43 | 3.92 | 27.47 | 3.91 | 2.93 |
| | 29.35 | 2.20 | 29.33 | 2.19 | 29.37 | 2.18 | 2.93 |
| | 27.54 | 3.86 | 27.45 | 3.90 | 27.50 | 3.89 | 2.62 |
| | 27.82 | 3.60 | 27.73 | 3.64 | 27.79 | 3.62 | 2.62 |
| | 24.39 | 6.71 | 24.37 | 6.70 | 24.41 | 6.70 | 6.90 |
| | 23.22 | 7.78 | 23.20 | 7.77 | 23.24 | 7.77 | 7.29 |
| | 23.90 | 7.16 | 23.88 | 7.14 | 23.92 | 7.15 | 7.23 |
| | 22.83 | 8.14 | 22.83 | 8.10 | 22.87 | 8.10 | 7.64 |
| | 23.78 | 7.27 | 23.78 | 7.24 | 23.82 | 7.24 | 7.19 |
| | 23.87 | 7.18 | 23.89 | 7.13 | 23.93 | 7.14 | 7.19 |
| | 28.86 | 2.65 | 28.82 | 2.65 | 28.86 | 2.64 | 3.14 |
| | 27.32 | 4.05 | 27.25 | 4.07 | 27.31 | 4.06 | 3.14 |
| | 28.93 | 2.59 | 28.91 | 2.57 | 28.95 | 2.57 | 3.14 |
| | 27.45 | 3.93 | 27.41 | 3.93 | 27.46 | 3.93 | 4.72 |
| | 26.93 | 4.41 | 26.89 | 4.41 | 26.91 | 4.42 | 4.72 |

| | | | | | | | |
|---|---|---|---|---|---|---|---|
| | 29.28 | 2.27 | 29.24 | 2.26 | 29.28 | 2.26 | 3.14 |
| | 27.73 | 3.68 | 27.66 | 3.71 | 27.71 | 3.70 | 3.14 |
| | 29.37 | 2.19 | 29.35 | 2.17 | 29.38 | 2.17 | 3.14 |
| **8** | 30.27 | 1.37 | 30.23 | 1.37 | 30.29 | 1.35 | 0.82 |
| | 30.72 | 0.96 | 30.67 | 0.96 | 30.71 | 0.96 | 0.82 |
| | 31.17 | 0.55 | 31.14 | 0.54 | 31.17 | 0.54 | 0.82 |
| | 30.56 | 1.11 | 30.56 | 1.06 | 30.59 | 1.07 | 1.13 |
| | 30.65 | 1.02 | 30.64 | 0.99 | 30.68 | 0.99 | 1.13 |
| | 30.56 | 1.11 | 30.54 | 1.08 | 30.58 | 1.08 | 1.13 |
| | 25.66 | 5.56 | 25.64 | 5.55 | 25.68 | 5.54 | 5.42 |
| | 29.95 | 1.66 | 29.93 | 1.64 | 29.97 | 1.63 | 1.05 |
| | 30.69 | 0.99 | 30.66 | 0.98 | 30.70 | 0.97 | 1.05 |
| | 30.76 | 0.93 | 30.75 | 0.90 | 30.78 | 0.90 | 1.05 |
| | 30.65 | 1.03 | 30.66 | 0.97 | 30.69 | 0.98 | 0.68 |
| | 30.60 | 1.07 | 30.60 | 1.03 | 30.63 | 1.03 | 0.68 |
| | 31.05 | 0.66 | 31.05 | 0.62 | 31.07 | 0.63 | 0.68 |
| **9** | 30.36 | 1.29 | 30.25 | 1.35 | 30.28 | 1.35 | 1.44 |
| | 30.10 | 1.53 | 29.98 | 1.60 | 30.01 | 1.60 | 1.69 |
| | 29.99 | 1.63 | 29.94 | 1.63 | 29.97 | 1.64 | 1.69 |
| | 29.81 | 1.79 | 29.75 | 1.80 | 29.79 | 1.80 | 1.59 |
| | 30.06 | 1.56 | 30.01 | 1.57 | 30.05 | 1.56 | 1.52 |
| | 30.56 | 1.10 | 30.53 | 1.09 | 30.56 | 1.10 | 1.52 |
| | 27.89 | 3.54 | 27.81 | 3.57 | 27.84 | 3.58 | 3.64 |
| | 30.17 | 1.46 | 30.13 | 1.46 | 30.15 | 1.47 | 2.21 |
| | 29.19 | 2.36 | 29.12 | 2.38 | 29.16 | 2.38 | 1.52 |
| | 29.67 | 1.92 | 29.59 | 1.95 | 29.62 | 1.96 | 1.77 |
| | 29.86 | 1.74 | 29.83 | 1.73 | 29.86 | 1.74 | 1.52 |
| | 30.18 | 1.45 | 30.14 | 1.44 | 30.17 | 1.45 | 1.52 |
| | 30.30 | 1.34 | 30.27 | 1.33 | 30.30 | 1.34 | 1.98 |
| | 28.98 | 2.55 | 28.93 | 2.55 | 28.97 | 2.55 | 1.28 |
| | 29.90 | 1.71 | 29.85 | 1.71 | 29.88 | 1.72 | 1.31 |
| | 29.59 | 1.99 | 29.52 | 2.02 | 29.56 | 2.01 | 1.77 |
| | 30.44 | 1.22 | 30.41 | 1.21 | 30.43 | 1.21 | 1.48 |
| | 30.35 | 1.29 | 30.26 | 1.34 | 30.30 | 1.33 | 1.49 |
| | 30.15 | 1.48 | 30.12 | 1.47 | 30.15 | 1.47 | 1.81 |
| | 29.81 | 1.79 | 29.77 | 1.79 | 29.80 | 1.79 | 1.28 |
| | 28.51 | 2.97 | 28.44 | 3.00 | 28.49 | 2.99 | 3.10 |
| | 29.92 | 1.69 | 29.89 | 1.68 | 29.91 | 1.69 | 1.74 |
| | 27.16 | 4.19 | 27.09 | 4.23 | 27.12 | 4.23 | 4.43 |
| | 27.38 | 3.99 | 27.29 | 4.04 | 27.32 | 4.05 | 4.26 |
| | 30.44 | 1.22 | 30.40 | 1.21 | 30.44 | 1.20 | 1.21 |
| | 30.18 | 1.46 | 30.16 | 1.43 | 30.19 | 1.43 | 1.21 |
| | 30.60 | 1.07 | 30.60 | 1.03 | 30.64 | 1.03 | 1.21 |
| | 30.81 | 0.88 | 30.77 | 0.87 | 30.81 | 0.87 | 0.88 |
| | 30.84 | 0.86 | 30.79 | 0.86 | 30.83 | 0.86 | 0.88 |
| | 30.67 | 1.01 | 30.61 | 1.02 | 30.66 | 1.01 | 0.88 |
| | 30.71 | 0.97 | 30.65 | 0.99 | 30.70 | 0.97 | 0.95 |
| | 30.47 | 1.18 | 30.43 | 1.19 | 30.47 | 1.18 | 0.95 |
| | 30.69 | 0.99 | 30.65 | 0.98 | 30.71 | 0.96 | 0.95 |
| | 30.37 | 1.28 | 30.34 | 1.27 | 30.37 | 1.27 | 1.13 |

| | | | | | | | |
|---|---|---|---|---|---|---|---|
| | 31.03 | 0.68 | 31.01 | 0.66 | 31.05 | 0.66 | 1.13 |
| | 30.18 | 1.45 | 30.12 | 1.47 | 30.17 | 1.45 | 1.13 |
| | 30.89 | 0.80 | 30.88 | 0.78 | 30.91 | 0.78 | 1.22 |
| | 30.68 | 1.00 | 30.68 | 0.95 | 30.71 | 0.97 | 1.22 |
| | 29.39 | 2.17 | 29.34 | 2.18 | 29.39 | 2.17 | 1.22 |
| | 29.60 | 1.98 | 29.56 | 1.98 | 29.61 | 1.97 | 0.99 |
| | 30.96 | 0.74 | 30.95 | 0.71 | 30.97 | 0.72 | 0.99 |
| | 30.76 | 0.92 | 30.76 | 0.89 | 30.78 | 0.90 | 0.99 |
| | 30.46 | 1.20 | 30.46 | 1.16 | 30.48 | 1.17 | 0.96 |
| | 31.03 | 0.68 | 31.02 | 0.65 | 31.05 | 0.65 | 0.96 |
| | 30.35 | 1.30 | 30.31 | 1.29 | 30.36 | 1.28 | 0.96 |
| **10** | 25.73 | 5.50 | 25.61 | 5.57 | 25.66 | 5.57 | 5.63 |
| | 28.02 | 3.42 | 27.94 | 3.45 | 28.00 | 3.44 | 3.43 |
| | 28.42 | 3.05 | 28.38 | 3.05 | 28.42 | 3.05 | 3.17 |
| | 29.13 | 2.41 | 29.04 | 2.45 | 29.08 | 2.44 | 2.23 |
| | 30.13 | 1.50 | 30.13 | 1.46 | 30.15 | 1.47 | 1.32 |
| | 30.14 | 1.49 | 30.10 | 1.48 | 30.13 | 1.49 | 1.10 |
| | 25.99 | 5.26 | 25.90 | 5.31 | 25.96 | 5.29 | 5.25 |
| | 26.50 | 4.80 | 26.42 | 4.83 | 26.47 | 4.83 | 4.64 |
| | 29.01 | 2.52 | 28.94 | 2.54 | 28.96 | 2.55 | 2.51 |
| | 29.26 | 2.29 | 29.19 | 2.31 | 29.23 | 2.31 | 1.90 |
| | 28.86 | 2.65 | 28.79 | 2.68 | 28.83 | 2.68 | 2.37 |
| | 27.33 | 4.04 | 27.21 | 4.12 | 27.27 | 4.10 | 4.77 |
| | 26.22 | 5.05 | 26.16 | 5.07 | 26.22 | 5.05 | 4.79 |
| | 28.83 | 2.68 | 28.75 | 2.71 | 28.79 | 2.71 | 2.67 |
| | 29.21 | 2.33 | 29.14 | 2.36 | 29.18 | 2.36 | 2.61 |
| | 30.69 | 0.99 | 30.68 | 0.95 | 30.71 | 0.96 | 0.83 |
| | 30.55 | 1.11 | 30.56 | 1.06 | 30.59 | 1.07 | 0.83 |
| | 30.67 | 1.00 | 30.68 | 0.96 | 30.71 | 0.96 | 0.83 |
| | 28.87 | 2.65 | 28.79 | 2.68 | 28.84 | 2.66 | 2.97 |
| | 29.65 | 1.93 | 29.58 | 1.96 | 29.63 | 1.95 | 2.97 |
| | 29.14 | 2.40 | 29.07 | 2.42 | 29.12 | 2.41 | 2.97 |
| | 30.63 | 1.05 | 30.62 | 1.01 | 30.66 | 1.01 | 1.06 |
| | 30.21 | 1.42 | 30.21 | 1.38 | 30.25 | 1.38 | 1.06 |
| | 30.72 | 0.96 | 30.71 | 0.93 | 30.74 | 0.93 | 1.06 |
| | 30.75 | 0.93 | 30.73 | 0.91 | 30.76 | 0.91 | 1.15 |
| | 30.28 | 1.36 | 30.24 | 1.36 | 30.28 | 1.35 | 1.15 |
| | 31.13 | 0.59 | 31.12 | 0.56 | 31.15 | 0.56 | 1.15 |
| | 31.20 | 0.52 | 31.16 | 0.52 | 31.21 | 0.51 | 1.04 |
| | 31.78 | 0.00 | 31.73 | 0.00 | 31.77 | 0.00 | 1.04 |
| | 32.92 | -1.04 | 32.80 | -0.98 | 32.85 | -0.98 | 1.04 |
| | 30.05 | 1.57 | 29.98 | 1.59 | 30.04 | 1.58 | 1.59 |
| | 29.97 | 1.64 | 29.87 | 1.69 | 29.94 | 1.66 | 1.59 |
| | 30.83 | 0.86 | 30.74 | 0.90 | 30.80 | 0.88 | 1.59 |
| | 23.68 | 7.36 | 23.65 | 7.35 | 23.71 | 7.35 | 7.61 |
| | 23.77 | 7.28 | 23.74 | 7.28 | 23.77 | 7.28 | 7.35 |
| | 23.84 | 7.21 | 23.82 | 7.20 | 23.88 | 7.19 | 6.90 |
| | 23.77 | 7.28 | 23.73 | 7.28 | 23.79 | 7.27 | 6.90 |
| | 24.11 | 6.97 | 24.10 | 6.94 | 24.14 | 6.95 | 7.11 |
| | 24.00 | 7.07 | 23.96 | 7.07 | 24.01 | 7.07 | 7.18 |

| | | | | | | | |
|---|---|---|---|---|---|---|---|
| | 24.32 | 6.78 | 24.31 | 6.75 | 24.35 | 6.76 | 6.69 |
| | 24.25 | 6.84 | 24.23 | 6.83 | 24.28 | 6.83 | 6.69 |
| **11** | 27.40 | 3.98 | 27.39 | 3.95 | 27.42 | 3.96 | 3.78 |
| | 28.96 | 2.56 | 28.91 | 2.57 | 28.95 | 2.56 | 2.97 |
| | 28.57 | 2.91 | 28.55 | 2.90 | 28.59 | 2.89 | 2.77 |
| | 24.74 | 6.40 | 24.68 | 6.42 | 24.72 | 6.42 | 6.45 |
| | 23.51 | 7.51 | 23.48 | 7.51 | 23.54 | 7.50 | 7.35 |
| | 24.15 | 6.94 | 24.10 | 6.94 | 24.17 | 6.92 | 6.97 |
| | 24.23 | 6.86 | 24.19 | 6.87 | 24.24 | 6.85 | 6.84 |
| | 23.71 | 7.33 | 23.70 | 7.31 | 23.75 | 7.30 | 7.30 |
| | 24.48 | 6.64 | 24.41 | 6.66 | 24.47 | 6.65 | 6.73 |
| | 24.37 | 6.73 | 24.35 | 6.72 | 24.40 | 6.71 | 6.84 |
| | 24.97 | 6.19 | 24.92 | 6.20 | 24.96 | 6.20 | 6.44 |
| | 29.76 | 1.84 | 29.69 | 1.85 | 29.73 | 1.86 | 2.10 |
| | 29.13 | 2.40 | 29.07 | 2.42 | 29.11 | 2.42 | 2.75 |
| | 28.51 | 2.97 | 28.47 | 2.96 | 28.51 | 2.96 | 3.65 |
| | 27.36 | 4.02 | 27.29 | 4.04 | 27.32 | 4.05 | 4.76 |
| | 30.87 | 0.82 | 30.86 | 0.79 | 30.89 | 0.80 | 1.45 |
| | 30.53 | 1.13 | 30.49 | 1.13 | 30.53 | 1.13 | 1.45 |
| | 30.44 | 1.21 | 30.45 | 1.16 | 30.48 | 1.17 | 1.45 |
| | 28.63 | 2.86 | 28.62 | 2.83 | 28.65 | 2.84 | 2.44 |
| | 29.39 | 2.17 | 29.32 | 2.20 | 29.37 | 2.19 | 2.44 |
| | 29.57 | 2.00 | 29.53 | 2.00 | 29.58 | 1.99 | 2.44 |
| | 28.71 | 2.78 | 28.65 | 2.81 | 28.69 | 2.80 | 3.28 |
| | 27.99 | 3.44 | 27.96 | 3.43 | 28.01 | 3.42 | 3.28 |
| | 29.14 | 2.39 | 29.07 | 2.43 | 29.12 | 2.41 | 3.28 |
| | 29.42 | 2.14 | 29.39 | 2.13 | 29.43 | 2.12 | 2.38 |
| | 28.86 | 2.65 | 28.86 | 2.61 | 28.90 | 2.61 | 2.38 |
| | 28.82 | 2.69 | 28.81 | 2.66 | 28.86 | 2.65 | 2.38 |
| | 27.43 | 3.95 | 27.41 | 3.93 | 27.46 | 3.93 | 4.09 |
| | 27.50 | 3.89 | 27.48 | 3.87 | 27.52 | 3.87 | 4.09 |
| | 26.99 | 4.35 | 27.00 | 4.31 | 27.04 | 4.31 | 4.09 |
| | 27.16 | 4.20 | 27.17 | 4.16 | 27.21 | 4.15 | 4.08 |
| | 27.63 | 3.77 | 27.61 | 3.75 | 27.66 | 3.75 | 4.08 |
| | 27.71 | 3.70 | 27.69 | 3.67 | 27.73 | 3.68 | 4.08 |
| | 28.89 | 2.62 | 28.88 | 2.59 | 28.93 | 2.58 | 2.33 |
| | 29.51 | 2.06 | 29.48 | 2.05 | 29.52 | 2.04 | 2.33 |
| | 28.88 | 2.64 | 28.87 | 2.60 | 28.91 | 2.60 | 2.33 |
| | 30.24 | 1.40 | 30.22 | 1.37 | 30.26 | 1.37 | 1.62 |
| | 30.19 | 1.45 | 30.13 | 1.46 | 30.18 | 1.45 | 1.62 |
| | 30.04 | 1.58 | 30.02 | 1.56 | 30.06 | 1.55 | 1.62 |
| | 31.09 | 0.63 | 31.07 | 0.60 | 31.11 | 0.60 | 1.10 |
| | 30.45 | 1.21 | 30.45 | 1.17 | 30.48 | 1.17 | 1.10 |
| | 30.69 | 0.99 | 30.67 | 0.97 | 30.69 | 0.98 | 1.10 |
| **12** | 29.11 | 2.43 | 28.96 | 2.52 | 29.01 | 2.51 | 2.56 |
| | 28.12 | 3.32 | 28.03 | 3.37 | 28.07 | 3.36 | 4.04 |
| | 29.33 | 2.22 | 29.21 | 2.29 | 29.26 | 2.29 | 2.23 |
| | 29.18 | 2.36 | 29.08 | 2.41 | 29.13 | 2.41 | 2.47 |
| | 28.46 | 3.01 | 28.39 | 3.04 | 28.42 | 3.05 | 2.85 |
| | 30.22 | 1.42 | 30.16 | 1.43 | 30.20 | 1.43 | 1.07 |

| | | | | | | | |
|---|---|---|---|---|---|---|---|
| | 30.71 | 0.97 | 30.64 | 0.99 | 30.69 | 0.98 | 1.07 |
| | 31.06 | 0.65 | 30.98 | 0.69 | 31.02 | 0.68 | 1.07 |
| | 26.62 | 4.69 | 26.55 | 4.72 | 26.58 | 4.72 | 4.76 |
| | 27.10 | 4.25 | 27.04 | 4.27 | 27.08 | 4.27 | 4.46 |
| | 30.62 | 1.06 | 30.59 | 1.04 | 30.62 | 1.05 | 1.17 |
| | 30.19 | 1.44 | 30.15 | 1.44 | 30.19 | 1.44 | 1.17 |
| | 30.43 | 1.22 | 30.35 | 1.26 | 30.40 | 1.24 | 1.17 |
| | 28.74 | 2.76 | 28.67 | 2.78 | 28.71 | 2.79 | 2.98 |
| | 28.88 | 2.64 | 28.82 | 2.65 | 28.84 | 2.66 | 2.68 |
| | 29.95 | 1.66 | 29.93 | 1.64 | 29.98 | 1.62 | 1.18 |
| | 30.50 | 1.16 | 30.48 | 1.14 | 30.52 | 1.14 | 1.18 |
| | 30.83 | 0.86 | 30.80 | 0.85 | 30.83 | 0.85 | 1.18 |
| | 30.69 | 0.99 | 30.68 | 0.95 | 30.71 | 0.96 | 1.29 |
| | 30.24 | 1.40 | 30.22 | 1.38 | 30.25 | 1.38 | 1.29 |
| | 30.20 | 1.44 | 30.18 | 1.41 | 30.21 | 1.42 | 1.29 |
| | 24.64 | 6.49 | 24.62 | 6.47 | 24.67 | 6.47 | 6.34 |
| | 23.89 | 7.17 | 23.85 | 7.17 | 23.89 | 7.17 | 7.41 |
| | 23.81 | 7.24 | 23.81 | 7.21 | 23.84 | 7.22 | 7.40 |
| **13** | 27.63 | 3.77 | 27.60 | 3.76 | 27.63 | 3.77 | 4.11 |
| | 28.55 | 2.93 | 28.49 | 2.95 | 28.52 | 2.96 | 1.84 |
| | 28.10 | 3.34 | 28.08 | 3.33 | 28.11 | 3.33 | 3.14 |
| | 28.14 | 3.31 | 28.05 | 3.35 | 28.10 | 3.34 | 2.98 |
| | 29.58 | 2.00 | 29.58 | 1.96 | 29.61 | 1.97 | 2.16 |
| | 28.72 | 2.78 | 28.67 | 2.78 | 28.72 | 2.77 | 2.16 |
| | 29.35 | 2.20 | 29.30 | 2.21 | 29.34 | 2.21 | 2.48 |
| | 29.71 | 1.88 | 29.67 | 1.88 | 29.72 | 1.86 | 2.48 |
| | 28.09 | 3.35 | 28.10 | 3.31 | 28.12 | 3.33 | 3.12 |
| | 28.49 | 2.99 | 28.47 | 2.96 | 28.51 | 2.96 | 3.12 |
| | 27.62 | 3.78 | 27.61 | 3.75 | 27.64 | 3.76 | 3.55 |
| | 28.70 | 2.80 | 28.65 | 2.81 | 28.68 | 2.81 | 3.55 |
| | 23.92 | 7.14 | 23.91 | 7.12 | 23.95 | 7.12 | 7.14 |
| | 24.23 | 6.86 | 24.18 | 6.87 | 24.22 | 6.87 | 6.20 |
| | 25.77 | 5.46 | 25.74 | 5.46 | 25.78 | 5.45 | 5.35 |
| | 24.34 | 6.76 | 24.34 | 6.73 | 24.37 | 6.74 | 6.66 |
| | 24.09 | 6.99 | 24.08 | 6.96 | 24.12 | 6.97 | 7.12 |
| | 29.32 | 2.24 | 29.23 | 2.28 | 29.29 | 2.26 | 1.83 |
| | 29.77 | 1.82 | 29.77 | 1.78 | 29.81 | 1.78 | 1.83 |
| | 29.70 | 1.89 | 29.67 | 1.87 | 29.71 | 1.87 | 1.83 |
| | 26.03 | 5.22 | 26.00 | 5.22 | 26.03 | 5.23 | 5.30 |
| | 29.35 | 2.20 | 29.30 | 2.21 | 29.34 | 2.21 | 2.48 |
| | 29.71 | 1.88 | 29.67 | 1.88 | 29.72 | 1.86 | 2.48 |
| | 28.09 | 3.35 | 28.10 | 3.31 | 28.12 | 3.33 | 2.90 |
| | 28.49 | 2.99 | 28.47 | 2.96 | 28.51 | 2.96 | 2.90 |
| | 27.62 | 3.78 | 27.61 | 3.75 | 27.64 | 3.76 | 3.13 |
| | 28.70 | 2.80 | 28.65 | 2.81 | 28.68 | 2.81 | 3.13 |
| **14** | 28.74 | 2.76 | 28.64 | 2.82 | 28.68 | 2.81 | 2.99 |
| | 28.37 | 3.09 | 28.29 | 3.13 | 28.34 | 3.12 | 3.36 |
| | 27.55 | 3.85 | 27.47 | 3.88 | 27.50 | 3.89 | 3.50 |
| | 28.62 | 2.87 | 28.59 | 2.86 | 28.63 | 2.85 | 2.72 |
| | 29.39 | 2.17 | 29.37 | 2.15 | 29.40 | 2.15 | 2.04 |

| | | | | | | | |
|---|---|---|---|---|---|---|---|
| | 25.02 | 6.14 | 24.97 | 6.16 | 25.01 | 6.15 | 6.38 |
| | 23.84 | 7.21 | 23.83 | 7.19 | 23.88 | 7.19 | 7.14 |
| | 29.50 | 2.07 | 29.50 | 2.03 | 29.54 | 2.03 | 1.78 |
| | 29.57 | 2.01 | 29.54 | 1.99 | 29.59 | 1.98 | 1.78 |
| | 29.64 | 1.94 | 29.63 | 1.91 | 29.67 | 1.91 | 1.78 |
| | 24.20 | 6.88 | 24.15 | 6.90 | 24.20 | 6.90 | 6.94 |
| | 24.76 | 6.38 | 24.74 | 6.36 | 24.79 | 6.36 | 6.42 |
| | 23.65 | 7.39 | 23.60 | 7.40 | 23.65 | 7.39 | 7.24 |
| | 24.14 | 6.94 | 24.08 | 6.96 | 24.13 | 6.96 | 6.98 |
| | 24.76 | 6.38 | 24.74 | 6.37 | 24.77 | 6.37 | 6.51 |
| | 25.33 | 5.86 | 25.25 | 5.89 | 25.30 | 5.89 | 6.23 |
| | 24.81 | 6.34 | 24.77 | 6.33 | 24.81 | 6.34 | 6.46 |
| | 24.13 | 6.95 | 24.12 | 6.93 | 24.16 | 6.93 | 7.05 |
| | 23.64 | 7.39 | 23.65 | 7.36 | 23.69 | 7.36 | 7.41 |
| | 24.32 | 6.77 | 24.29 | 6.78 | 24.33 | 6.78 | 6.97 |
| | 24.28 | 6.82 | 24.28 | 6.78 | 24.33 | 6.78 | 6.81 |
| **15** | 28.72 | 2.78 | 28.62 | 2.83 | 28.66 | 2.83 | 2.23 |
| | 26.71 | 4.61 | 26.66 | 4.62 | 26.68 | 4.63 | 5.41 |
| | 27.57 | 3.82 | 27.46 | 3.89 | 27.49 | 3.89 | 3.58 |
| | 26.06 | 5.20 | 25.97 | 5.25 | 26.01 | 5.25 | 5.68 |
| | 30.29 | 1.35 | 30.23 | 1.37 | 30.27 | 1.36 | 1.02 |
| | 30.70 | 0.98 | 30.67 | 0.97 | 30.70 | 0.97 | 1.02 |
| | 30.30 | 1.34 | 30.28 | 1.32 | 30.32 | 1.32 | 1.02 |
| | 23.30 | 7.70 | 23.27 | 7.70 | 23.32 | 7.70 | 8.01 |
| | 23.55 | 7.48 | 23.53 | 7.47 | 23.57 | 7.47 | 7.42 |
| | 23.57 | 7.46 | 23.54 | 7.45 | 23.58 | 7.46 | 7.54 |
| | 23.92 | 7.14 | 23.89 | 7.14 | 23.93 | 7.14 | 7.42 |
| | 23.82 | 7.23 | 23.75 | 7.26 | 23.81 | 7.25 | 8.01 |
| | 25.56 | 5.65 | 25.46 | 5.71 | 25.50 | 5.71 | 6.36 |
| | 29.54 | 2.04 | 29.49 | 2.04 | 29.54 | 2.03 | 1.68 |
| | 29.74 | 1.85 | 29.70 | 1.85 | 29.75 | 1.84 | 1.68 |
| | 29.88 | 1.73 | 29.86 | 1.71 | 29.90 | 1.70 | 1.68 |
| | 25.27 | 5.91 | 25.21 | 5.94 | 25.27 | 5.92 | 5.39 |
| | 29.69 | 1.90 | 29.60 | 1.94 | 29.63 | 1.94 | 1.99 |
| | 29.08 | 2.45 | 28.98 | 2.50 | 29.04 | 2.49 | 2.37 |
| | 26.98 | 4.36 | 26.87 | 4.43 | 26.90 | 4.43 | 5.48 |
| | 29.84 | 1.76 | 29.80 | 1.76 | 29.83 | 1.76 | 0.97 |
| | 30.71 | 0.97 | 30.67 | 0.97 | 30.70 | 0.97 | 0.97 |
| | 30.94 | 0.76 | 30.92 | 0.74 | 30.95 | 0.74 | 0.97 |
| | 30.56 | 1.11 | 30.54 | 1.08 | 30.57 | 1.09 | 1.02 |
| | 30.65 | 1.02 | 30.63 | 1.00 | 30.66 | 1.01 | 1.02 |
| | 30.38 | 1.27 | 30.35 | 1.26 | 30.38 | 1.26 | 1.02 |
| | 26.49 | 4.80 | 26.37 | 4.88 | 26.41 | 4.88 | 5.39 |
| | 24.35 | 6.75 | 24.33 | 6.74 | 24.38 | 6.73 | 6.75 |
| | 28.68 | 2.81 | 28.63 | 2.82 | 28.68 | 2.81 | 1.73 |
| | 29.57 | 2.01 | 29.52 | 2.02 | 29.57 | 2.00 | 1.73 |
| | 29.63 | 1.95 | 29.58 | 1.95 | 29.63 | 1.94 | 1.73 |
| | 29.59 | 1.99 | 29.54 | 2.00 | 29.59 | 1.98 | 2.05 |
| | 28.84 | 2.67 | 28.74 | 2.72 | 28.79 | 2.71 | 2.05 |
| | 29.68 | 1.90 | 29.59 | 1.95 | 29.64 | 1.93 | 2.05 |

| | | | | | | | |
|---|---|---|---|---|---|---|---|
| | 29.62 | 1.96 | 29.59 | 1.94 | 29.64 | 1.94 | 2.27 |
| | 29.04 | 2.48 | 29.01 | 2.48 | 29.05 | 2.48 | 2.27 |
| | 29.20 | 2.34 | 29.14 | 2.36 | 29.18 | 2.36 | 2.27 |
| | 28.97 | 2.55 | 28.92 | 2.56 | 28.97 | 2.55 | 2.18 |
| | 29.58 | 2.00 | 29.48 | 2.05 | 29.54 | 2.03 | 2.18 |
| | 29.81 | 1.78 | 29.77 | 1.78 | 29.82 | 1.77 | 2.18 |
| | 29.41 | 2.15 | 29.36 | 2.16 | 29.41 | 2.14 | 2.02 |
| | 28.89 | 2.63 | 28.82 | 2.65 | 28.87 | 2.64 | 2.02 |
| | 29.34 | 2.22 | 29.27 | 2.24 | 29.31 | 2.24 | 2.02 |
| | 29.37 | 2.19 | 29.30 | 2.21 | 29.35 | 2.20 | 2.00 |
| | 29.28 | 2.27 | 29.18 | 2.33 | 29.23 | 2.31 | 2.00 |
| | 29.19 | 2.35 | 29.14 | 2.36 | 29.18 | 2.36 | 2.00 |
| **16** | 27.67 | 3.73 | 27.66 | 3.70 | 27.69 | 3.72 | 3.75 |
| | 25.76 | 5.47 | 25.70 | 5.49 | 25.76 | 5.48 | 5.21 |
| | 26.23 | 5.04 | 26.15 | 5.08 | 26.20 | 5.07 | 5.04 |
| | 26.72 | 4.60 | 26.68 | 4.60 | 26.72 | 4.60 | 4.57 |
| | 26.21 | 5.06 | 26.14 | 5.09 | 26.17 | 5.10 | 5.03 |
| | 25.76 | 5.47 | 25.69 | 5.50 | 25.74 | 5.49 | 5.44 |
| | 24.33 | 6.77 | 24.27 | 6.79 | 24.33 | 6.78 | 6.62 |
| | 24.80 | 6.34 | 24.73 | 6.38 | 24.78 | 6.36 | 6.30 |
| | 24.43 | 6.68 | 24.38 | 6.69 | 24.43 | 6.68 | 6.58 |
| | 24.46 | 6.65 | 24.39 | 6.68 | 24.45 | 6.67 | 6.49 |
| **17** | 29.70 | 1.89 | 29.64 | 1.90 | 29.68 | 1.90 | 1.77 |
| | 27.65 | 3.75 | 27.64 | 3.73 | 27.65 | 3.75 | 3.46 |
| | 28.09 | 3.35 | 28.03 | 3.36 | 28.06 | 3.38 | 3.39 |
| | 25.76 | 5.47 | 25.72 | 5.48 | 25.74 | 5.49 | 5.59 |
| | 27.92 | 3.51 | 27.87 | 3.51 | 27.90 | 3.53 | 4.08 |
| | 27.78 | 3.63 | 27.72 | 3.65 | 27.76 | 3.65 | 3.62 |
| | 26.94 | 4.40 | 26.87 | 4.42 | 26.91 | 4.43 | 4.18 |
| | 27.86 | 3.56 | 27.82 | 3.56 | 27.84 | 3.57 | 2.76 |
| | 28.11 | 3.33 | 28.07 | 3.33 | 28.10 | 3.34 | 3.25 |
| | 27.94 | 3.48 | 27.92 | 3.47 | 27.94 | 3.48 | 3.23 |
| | 28.23 | 3.22 | 28.19 | 3.22 | 28.21 | 3.24 | 3.11 |
| | 28.30 | 3.16 | 28.24 | 3.18 | 28.27 | 3.19 | 2.94 |
| | 29.46 | 2.11 | 29.43 | 2.10 | 29.46 | 2.10 | 2.04 |
| | 27.52 | 3.87 | 27.48 | 3.87 | 27.50 | 3.88 | 3.93 |
| | 27.22 | 4.14 | 27.18 | 4.14 | 27.21 | 4.15 | 4.29 |
| | 29.33 | 2.23 | 29.28 | 2.23 | 29.32 | 2.23 | 2.36 |
| | 29.35 | 2.21 | 29.32 | 2.20 | 29.34 | 2.21 | 1.90 |
| | 28.62 | 2.87 | 28.57 | 2.88 | 28.61 | 2.87 | 2.28 |
| | 29.71 | 1.88 | 29.68 | 1.86 | 29.72 | 1.86 | 2.01 |
| | 26.34 | 4.94 | 26.27 | 4.97 | 26.32 | 4.96 | 5.36 |
| | 29.12 | 2.41 | 29.08 | 2.41 | 29.11 | 2.42 | 2.18 |
| | 27.29 | 4.07 | 27.29 | 4.04 | 27.32 | 4.05 | 4.03 |
| | 27.66 | 3.74 | 27.65 | 3.72 | 27.67 | 3.73 | 3.58 |
| | 30.56 | 1.11 | 30.56 | 1.07 | 30.58 | 1.08 | 0.89 |
| | 29.74 | 1.85 | 29.71 | 1.84 | 29.75 | 1.84 | 0.89 |
| | 30.01 | 1.61 | 30.00 | 1.57 | 30.04 | 1.58 | 0.89 |
| | 22.83 | 8.13 | 22.77 | 8.16 | 22.82 | 8.15 | 8.16 |
| | 23.50 | 7.52 | 23.49 | 7.50 | 23.53 | 7.50 | 7.57 |

| | | | | | | | |
|---|---|---|---|---|---|---|---|
| | 23.38 | 7.64 | 23.36 | 7.62 | 23.41 | 7.61 | 7.66 |
| | 23.54 | 7.49 | 23.53 | 7.47 | 23.57 | 7.47 | 7.57 |
| | 22.80 | 8.15 | 22.78 | 8.15 | 22.82 | 8.15 | 8.16 |
| | 27.27 | 4.09 | 27.25 | 4.08 | 27.28 | 4.09 | 3.62 |
| | 27.73 | 3.68 | 27.72 | 3.66 | 27.74 | 3.67 | 3.56 |
| | 27.60 | 3.80 | 27.59 | 3.77 | 27.62 | 3.78 | 3.75 |
| | 27.36 | 4.01 | 27.35 | 3.99 | 27.38 | 4.00 | 3.89 |
| **18** | 24.08 | 6.99 | 24.07 | 6.97 | 24.11 | 6.97 | 6.62 |
| | 29.23 | 2.31 | 29.20 | 2.31 | 29.23 | 2.31 | 1.98 |
| | 27.76 | 3.65 | 27.74 | 3.63 | 27.77 | 3.64 | 3.11 |
| | 25.14 | 6.03 | 25.11 | 6.02 | 25.15 | 6.03 | 5.70 |
| | 27.68 | 3.72 | 27.64 | 3.72 | 27.66 | 3.74 | 3.49 |
| | 28.92 | 2.59 | 28.86 | 2.61 | 28.89 | 2.62 | 2.20 |
| | 28.36 | 3.11 | 28.21 | 3.21 | 28.26 | 3.20 | 3.11 |
| | 29.24 | 2.31 | 29.13 | 2.37 | 29.17 | 2.36 | 2.77 |
| | 29.24 | 2.31 | 29.17 | 2.33 | 29.21 | 2.33 | 2.11 |
| | 30.01 | 1.60 | 29.94 | 1.63 | 30.00 | 1.61 | 0.94 |
| | 28.55 | 2.93 | 28.46 | 2.98 | 28.49 | 2.99 | 2.87 |
| | 27.29 | 4.08 | 27.26 | 4.07 | 27.30 | 4.07 | 3.58 |
| | 27.67 | 3.73 | 27.62 | 3.74 | 27.66 | 3.74 | 4.25 |
| | 29.68 | 1.90 | 29.65 | 1.89 | 29.69 | 1.90 | 1.93 |
| | 29.55 | 2.03 | 29.48 | 2.05 | 29.52 | 2.05 | 2.10 |
| | 26.95 | 4.39 | 26.92 | 4.38 | 26.94 | 4.40 | 4.56 |
| | 30.06 | 1.56 | 30.05 | 1.53 | 30.07 | 1.54 | 1.81 |
| | 29.70 | 1.89 | 29.65 | 1.89 | 29.70 | 1.88 | 1.81 |
| | 29.05 | 2.48 | 29.02 | 2.47 | 29.06 | 2.46 | 1.81 |
| | 30.48 | 1.18 | 30.49 | 1.13 | 30.52 | 1.13 | 1.17 |
| | 30.03 | 1.59 | 30.01 | 1.57 | 30.04 | 1.57 | 1.17 |
| | 30.47 | 1.19 | 30.45 | 1.17 | 30.48 | 1.17 | 1.17 |
| | 29.67 | 1.91 | 29.65 | 1.90 | 29.68 | 1.90 | 1.11 |
| | 30.54 | 1.12 | 30.49 | 1.13 | 30.53 | 1.12 | 1.11 |
| | 30.31 | 1.34 | 30.26 | 1.34 | 30.30 | 1.34 | 1.11 |
| **19** | 25.20 | 5.98 | 25.14 | 6.00 | 25.18 | 6.00 | 5.97 |
| | 28.86 | 2.65 | 28.79 | 2.67 | 28.83 | 2.67 | 2.91 |
| | 28.74 | 2.76 | 28.69 | 2.77 | 28.73 | 2.77 | 2.74 |
| | 26.73 | 4.59 | 26.67 | 4.61 | 26.70 | 4.61 | 4.97 |
| | 27.52 | 3.87 | 27.42 | 3.92 | 27.48 | 3.90 | 4.74 |
| | 25.10 | 6.06 | 25.06 | 6.07 | 25.10 | 6.08 | 6.00 |
| | 26.24 | 5.03 | 26.22 | 5.01 | 26.25 | 5.03 | 5.11 |
| | 24.28 | 6.82 | 24.26 | 6.80 | 24.32 | 6.78 | 6.99 |
| | 24.49 | 6.63 | 24.44 | 6.64 | 24.49 | 6.63 | 6.99 |
| | 24.48 | 6.63 | 24.45 | 6.63 | 24.50 | 6.62 | 6.73 |
| | 24.55 | 6.57 | 24.51 | 6.58 | 24.55 | 6.57 | 6.71 |
| | 26.07 | 5.19 | 25.92 | 5.29 | 25.97 | 5.28 | 6.99 |
| | 24.03 | 7.04 | 24.01 | 7.03 | 24.06 | 7.02 | 6.99 |
| | 26.77 | 4.56 | 26.73 | 4.55 | 26.77 | 4.56 | 4.31 |
| | 25.21 | 5.96 | 25.17 | 5.97 | 25.22 | 5.97 | 6.01 |
| | 27.24 | 4.12 | 27.21 | 4.12 | 27.25 | 4.12 | 4.02 |
| **20** | 27.55 | 3.84 | 27.52 | 3.84 | 27.54 | 3.85 | 3.96 |
| | 29.20 | 2.35 | 29.17 | 2.33 | 29.21 | 2.33 | 2.35 |

| | | | | | | | |
|---|---|---|---|---|---|---|---|
| | 30.19 | 1.44 | 30.19 | 1.40 | 30.22 | 1.41 | 1.44 |
| | 28.30 | 3.16 | 28.24 | 3.18 | 28.27 | 3.18 | 3.13 |
| | 30.54 | 1.13 | 30.45 | 1.17 | 30.49 | 1.16 | 1.26 |
| | 27.61 | 3.78 | 27.53 | 3.82 | 27.57 | 3.82 | 3.85 |
| | 22.85 | 8.12 | 22.81 | 8.12 | 22.85 | 8.13 | 8.08 |
| | 23.80 | 7.25 | 23.78 | 7.24 | 23.82 | 7.24 | 7.24 |
| | 23.97 | 7.10 | 23.96 | 7.07 | 23.99 | 7.08 | 7.08 |
| | 23.83 | 7.23 | 23.81 | 7.21 | 23.86 | 7.21 | 7.16 |
| | 29.60 | 1.98 | 29.61 | 1.93 | 29.64 | 1.94 | 1.89 |
| | 29.71 | 1.88 | 29.69 | 1.86 | 29.72 | 1.86 | 1.87 |
| | 28.48 | 3.00 | 28.46 | 2.98 | 28.49 | 2.98 | 3.21 |
| | 28.52 | 2.96 | 28.45 | 2.98 | 28.49 | 2.98 | 2.87 |
| | 28.80 | 2.71 | 28.78 | 2.69 | 28.82 | 2.69 | 2.74 |
| | 27.77 | 3.65 | 27.76 | 3.61 | 27.79 | 3.63 | 3.71 |
| | 25.06 | 6.11 | 25.03 | 6.10 | 25.08 | 6.09 | 5.92 |
| | 27.30 | 4.07 | 27.29 | 4.04 | 27.32 | 4.05 | 4.05 |
| | 27.36 | 4.01 | 27.38 | 3.96 | 27.42 | 3.96 | 4.14 |
| | 27.26 | 4.10 | 27.22 | 4.11 | 27.25 | 4.12 | 4.27 |
| | 28.52 | 2.96 | 28.46 | 2.98 | 28.49 | 2.98 | 3.11 |
| | 28.89 | 2.62 | 28.87 | 2.61 | 28.89 | 2.62 | 2.66 |
| **21** | 22.79 | 8.17 | 22.77 | 8.16 | 22.80 | 8.17 | 8.23 |
| | 29.56 | 2.01 | 29.52 | 2.01 | 29.56 | 2.01 | 1.72 |
| | 28.08 | 3.36 | 28.04 | 3.36 | 28.08 | 3.36 | 3.24 |
| | 27.97 | 3.46 | 27.97 | 3.42 | 28.00 | 3.44 | 3.18 |
| | 29.51 | 2.06 | 29.46 | 2.07 | 29.51 | 2.05 | 2.46 |
| | 27.59 | 3.81 | 27.59 | 3.77 | 27.61 | 3.79 | 3.62 |
| | 23.72 | 7.33 | 23.71 | 7.30 | 23.75 | 7.30 | 7.26 |
| | 23.97 | 7.09 | 23.96 | 7.07 | 24.00 | 7.08 | 7.18 |
| | 23.88 | 7.18 | 23.86 | 7.16 | 23.90 | 7.17 | 7.29 |
| | 28.95 | 2.57 | 28.86 | 2.61 | 28.89 | 2.62 | 2.88 |
| | 27.59 | 3.81 | 27.49 | 3.86 | 27.54 | 3.85 | 3.86 |
| | 28.91 | 2.61 | 28.84 | 2.63 | 28.91 | 2.61 | 1.78 |
| | 30.28 | 1.36 | 30.28 | 1.32 | 30.32 | 1.32 | 1.78 |
| | 27.11 | 4.24 | 27.06 | 4.26 | 27.09 | 4.26 | 4.45 |
| | 28.08 | 3.36 | 27.99 | 3.40 | 28.03 | 3.40 | 2.74 |
| | 28.62 | 2.87 | 28.60 | 2.85 | 28.63 | 2.86 | 3.36 |
| | 25.80 | 5.43 | 25.78 | 5.42 | 25.82 | 5.41 | 5.43 |
| | 29.21 | 2.33 | 29.15 | 2.35 | 29.19 | 2.35 | 2.14 |
| | 28.39 | 3.08 | 28.39 | 3.04 | 28.42 | 3.04 | 3.03 |
| | 29.32 | 2.24 | 29.28 | 2.23 | 29.32 | 2.23 | 1.73 |
| | 28.52 | 2.96 | 28.43 | 3.00 | 28.46 | 3.01 | 2.57 |
| | 29.95 | 1.67 | 29.92 | 1.64 | 29.96 | 1.64 | 1.78 |
| | 27.20 | 4.16 | 27.17 | 4.15 | 27.19 | 4.17 | 4.62 |
| | 29.26 | 2.29 | 29.21 | 2.29 | 29.27 | 2.28 | 1.72 |
| | 29.91 | 1.70 | 29.91 | 1.66 | 29.94 | 1.66 | 1.72 |
| | 29.99 | 1.62 | 29.99 | 1.58 | 30.03 | 1.58 | 1.72 |
| | 25.60 | 5.61 | 25.57 | 5.61 | 25.61 | 5.61 | 5.62 |
| | 27.89 | 3.53 | 27.87 | 3.52 | 27.90 | 3.52 | 3.59 |
| | 28.34 | 3.12 | 28.31 | 3.12 | 28.34 | 3.12 | 2.91 |
| | 29.84 | 1.76 | 29.81 | 1.75 | 29.84 | 1.75 | 1.62 |

| | | | | | | | |
|---|---|---|---|---|---|---|---|
| | 29.61 | 1.97 | 29.57 | 1.97 | 29.60 | 1.97 | 2.04 |
| | 28.42 | 3.05 | 28.36 | 3.07 | 28.40 | 3.06 | 3.03 |
| | 29.88 | 1.73 | 29.82 | 1.74 | 29.85 | 1.74 | 1.61 |
| | 28.00 | 3.44 | 27.94 | 3.45 | 27.97 | 3.45 | 3.06 |
| | 24.58 | 6.54 | 24.53 | 6.56 | 24.58 | 6.55 | 6.52 |
| | 24.03 | 7.04 | 24.02 | 7.02 | 24.06 | 7.02 | 7.17 |
| | 24.37 | 6.74 | 24.37 | 6.70 | 24.40 | 6.71 | 6.85 |
| | 24.01 | 7.06 | 23.98 | 7.06 | 24.02 | 7.05 | 7.09 |
| | 28.40 | 3.07 | 28.42 | 3.02 | 28.44 | 3.03 | 3.18 |
| | 28.73 | 2.77 | 28.75 | 2.71 | 28.79 | 2.71 | 2.71 |
| | 28.05 | 3.39 | 28.00 | 3.39 | 28.03 | 3.41 | 3.51 |
| **22** | 29.40 | 2.16 | 29.38 | 2.14 | 29.43 | 2.13 | 1.52 |
| | 30.06 | 1.56 | 30.01 | 1.57 | 30.05 | 1.56 | 1.52 |
| | 30.00 | 1.62 | 29.98 | 1.59 | 30.02 | 1.59 | 1.52 |
| | 29.64 | 1.95 | 29.61 | 1.93 | 29.65 | 1.93 | 1.52 |
| | 30.42 | 1.24 | 30.40 | 1.21 | 30.43 | 1.22 | 1.52 |
| | 30.01 | 1.61 | 30.01 | 1.57 | 30.04 | 1.57 | 1.52 |
| | 24.72 | 6.41 | 24.67 | 6.42 | 24.73 | 6.42 | 6.16 |
| | 23.79 | 7.26 | 23.80 | 7.22 | 23.84 | 7.22 | 7.21 |
| | 23.68 | 7.36 | 23.65 | 7.36 | 23.68 | 7.37 | 7.32 |
| | 28.33 | 3.13 | 28.22 | 3.19 | 28.29 | 3.16 | 3.27 |
| | 28.26 | 3.20 | 28.21 | 3.21 | 28.25 | 3.20 | 3.35 |
| | 23.97 | 7.10 | 23.98 | 7.06 | 24.02 | 7.06 | 6.93 |
| | 23.87 | 7.18 | 23.87 | 7.15 | 23.92 | 7.15 | 7.03 |
| | 23.99 | 7.08 | 23.94 | 7.09 | 23.98 | 7.10 | 7.48 |
| | 25.85 | 5.38 | 25.82 | 5.38 | 25.87 | 5.37 | 5.10 |
| | 25.87 | 5.37 | 25.82 | 5.38 | 25.87 | 5.37 | 4.99 |
| | 23.88 | 7.18 | 23.84 | 7.18 | 23.90 | 7.17 | 7.39 |
| | 23.63 | 7.40 | 23.60 | 7.40 | 23.64 | 7.41 | 7.49 |
| | 23.99 | 7.08 | 23.95 | 7.08 | 24.00 | 7.08 | 7.10 |
| | 23.84 | 7.22 | 23.80 | 7.22 | 23.84 | 7.22 | 7.19 |
| | 30.18 | 1.45 | 30.15 | 1.44 | 30.19 | 1.43 | 1.57 |
| | 29.94 | 1.67 | 29.92 | 1.65 | 29.96 | 1.64 | 1.57 |
| | 30.19 | 1.45 | 30.18 | 1.41 | 30.21 | 1.42 | 1.57 |
| | 31.48 | 0.27 | 31.39 | 0.31 | 31.45 | 0.29 | 1.29 |
| | 31.22 | 0.51 | 31.19 | 0.50 | 31.23 | 0.49 | 1.29 |
| | 33.17 | -1.27 | 33.04 | -1.19 | 33.09 | -1.21 | 1.29 |
| **23** | 27.20 | 4.16 | 27.19 | 4.13 | 27.23 | 4.13 | 4.62 |
| | 28.48 | 3.00 | 28.41 | 3.02 | 28.44 | 3.03 | 5.01 |
| | 28.71 | 2.79 | 28.69 | 2.77 | 28.73 | 2.77 | 2.05 |
| | 30.78 | 0.91 | 30.77 | 0.88 | 30.79 | 0.89 | 1.60 |
| | 26.37 | 4.91 | 26.36 | 4.88 | 26.39 | 4.90 | 5.58 |
| | 26.54 | 4.76 | 26.47 | 4.79 | 26.51 | 4.79 | 4.68 |
| | 28.01 | 3.43 | 27.97 | 3.42 | 28.02 | 3.41 | 3.90 |
| | 28.49 | 2.99 | 28.47 | 2.97 | 28.50 | 2.97 | 3.09 |
| | 28.36 | 3.11 | 28.29 | 3.13 | 28.32 | 3.14 | 3.70 |
| | 28.98 | 2.54 | 28.94 | 2.54 | 28.97 | 2.55 | 2.80 |
| | 29.69 | 1.90 | 29.65 | 1.90 | 29.68 | 1.90 | 2.71 |
| | 30.46 | 1.20 | 30.41 | 1.20 | 30.45 | 1.20 | 1.81 |
| | 30.44 | 1.21 | 30.37 | 1.24 | 30.41 | 1.23 | 1.81 |

| | | | | | | | |
|---|---|---|---|---|---|---|---|
| | 26.91 | 4.42 | 26.83 | 4.46 | 26.87 | 4.46 | 4.85 |
| | 30.01 | 1.60 | 29.98 | 1.59 | 30.01 | 1.60 | 1.81 |
| | 30.31 | 1.34 | 30.25 | 1.34 | 30.29 | 1.34 | 1.81 |
| | 27.70 | 3.71 | 27.64 | 3.73 | 27.66 | 3.74 | 4.32 |
| | 28.57 | 2.92 | 28.58 | 2.87 | 28.59 | 2.89 | 3.06 |
| | 29.09 | 2.45 | 29.08 | 2.42 | 29.11 | 2.42 | 3.06 |
| | 27.65 | 3.75 | 27.62 | 3.75 | 27.65 | 3.75 | 3.95 |
| | 27.79 | 3.63 | 27.77 | 3.60 | 27.81 | 3.60 | 3.92 |
| | 26.50 | 4.80 | 26.45 | 4.81 | 26.48 | 4.82 | 4.13 |
| | 27.31 | 4.06 | 27.29 | 4.04 | 27.31 | 4.06 | 3.85 |
| | 23.51 | 7.52 | 23.41 | 7.57 | 23.47 | 7.56 | 8.03 |

[a] Parameters of linear regression models are presented in Table 2 in main text.
[b] For the references to the experimental works, please, see the main text.

**Table S3.** Calculated $^{13}$C NMR shielding constants, together with scaled and experimental chemical shifts – the GIAO-DFT(PBE0) calculations with the first-level basis sets.

| Mol. | pcS-1 | | pecS-1 | | pecS-1 mod | | $\delta_{exp}$[b] |
|---|---|---|---|---|---|---|---|
| | $\sigma$ | $\delta_{scaled}$[a] | $\sigma$ | $\delta_{scaled}$[a] | $\sigma$ | $\delta_{scaled}$[a] | |
| **1** | 126.4 | 58.3 | 124.3 | 57.5 | 124.2 | 58.4 | 55.5 |
| | 149.4 | 36.3 | 147.1 | 36.3 | 147.8 | 36.1 | 33.3 |
| | 141.6 | 43.8 | 138.4 | 44.4 | 139.3 | 44.1 | 35.9 |
| | 46.7 | 134.9 | 40.4 | 135.5 | 42.7 | 135.4 | 133.2 |
| | 130.2 | 54.7 | 128.4 | 53.6 | 128.6 | 54.2 | 54.6 |
| | 132.5 | 52.5 | 131.2 | 51.0 | 131.5 | 51.4 | 52.0 |
| | 40.9 | 140.4 | 33.5 | 142.0 | 36.4 | 141.3 | 135.2 |
| | 75.9 | 106.8 | 70.1 | 107.9 | 72.3 | 107.4 | 107.3 |
| | 164.0 | 22.3 | 162.5 | 21.9 | 162.8 | 21.9 | 19.5 |
| | 149.3 | 36.4 | 147.1 | 36.2 | 147.3 | 36.5 | 38.5 |
| | 56.7 | 125.2 | 49.7 | 126.9 | 51.3 | 127.3 | 128.1 |
| | 48.6 | 133.1 | 41.5 | 134.5 | 43.8 | 134.3 | 138.1 |
| | 62.6 | 119.6 | 57.5 | 119.7 | 60.0 | 119.1 | 124.5 |
| | 36.9 | 144.3 | 31.3 | 144.1 | 32.6 | 144.9 | 145.0 |
| | 64.2 | 118.0 | 59.9 | 117.4 | 61.4 | 117.7 | 118.6 |
| | 47.5 | 134.1 | 40.8 | 135.2 | 43.1 | 135.0 | 137.1 |
| | 72.2 | 110.3 | 67.9 | 110.0 | 69.4 | 110.2 | 112.2 |
| | 172.4 | 14.2 | 171.6 | 13.4 | 172.1 | 13.1 | 13.0 |
| | 56.4 | 125.5 | 49.8 | 126.8 | 51.9 | 126.7 | 130.1 |
| | 63.5 | 118.7 | 58.1 | 119.1 | 60.4 | 118.7 | 119.8 |
| | 61.5 | 120.7 | 56.4 | 120.7 | 58.6 | 120.4 | 122.5 |
| | 61.4 | 120.7 | 55.1 | 121.9 | 56.6 | 122.2 | 122.8 |
| | 49.4 | 132.2 | 42.7 | 133.4 | 45.0 | 133.3 | 136.9 |
| | 69.1 | 113.4 | 64.9 | 112.7 | 66.4 | 113.0 | 114.4 |
| | 42.9 | 138.5 | 38.5 | 137.3 | 40.3 | 137.6 | 137.2 |
| | 78.6 | 104.2 | 75.1 | 103.3 | 75.8 | 104.2 | 106.7 |
| | 69.6 | 112.9 | 65.1 | 112.5 | 66.6 | 112.8 | 113.6 |

| | | | | | | | |
|---|---|---|---|---|---|---|---|
| | 30.5 | 150.4 | 23.2 | 151.6 | 26.6 | 150.6 | 152.0 |
| | 65.2 | 117.1 | 60.0 | 117.3 | 62.0 | 117.2 | 119.3 |
| **2** | 56.6 | 125.4 | 49.1 | 127.5 | 51.4 | 127.2 | 125.7 |
| | 68.5 | 114.0 | 62.7 | 114.8 | 64.6 | 114.7 | 115.1 |
| | 58.0 | 124.0 | 53.8 | 123.1 | 55.4 | 123.4 | 124.1 |
| | 46.6 | 134.9 | 39.6 | 136.3 | 41.9 | 136.1 | 137.0 |
| | 71.0 | 111.6 | 66.5 | 111.3 | 68.0 | 111.5 | 111.4 |
| | 59.6 | 122.5 | 54.2 | 122.7 | 56.5 | 122.4 | 122.3 |
| | 62.5 | 119.7 | 57.1 | 120.0 | 59.2 | 119.9 | 119.9 |
| | 61.9 | 120.3 | 56.6 | 120.5 | 58.3 | 120.7 | 120.4 |
| | 118.1 | 66.3 | 113.3 | 67.7 | 113.7 | 68.3 | 68.2 |
| | 66.5 | 115.9 | 61.5 | 115.9 | 63.2 | 116.1 | 115.1 |
| | 57.9 | 124.1 | 50.2 | 126.4 | 52.3 | 126.3 | 125.7 |
| | 47.2 | 134.4 | 40.1 | 135.9 | 42.3 | 135.8 | 137.0 |
| | 55.1 | 126.8 | 50.4 | 126.3 | 52.2 | 126.5 | 124.1 |
| | 61.8 | 120.3 | 56.5 | 120.6 | 58.3 | 120.7 | 120.4 |
| | 62.8 | 119.4 | 57.3 | 119.8 | 59.4 | 119.6 | 119.9 |
| | 60.2 | 121.9 | 54.8 | 122.2 | 57.0 | 121.9 | 122.3 |
| | 72.1 | 110.5 | 67.6 | 110.2 | 69.2 | 110.4 | 111.4 |
| | -22.3 | 201.1 | -26.4 | 197.7 | -24.4 | 198.9 | 201.5 |
| | 62.9 | 119.3 | 55.4 | 121.6 | 57.6 | 121.4 | 120.8 |
| | 20.0 | 160.5 | 12.6 | 161.5 | 16.2 | 160.4 | 160.3 |
| | 55.7 | 126.2 | 50.2 | 126.4 | 52.0 | 126.6 | 125.5 |
| | 63.5 | 118.7 | 58.2 | 119.0 | 60.2 | 118.8 | 119.4 |
| | 43.0 | 138.4 | 36.9 | 138.8 | 39.4 | 138.6 | 137.6 |
| | 69.5 | 113.0 | 65.1 | 112.5 | 66.4 | 113.0 | 112.9 |
| **3** | 145.7 | 39.9 | 141.5 | 41.5 | 142.4 | 41.1 | 40.3 |
| | 127.7 | 57.1 | 124.7 | 57.1 | 124.4 | 58.2 | 64.8 |
| | 147.1 | 38.5 | 143.8 | 39.3 | 144.6 | 39.1 | 39.2 |
| | 114.5 | 69.8 | 109.8 | 70.9 | 111.0 | 70.8 | 72.2 |
| | 121.4 | 63.2 | 119.9 | 61.6 | 120.1 | 62.3 | 65.5 |
| | 143.3 | 42.1 | 140.9 | 42.0 | 141.1 | 42.4 | 41.7 |
| | 152.8 | 33.0 | 151.4 | 32.2 | 151.9 | 32.1 | 32.7 |
| | 34.3 | 146.7 | 29.2 | 146.0 | 31.4 | 146.1 | 138.6 |
| | 6.1 | 173.9 | 0.2 | 173.0 | 3.0 | 173.0 | 172.7 |
| | 47.5 | 134.1 | 42.8 | 133.4 | 44.1 | 134.1 | 137.9 |
| | 136.5 | 48.6 | 135.1 | 47.4 | 135.2 | 48.0 | 49.5 |
| | 143.9 | 41.6 | 142.3 | 40.7 | 142.8 | 40.8 | 41.6 |
| | 144.3 | 41.1 | 143.1 | 40.0 | 143.0 | 40.5 | 41.9 |
| | 126.0 | 58.7 | 125.0 | 56.8 | 125.0 | 57.6 | 52.9 |
| | 39.0 | 142.2 | 34.0 | 141.5 | 34.8 | 142.9 | 143.3 |
| | 163.2 | 23.0 | 161.1 | 23.2 | 162.0 | 22.6 | 21.1 |
| | 157.7 | 28.3 | 156.5 | 27.5 | 157.2 | 27.2 | 25.9 |
| | 161.3 | 24.8 | 159.6 | 24.6 | 160.7 | 23.9 | 24.5 |
| | 49.7 | 132.0 | 42.4 | 133.7 | 45.2 | 133.1 | 132.6 |
| | 157.0 | 29.0 | 155.4 | 28.5 | 156.3 | 28.0 | 26.5 |
| | 49.3 | 132.3 | 44.6 | 131.6 | 46.8 | 131.5 | 128.5 |
| | 153.5 | 32.3 | 151.7 | 31.9 | 152.7 | 31.4 | 25.9 |
| | 54.4 | 127.5 | 47.4 | 129.1 | 49.8 | 128.7 | 128.5 |
| | 154.6 | 31.3 | 152.8 | 30.9 | 153.7 | 30.5 | 25.8 |

| | | | | | | | |
|---|---|---|---|---|---|---|---|
| | 47.1 | 134.4 | 42.3 | 133.8 | 44.3 | 133.9 | 131.8 |
| | 42.7 | 138.7 | 38.3 | 137.5 | 40.1 | 137.8 | 137.8 |
| | 61.9 | 120.2 | 55.2 | 121.8 | 57.0 | 121.9 | 121.0 |
| | 67.9 | 114.5 | 63.6 | 114.0 | 65.1 | 114.2 | 113.7 |
| | 43.3 | 138.1 | 36.1 | 139.6 | 38.4 | 139.4 | 140.6 |
| | 21.7 | 158.9 | 15.5 | 158.8 | 17.6 | 159.1 | 148.1 |
| | 59.2 | 122.9 | 54.6 | 122.3 | 56.1 | 122.8 | 121.6 |
| | 71.0 | 111.5 | 66.6 | 111.1 | 68.1 | 111.4 | 111.9 |
| | 61.8 | 120.3 | 57.2 | 119.9 | 59.5 | 119.5 | 121.0 |
| | 62.2 | 120.0 | 56.6 | 120.5 | 58.9 | 120.1 | 119.5 |
| | 52.3 | 129.4 | 46.6 | 129.8 | 49.0 | 129.5 | 128.2 |
| | 14.5 | 165.8 | 8.4 | 165.4 | 12.1 | 164.3 | 167.0 |
| **4** | 18.9 | 161.6 | 13.0 | 161.0 | 15.0 | 161.6 | 161.4 |
| | 130.7 | 54.2 | 128.5 | 53.5 | 128.2 | 54.6 | 55.0 |
| | 11.5 | 168.6 | 6.9 | 166.7 | 8.6 | 167.6 | 168.2 |
| | 135.9 | 49.3 | 133.8 | 48.6 | 133.8 | 49.3 | 48.7 |
| | 131.3 | 53.6 | 125.8 | 56.1 | 127.7 | 55.1 | 52.4 |
| | 45.7 | 135.8 | 38.8 | 137.1 | 40.6 | 137.4 | 134.9 |
| | 57.7 | 124.3 | 52.4 | 124.4 | 54.2 | 124.5 | 122.6 |
| | 58.4 | 123.6 | 52.9 | 124.0 | 54.7 | 124.1 | 122.8 |
| | 49.5 | 132.2 | 43.5 | 132.7 | 45.9 | 132.4 | 130.0 |
| | 70.7 | 111.8 | 66.2 | 111.6 | 67.5 | 111.9 | 111.1 |
| | 37.2 | 144.0 | 29.8 | 145.4 | 32.9 | 144.7 | 144.8 |
| | 143.9 | 41.6 | 139.3 | 43.5 | 140.7 | 42.8 | 41.8 |
| | 97.7 | 85.9 | 94.7 | 85.0 | 95.1 | 85.9 | 87.3 |
| | 89.8 | 93.5 | 85.5 | 93.6 | 86.5 | 94.0 | 92.6 |
| | 154.8 | 31.1 | 152.2 | 31.5 | 152.6 | 31.5 | 31.1 |
| | 114.2 | 70.1 | 112.5 | 68.4 | 112.6 | 69.3 | 69.2 |
| | 148.7 | 37.0 | 146.5 | 36.8 | 146.8 | 37.0 | 35.8 |
| | 135.6 | 49.5 | 130.7 | 51.5 | 131.7 | 51.3 | 50.8 |
| | 107.6 | 76.4 | 104.6 | 75.8 | 104.6 | 76.9 | 76.2 |
| | 11.2 | 168.9 | 6.3 | 167.4 | 9.3 | 167.0 | 169.1 |
| | 132.2 | 52.8 | 131.2 | 51.1 | 131.4 | 51.5 | 51.7 |
| | 89.7 | 93.6 | 86.4 | 92.8 | 86.1 | 94.4 | 93.6 |
| | 135.0 | 50.1 | 133.4 | 49.0 | 133.7 | 49.4 | 48.6 |
| | 131.1 | 53.8 | 129.9 | 52.3 | 130.3 | 52.6 | 51.6 |
| | 152.7 | 33.1 | 151.6 | 32.1 | 152.2 | 31.9 | 30.6 |
| | 127.9 | 56.9 | 123.0 | 58.7 | 124.6 | 58.0 | 57.0 |
| | 42.4 | 139.0 | 36.1 | 139.6 | 37.8 | 140.0 | 136.6 |
| | 63.1 | 119.1 | 58.4 | 118.8 | 60.1 | 119.0 | 116.3 |
| | 58.7 | 123.3 | 53.5 | 123.4 | 55.4 | 123.4 | 121.6 |
| | 66.5 | 115.9 | 61.3 | 116.1 | 63.0 | 116.2 | 116.5 |
| | 40.7 | 140.6 | 34.5 | 141.1 | 37.4 | 140.4 | 142.0 |
| | 46.0 | 135.5 | 38.6 | 137.3 | 41.7 | 136.3 | 136.1 |
| | 158.3 | 27.7 | 156.7 | 27.3 | 157.6 | 26.7 | 25.5 |
| | 102.5 | 81.3 | 99.7 | 80.4 | 100.2 | 81.0 | 80.5 |
| | 154.0 | 31.8 | 150.5 | 33.0 | 151.5 | 32.5 | 31.2 |
| | 152.0 | 33.8 | 149.5 | 34.0 | 150.0 | 34.0 | 31.6 |
| | 117.2 | 67.2 | 115.7 | 65.5 | 115.9 | 66.2 | 65.1 |
| | 146.7 | 38.8 | 144.5 | 38.6 | 145.3 | 38.4 | 36.8 |

| | | | | | | | |
|---|---|---|---|---|---|---|---|
| | 141.3 | 44.0 | 136.2 | 46.4 | 137.8 | 45.5 | 46.3 |
| | 121.4 | 63.1 | 118.5 | 62.9 | 118.2 | 64.1 | 64.7 |
| | 153.6 | 32.2 | 151.2 | 32.4 | 151.4 | 32.6 | 29.3 |
| | 134.4 | 50.7 | 132.7 | 49.7 | 132.7 | 50.4 | 50.7 |
| | 21.7 | 158.8 | 15.7 | 158.6 | 17.6 | 159.1 | 158.7 |
| | 132.0 | 53.0 | 130.5 | 51.7 | 130.2 | 52.7 | 53.5 |
| | 19.1 | 161.3 | 14.4 | 159.8 | 16.7 | 160.0 | 158.7 |
| | 78.0 | 104.8 | 73.0 | 105.2 | 74.6 | 105.3 | 104.6 |
| | 123.8 | 60.9 | 118.1 | 63.2 | 119.7 | 62.6 | 60.9 |
| | 45.5 | 136.0 | 39.0 | 136.8 | 40.2 | 137.8 | 135.4 |
| | 54.6 | 127.2 | 49.6 | 127.0 | 51.2 | 127.4 | 124.4 |
| | 57.4 | 124.6 | 52.0 | 124.8 | 53.7 | 125.0 | 123.4 |
| | 48.8 | 132.8 | 43.3 | 132.9 | 45.5 | 132.8 | 131.2 |
| | 70.3 | 112.2 | 66.0 | 111.7 | 67.1 | 112.3 | 111.6 |
| | 37.7 | 143.5 | 30.8 | 144.6 | 33.7 | 143.9 | 144.7 |
| | 147.0 | 38.6 | 142.8 | 40.2 | 143.8 | 39.8 | 39.8 |
| | 98.0 | 85.6 | 95.2 | 84.5 | 95.6 | 85.4 | 87.1 |
| | 83.7 | 99.3 | 80.1 | 98.7 | 80.9 | 99.3 | 98.4 |
| | 156.8 | 29.2 | 154.3 | 29.5 | 154.2 | 30.0 | 28.2 |
| | 119.0 | 65.5 | 117.9 | 63.4 | 118.1 | 64.1 | 64.3 |
| | 148.2 | 37.4 | 146.3 | 37.0 | 146.8 | 37.0 | 37.0 |
| | 136.8 | 48.3 | 131.8 | 50.5 | 133.0 | 50.0 | 49.1 |
| | 112.1 | 72.1 | 109.4 | 71.3 | 108.8 | 73.0 | 72.1 |
| | 12.2 | 168.0 | 7.0 | 166.7 | 10.2 | 166.1 | 168.6 |
| | 132.1 | 52.8 | 131.2 | 51.0 | 131.4 | 51.5 | 51.9 |
| | 89.7 | 93.6 | 86.3 | 92.8 | 85.9 | 94.6 | 93.2 |
| | 134.8 | 50.3 | 133.4 | 49.0 | 133.6 | 49.5 | 48.6 |
| | 131.3 | 53.6 | 130.1 | 52.1 | 130.5 | 52.4 | 51.6 |
| | 151.2 | 34.5 | 150.2 | 33.3 | 150.8 | 33.2 | 31.4 |
| | 127.5 | 57.3 | 122.8 | 58.9 | 124.3 | 58.3 | 56.0 |
| | 43.3 | 138.1 | 37.2 | 138.6 | 38.6 | 139.3 | 135.9 |
| | 60.5 | 121.6 | 56.0 | 121.0 | 57.6 | 121.3 | 115.9 |
| | 58.5 | 123.5 | 53.2 | 123.7 | 55.1 | 123.7 | 120.9 |
| | 56.6 | 125.4 | 51.4 | 125.4 | 53.4 | 125.3 | 118.9 |
| | 42.7 | 138.7 | 36.6 | 139.2 | 39.1 | 138.9 | 142.0 |
| | 40.0 | 141.3 | 32.9 | 142.5 | 36.0 | 141.7 | 136.9 |
| | 158.3 | 27.8 | 156.8 | 27.2 | 157.6 | 26.8 | 25.5 |
| | 102.7 | 81.1 | 99.9 | 80.2 | 100.5 | 80.8 | 80.5 |
| | 153.2 | 32.6 | 150.1 | 33.4 | 150.8 | 33.2 | 32.3 |
| | 152.3 | 33.5 | 150.0 | 33.6 | 150.4 | 33.6 | 32.1 |
| | 117.6 | 66.8 | 116.2 | 65.0 | 116.4 | 65.7 | 64.8 |
| | 147.4 | 38.1 | 145.5 | 37.7 | 146.1 | 37.6 | 36.2 |
| | 141.3 | 44.0 | 136.3 | 46.3 | 137.8 | 45.5 | 46.1 |
| | 121.8 | 62.8 | 118.9 | 62.5 | 118.5 | 63.7 | 64.7 |
| | 149.1 | 36.6 | 147.2 | 36.1 | 147.7 | 36.2 | 36.0 |
| | 141.2 | 44.1 | 139.6 | 43.2 | 139.3 | 44.1 | 45.2 |
| **5** | 124.1 | 60.5 | 119.4 | 62.0 | 120.5 | 61.9 | 62.3 |
| | 122.0 | 62.6 | 118.9 | 62.5 | 119.0 | 63.3 | 63.8 |
| | 143.2 | 42.2 | 141.3 | 41.7 | 141.7 | 41.8 | 42.5 |
| | 97.1 | 86.5 | 93.4 | 86.2 | 94.1 | 86.8 | 88.0 |

| | | | | | | | |
|---|---|---|---|---|---|---|---|
| | 131.3 | 53.7 | 128.2 | 53.8 | 128.8 | 54.0 | 53.7 |
| | 141.5 | 43.8 | 138.4 | 44.4 | 139.5 | 43.9 | 44.6 |
| | 107.8 | 76.2 | 103.6 | 76.7 | 104.7 | 76.8 | 77.7 |
| | 150.7 | 35.0 | 149.2 | 34.3 | 149.5 | 34.4 | 35.9 |
| | 102.5 | 81.3 | 98.9 | 81.1 | 99.5 | 81.7 | 83.5 |
| | 129.2 | 55.7 | 125.0 | 56.8 | 126.0 | 56.7 | 57.5 |
| | 31.7 | 149.2 | 26.6 | 148.4 | 28.4 | 148.9 | 145.9 |
| | 137.3 | 47.8 | 133.7 | 48.7 | 134.5 | 48.7 | 49.5 |
| | 146.4 | 39.2 | 144.4 | 38.7 | 145.1 | 38.6 | 35.9 |
| | 45.6 | 136.0 | 39.4 | 136.5 | 42.4 | 135.7 | 133.7 |
| | 169.4 | 17.1 | 168.3 | 16.5 | 168.9 | 16.1 | 17.4 |
| | 160.3 | 25.8 | 158.0 | 26.1 | 159.0 | 25.5 | 25.0 |
| | 111.5 | 72.6 | 106.8 | 73.7 | 108.3 | 73.4 | 73.8 |
| | 159.8 | 26.3 | 158.5 | 25.6 | 159.0 | 25.5 | 27.6 |
| | 143.0 | 42.4 | 139.2 | 43.6 | 140.8 | 42.6 | 43.2 |
| | 103.0 | 80.8 | 99.9 | 80.2 | 100.3 | 80.9 | 82.2 |
| | 167.6 | 18.8 | 167.0 | 17.7 | 167.0 | 17.9 | 17.1 |
| | 140.6 | 44.7 | 138.4 | 44.3 | 139.0 | 44.3 | 42.1 |
| | 0.1 | 179.6 | -6.6 | 179.4 | -3.1 | 178.7 | 178.9 |
| | 136.9 | 48.2 | 133.5 | 48.9 | 134.2 | 48.9 | 50.1 |
| | 162.2 | 24.0 | 159.9 | 24.4 | 160.8 | 23.7 | 24.0 |
| | 156.2 | 29.8 | 154.8 | 29.0 | 155.4 | 28.9 | 30.0 |
| | 174.5 | 12.2 | 173.3 | 11.9 | 174.0 | 11.3 | 12.7 |
| | 143.4 | 42.1 | 139.9 | 42.9 | 141.3 | 42.2 | 43.5 |
| | -0.3 | 179.9 | -6.7 | 179.5 | -3.5 | 179.1 | 178.9 |
| | 174.7 | 12.0 | 173.5 | 11.6 | 174.2 | 11.1 | 12.9 |
| **6** | 45.4 | 136.1 | 39.6 | 136.3 | 42.0 | 136.1 | 135.3 |
| | 54.2 | 127.6 | 48.7 | 127.9 | 51.0 | 127.6 | 128.0 |
| | 53.2 | 128.6 | 47.5 | 128.9 | 49.4 | 129.1 | 127.4 |
| | 62.7 | 119.5 | 54.4 | 122.5 | 57.0 | 121.9 | 121.3 |
| | 37.1 | 144.1 | 30.3 | 145.0 | 32.6 | 144.9 | 146.3 |
| | 54.6 | 127.3 | 49.6 | 127.0 | 51.2 | 127.4 | 131.8 |
| | 17.9 | 162.5 | 13.0 | 161.1 | 15.5 | 161.1 | 162.2 |
| | 23.8 | 156.8 | 19.4 | 155.1 | 20.7 | 156.2 | 155.3 |
| | 49.9 | 131.8 | 43.3 | 132.9 | 45.4 | 132.9 | 133.7 |
| | 55.2 | 126.7 | 48.3 | 128.2 | 50.0 | 128.5 | 130.4 |
| | 14.5 | 165.8 | 9.4 | 164.5 | 12.3 | 164.1 | 168.4 |
| | 125.9 | 58.9 | 123.0 | 58.6 | 123.6 | 59.0 | 50.7 |
| | 52.4 | 129.4 | 46.4 | 130.0 | 48.5 | 129.9 | 128.6 |
| | 47.5 | 134.0 | 42.5 | 133.6 | 44.3 | 133.9 | 127.8 |
| | 52.5 | 129.3 | 47.4 | 129.1 | 49.6 | 128.9 | 129.3 |
| | 49.6 | 132.1 | 44.0 | 132.3 | 46.0 | 132.2 | 129.9 |
| | 140.5 | 44.8 | 138.0 | 44.8 | 139.1 | 44.3 | 42.9 |
| | 106.4 | 77.5 | 102.2 | 78.1 | 103.6 | 77.8 | 80.9 |
| | 45.0 | 136.4 | 38.0 | 137.8 | 39.8 | 138.1 | 139.6 |
| | 44.3 | 137.2 | 37.7 | 138.1 | 40.2 | 137.8 | 136.3 |
| | 97.2 | 86.4 | 95.1 | 84.6 | 94.8 | 86.2 | 86.8 |
| | 57.1 | 124.9 | 52.4 | 124.4 | 53.9 | 124.8 | 124.0 |
| | 57.3 | 124.7 | 52.4 | 124.4 | 54.2 | 124.5 | 126.0 |
| | 49.1 | 132.6 | 43.7 | 132.5 | 45.9 | 132.4 | 129.8 |

| | | | | | | | |
|---|---|---|---|---|---|---|---|
| | 64.4 | 117.9 | 59.8 | 117.5 | 61.0 | 118.1 | 115.9 |
| | 117.9 | 66.5 | 115.4 | 65.7 | 116.0 | 66.2 | 62.0 |
| | 9.6 | 170.5 | 4.7 | 168.9 | 7.5 | 168.7 | 171.4 |
| | 153.4 | 32.5 | 151.4 | 32.2 | 152.2 | 31.9 | 34.5 |
| | 161.0 | 25.1 | 158.6 | 25.5 | 160.0 | 24.5 | 25.2 |
| | 162.3 | 23.9 | 161.4 | 22.9 | 161.7 | 22.9 | 23.6 |
| | 163.0 | 23.2 | 161.9 | 22.5 | 162.1 | 22.5 | 21.4 |
| **7** | 111.7 | 72.5 | 108.0 | 72.7 | 107.8 | 73.9 | 73.8 |
| | 100.2 | 83.5 | 98.0 | 82.0 | 97.3 | 83.8 | 80.2 |
| | 109.8 | 74.3 | 105.7 | 74.7 | 105.6 | 76.0 | 73.6 |
| | 141.2 | 44.1 | 138.7 | 44.0 | 139.7 | 43.7 | 42.7 |
| | 52.0 | 129.8 | 45.3 | 131.0 | 47.3 | 131.0 | 126.7 |
| | 107.2 | 76.8 | 104.0 | 76.4 | 103.6 | 77.9 | 76.3 |
| | 19.7 | 160.8 | 15.6 | 158.6 | 18.3 | 158.5 | 163.1 |
| | 16.7 | 163.7 | 11.6 | 162.4 | 14.7 | 161.9 | 165.6 |
| | 32.9 | 148.1 | 25.7 | 149.3 | 28.7 | 148.7 | 148.4 |
| | 47.4 | 134.2 | 40.3 | 135.7 | 42.9 | 135.2 | 134.1 |
| | 53.7 | 128.2 | 49.2 | 127.4 | 50.8 | 127.7 | 127.3 |
| | 57.6 | 124.4 | 52.2 | 124.6 | 54.1 | 124.6 | 125.0 |
| | 122.1 | 62.4 | 120.8 | 60.7 | 121.8 | 60.6 | 60.4 |
| | 73.1 | 109.5 | 67.2 | 110.7 | 69.4 | 110.2 | 107.8 |
| | 52.7 | 129.1 | 45.4 | 130.9 | 47.5 | 130.8 | 130.4 |
| | 71.1 | 111.4 | 66.4 | 111.4 | 68.0 | 111.5 | 111.1 |
| | 160.8 | 25.3 | 160.1 | 24.2 | 160.4 | 24.1 | 27.5 |
| | 158.0 | 28.0 | 156.0 | 27.9 | 156.4 | 27.9 | 27.1 |
| | 61.9 | 120.3 | 56.4 | 120.7 | 58.4 | 120.6 | 120.3 |
| | 72.1 | 110.5 | 66.7 | 111.1 | 68.5 | 111.0 | 111.5 |
| | 107.4 | 76.5 | 103.5 | 76.8 | 103.2 | 78.2 | 74.9 |
| | 50.0 | 131.7 | 44.2 | 132.0 | 46.5 | 131.9 | 131.4 |
| | 57.0 | 124.9 | 52.3 | 124.5 | 54.2 | 124.6 | 119.2 |
| | 109.0 | 75.0 | 105.1 | 75.4 | 104.8 | 76.7 | 76.6 |
| | 59.6 | 122.5 | 54.2 | 122.7 | 56.2 | 122.6 | 122.8 |
| | 17.1 | 163.3 | 12.2 | 161.9 | 15.0 | 161.6 | 165.6 |
| | 62.9 | 119.3 | 57.5 | 119.7 | 59.6 | 119.5 | 120.6 |
| | 14.2 | 166.1 | 9.2 | 164.7 | 12.4 | 164.0 | 166.8 |
| | 160.2 | 25.9 | 159.5 | 24.7 | 159.9 | 24.6 | 28.3 |
| | 121.0 | 63.6 | 119.2 | 62.2 | 120.1 | 62.3 | 61.1 |
| | 162.0 | 24.2 | 161.4 | 22.9 | 161.6 | 23.0 | 27.5 |
| **8** | 146.4 | 39.2 | 141.8 | 41.1 | 143.1 | 40.5 | 40.5 |
| | 129.6 | 55.3 | 126.3 | 55.6 | 126.6 | 56.1 | 56.7 |
| | 121.7 | 62.8 | 118.7 | 62.7 | 119.3 | 63.0 | 62.9 |
| | 155.0 | 30.9 | 151.0 | 32.6 | 152.0 | 32.1 | 31.7 |
| | 103.1 | 80.8 | 100.5 | 79.6 | 100.8 | 80.4 | 81.1 |
| | 136.1 | 49.1 | 132.4 | 49.9 | 133.1 | 49.9 | 50.3 |
| | 153.2 | 32.7 | 151.1 | 32.5 | 152.0 | 32.1 | 32.2 |
| | 145.7 | 39.8 | 143.9 | 39.2 | 144.3 | 39.4 | 39.9 |
| | 143.8 | 41.6 | 140.7 | 42.2 | 142.3 | 41.3 | 42.0 |
| | 164.7 | 21.5 | 162.2 | 22.1 | 163.1 | 21.6 | 21.1 |
| | 149.5 | 36.2 | 144.6 | 38.5 | 146.3 | 37.5 | 37.2 |
| | 75.5 | 107.2 | 71.3 | 106.8 | 72.1 | 107.6 | 109.3 |

| | | | | | | | |
|---|---|---|---|---|---|---|---|
| | 169.9 | 16.6 | 168.3 | 16.5 | 168.6 | 16.4 | 16.3 |
| | 152.8 | 33.1 | 151.0 | 32.6 | 151.5 | 32.5 | 32.3 |
| | 36.7 | 144.4 | 30.0 | 145.3 | 32.1 | 145.4 | 140.8 |
| | 147.8 | 37.9 | 145.6 | 37.6 | 146.1 | 37.6 | 37.5 |
| | 172.0 | 14.6 | 171.0 | 14.0 | 171.5 | 13.6 | 15.0 |
| | 154.4 | 31.5 | 152.4 | 31.3 | 153.4 | 30.7 | 31.7 |
| | 57.1 | 124.9 | 53.0 | 123.8 | 55.1 | 123.7 | 121.8 |
| | 166.4 | 20.0 | 164.9 | 19.6 | 165.2 | 19.6 | 19.4 |
| | 143.9 | 41.5 | 141.6 | 41.3 | 142.3 | 41.2 | 39.0 |
| | 155.3 | 30.6 | 153.4 | 30.3 | 154.3 | 29.9 | 30.4 |
| | 112.7 | 71.5 | 110.0 | 70.8 | 110.5 | 71.3 | 78.2 |
| | 156.9 | 29.1 | 155.4 | 28.5 | 155.9 | 28.4 | 29.3 |
| | 155.7 | 30.2 | 152.7 | 31.1 | 154.3 | 29.9 | 30.6 |
| | 118.1 | 66.3 | 116.6 | 64.6 | 116.9 | 65.3 | 66.9 |
| | 169.4 | 17.1 | 168.3 | 16.5 | 168.8 | 16.2 | 17.3 |
| | 82.4 | 100.6 | 80.1 | 98.6 | 80.6 | 99.6 | 102.5 |
| | 114.6 | 69.7 | 111.4 | 69.5 | 112.7 | 69.3 | 71.8 |
| | 107.7 | 76.3 | 104.9 | 75.5 | 106.1 | 75.4 | 78.2 |
| | 108.5 | 75.6 | 105.5 | 74.9 | 106.6 | 75.0 | 78.2 |
| | 112.3 | 71.9 | 109.4 | 71.4 | 110.4 | 71.4 | 75.5 |
| | 120.5 | 64.1 | 119.1 | 62.3 | 120.2 | 62.1 | 62.7 |
| **9** | 147.2 | 38.4 | 142.8 | 40.3 | 143.9 | 39.8 | 40.1 |
| | 124.9 | 59.8 | 121.4 | 60.1 | 122.0 | 60.5 | 60.9 |
| | 143.2 | 42.2 | 138.5 | 44.2 | 139.7 | 43.7 | 44.0 |
| | 134.7 | 50.3 | 131.4 | 50.8 | 131.9 | 51.1 | 51.6 |
| | 168.5 | 17.9 | 165.8 | 18.8 | 166.7 | 18.2 | 17.3 |
| | 148.5 | 37.1 | 146.2 | 37.0 | 146.7 | 37.1 | 37.4 |
| | 109.6 | 74.5 | 106.9 | 73.7 | 107.2 | 74.4 | 76.1 |
| | 146.2 | 39.4 | 143.6 | 39.5 | 144.1 | 39.6 | 37.9 |
| | 132.3 | 52.7 | 127.6 | 54.4 | 128.4 | 54.4 | 53.5 |
| | 145.9 | 39.6 | 141.9 | 41.1 | 142.5 | 41.1 | 38.5 |
| | 138.2 | 47.0 | 134.7 | 47.7 | 135.2 | 48.0 | 42.6 |
| | 157.8 | 28.2 | 153.2 | 30.5 | 154.5 | 29.7 | 30.0 |
| | 148.1 | 37.5 | 146.0 | 37.3 | 146.5 | 37.3 | 37.3 |
| | 157.9 | 28.1 | 155.5 | 28.4 | 155.8 | 28.5 | 32.1 |
| | 154.9 | 31.0 | 152.7 | 31.0 | 152.9 | 31.3 | 35.5 |
| | 160.5 | 25.6 | 156.2 | 27.8 | 157.8 | 26.6 | 27.8 |
| | 152.9 | 32.9 | 150.7 | 32.9 | 151.4 | 32.7 | 32.2 |
| | 155.0 | 30.9 | 152.7 | 31.0 | 153.4 | 30.7 | 35.0 |
| | 152.8 | 33.0 | 149.9 | 33.6 | 151.0 | 33.0 | 31.1 |
| | 117.4 | 67.0 | 113.7 | 67.3 | 115.1 | 66.9 | 68.7 |
| | 1.9 | 177.8 | -3.9 | 176.9 | -0.7 | 176.4 | 176.8 |
| | 102.4 | 81.4 | 99.2 | 80.9 | 99.7 | 81.5 | 83.8 |
| | 170.3 | 16.2 | 169.1 | 15.8 | 169.8 | 15.3 | 15.5 |
| | 173.6 | 13.0 | 172.0 | 13.1 | 172.3 | 12.9 | 12.8 |
| | 170.9 | 15.6 | 169.0 | 15.8 | 169.5 | 15.5 | 13.1 |
| | 168.9 | 17.5 | 167.2 | 17.5 | 167.6 | 17.3 | 21.5 |
| | 149.3 | 36.3 | 148.1 | 35.3 | 148.3 | 35.6 | 30.6 |
| | 153.3 | 32.6 | 152.2 | 31.5 | 152.4 | 31.7 | 30.0 |
| | 150.6 | 35.1 | 149.5 | 34.0 | 149.7 | 34.2 | 34.1 |

| | | | | | | | |
|---|---|---|---|---|---|---|---|
| | -0.7 | 180.4 | -5.4 | 178.2 | -3.0 | 178.6 | 178.0 |
| **10** | 130.5 | 54.4 | 127.5 | 54.5 | 128.1 | 54.7 | 57.9 |
| | 161.7 | 24.5 | 159.6 | 24.6 | 160.1 | 24.4 | 25.2 |
| | 151.9 | 33.9 | 148.9 | 34.5 | 150.5 | 33.5 | 30.5 |
| | 143.8 | 41.7 | 141.8 | 41.2 | 142.5 | 41.0 | 44.1 |
| | 137.8 | 47.4 | 134.9 | 47.6 | 135.5 | 47.7 | 50.5 |
| | 72.7 | 109.9 | 67.2 | 110.6 | 68.9 | 110.6 | 110.6 |
| | 9.4 | 170.6 | 4.7 | 168.8 | 7.3 | 168.9 | 171.3 |
| | 111.8 | 72.3 | 109.0 | 71.7 | 109.9 | 71.8 | 71.7 |
| | 146.9 | 38.7 | 144.0 | 39.1 | 145.1 | 38.6 | 40.8 |
| | 138.4 | 46.9 | 136.1 | 46.5 | 136.6 | 46.6 | 42.6 |
| | 138.1 | 47.1 | 135.6 | 47.0 | 136.2 | 47.0 | 47.1 |
| | 2.3 | 177.5 | -2.1 | 175.2 | 0.5 | 175.3 | 174.9 |
| | 49.5 | 132.2 | 43.5 | 132.7 | 46.1 | 132.2 | 134.1 |
| | 41.3 | 140.0 | 37.0 | 138.8 | 38.6 | 139.3 | 130.6 |
| | 144.2 | 41.2 | 142.5 | 40.5 | 143.2 | 40.4 | 42.1 |
| | 56.0 | 126.0 | 48.7 | 127.9 | 50.6 | 127.9 | 128.5 |
| | 3.5 | 176.3 | -1.6 | 174.7 | 1.3 | 174.5 | 177.9 |
| | 163.0 | 23.2 | 162.4 | 22.0 | 162.7 | 22.0 | 22.0 |
| | 155.6 | 30.3 | 154.7 | 29.2 | 154.7 | 29.5 | 31.1 |
| | 46.8 | 134.8 | 40.9 | 135.1 | 42.7 | 135.4 | 132.7 |
| | 56.4 | 125.6 | 52.5 | 124.3 | 54.1 | 124.6 | 124.2 |
| | 46.7 | 134.8 | 40.1 | 135.9 | 42.3 | 135.8 | 138.1 |
| | 166.2 | 20.1 | 165.2 | 19.3 | 166.1 | 18.8 | 19.6 |
| | 174.1 | 12.6 | 172.9 | 12.2 | 173.4 | 11.9 | 20.0 |
| | 6.9 | 173.0 | 2.6 | 170.8 | 5.7 | 170.4 | 172.1 |
| | 169.5 | 17.0 | 168.7 | 16.2 | 169.3 | 15.7 | 18.5 |
| | 166.2 | 20.1 | 164.2 | 20.3 | 164.8 | 20.0 | 18.2 |
| | 62.0 | 120.2 | 57.1 | 120.0 | 58.8 | 120.2 | 119.3 |
| | 70.9 | 111.7 | 66.8 | 111.0 | 68.2 | 111.3 | 112.3 |
| | 52.0 | 129.8 | 46.2 | 130.2 | 48.3 | 130.1 | 128.2 |
| | 54.0 | 127.8 | 48.1 | 128.4 | 50.3 | 128.2 | 128.2 |
| | 62.4 | 119.8 | 57.3 | 119.8 | 59.3 | 119.7 | 119.8 |
| | 60.0 | 122.1 | 55.1 | 121.9 | 57.0 | 121.9 | 122.4 |
| | 69.0 | 113.4 | 63.6 | 113.9 | 65.5 | 113.8 | 116.2 |
| | 67.7 | 114.7 | 62.6 | 114.9 | 64.2 | 115.1 | 116.2 |
| | 24.8 | 155.9 | 17.2 | 157.2 | 20.9 | 156.0 | 157.7 |
| **11** | 135.1 | 50.0 | 132.4 | 49.9 | 133.4 | 49.7 | 49.4 |
| | 17.5 | 162.9 | 12.4 | 161.6 | 14.1 | 162.4 | 169.4 |
| | 68.0 | 114.4 | 60.9 | 116.5 | 63.2 | 116.0 | 106.6 |
| | 41.8 | 139.6 | 34.5 | 141.1 | 37.1 | 140.7 | 141.5 |
| | 153.0 | 32.8 | 151.0 | 32.6 | 151.5 | 32.6 | 36.9 |
| | 21.2 | 159.4 | 14.5 | 159.7 | 17.9 | 158.8 | 165.3 |
| | 65.0 | 117.3 | 58.8 | 118.5 | 60.4 | 118.7 | 124.5 |
| | 23.6 | 157.0 | 17.1 | 157.3 | 20.6 | 156.3 | 158.1 |
| | 73.3 | 109.3 | 69.0 | 108.9 | 69.8 | 109.8 | 117.3 |
| | 59.6 | 122.5 | 53.3 | 123.6 | 55.0 | 123.8 | 124.4 |
| | 44.0 | 137.5 | 39.4 | 136.5 | 40.7 | 137.3 | 134.8 |
| | 62.3 | 119.9 | 56.0 | 121.0 | 58.0 | 121.0 | 120.6 |
| | 31.1 | 149.8 | 25.9 | 149.1 | 27.9 | 149.4 | 151.8 |

| | | | | | | | |
|---|---|---|---|---|---|---|---|
| | 72.9 | 109.7 | 65.8 | 111.9 | 67.6 | 111.9 | 115.2 |
| | 49.2 | 132.5 | 42.0 | 134.1 | 44.1 | 134.1 | 137.2 |
| | 63.5 | 118.7 | 58.3 | 118.9 | 59.4 | 119.6 | 120.5 |
| | 43.5 | 137.9 | 36.5 | 139.2 | 39.1 | 138.8 | 136.9 |
| | 77.6 | 105.2 | 71.9 | 106.2 | 73.3 | 106.5 | 107.9 |
| | 24.7 | 155.9 | 18.6 | 155.9 | 20.6 | 156.3 | 157.8 |
| | 62.3 | 119.9 | 55.6 | 121.4 | 57.7 | 121.2 | 120.6 |
| | 47.8 | 133.8 | 43.3 | 132.9 | 44.8 | 133.4 | 135.0 |
| | 55.5 | 126.4 | 49.4 | 127.2 | 50.7 | 127.8 | 124.5 |
| | 47.0 | 134.6 | 39.8 | 136.2 | 41.7 | 136.4 | 136.6 |
| | 69.1 | 113.3 | 61.7 | 115.8 | 63.4 | 115.8 | 114.9 |
| | 28.3 | 152.5 | 22.6 | 152.1 | 24.9 | 152.3 | 152.5 |
| | 22.0 | 158.5 | 15.7 | 158.6 | 17.9 | 158.8 | 157.8 |
| | 78.5 | 104.3 | 72.4 | 105.7 | 73.7 | 106.1 | 107.7 |
| | 43.4 | 138.0 | 36.5 | 139.2 | 39.1 | 138.8 | 136.8 |
| | 66.5 | 115.8 | 61.2 | 116.2 | 62.5 | 116.7 | 119.3 |
| | 64.2 | 118.1 | 58.2 | 119.0 | 59.8 | 119.3 | 118.9 |
| | 26.4 | 154.3 | 20.4 | 154.2 | 23.9 | 153.2 | 155.9 |
| | 85.6 | 97.5 | 81.7 | 97.1 | 82.7 | 97.6 | 101.7 |
| | 27.6 | 153.2 | 20.9 | 153.7 | 24.3 | 152.8 | 155.5 |
| | 67.6 | 114.8 | 60.8 | 116.6 | 62.4 | 116.8 | 116.4 |
| | 42.8 | 138.6 | 35.7 | 139.9 | 38.4 | 139.5 | 134.7 |
| | 151.6 | 34.2 | 149.6 | 33.9 | 149.2 | 34.7 | 34.7 |
| | 144.4 | 41.1 | 141.8 | 41.2 | 142.7 | 40.9 | 44.0 |
| | 138.9 | 46.4 | 136.8 | 45.8 | 136.9 | 46.4 | 48.9 |
| | 170.9 | 15.6 | 170.3 | 14.7 | 170.5 | 14.6 | 19.2 |
| | 124.4 | 60.3 | 123.5 | 58.2 | 123.6 | 59.0 | 61.0 |
| | 163.9 | 22.4 | 163.0 | 21.4 | 163.1 | 21.6 | 22.1 |
| | 130.4 | 54.5 | 129.1 | 53.0 | 129.5 | 53.4 | 56.9 |
| | 131.3 | 53.6 | 130.0 | 52.2 | 130.3 | 52.6 | 56.9 |
| | 164.1 | 22.1 | 163.3 | 21.2 | 163.3 | 21.4 | 22.2 |
| | 165.8 | 20.5 | 164.7 | 19.8 | 165.2 | 19.7 | 19.7 |
| | 164.1 | 22.2 | 163.5 | 21.0 | 163.8 | 20.9 | 20.8 |
| **12** | 123.9 | 60.7 | 118.5 | 62.8 | 119.5 | 62.8 | 65.7 |
| | 133.8 | 51.3 | 128.3 | 53.8 | 129.7 | 53.1 | 51.3 |
| | 136.9 | 48.3 | 133.6 | 48.8 | 133.8 | 49.3 | 48.1 |
| | 141.0 | 44.3 | 135.2 | 47.3 | 136.7 | 46.5 | 45.9 |
| | 149.1 | 36.5 | 144.1 | 39.0 | 145.9 | 37.9 | 37.9 |
| | 133.4 | 51.6 | 130.3 | 51.9 | 131.7 | 51.3 | 53.9 |
| | 124.5 | 60.1 | 121.0 | 60.5 | 121.9 | 60.6 | 60.6 |
| | 167.0 | 19.4 | 164.4 | 20.1 | 165.6 | 19.2 | 18.9 |
| | 150.9 | 34.8 | 149.3 | 34.2 | 149.6 | 34.3 | 30.8 |
| | 105.3 | 78.6 | 102.6 | 77.6 | 103.4 | 78.0 | 79.1 |
| | -32.7 | 211.0 | -37.8 | 208.4 | -36.1 | 209.9 | 206.1 |
| | 148.5 | 37.2 | 145.4 | 37.8 | 146.6 | 37.2 | 36.4 |
| | 105.2 | 78.7 | 101.1 | 79.1 | 102.6 | 78.8 | 80.3 |
| | 108.6 | 75.5 | 105.4 | 75.1 | 105.7 | 75.9 | 77.8 |
| | 170.0 | 16.5 | 167.7 | 17.0 | 168.4 | 16.6 | 17.6 |
| | 119.9 | 64.6 | 117.0 | 64.3 | 117.3 | 64.9 | 65.3 |
| | 164.5 | 21.8 | 163.0 | 21.5 | 163.4 | 21.3 | 21.4 |

| | | | | | | | |
|---|---|---|---|---|---|---|---|
| | 13.2 | 167.1 | 7.5 | 166.2 | 10.2 | 166.1 | 166.6 |
| | 150.1 | 35.6 | 147.7 | 35.7 | 148.6 | 35.3 | 35.6 |
| | 166.5 | 19.9 | 165.2 | 19.4 | 165.4 | 19.4 | 20.7 |
| | 157.3 | 28.7 | 156.2 | 27.7 | 156.6 | 27.8 | 30.2 |
| | 60.8 | 121.3 | 53.8 | 123.1 | 56.1 | 122.7 | 120.0 |
| | 11.0 | 169.1 | 5.1 | 168.4 | 8.0 | 168.2 | 169.1 |
| | 69.8 | 112.7 | 64.8 | 112.9 | 66.8 | 112.7 | 109.7 |
| | 40.6 | 140.7 | 35.1 | 140.5 | 37.6 | 140.2 | 141.1 |
| | 38.0 | 143.2 | 32.2 | 143.2 | 34.9 | 142.8 | 143.2 |
| **13** | 122.1 | 62.5 | 119.6 | 61.9 | 119.3 | 63.0 | 62.9 |
| | 134.9 | 50.2 | 129.4 | 52.7 | 131.1 | 51.8 | 53.5 |
| | 142.2 | 43.2 | 139.4 | 43.4 | 140.8 | 42.7 | 41.4 |
| | 98.9 | 84.7 | 97.0 | 82.8 | 97.2 | 83.9 | 82.7 |
| | 120.9 | 63.6 | 118.4 | 62.9 | 118.4 | 63.8 | 65.4 |
| | 158.9 | 27.2 | 155.6 | 28.3 | 156.5 | 27.8 | 29.2 |
| | 161.7 | 24.5 | 159.4 | 24.8 | 160.1 | 24.4 | 23.3 |
| | 144.8 | 40.7 | 142.8 | 40.2 | 143.4 | 40.2 | 37.3 |
| | 38.5 | 142.8 | 31.1 | 144.2 | 33.4 | 144.2 | 133.9 |
| | 29.5 | 151.3 | 22.4 | 152.3 | 25.2 | 152.0 | 146.5 |
| | 126.9 | 57.8 | 125.6 | 56.3 | 125.6 | 57.0 | 53.8 |
| | 31.5 | 149.5 | 24.2 | 150.6 | 26.8 | 150.5 | 141.5 |
| | 126.8 | 58.0 | 125.0 | 56.8 | 124.8 | 57.8 | 52.0 |
| | 59.7 | 122.4 | 54.4 | 122.5 | 56.4 | 122.5 | 122.7 |
| | 74.5 | 108.2 | 69.5 | 108.5 | 71.3 | 108.4 | 103.8 |
| | 61.1 | 121.0 | 57.3 | 119.9 | 59.4 | 119.6 | 117.7 |
| | 62.4 | 119.8 | 57.0 | 120.1 | 59.1 | 119.9 | 117.2 |
| | 55.3 | 126.6 | 49.5 | 127.1 | 51.7 | 126.9 | 128.2 |
| | 170.6 | 16.0 | 169.7 | 15.1 | 170.2 | 14.9 | 13.3 |
| **14** | 150.9 | 34.9 | 146.8 | 36.5 | 147.6 | 36.2 | 35.1 |
| | 158.1 | 27.9 | 154.4 | 29.5 | 155.3 | 28.9 | 28.5 |
| | 151.4 | 34.3 | 148.1 | 35.3 | 148.7 | 35.2 | 37.2 |
| | 84.0 | 99.1 | 80.4 | 98.3 | 80.7 | 99.5 | 102.6 |
| | 151.2 | 34.5 | 148.6 | 34.8 | 149.0 | 34.9 | 36.2 |
| | 66.7 | 115.7 | 59.3 | 118.0 | 61.6 | 117.6 | 116.8 |
| | 72.6 | 110.0 | 66.2 | 111.6 | 67.5 | 112.0 | 117.5 |
| | 62.6 | 119.6 | 57.5 | 119.7 | 59.9 | 119.1 | 122.9 |
| | 40.5 | 140.8 | 33.5 | 142.0 | 36.6 | 141.1 | 133.7 |
| | 69.2 | 113.2 | 62.3 | 115.2 | 64.5 | 114.8 | 113.4 |
| | 28.3 | 152.5 | 21.5 | 153.2 | 24.2 | 152.9 | 154.5 |
| | 27.7 | 153.1 | 20.9 | 153.7 | 23.8 | 153.3 | 155.0 |
| | 25.0 | 155.7 | 17.8 | 156.6 | 21.0 | 155.9 | 156.7 |
| | 54.0 | 127.9 | 47.7 | 128.7 | 49.9 | 128.6 | 127.9 |
| | 161.5 | 24.7 | 160.7 | 23.6 | 160.9 | 23.7 | 23.9 |
| | 77.2 | 105.5 | 73.0 | 105.2 | 73.4 | 106.4 | 105.0 |
| | 81.5 | 101.5 | 77.1 | 101.5 | 78.2 | 101.9 | 104.6 |
| | 23.7 | 156.9 | 16.9 | 157.5 | 20.3 | 156.5 | 159.9 |
| | 50.0 | 131.7 | 43.9 | 132.3 | 46.2 | 132.1 | 130.3 |
| | 79.4 | 103.5 | 75.1 | 103.2 | 76.2 | 103.8 | 105.4 |
| | 53.1 | 128.7 | 46.7 | 129.7 | 48.5 | 130.0 | 131.1 |
| | 75.8 | 107.0 | 71.2 | 106.9 | 72.7 | 107.0 | 109.9 |

| | | | | | | | |
|---|---|---|---|---|---|---|---|
| | 24.3 | 156.3 | 17.0 | 157.3 | 20.9 | 156.0 | 157.9 |
| | 82.1 | 100.9 | 77.4 | 101.1 | 78.4 | 101.7 | 103.9 |
| | 77.7 | 105.1 | 72.9 | 105.3 | 74.4 | 105.5 | 107.1 |
| | 22.2 | 158.4 | 14.7 | 159.5 | 18.6 | 158.2 | 157.7 |
| | 27.7 | 153.1 | 20.7 | 153.9 | 23.2 | 153.9 | 156.7 |
| | 80.4 | 102.5 | 76.1 | 102.3 | 77.4 | 102.6 | 102.2 |
| | 61.3 | 120.8 | 53.8 | 123.1 | 56.3 | 122.6 | 122.5 |
| | 27.3 | 153.5 | 19.5 | 155.0 | 22.3 | 154.7 | 153.3 |
| | 59.2 | 122.9 | 54.8 | 122.2 | 56.4 | 122.5 | 122.0 |
| | 87.4 | 95.8 | 83.9 | 95.1 | 84.7 | 95.7 | 98.4 |
| | 71.1 | 111.5 | 66.0 | 111.7 | 67.9 | 111.5 | 113.4 |
| | 25.8 | 154.9 | 18.3 | 156.2 | 22.0 | 155.0 | 157.5 |
| **15** | 142.5 | 42.9 | 139.1 | 43.6 | 140.2 | 43.3 | 45.9 |
| | 90.6 | 92.7 | 87.4 | 91.8 | 87.9 | 92.7 | 89.4 |
| | 96.5 | 87.0 | 92.7 | 86.8 | 93.2 | 87.6 | 84.6 |
| | 139.0 | 46.3 | 135.6 | 46.9 | 136.8 | 46.5 | 46.8 |
| | 97.8 | 85.8 | 94.3 | 85.4 | 94.8 | 86.1 | 76.5 |
| | 173.6 | 13.1 | 172.4 | 12.6 | 172.9 | 12.3 | 12.1 |
| | 8.5 | 171.5 | 3.6 | 169.9 | 6.1 | 170.0 | 170.5 |
| | 52.6 | 129.2 | 45.0 | 131.3 | 47.2 | 131.2 | 130.0 |
| | 49.4 | 132.3 | 43.5 | 132.7 | 45.5 | 132.7 | 130.0 |
| | 51.7 | 130.1 | 46.1 | 130.3 | 48.2 | 130.2 | 128.5 |
| | 48.6 | 133.0 | 43.0 | 133.1 | 45.2 | 133.1 | 133.1 |
| | 8.4 | 171.6 | 3.0 | 170.4 | 6.1 | 170.1 | 169.7 |
| | 161.0 | 25.2 | 160.0 | 24.2 | 160.6 | 24.0 | 20.6 |
| | 8.7 | 171.3 | 3.9 | 169.6 | 7.1 | 169.1 | 166.2 |
| | 163.5 | 22.7 | 163.0 | 21.5 | 163.4 | 21.3 | 22.1 |
| | 108.6 | 75.4 | 105.0 | 75.4 | 105.6 | 76.0 | 71.2 |
| | 50.2 | 131.5 | 44.5 | 131.8 | 46.7 | 131.7 | 133.9 |
| | 160.8 | 25.3 | 159.9 | 24.3 | 160.1 | 24.4 | 20.5 |
| | 47.3 | 134.3 | 42.9 | 133.2 | 44.6 | 133.6 | 124.1 |
| | 7.2 | 172.8 | 2.0 | 171.4 | 5.0 | 171.1 | 169.0 |
| | 166.0 | 20.3 | 164.8 | 19.8 | 165.4 | 19.4 | 13.7 |
| | 147.2 | 38.4 | 145.3 | 37.9 | 145.2 | 38.5 | 39.9 |
| | 150.1 | 35.6 | 146.0 | 37.2 | 147.3 | 36.5 | 39.2 |
| | 97.3 | 86.3 | 94.5 | 85.2 | 95.1 | 85.9 | 74.3 |
| | 160.6 | 25.5 | 159.3 | 24.9 | 159.8 | 24.7 | 24.1 |
| | 159.3 | 26.8 | 158.0 | 26.0 | 158.2 | 26.3 | 25.5 |
| | 9.4 | 170.7 | 3.7 | 169.7 | 7.1 | 169.1 | 170.0 |
| | 166.7 | 19.6 | 165.6 | 19.1 | 166.3 | 18.6 | 15.2 |
| | 100.7 | 83.0 | 97.3 | 82.6 | 97.9 | 83.2 | 74.1 |
| | -25.2 | 203.9 | -30.1 | 201.2 | -27.2 | 201.5 | 197.5 |
| | 40.4 | 140.9 | 35.1 | 140.5 | 37.7 | 140.2 | 132.1 |
| | 11.3 | 168.9 | 5.7 | 167.9 | 8.5 | 167.7 | 170.2 |
| | 165.7 | 20.6 | 164.6 | 19.9 | 165.3 | 19.5 | 20.5 |
| | 36.9 | 144.2 | 32.1 | 143.3 | 34.0 | 143.6 | 136.9 |
| | 161.5 | 24.6 | 160.8 | 23.4 | 161.0 | 23.6 | 20.5 |
| **16** | 12.4 | 167.8 | 7.3 | 166.4 | 10.1 | 166.2 | 169.3 |
| | 56.5 | 125.5 | 49.9 | 126.7 | 51.9 | 126.8 | 125.3 |
| | 122.2 | 62.4 | 120.9 | 60.6 | 121.6 | 60.9 | 63.9 |

| | | | | | | | |
|---|---|---|---|---|---|---|---|
| | 117.0 | 67.3 | 114.4 | 66.7 | 115.0 | 67.1 | 66.8 |
| | 116.5 | 67.8 | 113.6 | 67.4 | 114.4 | 67.6 | 69.5 |
| | 91.6 | 91.7 | 89.2 | 90.1 | 90.4 | 90.3 | 90.7 |
| | 108.5 | 75.6 | 105.8 | 74.7 | 106.9 | 74.7 | 76.9 |
| | 110.0 | 74.1 | 107.3 | 73.3 | 108.0 | 73.7 | 75.0 |
| | 11.1 | 169.0 | 6.2 | 167.5 | 8.6 | 167.6 | 169.6 |
| | 57.9 | 124.1 | 51.6 | 125.1 | 53.3 | 125.4 | 124.8 |
| | 74.8 | 107.9 | 70.3 | 107.7 | 71.4 | 108.3 | 107.7 |
| | 37.7 | 143.5 | 31.1 | 144.2 | 34.6 | 143.1 | 145.1 |
| | 45.0 | 136.5 | 38.0 | 137.8 | 41.4 | 136.6 | 136.3 |
| | 40.4 | 140.9 | 34.6 | 141.0 | 37.2 | 140.7 | 144.2 |
| | 67.4 | 115.0 | 60.2 | 117.1 | 62.8 | 116.4 | 115.0 |
| | 10.3 | 169.8 | 5.4 | 168.2 | 7.8 | 168.4 | 170.5 |
| | 56.8 | 125.1 | 50.5 | 126.1 | 52.1 | 126.5 | 125.1 |
| | 69.3 | 113.2 | 62.8 | 114.7 | 65.0 | 114.4 | 113.7 |
| | 41.1 | 140.2 | 35.0 | 140.6 | 38.1 | 139.8 | 144.1 |
| | 49.4 | 132.3 | 42.6 | 133.6 | 45.9 | 132.4 | 135.9 |
| | 40.0 | 141.3 | 33.4 | 142.1 | 36.7 | 141.0 | 145.1 |
| | 76.7 | 106.0 | 72.6 | 105.6 | 73.3 | 106.5 | 107.2 |
| | 10.7 | 169.4 | 5.9 | 167.7 | 8.7 | 167.6 | 170.0 |
| | 57.2 | 124.8 | 50.8 | 125.9 | 52.3 | 126.3 | 125.2 |
| | 74.7 | 108.0 | 70.5 | 107.6 | 71.4 | 108.3 | 108.3 |
| | 37.5 | 143.7 | 30.9 | 144.5 | 34.4 | 143.3 | 145.1 |
| | 45.7 | 135.9 | 38.7 | 137.1 | 42.2 | 135.9 | 136.0 |
| | 40.4 | 140.9 | 34.6 | 141.0 | 37.2 | 140.6 | 144.2 |
| | 66.5 | 115.8 | 59.6 | 117.7 | 62.2 | 117.0 | 115.1 |
| | 67.5 | 114.9 | 60.9 | 116.5 | 63.2 | 116.0 | 113.9 |
| | 40.8 | 140.5 | 34.7 | 140.9 | 37.7 | 140.2 | 144.1 |
| | 49.3 | 132.4 | 42.5 | 133.6 | 45.7 | 132.5 | 136.0 |
| | 39.9 | 141.4 | 33.3 | 142.2 | 36.6 | 141.1 | 145.1 |
| | 75.9 | 106.8 | 71.7 | 106.4 | 72.5 | 107.2 | 107.2 |
| **17** | 117.8 | 66.6 | 114.8 | 66.3 | 116.2 | 65.9 | 70.8 |
| | 109.3 | 74.7 | 106.2 | 74.3 | 107.2 | 74.4 | 78.9 |
| | 94.2 | 89.3 | 91.4 | 88.0 | 92.0 | 88.8 | 93.7 |
| | 106.6 | 77.4 | 103.9 | 76.4 | 104.7 | 76.8 | 83.1 |
| | 112.0 | 72.2 | 108.5 | 72.1 | 109.4 | 72.4 | 77.8 |
| | 87.5 | 95.6 | 85.1 | 94.0 | 85.4 | 95.0 | 105.9 |
| | 111.1 | 73.0 | 107.7 | 72.9 | 108.9 | 72.9 | 77.6 |
| | 113.7 | 70.5 | 111.0 | 69.8 | 112.3 | 69.6 | 70.7 |
| | 108.4 | 75.6 | 105.6 | 74.9 | 106.7 | 74.9 | 77.7 |
| | 111.2 | 72.9 | 109.0 | 71.7 | 109.9 | 71.9 | 75.9 |
| | 3.6 | 176.2 | -1.3 | 174.4 | 1.7 | 174.1 | 175.8 |
| | 152.0 | 33.7 | 148.8 | 34.6 | 150.3 | 33.7 | 32.2 |
| | 152.8 | 33.0 | 150.7 | 32.9 | 151.4 | 32.6 | 32.2 |
| | 111.0 | 73.1 | 108.5 | 72.1 | 109.3 | 72.4 | 71.5 |
| | 112.5 | 71.7 | 107.0 | 73.6 | 109.1 | 72.6 | 75.4 |
| | 110.8 | 73.3 | 107.3 | 73.3 | 108.4 | 73.3 | 76.3 |
| | 157.9 | 28.1 | 155.9 | 28.1 | 156.5 | 27.8 | 29.3 |
| | 85.0 | 98.0 | 79.9 | 98.8 | 80.9 | 99.3 | 100.5 |
| | -39.1 | 217.2 | -45.6 | 215.6 | -43.1 | 216.6 | 213.7 |

| | | | | | | | |
|---|---|---|---|---|---|---|---|
| | 154.7 | 31.2 | 152.3 | 31.4 | 153.7 | 30.5 | 32.7 |
| | 115.5 | 68.8 | 112.7 | 68.3 | 113.2 | 68.7 | 70.9 |
| | 156.3 | 29.6 | 153.6 | 30.2 | 155.2 | 29.1 | 34.3 |
| | 118.8 | 65.7 | 117.4 | 63.9 | 117.6 | 64.6 | 63.4 |
| | 172.6 | 14.0 | 171.9 | 13.2 | 172.2 | 13.0 | 13.1 |
| | 14.7 | 165.6 | 9.9 | 164.0 | 12.4 | 164.0 | 167.8 |
| | 53.6 | 128.2 | 45.6 | 130.7 | 48.5 | 129.9 | 132.1 |
| | 51.1 | 130.6 | 45.3 | 131.0 | 47.1 | 131.3 | 130.8 |
| | 52.7 | 129.1 | 46.9 | 129.5 | 49.0 | 129.4 | 129.9 |
| | 46.8 | 134.7 | 41.1 | 134.9 | 43.2 | 134.9 | 134.4 |
| | 52.7 | 129.1 | 47.1 | 129.4 | 49.2 | 129.3 | 129.9 |
| | 51.4 | 130.4 | 45.8 | 130.6 | 47.7 | 130.7 | 130.8 |
| | 120.7 | 63.8 | 119.0 | 62.4 | 120.1 | 62.3 | 62.0 |
| | 123.5 | 61.1 | 122.2 | 59.4 | 123.3 | 59.2 | 62.3 |
| **18** | 28.1 | 152.7 | 23.7 | 151.1 | 25.8 | 151.4 | 142.8 |
| | 148.5 | 37.1 | 146.0 | 37.2 | 146.9 | 36.9 | 35.1 |
| | 108.5 | 75.5 | 104.1 | 76.3 | 105.0 | 76.6 | 76.3 |
| | 130.0 | 54.9 | 124.4 | 57.3 | 126.0 | 56.6 | 53.4 |
| | -32.9 | 211.3 | -38.6 | 209.1 | -36.2 | 210.0 | 204.3 |
| | 51.7 | 130.0 | 46.9 | 129.5 | 48.9 | 129.6 | 127.1 |
| | 109.8 | 74.3 | 107.7 | 72.9 | 108.5 | 73.2 | 72.3 |
| | 158.8 | 27.2 | 156.6 | 27.4 | 157.4 | 27.0 | 26.6 |
| | 150.7 | 35.0 | 147.5 | 35.8 | 148.2 | 35.6 | 38.2 |
| | 155.1 | 30.8 | 151.0 | 32.6 | 152.1 | 32.0 | 29.8 |
| | 79.8 | 103.1 | 76.3 | 102.1 | 76.4 | 103.5 | 106.8 |
| | -27.2 | 205.8 | -31.6 | 202.6 | -30.0 | 204.2 | 209.8 |
| | 127.6 | 57.2 | 123.5 | 58.2 | 124.0 | 58.5 | 53.8 |
| | 102.9 | 80.9 | 98.2 | 81.7 | 98.9 | 82.2 | 80.4 |
| | 105.3 | 78.6 | 100.5 | 79.6 | 101.8 | 79.5 | 78.5 |
| | 161.0 | 25.2 | 158.3 | 25.8 | 159.5 | 25.0 | 25.7 |
| | 160.8 | 25.3 | 158.5 | 25.6 | 159.1 | 25.4 | 24.7 |
| | 155.3 | 30.6 | 150.6 | 33.0 | 152.3 | 31.8 | 30.4 |
| | 133.3 | 51.7 | 130.3 | 51.9 | 130.4 | 52.5 | 49.3 |
| | 123.4 | 61.3 | 121.4 | 60.2 | 122.2 | 60.3 | 60.4 |
| | 149.6 | 36.1 | 147.1 | 36.2 | 147.8 | 36.1 | 31.2 |
| | 106.7 | 77.3 | 104.3 | 76.1 | 104.6 | 76.9 | 76.3 |
| | 101.8 | 82.0 | 97.7 | 82.2 | 98.2 | 83.0 | 80.6 |
| | 7.3 | 172.6 | 1.0 | 172.3 | 4.1 | 171.9 | 171.8 |
| | 8.1 | 171.9 | 2.1 | 171.3 | 5.1 | 171.0 | 167.3 |
| | 166.7 | 19.6 | 165.8 | 18.8 | 166.2 | 18.7 | 21.6 |
| | 155.6 | 30.3 | 154.7 | 29.1 | 155.2 | 29.0 | 24.4 |
| | 170.7 | 15.8 | 169.1 | 15.8 | 169.5 | 15.6 | 13.2 |
| **19** | 24.5 | 156.2 | 18.0 | 156.5 | 21.5 | 155.4 | 157.4 |
| | 89.7 | 93.6 | 85.5 | 93.5 | 86.7 | 93.8 | 96.5 |
| | 23.7 | 156.9 | 17.1 | 157.3 | 20.3 | 156.6 | 155.5 |
| | 85.9 | 97.2 | 79.8 | 98.9 | 81.2 | 99.0 | 100.3 |
| | 25.7 | 155.0 | 19.2 | 155.3 | 22.2 | 154.8 | 153.8 |
| | 85.2 | 97.9 | 79.8 | 98.9 | 81.0 | 99.2 | 106.7 |
| | 157.7 | 28.3 | 156.0 | 27.9 | 156.4 | 28.0 | 28.6 |
| | 125.2 | 59.5 | 121.7 | 59.9 | 123.1 | 59.4 | 66.1 |

| | | | | | | | |
|---|---|---|---|---|---|---|---|
| | 108.9 | 75.1 | 105.6 | 74.9 | 106.2 | 75.4 | 79.0 |
| | 53.5 | 128.4 | 47.2 | 129.2 | 48.8 | 129.7 | 130.4 |
| | 69.4 | 113.1 | 64.2 | 113.5 | 65.3 | 114.0 | 115.8 |
| | 58.9 | 123.2 | 53.6 | 123.3 | 55.5 | 123.3 | 120.3 |
| | 69.0 | 113.5 | 64.6 | 113.1 | 65.8 | 113.6 | 115.1 |
| | 39.8 | 141.5 | 32.8 | 142.7 | 36.2 | 141.6 | 145.1 |
| | 38.4 | 142.8 | 31.3 | 144.0 | 34.7 | 142.9 | 145.1 |
| | 154.2 | 31.7 | 150.9 | 32.7 | 151.4 | 32.7 | 36.5 |
| | 117.3 | 67.1 | 114.2 | 66.8 | 115.2 | 66.9 | 72.6 |
| | 82.5 | 100.5 | 76.3 | 102.2 | 77.8 | 102.2 | 100.3 |
| | 23.8 | 156.8 | 17.4 | 157.0 | 20.5 | 156.4 | 155.6 |
| | 105.7 | 78.3 | 103.2 | 77.1 | 103.6 | 77.9 | 76.6 |
| | 22.7 | 157.8 | 16.4 | 157.9 | 19.4 | 157.4 | 158.0 |
| | 89.1 | 94.2 | 85.5 | 93.5 | 86.5 | 94.0 | 96.3 |
| | 23.4 | 157.3 | 17.3 | 157.1 | 20.4 | 156.5 | 157.2 |
| | 88.9 | 94.3 | 85.8 | 93.3 | 86.2 | 94.2 | 95.9 |
| | 52.5 | 129.3 | 45.9 | 130.4 | 47.2 | 131.1 | 132.0 |
| | 65.6 | 116.7 | 60.2 | 117.1 | 62.3 | 116.9 | 119.0 |
| | 69.7 | 112.8 | 64.9 | 112.8 | 66.1 | 113.2 | 115.1 |
| | 37.7 | 143.5 | 30.4 | 144.9 | 34.1 | 143.5 | 145.1 |
| | 69.3 | 113.1 | 64.5 | 113.1 | 66.0 | 113.4 | 115.3 |
| | 39.4 | 141.8 | 32.5 | 143.0 | 35.9 | 141.8 | 145.1 |
| **20** | 123.5 | 61.1 | 121.0 | 60.5 | 121.1 | 61.3 | 60.1 |
| | 159.4 | 26.7 | 157.1 | 26.9 | 157.7 | 26.7 | 26.9 |
| | 154.0 | 31.9 | 150.3 | 33.2 | 151.3 | 32.7 | 31.7 |
| | 138.5 | 46.8 | 134.2 | 48.2 | 135.3 | 47.8 | 48.2 |
| | 123.4 | 61.2 | 120.1 | 61.4 | 119.8 | 62.5 | 60.1 |
| | 37.7 | 143.5 | 30.3 | 145.0 | 33.1 | 144.4 | 142.2 |
| | 64.6 | 117.6 | 59.2 | 118.0 | 60.7 | 118.4 | 116.3 |
| | 52.0 | 129.8 | 46.1 | 130.2 | 48.3 | 130.1 | 128.8 |
| | 57.1 | 124.9 | 51.3 | 125.4 | 53.3 | 125.4 | 124.4 |
| | 58.2 | 123.8 | 52.6 | 124.3 | 54.5 | 124.3 | 122.8 |
| | 46.6 | 135.0 | 39.1 | 136.8 | 41.2 | 136.8 | 132.9 |
| | 134.3 | 50.8 | 129.3 | 52.8 | 130.7 | 52.2 | 52.3 |
| | 141.7 | 43.7 | 140.2 | 42.6 | 140.8 | 42.7 | 42.7 |
| | 134.4 | 50.6 | 133.1 | 49.2 | 133.4 | 49.6 | 50.3 |
| | 132.9 | 52.1 | 131.1 | 51.1 | 131.1 | 51.8 | 52.9 |
| | 33.0 | 148.0 | 25.6 | 149.4 | 27.8 | 149.5 | 140.0 |
| | 49.0 | 132.7 | 45.3 | 131.0 | 47.0 | 131.3 | 127.9 |
| | 120.0 | 64.5 | 118.5 | 62.8 | 118.7 | 63.6 | 64.8 |
| | 106.3 | 77.6 | 103.6 | 76.7 | 104.2 | 77.2 | 77.6 |
| | 141.6 | 43.7 | 139.6 | 43.2 | 140.5 | 42.9 | 42.8 |
| | 8.7 | 171.3 | 3.1 | 170.3 | 5.6 | 170.5 | 170.3 |
| **21** | 54.0 | 127.9 | 48.1 | 128.4 | 50.4 | 128.2 | 128.0 |
| | 56.6 | 125.4 | 50.8 | 125.9 | 53.0 | 125.7 | 125.3 |
| | 59.1 | 123.0 | 53.7 | 123.1 | 55.7 | 123.1 | 122.3 |
| | 38.4 | 142.8 | 30.9 | 144.5 | 33.4 | 144.2 | 137.4 |
| | 41.4 | 139.9 | 34.5 | 141.1 | 37.1 | 140.7 | 141.4 |
| | 65.7 | 116.6 | 60.5 | 116.9 | 62.0 | 117.2 | 117.9 |
| | 132.3 | 52.6 | 126.6 | 55.3 | 128.5 | 54.3 | 52.8 |

| | | | | | | | |
|---|---|---|---|---|---|---|---|
| | 124.2 | 60.5 | 120.9 | 60.6 | 120.8 | 61.6 | 63.7 |
| | 8.8 | 171.3 | 4.5 | 169.0 | 7.0 | 169.2 | 167.7 |
| | 140.3 | 45.0 | 136.1 | 46.5 | 137.3 | 46.0 | 47.8 |
| | 132.0 | 53.0 | 129.1 | 53.0 | 129.1 | 53.7 | 57.5 |
| | 139.7 | 45.6 | 136.2 | 46.3 | 137.8 | 45.5 | 39.0 |
| | 141.4 | 43.9 | 139.4 | 43.4 | 140.0 | 43.4 | 36.5 |
| | 126.2 | 58.5 | 124.9 | 56.9 | 124.9 | 57.7 | 54.1 |
| | 117.4 | 66.9 | 114.7 | 66.4 | 115.0 | 67.1 | 66.3 |
| | 165.2 | 21.1 | 162.7 | 21.7 | 163.6 | 21.2 | 23.8 |
| | 156.7 | 29.3 | 153.3 | 30.4 | 154.2 | 30.0 | 29.3 |
| | 171.2 | 15.4 | 170.3 | 14.7 | 170.6 | 14.5 | 13.7 |
| | 59.1 | 123.0 | 55.4 | 121.6 | 57.3 | 121.6 | 117.7 |
| | 34.5 | 146.6 | 27.3 | 147.8 | 29.7 | 147.7 | 140.5 |
| | 126.8 | 58.0 | 125.2 | 56.6 | 124.9 | 57.7 | 52.3 |
| | 117.7 | 66.7 | 115.4 | 65.8 | 116.1 | 66.0 | 69.5 |
| | 136.3 | 48.8 | 132.6 | 49.7 | 133.7 | 49.3 | 47.9 |
| | 116.8 | 67.5 | 113.2 | 67.8 | 113.3 | 68.7 | 69.4 |
| | 29.4 | 151.5 | 22.6 | 152.2 | 25.3 | 151.9 | 150.0 |
| | 133.4 | 51.6 | 128.5 | 53.5 | 129.7 | 53.1 | 51.8 |
| | 46.8 | 134.8 | 39.6 | 136.4 | 41.5 | 136.5 | 134.3 |
| | 76.1 | 106.6 | 71.3 | 106.8 | 72.7 | 107.1 | 108.3 |
| | 54.2 | 127.6 | 48.4 | 128.2 | 50.3 | 128.2 | 128.2 |
| | 63.9 | 118.4 | 58.7 | 118.6 | 60.5 | 118.5 | 119.8 |
| | 59.0 | 123.0 | 53.5 | 123.4 | 55.5 | 123.3 | 122.2 |
| | 156.2 | 29.7 | 152.8 | 30.9 | 153.6 | 30.6 | 27.7 |
| | 156.2 | 29.7 | 154.6 | 29.3 | 154.8 | 29.4 | 28.5 |
| | 122.9 | 61.7 | 119.9 | 61.6 | 119.9 | 62.4 | 61.7 |
| | 131.0 | 53.9 | 129.4 | 52.7 | 129.8 | 53.1 | 53.7 |
| | 143.4 | 42.0 | 141.5 | 41.4 | 142.2 | 41.3 | 41.2 |
| | 126.3 | 58.4 | 124.8 | 57.0 | 124.4 | 58.2 | 58.1 |
| | 42.8 | 138.6 | 35.6 | 140.0 | 37.9 | 139.9 | 133.8 |
| | 57.8 | 124.2 | 53.8 | 123.1 | 55.9 | 122.9 | 122.2 |
| | 172.2 | 14.4 | 171.4 | 13.6 | 171.8 | 13.4 | 13.0 |
| **22** | 146.8 | 38.8 | 142.7 | 40.3 | 143.9 | 39.7 | 40.0 |
| | 37.1 | 144.1 | 31.6 | 143.8 | 33.3 | 144.3 | 143.4 |
| | 81.8 | 101.1 | 76.5 | 101.9 | 78.4 | 101.6 | 102.7 |
| | 55.8 | 126.2 | 48.6 | 128.0 | 50.3 | 128.2 | 129.9 |
| | 68.6 | 113.8 | 62.5 | 115.0 | 64.2 | 115.1 | 118.0 |
| | 48.5 | 133.1 | 41.7 | 134.4 | 43.8 | 134.4 | 136.5 |
| | 161.5 | 24.6 | 160.3 | 24.0 | 160.4 | 24.1 | 27.9 |
| | 156.7 | 29.2 | 155.9 | 28.0 | 156.1 | 28.2 | 27.3 |
| | 31.4 | 149.5 | 26.0 | 149.0 | 27.9 | 149.4 | 146.8 |
| | -7.4 | 186.8 | -13.7 | 185.9 | -11.1 | 186.3 | 188.3 |
| | 27.3 | 153.4 | 21.6 | 153.1 | 23.7 | 153.4 | 147.1 |
| | 46.6 | 134.9 | 40.4 | 135.5 | 42.4 | 135.7 | 135.2 |
| | 30.5 | 150.4 | 24.4 | 150.5 | 27.8 | 149.5 | 153.8 |
| | 62.7 | 119.5 | 57.8 | 119.3 | 59.5 | 119.6 | 119.7 |
| | 71.7 | 110.8 | 67.2 | 110.6 | 68.8 | 110.8 | 111.5 |
| | 74.0 | 108.6 | 68.9 | 109.0 | 70.4 | 109.2 | 108.3 |
| | -3.5 | 183.0 | -9.9 | 182.4 | -7.7 | 183.0 | 184.4 |

| | | | | | | | |
|---|---|---|---|---|---|---|---|
| | 157.3 | 28.7 | 155.2 | 28.7 | 155.8 | 28.5 | 28.8 |
| | 54.7 | 127.1 | 47.3 | 129.1 | 49.8 | 128.7 | 128.0 |
| | 62.7 | 119.5 | 57.3 | 119.8 | 59.5 | 119.5 | 119.6 |
| | 60.6 | 121.5 | 55.2 | 121.8 | 57.5 | 121.5 | 121.8 |
| | 46.8 | 134.8 | 40.0 | 135.9 | 42.4 | 135.7 | 137.3 |
| | 58.2 | 123.9 | 54.4 | 122.5 | 56.2 | 122.7 | 127.6 |
| | 65.8 | 116.5 | 62.2 | 115.3 | 64.3 | 115.0 | 111.6 |
| | 60.3 | 121.8 | 55.3 | 121.7 | 57.6 | 121.3 | 122.5 |
| | 61.1 | 121.1 | 55.9 | 121.1 | 57.8 | 121.2 | 120.8 |
| | 70.5 | 112.0 | 66.8 | 111.0 | 68.0 | 111.5 | 112.6 |
| | 42.7 | 138.7 | 36.1 | 139.6 | 38.8 | 139.0 | 133.4 |
| | 62.0 | 120.1 | 57.1 | 120.0 | 59.3 | 119.7 | 120.5 |
| | 59.4 | 122.7 | 54.6 | 122.3 | 56.7 | 122.2 | 122.6 |
| | 160.2 | 25.9 | 159.5 | 24.7 | 159.6 | 24.9 | 25.8 |
| | 170.1 | 16.4 | 168.4 | 16.4 | 168.9 | 16.1 | 17.8 |
| **23** | 139.9 | 45.4 | 137.2 | 45.4 | 137.7 | 45.6 | 46.9 |
| | 124.7 | 60.0 | 122.1 | 59.5 | 122.7 | 59.8 | 55.1 |
| | 153.5 | 32.3 | 151.2 | 32.4 | 152.1 | 32.0 | 30.2 |
| | 111.6 | 72.6 | 109.9 | 70.8 | 110.2 | 71.6 | 71.1 |
| | 10.1 | 170.0 | 5.8 | 167.8 | 8.7 | 167.5 | 171.6 |
| | 133.8 | 51.3 | 130.9 | 51.3 | 131.9 | 51.1 | 52.9 |
| | 146.3 | 39.2 | 144.9 | 38.3 | 145.4 | 38.4 | 41.4 |
| | 33.5 | 147.5 | 29.0 | 146.1 | 31.7 | 145.8 | 154.6 |
| | 133.1 | 51.9 | 130.4 | 51.8 | 131.6 | 51.4 | 49.3 |
| | 139.2 | 46.1 | 138.0 | 44.7 | 138.5 | 44.9 | 37.3 |
| | 152.1 | 33.7 | 149.7 | 33.8 | 150.4 | 33.6 | 23.7 |
| | 5.8 | 174.1 | 1.2 | 172.1 | 3.9 | 172.1 | 172.0 |
| | 15.2 | 165.1 | 10.5 | 163.4 | 13.1 | 163.4 | 168.1 |
| | 9.6 | 170.5 | 4.4 | 169.1 | 7.5 | 168.7 | 172.4 |
| | 133.0 | 52.0 | 130.3 | 51.8 | 130.9 | 52.1 | 55.4 |
| | 154.8 | 31.1 | 152.8 | 30.9 | 153.7 | 30.5 | 29.8 |
| | 74.8 | 107.8 | 69.5 | 108.5 | 71.9 | 107.8 | 105.9 |
| | 128.4 | 56.4 | 125.7 | 56.1 | 126.3 | 56.3 | 57.0 |
| | 4.0 | 175.8 | -0.2 | 173.4 | 2.6 | 173.3 | 173.6 |
| | 140.3 | 45.0 | 139.0 | 43.7 | 139.8 | 43.6 | 40.0 |
| | 123.9 | 60.7 | 122.4 | 59.2 | 123.4 | 59.1 | 63.3 |
| | 6.9 | 173.0 | 2.7 | 170.7 | 5.8 | 170.3 | 173.1 |
| | 119.2 | 65.2 | 117.8 | 63.5 | 118.6 | 63.6 | 60.0 |
| | 52.7 | 129.1 | 48.4 | 128.1 | 50.6 | 128.0 | 135.9 |
| | 30.9 | 150.0 | 26.3 | 148.7 | 29.4 | 147.9 | 157.5 |

[a] Parameters of linear regression models are presented in Table 2 in main text.
[b] For the references to the experimental works, please, see the main text.

**Table S4**. Calculated $^{13}$C NMR shielding constants, together with scaled and experimental chemical shifts – the GIAO-DFT(PBE0) calculations with the second-level basis sets.

| Mol. | pcS-2 | | pecS-2 | | pecS-2 mod | | $\delta_{exp}$[b] |
|---|---|---|---|---|---|---|---|
| | $\sigma$ | $\delta_{scaled}$[a] | $\sigma$ | $\delta_{scaled}$[a] | $\sigma$ | $\delta_{scaled}$[a] | |
| **1** | 121.3 | 59.3 | 120.9 | 59.0 | 121.2 | 59.0 | 55.5 |
| | 145.5 | 36.5 | 145.0 | 36.5 | 145.3 | 36.4 | 33.3 |
| | 136.9 | 44.6 | 136.2 | 44.6 | 136.5 | 44.7 | 35.9 |
| | 40.2 | 135.4 | 39.4 | 135.3 | 40.0 | 135.4 | 133.2 |
| | 126.3 | 54.6 | 125.9 | 54.3 | 126.2 | 54.3 | 54.6 |
| | 128.6 | 52.3 | 128.6 | 51.8 | 128.6 | 52.1 | 52.0 |
| | 33.3 | 141.9 | 32.4 | 141.9 | 33.0 | 142.0 | 135.2 |
| | 70.0 | 107.4 | 68.9 | 107.7 | 69.5 | 107.6 | 107.3 |
| | 160.7 | 22.2 | 160.2 | 22.2 | 160.6 | 22.0 | 19.5 |
| | 145.5 | 36.5 | 144.9 | 36.5 | 145.2 | 36.5 | 38.5 |
| | 49.4 | 126.7 | 48.3 | 127.0 | 48.9 | 126.9 | 128.1 |
| | 41.5 | 134.2 | 40.6 | 134.2 | 41.1 | 134.4 | 138.1 |
| | 58.0 | 118.7 | 57.2 | 118.7 | 57.7 | 118.7 | 124.5 |
| | 29.6 | 145.3 | 28.8 | 145.3 | 29.4 | 145.3 | 145.0 |
| | 59.6 | 117.1 | 58.7 | 117.3 | 59.3 | 117.2 | 118.6 |
| | 41.0 | 134.6 | 40.1 | 134.7 | 40.7 | 134.7 | 137.1 |
| | 67.5 | 109.7 | 66.5 | 110.0 | 67.1 | 109.8 | 112.2 |
| | 171.1 | 12.6 | 170.2 | 12.8 | 170.6 | 12.6 | 13.0 |
| | 49.6 | 126.5 | 48.4 | 126.9 | 49.0 | 126.9 | 130.1 |
| | 58.9 | 117.8 | 57.9 | 118.0 | 58.5 | 118.0 | 119.8 |
| | 56.9 | 119.7 | 56.0 | 119.8 | 56.5 | 119.8 | 122.5 |
| | 54.6 | 121.8 | 53.5 | 122.2 | 54.1 | 122.1 | 122.8 |
| | 42.5 | 133.2 | 41.6 | 133.2 | 42.1 | 133.4 | 136.9 |
| | 64.9 | 112.1 | 64.0 | 112.3 | 64.6 | 112.2 | 114.4 |
| | 37.4 | 137.9 | 36.8 | 137.7 | 37.4 | 137.8 | 137.2 |
| | 73.8 | 103.8 | 72.7 | 104.1 | 73.4 | 104.0 | 106.7 |
| | 64.8 | 112.2 | 63.9 | 112.4 | 64.5 | 112.4 | 113.6 |
| | 24.3 | 150.2 | 23.6 | 150.2 | 24.0 | 150.4 | 152.0 |
| | 60.4 | 116.4 | 59.3 | 116.7 | 59.8 | 116.7 | 119.3 |
| **2** | 49.6 | 126.5 | 48.4 | 126.9 | 49.2 | 126.7 | 125.7 |
| | 62.5 | 114.4 | 61.5 | 114.6 | 62.1 | 114.6 | 115.1 |
| | 53.4 | 122.9 | 52.8 | 122.7 | 53.4 | 122.8 | 124.1 |
| | 39.7 | 135.8 | 38.8 | 135.9 | 39.3 | 136.0 | 137.0 |
| | 66.1 | 111.1 | 65.0 | 111.4 | 65.7 | 111.2 | 111.4 |
| | 54.7 | 121.8 | 53.8 | 121.9 | 54.3 | 121.9 | 122.3 |
| | 57.7 | 118.9 | 56.7 | 119.1 | 57.3 | 119.1 | 119.9 |
| | 56.5 | 120.1 | 55.6 | 120.2 | 56.2 | 120.1 | 120.4 |
| | 111.3 | 68.6 | 110.5 | 68.7 | 111.1 | 68.5 | 68.2 |
| | 61.0 | 115.8 | 60.0 | 116.0 | 60.6 | 116.0 | 115.1 |
| | 50.7 | 125.5 | 49.6 | 125.7 | 50.3 | 125.7 | 125.7 |
| | 40.2 | 135.3 | 39.3 | 135.5 | 39.8 | 135.5 | 137.0 |
| | 50.1 | 126.0 | 49.6 | 125.8 | 50.1 | 125.9 | 124.1 |

| | | | | | | | |
|---|---|---|---|---|---|---|---|
| | 56.7 | 119.9 | 55.8 | 120.0 | 56.5 | 119.9 | 120.4 |
| | 58.0 | 118.6 | 57.0 | 118.8 | 57.6 | 118.8 | 119.9 |
| | 55.3 | 121.2 | 54.4 | 121.3 | 54.9 | 121.3 | 122.3 |
| | 67.2 | 110.0 | 66.2 | 110.3 | 66.8 | 110.2 | 111.4 |
| | -28.7 | 200.0 | -29.4 | 199.7 | -28.4 | 199.7 | 201.5 |
| | 56.2 | 120.3 | 55.1 | 120.7 | 55.7 | 120.6 | 120.8 |
| | 13.1 | 160.8 | 12.3 | 160.7 | 12.9 | 160.9 | 160.3 |
| | 50.5 | 125.7 | 49.5 | 125.8 | 50.1 | 125.9 | 125.5 |
| | 58.8 | 117.9 | 57.8 | 118.1 | 58.3 | 118.2 | 119.4 |
| | 37.4 | 138.0 | 36.5 | 138.1 | 36.9 | 138.2 | 137.6 |
| | 64.8 | 112.2 | 63.8 | 112.5 | 64.5 | 112.4 | 112.9 |
| **3** | 140.6 | 41.2 | 139.8 | 41.3 | 140.1 | 41.3 | 40.3 |
| | 122.6 | 58.1 | 122.2 | 57.8 | 122.5 | 57.8 | 64.8 |
| | 142.9 | 39.0 | 142.0 | 39.2 | 142.4 | 39.1 | 39.2 |
| | 108.1 | 71.6 | 107.4 | 71.6 | 107.6 | 71.8 | 72.2 |
| | 117.3 | 63.0 | 117.0 | 62.7 | 117.2 | 62.8 | 65.5 |
| | 139.8 | 41.9 | 139.3 | 41.8 | 139.5 | 41.8 | 41.7 |
| | 149.7 | 32.6 | 149.2 | 32.5 | 149.4 | 32.5 | 32.7 |
| | 28.1 | 146.7 | 27.3 | 146.6 | 27.8 | 146.8 | 138.6 |
| | -1.4 | 174.4 | -2.0 | 174.1 | -0.9 | 173.8 | 172.7 |
| | 42.3 | 133.3 | 41.7 | 133.2 | 42.2 | 133.3 | 137.9 |
| | 132.4 | 48.8 | 132.2 | 48.4 | 132.5 | 48.4 | 49.5 |
| | 140.6 | 41.2 | 140.1 | 41.0 | 140.3 | 41.1 | 41.6 |
| | 140.6 | 41.2 | 140.3 | 40.9 | 140.5 | 40.9 | 41.9 |
| | 122.0 | 58.6 | 121.8 | 58.1 | 122.0 | 58.3 | 52.9 |
| | 32.2 | 142.9 | 31.3 | 142.9 | 32.0 | 142.9 | 143.3 |
| | 160.3 | 22.6 | 159.6 | 22.7 | 159.9 | 22.6 | 21.1 |
| | 154.8 | 27.8 | 154.3 | 27.7 | 154.5 | 27.7 | 25.9 |
| | 158.3 | 24.5 | 157.9 | 24.4 | 158.1 | 24.3 | 24.5 |
| | 43.1 | 132.6 | 42.3 | 132.6 | 42.8 | 132.7 | 132.6 |
| | 153.9 | 28.6 | 153.5 | 28.4 | 153.6 | 28.5 | 26.5 |
| | 44.6 | 131.3 | 43.7 | 131.3 | 44.4 | 131.2 | 128.5 |
| | 150.5 | 31.9 | 149.8 | 32.0 | 150.0 | 31.9 | 25.9 |
| | 47.6 | 128.4 | 46.4 | 128.8 | 47.0 | 128.8 | 128.5 |
| | 151.6 | 30.8 | 151.0 | 30.8 | 151.3 | 30.7 | 25.8 |
| | 42.2 | 133.5 | 41.4 | 133.5 | 42.0 | 133.5 | 131.8 |
| | 37.2 | 138.2 | 36.6 | 137.9 | 37.2 | 138.0 | 137.8 |
| | 55.2 | 121.3 | 54.0 | 121.6 | 54.7 | 121.6 | 121.0 |
| | 63.7 | 113.3 | 62.7 | 113.5 | 63.3 | 113.4 | 113.7 |
| | 36.1 | 139.1 | 35.3 | 139.2 | 35.8 | 139.3 | 140.6 |
| | 15.3 | 158.7 | 14.4 | 158.7 | 14.9 | 158.9 | 148.1 |
| | 54.3 | 122.1 | 53.4 | 122.2 | 54.0 | 122.2 | 121.6 |
| | 66.3 | 110.9 | 65.3 | 111.1 | 65.9 | 111.0 | 111.9 |
| | 57.6 | 119.0 | 56.5 | 119.3 | 57.1 | 119.3 | 121.0 |
| | 57.6 | 119.0 | 56.6 | 119.2 | 57.1 | 119.2 | 119.5 |
| | 47.3 | 128.7 | 46.4 | 128.8 | 46.9 | 128.9 | 128.2 |
| | 8.2 | 165.4 | 7.3 | 165.4 | 8.2 | 165.2 | 167.0 |
| **4** | 11.7 | 162.1 | 11.2 | 161.8 | 11.8 | 161.8 | 161.4 |
| | 126.3 | 54.5 | 125.9 | 54.3 | 126.2 | 54.3 | 55.0 |
| | 4.6 | 168.7 | 4.1 | 168.4 | 4.8 | 168.4 | 168.2 |

| | | | | | | | |
|---|---|---|---|---|---|---|---|
| | 132.3 | 48.9 | 131.8 | 48.8 | 132.1 | 48.8 | 48.7 |
| | 125.9 | 54.9 | 125.3 | 54.9 | 125.4 | 55.1 | 52.4 |
| | 38.9 | 136.6 | 37.9 | 136.7 | 38.5 | 136.7 | 134.9 |
| | 52.5 | 123.8 | 51.7 | 123.8 | 52.3 | 123.8 | 122.6 |
| | 53.4 | 122.9 | 52.4 | 123.1 | 53.0 | 123.1 | 122.8 |
| | 44.3 | 131.5 | 43.4 | 131.6 | 43.9 | 131.7 | 130.0 |
| | 66.0 | 111.1 | 64.9 | 111.4 | 65.6 | 111.3 | 111.1 |
| | 30.3 | 144.6 | 29.7 | 144.5 | 30.2 | 144.6 | 144.8 |
| | 138.7 | 42.9 | 138.0 | 43.0 | 138.2 | 43.1 | 41.8 |
| | 92.4 | 86.3 | 92.0 | 86.1 | 92.4 | 86.1 | 87.3 |
| | 84.8 | 93.5 | 83.6 | 93.9 | 84.4 | 93.7 | 92.6 |
| | 151.4 | 31.0 | 150.6 | 31.1 | 150.8 | 31.2 | 31.1 |
| | 110.3 | 69.6 | 110.0 | 69.2 | 110.4 | 69.2 | 69.2 |
| | 145.4 | 36.6 | 145.0 | 36.5 | 145.0 | 36.6 | 35.8 |
| | 130.2 | 50.9 | 129.4 | 51.0 | 129.8 | 50.9 | 50.8 |
| | 102.3 | 77.1 | 102.0 | 76.7 | 102.3 | 76.8 | 76.2 |
| | 5.3 | 168.1 | 4.7 | 167.9 | 5.7 | 167.6 | 169.1 |
| | 129.6 | 51.5 | 129.1 | 51.3 | 129.2 | 51.5 | 51.7 |
| | 83.2 | 95.0 | 82.8 | 94.7 | 83.3 | 94.7 | 93.6 |
| | 130.9 | 50.2 | 130.8 | 49.7 | 130.9 | 49.9 | 48.6 |
| | 127.5 | 53.4 | 127.4 | 52.9 | 127.5 | 53.1 | 51.6 |
| | 150.3 | 32.0 | 149.8 | 32.0 | 150.1 | 31.8 | 30.6 |
| | 122.3 | 58.3 | 121.5 | 58.4 | 122.0 | 58.3 | 57.0 |
| | 35.7 | 139.6 | 34.8 | 139.7 | 35.4 | 139.7 | 136.6 |
| | 58.2 | 118.4 | 57.3 | 118.6 | 58.0 | 118.4 | 116.3 |
| | 53.5 | 122.9 | 52.7 | 122.9 | 53.1 | 123.0 | 121.6 |
| | 61.4 | 115.4 | 60.5 | 115.6 | 61.0 | 115.6 | 116.5 |
| | 34.7 | 140.5 | 33.9 | 140.5 | 34.5 | 140.5 | 142.0 |
| | 39.4 | 136.1 | 38.7 | 136.0 | 39.3 | 136.0 | 136.1 |
| | 155.3 | 27.4 | 154.8 | 27.3 | 155.0 | 27.2 | 25.5 |
| | 97.3 | 81.8 | 96.9 | 81.5 | 97.2 | 81.6 | 80.5 |
| | 150.0 | 32.3 | 149.2 | 32.4 | 149.4 | 32.5 | 31.2 |
| | 148.2 | 34.0 | 147.6 | 34.0 | 148.0 | 33.8 | 31.6 |
| | 113.3 | 66.8 | 112.9 | 66.5 | 113.2 | 66.5 | 65.1 |
| | 143.4 | 38.5 | 142.9 | 38.4 | 143.0 | 38.5 | 36.8 |
| | 135.9 | 45.5 | 135.2 | 45.6 | 135.4 | 45.7 | 46.3 |
| | 116.4 | 63.9 | 115.9 | 63.7 | 116.3 | 63.6 | 64.7 |
| | 149.8 | 32.5 | 149.2 | 32.5 | 149.5 | 32.4 | 29.3 |
| | 130.5 | 50.6 | 130.1 | 50.4 | 130.3 | 50.5 | 50.7 |
| | 14.7 | 159.3 | 14.3 | 158.9 | 14.9 | 159.0 | 158.7 |
| | 127.9 | 53.1 | 127.5 | 52.8 | 127.8 | 52.8 | 53.5 |
| | 13.5 | 160.4 | 13.1 | 160.0 | 13.6 | 160.2 | 158.7 |
| | 72.6 | 104.9 | 71.6 | 105.2 | 72.3 | 105.0 | 104.6 |
| | 118.3 | 62.1 | 117.6 | 62.1 | 117.7 | 62.3 | 60.9 |
| | 38.8 | 136.6 | 38.0 | 136.6 | 38.6 | 136.7 | 135.4 |
| | 49.5 | 126.6 | 48.7 | 126.6 | 49.3 | 126.6 | 124.4 |
| | 52.5 | 123.8 | 51.5 | 124.0 | 52.1 | 123.9 | 123.4 |
| | 43.9 | 131.9 | 43.0 | 132.0 | 43.5 | 132.1 | 131.2 |
| | 65.8 | 111.3 | 65.0 | 111.4 | 65.5 | 111.4 | 111.6 |
| | 31.1 | 143.9 | 30.5 | 143.7 | 31.0 | 143.8 | 144.7 |

| | | | | | | | |
|---|---|---|---|---|---|---|---|
| | 141.5 | 40.3 | 140.9 | 40.3 | 141.2 | 40.2 | 39.8 |
| | 92.9 | 85.9 | 92.4 | 85.7 | 92.8 | 85.7 | 87.1 |
| | 79.0 | 98.9 | 77.9 | 99.3 | 78.7 | 99.0 | 98.4 |
| | 153.3 | 29.2 | 152.5 | 29.4 | 152.8 | 29.3 | 28.2 |
| | 115.5 | 64.7 | 115.3 | 64.3 | 115.4 | 64.4 | 64.3 |
| | 145.1 | 36.9 | 144.6 | 36.8 | 144.9 | 36.7 | 37.0 |
| | 131.4 | 49.8 | 130.6 | 49.9 | 130.8 | 50.0 | 49.1 |
| | 107.1 | 72.6 | 106.7 | 72.3 | 106.9 | 72.4 | 72.1 |
| | 6.3 | 167.2 | 5.5 | 167.1 | 6.7 | 166.7 | 168.6 |
| | 129.6 | 51.5 | 129.1 | 51.3 | 129.2 | 51.5 | 51.9 |
| | 83.1 | 95.1 | 82.8 | 94.7 | 83.2 | 94.7 | 93.2 |
| | 130.8 | 50.3 | 130.6 | 49.9 | 130.8 | 50.0 | 48.6 |
| | 127.7 | 53.3 | 127.5 | 52.8 | 127.7 | 52.9 | 51.6 |
| | 148.8 | 33.4 | 148.3 | 33.3 | 148.7 | 33.1 | 31.4 |
| | 122.0 | 58.6 | 121.2 | 58.7 | 121.6 | 58.6 | 56.0 |
| | 36.5 | 138.8 | 35.6 | 138.9 | 36.3 | 138.9 | 135.9 |
| | 55.8 | 120.7 | 54.9 | 120.8 | 55.6 | 120.7 | 115.9 |
| | 53.3 | 123.0 | 52.5 | 123.0 | 53.0 | 123.1 | 120.9 |
| | 51.8 | 124.4 | 51.0 | 124.5 | 51.7 | 124.4 | 118.9 |
| | 36.4 | 138.9 | 35.6 | 138.9 | 36.3 | 138.9 | 142.0 |
| | 33.4 | 141.7 | 32.7 | 141.6 | 33.4 | 141.6 | 136.9 |
| | 155.2 | 27.4 | 154.7 | 27.3 | 155.0 | 27.2 | 25.5 |
| | 97.6 | 81.5 | 97.2 | 81.2 | 97.4 | 81.4 | 80.5 |
| | 149.0 | 33.3 | 148.2 | 33.5 | 148.6 | 33.3 | 32.3 |
| | 148.5 | 33.7 | 147.8 | 33.8 | 148.4 | 33.5 | 32.1 |
| | 113.9 | 66.2 | 113.5 | 65.9 | 113.8 | 66.0 | 64.8 |
| | 144.2 | 37.7 | 143.7 | 37.6 | 143.9 | 37.7 | 36.2 |
| | 135.9 | 45.6 | 135.2 | 45.6 | 135.4 | 45.6 | 46.1 |
| | 116.8 | 63.5 | 116.3 | 63.3 | 116.7 | 63.2 | 64.7 |
| | 145.7 | 36.4 | 145.0 | 36.4 | 145.4 | 36.2 | 36.0 |
| | 137.2 | 44.3 | 136.9 | 44.0 | 137.1 | 44.1 | 45.2 |
| **5** | 118.6 | 61.8 | 118.0 | 61.7 | 118.2 | 61.8 | 62.3 |
| | 117.5 | 62.8 | 116.9 | 62.8 | 117.1 | 62.9 | 63.8 |
| | 139.8 | 41.9 | 139.3 | 41.8 | 139.4 | 41.9 | 42.5 |
| | 91.4 | 87.3 | 91.0 | 87.0 | 91.0 | 87.4 | 88.0 |
| | 127.2 | 53.7 | 126.7 | 53.6 | 126.9 | 53.7 | 53.7 |
| | 137.9 | 43.6 | 137.5 | 43.5 | 137.4 | 43.8 | 44.6 |
| | 101.9 | 77.5 | 101.5 | 77.2 | 101.5 | 77.6 | 77.7 |
| | 148.1 | 34.1 | 147.5 | 34.0 | 147.8 | 34.0 | 35.9 |
| | 97.2 | 81.9 | 96.6 | 81.8 | 96.9 | 81.9 | 83.5 |
| | 124.3 | 56.5 | 123.7 | 56.4 | 123.8 | 56.6 | 57.5 |
| | 25.6 | 149.1 | 25.0 | 148.9 | 25.6 | 148.9 | 145.9 |
| | 132.3 | 48.9 | 131.7 | 48.8 | 132.0 | 48.9 | 49.5 |
| | 142.9 | 39.0 | 142.3 | 39.0 | 142.7 | 38.8 | 35.9 |
| | 39.6 | 135.9 | 38.9 | 135.8 | 39.4 | 135.9 | 133.7 |
| | 167.3 | 16.1 | 166.7 | 16.1 | 167.1 | 15.9 | 17.4 |
| | 156.9 | 25.9 | 156.3 | 25.9 | 156.6 | 25.8 | 25.0 |
| | 105.3 | 74.2 | 104.8 | 74.1 | 105.0 | 74.3 | 73.8 |
| | 157.5 | 25.3 | 156.9 | 25.3 | 157.2 | 25.2 | 27.6 |
| | 138.5 | 43.1 | 137.8 | 43.2 | 138.0 | 43.2 | 43.2 |

| | | | | | | | |
|---|---|---|---|---|---|---|---|
| | 97.8 | 81.3 | 97.3 | 81.1 | 97.6 | 81.2 | 82.2 |
| | 165.9 | 17.4 | 165.1 | 17.6 | 165.5 | 17.4 | 17.1 |
| | 136.6 | 44.9 | 136.0 | 44.8 | 136.4 | 44.7 | 42.1 |
| | -7.5 | 180.1 | -8.2 | 179.9 | -7.0 | 179.5 | 178.9 |
| | 131.9 | 49.3 | 131.2 | 49.3 | 131.5 | 49.3 | 50.1 |
| | 158.8 | 24.1 | 158.2 | 24.1 | 158.5 | 24.0 | 24.0 |
| | 153.6 | 28.9 | 153.0 | 28.9 | 153.4 | 28.7 | 30.0 |
| | 172.8 | 11.0 | 172.1 | 11.0 | 172.3 | 11.0 | 12.7 |
| | 138.9 | 42.7 | 138.2 | 42.8 | 138.5 | 42.8 | 43.5 |
| | -7.7 | 180.3 | -8.4 | 180.1 | -7.2 | 179.8 | 178.9 |
| | 172.9 | 10.8 | 172.3 | 10.8 | 172.5 | 10.8 | 12.9 |
| **6** | 40.0 | 135.5 | 39.1 | 135.6 | 39.6 | 135.7 | 135.3 |
| | 49.4 | 126.7 | 48.4 | 126.9 | 48.9 | 127.0 | 128.0 |
| | 48.0 | 128.0 | 47.1 | 128.2 | 47.6 | 128.2 | 127.4 |
| | 55.4 | 121.1 | 54.1 | 121.6 | 54.8 | 121.5 | 121.3 |
| | 29.7 | 145.2 | 28.9 | 145.1 | 29.4 | 145.3 | 146.3 |
| | 49.6 | 126.5 | 48.7 | 126.6 | 49.3 | 126.6 | 131.8 |
| | 11.5 | 162.3 | 10.7 | 162.2 | 11.6 | 162.1 | 162.2 |
| | 16.9 | 157.2 | 16.4 | 156.8 | 17.2 | 156.8 | 155.3 |
| | 43.1 | 132.6 | 42.4 | 132.5 | 42.9 | 132.6 | 133.7 |
| | 48.6 | 127.4 | 47.5 | 127.8 | 48.2 | 127.7 | 130.4 |
| | 7.8 | 165.7 | 7.2 | 165.5 | 8.2 | 165.3 | 168.4 |
| | 120.9 | 59.6 | 120.3 | 59.6 | 120.6 | 59.6 | 50.7 |
| | 47.0 | 128.9 | 46.2 | 129.0 | 46.6 | 129.1 | 128.6 |
| | 42.4 | 133.2 | 41.6 | 133.3 | 42.1 | 133.3 | 127.8 |
| | 47.8 | 128.2 | 47.0 | 128.2 | 47.5 | 128.3 | 129.3 |
| | 44.5 | 131.3 | 43.7 | 131.3 | 44.0 | 131.6 | 129.9 |
| | 137.2 | 44.3 | 136.5 | 44.4 | 136.8 | 44.3 | 42.9 |
| | 100.7 | 78.6 | 100.0 | 78.5 | 100.2 | 78.8 | 80.9 |
| | 38.0 | 137.4 | 37.1 | 137.5 | 37.7 | 137.5 | 139.6 |
| | 37.9 | 137.5 | 37.2 | 137.4 | 37.7 | 137.6 | 136.3 |
| | 92.2 | 86.5 | 92.1 | 86.0 | 92.3 | 86.2 | 86.8 |
| | 52.2 | 124.1 | 51.3 | 124.2 | 51.9 | 124.2 | 124.0 |
| | 52.7 | 123.6 | 51.7 | 123.8 | 52.3 | 123.8 | 126.0 |
| | 44.1 | 131.7 | 43.2 | 131.7 | 43.7 | 131.9 | 129.8 |
| | 59.6 | 117.1 | 58.7 | 117.3 | 59.2 | 117.3 | 115.9 |
| | 113.2 | 66.8 | 112.9 | 66.5 | 113.1 | 66.7 | 62.0 |
| | 3.0 | 170.3 | 2.4 | 170.0 | 3.5 | 169.7 | 171.4 |
| | 150.3 | 32.0 | 149.7 | 32.0 | 150.0 | 31.9 | 34.5 |
| | 157.2 | 25.5 | 156.8 | 25.4 | 156.8 | 25.5 | 25.2 |
| | 160.2 | 22.7 | 159.6 | 22.8 | 159.9 | 22.6 | 23.6 |
| | 160.8 | 22.2 | 160.2 | 22.2 | 160.4 | 22.2 | 21.4 |
| **7** | 105.3 | 74.2 | 105.0 | 73.9 | 105.2 | 74.1 | 73.8 |
| | 94.9 | 84.1 | 94.7 | 83.5 | 94.9 | 83.8 | 80.2 |
| | 103.5 | 76.0 | 102.8 | 75.9 | 103.0 | 76.2 | 73.6 |
| | 137.6 | 43.9 | 137.0 | 43.9 | 137.3 | 43.9 | 42.7 |
| | 45.4 | 130.4 | 44.6 | 130.5 | 45.0 | 130.6 | 126.7 |
| | 101.3 | 78.0 | 100.6 | 78.0 | 100.7 | 78.3 | 76.3 |
| | 13.4 | 160.5 | 12.8 | 160.3 | 13.8 | 160.0 | 163.1 |
| | 9.5 | 164.1 | 8.9 | 163.9 | 9.9 | 163.7 | 165.6 |

| | | | | | | | |
|---|---|---|---|---|---|---|---|
| | 26.2 | 148.5 | 25.6 | 148.3 | 26.1 | 148.4 | 148.4 |
| | 40.6 | 134.9 | 39.9 | 134.9 | 40.4 | 134.9 | 134.1 |
| | 48.9 | 127.2 | 48.2 | 127.1 | 48.8 | 127.1 | 127.3 |
| | 52.4 | 123.9 | 51.6 | 123.9 | 52.2 | 123.9 | 125.0 |
| | 119.0 | 61.4 | 118.4 | 61.4 | 118.6 | 61.5 | 60.4 |
| | 67.2 | 110.0 | 66.1 | 110.3 | 66.8 | 110.2 | 107.8 |
| | 46.0 | 129.9 | 45.0 | 130.1 | 45.6 | 130.1 | 130.4 |
| | 66.4 | 110.8 | 65.3 | 111.1 | 66.0 | 110.9 | 111.1 |
| | 158.5 | 24.3 | 158.0 | 24.2 | 158.1 | 24.3 | 27.5 |
| | 154.5 | 28.0 | 153.7 | 28.3 | 154.0 | 28.1 | 27.1 |
| | 57.1 | 119.5 | 56.1 | 119.7 | 56.7 | 119.6 | 120.3 |
| | 66.5 | 110.6 | 65.6 | 110.8 | 66.2 | 110.7 | 111.5 |
| | 100.8 | 78.4 | 100.1 | 78.5 | 100.3 | 78.7 | 74.9 |
| | 44.9 | 131.0 | 44.0 | 131.0 | 44.5 | 131.2 | 131.4 |
| | 52.1 | 124.1 | 51.3 | 124.2 | 51.9 | 124.2 | 119.2 |
| | 102.6 | 76.8 | 101.9 | 76.8 | 102.0 | 77.1 | 76.6 |
| | 54.7 | 121.8 | 53.7 | 122.0 | 54.3 | 121.9 | 122.8 |
| | 10.0 | 163.7 | 9.3 | 163.5 | 10.3 | 163.3 | 165.6 |
| | 58.2 | 118.5 | 57.2 | 118.7 | 57.8 | 118.7 | 120.6 |
| | 7.4 | 166.1 | 6.7 | 166.0 | 7.6 | 165.8 | 166.8 |
| | 157.8 | 25.0 | 157.4 | 24.8 | 157.5 | 24.9 | 28.3 |
| | 117.1 | 63.2 | 116.5 | 63.1 | 116.7 | 63.2 | 61.1 |
| | 159.8 | 23.2 | 159.3 | 23.0 | 159.4 | 23.1 | 27.5 |
| **8** | 141.1 | 40.6 | 140.5 | 40.7 | 140.7 | 40.7 | 40.5 |
| | 124.4 | 56.3 | 123.8 | 56.3 | 124.1 | 56.3 | 56.7 |
| | 117.2 | 63.1 | 116.8 | 62.8 | 117.1 | 62.8 | 62.9 |
| | 150.4 | 31.9 | 149.7 | 32.0 | 150.0 | 31.9 | 31.7 |
| | 98.3 | 80.9 | 98.0 | 80.5 | 98.1 | 80.7 | 81.1 |
| | 131.0 | 50.1 | 130.4 | 50.1 | 130.8 | 50.0 | 50.3 |
| | 150.3 | 32.1 | 149.8 | 32.0 | 150.0 | 31.9 | 32.2 |
| | 142.0 | 39.9 | 141.6 | 39.6 | 141.9 | 39.6 | 39.9 |
| | 139.9 | 41.8 | 139.3 | 41.8 | 139.5 | 41.8 | 42.0 |
| | 161.8 | 21.2 | 161.1 | 21.3 | 161.4 | 21.2 | 21.1 |
| | 144.3 | 37.7 | 143.5 | 37.8 | 143.8 | 37.8 | 37.2 |
| | 68.8 | 108.5 | 68.6 | 108.0 | 68.6 | 108.5 | 109.3 |
| | 167.7 | 15.7 | 167.1 | 15.7 | 167.2 | 15.7 | 16.3 |
| | 149.7 | 32.6 | 149.1 | 32.6 | 149.4 | 32.5 | 32.3 |
| | 29.7 | 145.2 | 28.8 | 145.3 | 29.3 | 145.4 | 140.8 |
| | 144.3 | 37.6 | 143.8 | 37.6 | 144.0 | 37.6 | 37.5 |
| | 170.0 | 13.5 | 169.4 | 13.5 | 169.7 | 13.5 | 15.0 |
| | 151.1 | 31.3 | 150.5 | 31.2 | 151.0 | 31.0 | 31.7 |
| | 52.6 | 123.8 | 51.9 | 123.6 | 52.4 | 123.7 | 121.8 |
| | 164.2 | 19.0 | 163.6 | 19.0 | 163.8 | 18.9 | 19.4 |
| | 140.3 | 41.4 | 139.6 | 41.5 | 140.1 | 41.3 | 39.0 |
| | 152.2 | 30.2 | 151.6 | 30.2 | 152.0 | 30.1 | 30.4 |
| | 107.5 | 72.2 | 107.2 | 71.9 | 107.4 | 72.0 | 78.2 |
| | 153.6 | 29.0 | 153.1 | 28.8 | 153.4 | 28.7 | 29.3 |
| | 151.4 | 31.0 | 151.1 | 30.7 | 151.1 | 30.9 | 30.6 |
| | 114.0 | 66.1 | 113.8 | 65.7 | 113.9 | 65.9 | 66.9 |
| | 167.3 | 16.1 | 166.8 | 16.0 | 167.0 | 15.9 | 17.3 |

| | | | | | | | |
|---|---|---|---|---|---|---|---|
| | 76.8 | 101.0 | 76.6 | 100.5 | 76.9 | 100.7 | 102.5 |
| | 109.6 | 70.2 | 109.0 | 70.1 | 109.2 | 70.3 | 71.8 |
| | 102.5 | 76.9 | 101.9 | 76.8 | 102.3 | 76.8 | 78.2 |
| | 103.2 | 76.2 | 103.0 | 75.8 | 103.2 | 75.9 | 78.2 |
| | 107.4 | 72.2 | 107.1 | 71.9 | 107.3 | 72.1 | 75.5 |
| | 117.2 | 63.1 | 116.8 | 62.9 | 116.9 | 63.1 | 62.7 |
| **9** | 141.8 | 40.0 | 140.9 | 40.2 | 141.5 | 40.0 | 40.1 |
| | 119.6 | 60.9 | 118.8 | 61.0 | 119.3 | 60.8 | 60.9 |
| | 138.1 | 43.4 | 137.2 | 43.7 | 137.6 | 43.6 | 44.0 |
| | 129.7 | 51.4 | 128.9 | 51.5 | 129.3 | 51.4 | 51.6 |
| | 165.4 | 17.8 | 164.7 | 18.0 | 165.1 | 17.7 | 17.3 |
| | 144.8 | 37.2 | 144.2 | 37.2 | 144.4 | 37.2 | 37.4 |
| | 104.4 | 75.1 | 103.7 | 75.1 | 104.2 | 75.0 | 76.1 |
| | 142.7 | 39.1 | 141.9 | 39.3 | 142.3 | 39.1 | 37.9 |
| | 126.6 | 54.3 | 125.8 | 54.4 | 126.3 | 54.2 | 53.5 |
| | 141.0 | 40.8 | 140.0 | 41.1 | 140.5 | 40.9 | 38.5 |
| | 133.8 | 47.5 | 133.0 | 47.6 | 133.3 | 47.7 | 42.6 |
| | 153.0 | 29.5 | 152.2 | 29.7 | 152.5 | 29.6 | 30.0 |
| | 144.7 | 37.2 | 144.2 | 37.2 | 144.4 | 37.2 | 37.3 |
| | 154.4 | 28.2 | 153.7 | 28.3 | 154.1 | 28.1 | 32.1 |
| | 151.9 | 30.5 | 151.3 | 30.5 | 151.5 | 30.5 | 35.5 |
| | 156.1 | 26.6 | 155.3 | 26.8 | 155.4 | 26.9 | 27.8 |
| | 149.5 | 32.8 | 149.0 | 32.7 | 149.2 | 32.6 | 32.2 |
| | 151.9 | 30.6 | 151.5 | 30.4 | 151.5 | 30.5 | 35.0 |
| | 149.5 | 32.8 | 148.7 | 32.9 | 149.3 | 32.6 | 31.1 |
| | 112.3 | 67.7 | 111.6 | 67.7 | 111.8 | 67.9 | 68.7 |
| | -4.9 | 177.7 | -5.6 | 177.5 | -4.3 | 177.0 | 176.8 |
| | 97.2 | 81.8 | 96.8 | 81.6 | 96.8 | 81.9 | 83.8 |
| | 168.5 | 15.0 | 167.8 | 15.0 | 168.1 | 14.9 | 15.5 |
| | 171.5 | 12.2 | 170.7 | 12.3 | 170.9 | 12.3 | 12.8 |
| | 168.5 | 14.9 | 168.0 | 14.9 | 168.2 | 14.8 | 13.1 |
| | 166.8 | 16.6 | 166.1 | 16.7 | 166.4 | 16.6 | 21.5 |
| | 146.8 | 35.3 | 146.1 | 35.4 | 146.4 | 35.4 | 30.6 |
| | 150.8 | 31.6 | 150.2 | 31.6 | 150.5 | 31.4 | 30.0 |
| | 148.2 | 34.0 | 147.6 | 34.0 | 147.9 | 33.9 | 34.1 |
| | -6.5 | 179.1 | -7.2 | 179.0 | -5.9 | 178.6 | 178.0 |
| **10** | 125.8 | 55.0 | 125.3 | 54.8 | 125.5 | 55.0 | 57.9 |
| | 158.5 | 24.3 | 157.7 | 24.5 | 158.1 | 24.3 | 25.2 |
| | 147.9 | 34.3 | 147.3 | 34.3 | 147.4 | 34.4 | 30.5 |
| | 140.2 | 41.5 | 139.7 | 41.3 | 140.0 | 41.3 | 44.1 |
| | 132.8 | 48.4 | 132.5 | 48.2 | 132.5 | 48.3 | 50.5 |
| | 66.5 | 110.6 | 65.5 | 110.9 | 66.1 | 110.8 | 110.6 |
| | 2.9 | 170.4 | 2.2 | 170.2 | 3.2 | 170.0 | 171.3 |
| | 106.9 | 72.7 | 106.6 | 72.4 | 106.7 | 72.7 | 71.7 |
| | 143.1 | 38.8 | 142.4 | 38.9 | 142.5 | 39.0 | 40.8 |
| | 134.7 | 46.7 | 134.0 | 46.8 | 134.3 | 46.7 | 42.6 |
| | 133.9 | 47.4 | 133.4 | 47.3 | 133.4 | 47.5 | 47.1 |
| | -3.8 | 176.6 | -4.3 | 176.3 | -3.3 | 176.1 | 174.9 |
| | 43.6 | 132.1 | 42.8 | 132.2 | 43.4 | 132.2 | 134.1 |
| | 36.2 | 139.1 | 35.6 | 138.9 | 36.2 | 139.0 | 130.6 |

| | | | | | | | |
|---|---|---|---|---|---|---|---|
| | 141.3 | 40.5 | 140.5 | 40.6 | 140.9 | 40.5 | 42.1 |
| | 49.0 | 127.1 | 47.9 | 127.4 | 48.6 | 127.3 | 128.5 |
| | -3.0 | 175.9 | -3.7 | 175.7 | -2.7 | 175.5 | 177.9 |
| | 161.1 | 21.9 | 160.6 | 21.8 | 160.8 | 21.7 | 22.0 |
| | 152.8 | 29.7 | 152.4 | 29.5 | 152.5 | 29.5 | 31.1 |
| | 40.8 | 134.8 | 39.6 | 135.1 | 40.2 | 135.2 | 132.7 |
| | 51.8 | 124.4 | 51.2 | 124.2 | 51.8 | 124.3 | 124.2 |
| | 40.0 | 135.5 | 39.2 | 135.5 | 39.7 | 135.6 | 138.1 |
| | 164.4 | 18.8 | 163.8 | 18.8 | 164.1 | 18.7 | 19.6 |
| | 172.4 | 11.3 | 171.8 | 11.4 | 172.0 | 11.3 | 20.0 |
| | 1.6 | 171.6 | 0.9 | 171.4 | 2.0 | 171.1 | 172.1 |
| | 167.9 | 15.5 | 167.2 | 15.7 | 167.5 | 15.5 | 18.5 |
| | 163.7 | 19.5 | 163.1 | 19.5 | 163.3 | 19.5 | 18.2 |
| | 57.3 | 119.3 | 56.3 | 119.5 | 56.9 | 119.5 | 119.3 |
| | 66.2 | 110.9 | 65.2 | 111.1 | 65.9 | 111.0 | 112.3 |
| | 46.6 | 129.4 | 45.7 | 129.4 | 46.2 | 129.5 | 128.2 |
| | 48.5 | 127.6 | 47.6 | 127.6 | 48.1 | 127.7 | 128.2 |
| | 57.8 | 118.8 | 56.9 | 119.0 | 57.4 | 119.0 | 119.8 |
| | 55.4 | 121.1 | 54.5 | 121.2 | 55.0 | 121.2 | 122.4 |
| | 64.0 | 113.0 | 63.0 | 113.3 | 63.5 | 113.3 | 116.2 |
| | 62.6 | 114.4 | 61.5 | 114.7 | 62.0 | 114.6 | 116.2 |
| | 18.4 | 155.8 | 17.6 | 155.7 | 18.1 | 155.9 | 157.7 |
| **11** | 130.6 | 50.5 | 130.4 | 50.1 | 130.3 | 50.5 | 49.4 |
| | 10.0 | 163.7 | 9.0 | 163.8 | 9.8 | 163.8 | 169.4 |
| | 61.8 | 115.0 | 60.6 | 115.5 | 61.2 | 115.4 | 106.6 |
| | 34.6 | 140.6 | 33.7 | 140.6 | 34.2 | 140.8 | 141.5 |
| | 149.6 | 32.7 | 148.9 | 32.8 | 149.1 | 32.7 | 36.9 |
| | 14.6 | 159.4 | 13.9 | 159.2 | 14.6 | 159.3 | 165.3 |
| | 58.6 | 118.1 | 57.4 | 118.4 | 58.1 | 118.4 | 124.5 |
| | 17.5 | 156.6 | 16.7 | 156.6 | 17.3 | 156.7 | 158.1 |
| | 68.5 | 108.8 | 67.4 | 109.1 | 68.0 | 109.0 | 117.3 |
| | 53.0 | 123.4 | 51.9 | 123.6 | 52.6 | 123.5 | 124.4 |
| | 38.7 | 136.8 | 37.9 | 136.8 | 38.5 | 136.8 | 134.8 |
| | 56.0 | 120.5 | 54.9 | 120.9 | 55.5 | 120.8 | 120.6 |
| | 25.2 | 149.4 | 24.4 | 149.4 | 25.0 | 149.5 | 151.8 |
| | 66.1 | 111.1 | 64.8 | 111.5 | 65.5 | 111.4 | 115.2 |
| | 42.1 | 133.6 | 41.0 | 133.8 | 41.7 | 133.8 | 137.2 |
| | 58.4 | 118.3 | 57.5 | 118.4 | 58.1 | 118.4 | 120.5 |
| | 36.5 | 138.8 | 35.5 | 139.0 | 36.0 | 139.1 | 136.9 |
| | 72.5 | 105.0 | 71.5 | 105.3 | 72.0 | 105.2 | 107.9 |
| | 18.1 | 156.1 | 17.4 | 156.0 | 17.9 | 156.1 | 157.8 |
| | 55.6 | 120.9 | 54.4 | 121.3 | 55.0 | 121.2 | 120.6 |
| | 42.6 | 133.0 | 41.8 | 133.1 | 42.5 | 133.0 | 135.0 |
| | 48.9 | 127.2 | 47.9 | 127.4 | 48.5 | 127.4 | 124.5 |
| | 39.7 | 135.8 | 38.8 | 135.9 | 39.4 | 135.9 | 136.6 |
| | 62.2 | 114.7 | 60.9 | 115.2 | 61.6 | 115.0 | 114.9 |
| | 22.4 | 152.1 | 21.7 | 152.0 | 22.2 | 152.1 | 152.5 |
| | 15.4 | 158.6 | 14.8 | 158.4 | 15.3 | 158.6 | 157.8 |
| | 73.1 | 104.5 | 71.9 | 104.9 | 72.5 | 104.8 | 107.7 |
| | 36.5 | 138.8 | 35.6 | 138.9 | 36.1 | 139.0 | 136.8 |

| | | | | | | | |
|---|---|---|---|---|---|---|---|
| | 61.3 | 115.6 | 60.3 | 115.8 | 60.9 | 115.7 | 119.3 |
| | 57.6 | 119.0 | 56.5 | 119.4 | 57.1 | 119.3 | 118.9 |
| | 20.9 | 153.5 | 20.2 | 153.3 | 20.7 | 153.5 | 155.9 |
| | 81.4 | 96.7 | 80.3 | 97.1 | 80.8 | 97.0 | 101.7 |
| | 21.5 | 152.9 | 20.7 | 152.8 | 21.3 | 153.0 | 155.5 |
| | 60.6 | 116.2 | 59.4 | 116.6 | 60.1 | 116.5 | 116.4 |
| | 36.0 | 139.3 | 35.2 | 139.3 | 35.6 | 139.5 | 134.7 |
| | 147.5 | 34.6 | 146.8 | 34.7 | 147.2 | 34.5 | 34.7 |
| | 139.8 | 41.9 | 139.5 | 41.5 | 139.6 | 41.7 | 44.0 |
| | 134.2 | 47.2 | 133.7 | 47.0 | 133.7 | 47.2 | 48.9 |
| | 169.2 | 14.3 | 168.6 | 14.4 | 168.7 | 14.4 | 19.2 |
| | 121.1 | 59.5 | 120.8 | 59.1 | 121.0 | 59.2 | 61.0 |
| | 161.8 | 21.3 | 160.9 | 21.5 | 161.3 | 21.3 | 22.1 |
| | 127.6 | 53.3 | 127.2 | 53.1 | 127.4 | 53.2 | 56.9 |
| | 128.3 | 52.7 | 128.0 | 52.4 | 128.1 | 52.5 | 56.9 |
| | 161.9 | 21.1 | 161.2 | 21.3 | 161.6 | 21.1 | 22.2 |
| | 163.9 | 19.3 | 163.2 | 19.3 | 163.5 | 19.3 | 19.7 |
| | 161.9 | 21.2 | 161.4 | 21.1 | 161.6 | 21.0 | 20.8 |
| **12** | 117.2 | 63.1 | 116.5 | 63.1 | 117.0 | 63.0 | 65.7 |
| | 127.9 | 53.0 | 127.0 | 53.3 | 127.3 | 53.3 | 51.3 |
| | 131.8 | 49.4 | 131.0 | 49.5 | 131.3 | 49.5 | 48.1 |
| | 135.1 | 46.3 | 134.3 | 46.4 | 134.7 | 46.4 | 45.9 |
| | 144.1 | 37.8 | 143.3 | 38.1 | 143.5 | 38.0 | 37.9 |
| | 128.9 | 52.1 | 128.3 | 52.1 | 128.6 | 52.1 | 53.9 |
| | 119.4 | 61.1 | 118.7 | 61.1 | 119.0 | 61.0 | 60.6 |
| | 163.8 | 19.4 | 163.1 | 19.4 | 163.4 | 19.3 | 18.9 |
| | 147.9 | 34.3 | 147.4 | 34.2 | 147.6 | 34.2 | 30.8 |
| | 100.4 | 78.9 | 100.0 | 78.5 | 100.1 | 78.8 | 79.1 |
| | -40.1 | 210.7 | -41.1 | 210.7 | -40.1 | 210.7 | 206.1 |
| | 145.2 | 36.8 | 144.3 | 37.1 | 144.8 | 36.9 | 36.4 |
| | 99.4 | 79.8 | 98.9 | 79.6 | 99.1 | 79.8 | 80.3 |
| | 103.0 | 76.4 | 102.3 | 76.4 | 102.7 | 76.4 | 77.8 |
| | 167.4 | 15.9 | 166.6 | 16.2 | 166.9 | 16.1 | 17.6 |
| | 115.5 | 64.7 | 114.9 | 64.6 | 115.2 | 64.7 | 65.3 |
| | 162.0 | 21.0 | 161.4 | 21.1 | 161.6 | 21.0 | 21.4 |
| | 6.2 | 167.3 | 5.5 | 167.1 | 6.7 | 166.7 | 166.6 |
| | 146.7 | 35.4 | 146.0 | 35.5 | 146.2 | 35.5 | 35.6 |
| | 164.4 | 18.8 | 163.7 | 18.9 | 163.9 | 18.9 | 20.7 |
| | 155.2 | 27.4 | 154.5 | 27.5 | 154.8 | 27.4 | 30.2 |
| | 54.5 | 121.9 | 53.5 | 122.2 | 54.1 | 122.1 | 120.0 |
| | 3.9 | 169.4 | 3.1 | 169.3 | 4.2 | 169.0 | 169.1 |
| | 65.6 | 111.5 | 64.6 | 111.7 | 65.1 | 111.7 | 109.7 |
| | 35.5 | 139.7 | 34.8 | 139.6 | 35.5 | 139.6 | 141.1 |
| | 32.9 | 142.2 | 32.2 | 142.1 | 32.8 | 142.2 | 143.2 |
| **13** | 117.1 | 63.2 | 116.7 | 62.9 | 117.0 | 63.0 | 62.9 |
| | 129.4 | 51.6 | 128.9 | 51.5 | 128.9 | 51.7 | 53.5 |
| | 138.2 | 43.4 | 137.6 | 43.3 | 137.9 | 43.3 | 41.4 |
| | 93.7 | 85.2 | 93.5 | 84.6 | 93.7 | 84.8 | 82.7 |
| | 116.3 | 63.9 | 116.1 | 63.5 | 116.2 | 63.7 | 65.4 |
| | 154.8 | 27.8 | 154.1 | 27.9 | 154.4 | 27.8 | 29.2 |

| | | | | | | | |
|---|---|---|---|---|---|---|---|
| | 158.6 | 24.2 | 158.1 | 24.1 | 158.2 | 24.3 | 23.3 |
| | 141.4 | 40.3 | 141.2 | 40.0 | 141.3 | 40.1 | 37.3 |
| | 31.4 | 143.6 | 30.5 | 143.7 | 31.1 | 143.7 | 133.9 |
| | 22.6 | 151.8 | 22.0 | 151.7 | 22.6 | 151.7 | 146.5 |
| | 123.0 | 57.6 | 122.8 | 57.2 | 122.9 | 57.4 | 53.8 |
| | 24.1 | 150.5 | 23.3 | 150.4 | 23.9 | 150.5 | 141.5 |
| | 122.8 | 57.8 | 122.3 | 57.7 | 122.6 | 57.7 | 52.0 |
| | 54.4 | 122.0 | 53.6 | 122.1 | 54.2 | 122.0 | 122.7 |
| | 69.3 | 108.0 | 68.4 | 108.2 | 68.9 | 108.1 | 103.8 |
| | 56.8 | 119.8 | 56.0 | 119.8 | 56.7 | 119.7 | 117.7 |
| | 57.6 | 119.0 | 56.7 | 119.2 | 57.2 | 119.2 | 117.2 |
| | 50.3 | 125.9 | 49.3 | 126.0 | 49.9 | 126.1 | 128.2 |
| | 168.9 | 14.6 | 168.1 | 14.8 | 168.4 | 14.6 | 13.3 |
| **14** | 145.8 | 36.3 | 145.0 | 36.4 | 145.4 | 36.2 | 35.1 |
| | 153.6 | 29.0 | 152.8 | 29.1 | 153.0 | 29.1 | 28.5 |
| | 146.6 | 35.5 | 145.7 | 35.7 | 146.1 | 35.6 | 37.2 |
| | 77.3 | 100.6 | 76.9 | 100.2 | 77.2 | 100.4 | 102.6 |
| | 147.5 | 34.7 | 146.9 | 34.7 | 147.2 | 34.6 | 36.2 |
| | 59.9 | 116.9 | 58.9 | 117.1 | 59.4 | 117.1 | 116.8 |
| | 66.1 | 111.0 | 64.9 | 111.4 | 65.7 | 111.2 | 117.5 |
| | 57.7 | 118.9 | 56.9 | 119.0 | 57.5 | 118.9 | 122.9 |
| | 33.6 | 141.6 | 32.7 | 141.6 | 33.2 | 141.8 | 133.7 |
| | 62.6 | 114.4 | 61.4 | 114.7 | 62.2 | 114.5 | 113.4 |
| | 21.6 | 152.8 | 21.0 | 152.6 | 21.4 | 152.8 | 154.5 |
| | 21.0 | 153.3 | 20.5 | 153.0 | 20.9 | 153.3 | 155.0 |
| | 18.3 | 155.9 | 17.5 | 155.8 | 18.1 | 156.0 | 156.7 |
| | 48.2 | 127.8 | 47.3 | 127.9 | 47.8 | 128.0 | 127.9 |
| | 159.4 | 23.5 | 158.7 | 23.6 | 159.1 | 23.4 | 23.9 |
| | 72.7 | 104.8 | 71.6 | 105.1 | 72.4 | 104.9 | 105.0 |
| | 77.0 | 100.8 | 75.8 | 101.2 | 76.4 | 101.1 | 104.6 |
| | 17.4 | 156.7 | 16.7 | 156.6 | 17.3 | 156.7 | 159.9 |
| | 44.3 | 131.4 | 43.4 | 131.6 | 44.0 | 131.6 | 130.3 |
| | 75.0 | 102.7 | 73.9 | 103.0 | 74.5 | 102.9 | 105.4 |
| | 45.8 | 130.1 | 44.7 | 130.4 | 45.3 | 130.4 | 131.1 |
| | 71.3 | 106.2 | 70.1 | 106.6 | 70.8 | 106.4 | 109.9 |
| | 18.0 | 156.2 | 17.3 | 156.0 | 17.8 | 156.3 | 157.9 |
| | 76.9 | 100.9 | 75.7 | 101.4 | 76.3 | 101.2 | 103.9 |
| | 73.1 | 104.5 | 71.9 | 104.9 | 72.6 | 104.7 | 107.1 |
| | 15.7 | 158.3 | 15.0 | 158.2 | 15.5 | 158.4 | 157.7 |
| | 20.6 | 153.7 | 19.9 | 153.6 | 20.5 | 153.7 | 156.7 |
| | 76.4 | 101.4 | 75.3 | 101.7 | 76.0 | 101.5 | 102.2 |
| | 54.5 | 121.9 | 53.3 | 122.3 | 53.9 | 122.3 | 122.5 |
| | 20.0 | 154.3 | 19.3 | 154.2 | 19.8 | 154.4 | 153.3 |
| | 54.6 | 121.9 | 53.6 | 122.0 | 54.2 | 122.0 | 122.0 |
| | 82.9 | 95.2 | 81.7 | 95.7 | 82.4 | 95.5 | 98.4 |
| | 66.7 | 110.5 | 65.7 | 110.7 | 66.2 | 110.7 | 113.4 |
| | 19.4 | 154.8 | 18.7 | 154.7 | 19.2 | 155.0 | 157.5 |
| **15** | 138.1 | 43.5 | 137.3 | 43.6 | 137.6 | 43.5 | 45.9 |
| | 85.4 | 92.9 | 85.0 | 92.7 | 85.3 | 92.8 | 89.4 |
| | 90.9 | 87.8 | 90.3 | 87.7 | 90.7 | 87.7 | 84.6 |

| | | | | | | | |
|---|---|---|---|---|---|---|---|
| | 134.7 | 46.7 | 133.9 | 46.8 | 134.3 | 46.7 | 46.8 |
| | 92.3 | 86.4 | 91.8 | 86.3 | 92.1 | 86.4 | 76.5 |
| | 171.6 | 12.1 | 170.9 | 12.2 | 171.1 | 12.1 | 12.1 |
| | 2.1 | 171.1 | 1.4 | 170.9 | 2.6 | 170.6 | 170.5 |
| | 45.8 | 130.1 | 44.6 | 130.4 | 45.2 | 130.5 | 130.0 |
| | 44.0 | 131.8 | 43.0 | 131.9 | 43.6 | 132.0 | 130.0 |
| | 46.6 | 129.3 | 45.6 | 129.5 | 46.2 | 129.6 | 128.5 |
| | 43.5 | 132.3 | 42.5 | 132.4 | 43.0 | 132.5 | 133.1 |
| | 1.9 | 171.3 | 1.2 | 171.1 | 2.3 | 170.8 | 169.7 |
| | 159.2 | 23.7 | 158.3 | 24.0 | 158.6 | 23.9 | 20.6 |
| | 2.6 | 170.6 | 2.1 | 170.3 | 3.1 | 170.0 | 166.2 |
| | 162.1 | 20.9 | 161.2 | 21.2 | 161.7 | 21.0 | 22.1 |
| | 103.0 | 76.4 | 102.5 | 76.2 | 102.9 | 76.2 | 71.2 |
| | 44.0 | 131.8 | 43.1 | 131.9 | 43.7 | 131.9 | 133.9 |
| | 158.8 | 24.1 | 158.0 | 24.3 | 158.4 | 24.1 | 20.5 |
| | 42.7 | 133.0 | 42.0 | 132.9 | 42.6 | 132.9 | 124.1 |
| | 1.1 | 172.1 | 0.5 | 171.8 | 1.6 | 171.5 | 169.0 |
| | 164.3 | 18.9 | 163.3 | 19.3 | 163.7 | 19.0 | 13.7 |
| | 143.5 | 38.4 | 142.8 | 38.5 | 143.1 | 38.4 | 39.9 |
| | 145.3 | 36.7 | 144.5 | 36.9 | 144.8 | 36.8 | 39.2 |
| | 92.2 | 86.5 | 91.9 | 86.2 | 92.1 | 86.3 | 74.3 |
| | 158.4 | 24.4 | 157.8 | 24.5 | 158.1 | 24.3 | 24.1 |
| | 156.9 | 25.9 | 156.2 | 25.9 | 156.6 | 25.8 | 25.5 |
| | 2.9 | 170.3 | 2.2 | 170.1 | 3.3 | 169.8 | 170.0 |
| | 165.1 | 18.1 | 164.2 | 18.4 | 164.5 | 18.3 | 15.2 |
| | 95.7 | 83.3 | 94.9 | 83.4 | 95.3 | 83.3 | 74.1 |
| | -31.2 | 202.3 | -32.0 | 202.2 | -31.0 | 202.1 | 197.5 |
| | 35.0 | 140.2 | 34.1 | 140.3 | 34.8 | 140.2 | 132.1 |
| | 4.2 | 169.1 | 3.6 | 168.9 | 4.6 | 168.6 | 170.2 |
| | 163.9 | 19.3 | 163.0 | 19.6 | 163.3 | 19.5 | 20.5 |
| | 31.9 | 143.2 | 31.3 | 142.9 | 31.8 | 143.1 | 136.9 |
| | 159.5 | 23.4 | 158.7 | 23.6 | 159.1 | 23.3 | 20.5 |
| **16** | 6.2 | 167.2 | 5.6 | 167.0 | 6.6 | 166.8 | 169.3 |
| | 49.4 | 126.7 | 48.3 | 127.0 | 48.9 | 127.0 | 125.3 |
| | 119.0 | 61.4 | 118.6 | 61.2 | 118.8 | 61.3 | 63.9 |
| | 112.5 | 67.5 | 111.9 | 67.4 | 112.2 | 67.5 | 66.8 |
| | 111.6 | 68.3 | 111.2 | 68.1 | 111.3 | 68.3 | 69.5 |
| | 86.6 | 91.8 | 86.3 | 91.4 | 86.5 | 91.6 | 90.7 |
| | 103.9 | 75.5 | 103.4 | 75.3 | 103.6 | 75.5 | 76.9 |
| | 105.2 | 74.4 | 104.7 | 74.2 | 104.9 | 74.3 | 75.0 |
| | 4.6 | 168.7 | 4.2 | 168.3 | 5.1 | 168.2 | 169.6 |
| | 50.9 | 125.3 | 49.8 | 125.6 | 50.4 | 125.6 | 124.8 |
| | 69.8 | 107.5 | 68.8 | 107.8 | 69.5 | 107.6 | 107.7 |
| | 31.9 | 143.1 | 31.0 | 143.2 | 31.6 | 143.3 | 145.1 |
| | 39.1 | 136.4 | 38.1 | 136.5 | 38.7 | 136.6 | 136.3 |
| | 34.3 | 140.8 | 33.6 | 140.8 | 34.2 | 140.8 | 144.2 |
| | 60.7 | 116.1 | 59.5 | 116.5 | 60.1 | 116.5 | 115.0 |
| | 3.8 | 169.5 | 3.4 | 169.1 | 4.3 | 168.9 | 170.5 |
| | 49.7 | 126.4 | 48.7 | 126.7 | 49.2 | 126.7 | 125.1 |
| | 62.9 | 114.0 | 61.8 | 114.4 | 62.4 | 114.3 | 113.7 |

| | | | | | | | |
|---|---|---|---|---|---|---|---|
| | 35.3 | 139.9 | 34.4 | 140.0 | 35.0 | 140.1 | 144.1 |
| | 43.6 | 132.2 | 42.6 | 132.3 | 43.2 | 132.3 | 135.9 |
| | 34.1 | 141.1 | 33.2 | 141.1 | 33.8 | 141.2 | 145.1 |
| | 71.7 | 105.8 | 70.6 | 106.1 | 71.3 | 106.0 | 107.2 |
| | 4.9 | 168.5 | 4.4 | 168.2 | 5.3 | 168.0 | 170.0 |
| | 50.2 | 126.0 | 49.1 | 126.3 | 49.6 | 126.3 | 125.2 |
| | 69.9 | 107.4 | 68.9 | 107.7 | 69.6 | 107.6 | 108.3 |
| | 31.6 | 143.4 | 30.8 | 143.4 | 31.3 | 143.5 | 145.1 |
| | 39.8 | 135.7 | 38.8 | 135.9 | 39.4 | 135.9 | 136.0 |
| | 34.4 | 140.8 | 33.6 | 140.7 | 34.3 | 140.7 | 144.2 |
| | 60.0 | 116.8 | 58.8 | 117.1 | 59.4 | 117.1 | 115.1 |
| | 61.1 | 115.8 | 60.0 | 116.1 | 60.6 | 116.0 | 113.9 |
| | 34.9 | 140.3 | 34.0 | 140.4 | 34.7 | 140.4 | 144.1 |
| | 43.4 | 132.4 | 42.4 | 132.5 | 43.1 | 132.5 | 136.0 |
| | 33.9 | 141.3 | 33.0 | 141.3 | 33.6 | 141.4 | 145.1 |
| | 70.8 | 106.6 | 69.8 | 106.8 | 70.4 | 106.8 | 107.2 |
| **17** | 113.3 | 66.7 | 112.6 | 66.8 | 112.9 | 66.9 | 70.8 |
| | 104.0 | 75.5 | 103.8 | 75.0 | 103.9 | 75.3 | 78.9 |
| | 88.7 | 89.8 | 88.4 | 89.4 | 88.7 | 89.6 | 93.7 |
| | 101.7 | 77.6 | 101.3 | 77.3 | 101.5 | 77.5 | 83.1 |
| | 106.4 | 73.2 | 106.1 | 72.9 | 106.2 | 73.1 | 77.8 |
| | 81.6 | 96.5 | 81.5 | 95.9 | 81.7 | 96.1 | 105.9 |
| | 105.7 | 73.9 | 105.4 | 73.5 | 105.5 | 73.8 | 77.6 |
| | 109.0 | 70.8 | 108.4 | 70.7 | 108.7 | 70.8 | 70.7 |
| | 103.2 | 76.2 | 102.8 | 75.9 | 103.1 | 76.0 | 77.7 |
| | 106.6 | 73.0 | 106.2 | 72.8 | 106.5 | 72.8 | 75.9 |
| | -2.4 | 175.3 | -3.0 | 175.0 | -2.0 | 174.8 | 175.8 |
| | 148.1 | 34.1 | 147.2 | 34.4 | 147.5 | 34.3 | 32.2 |
| | 149.7 | 32.6 | 149.0 | 32.6 | 149.3 | 32.6 | 32.2 |
| | 106.3 | 73.3 | 105.9 | 73.1 | 106.0 | 73.3 | 71.5 |
| | 106.5 | 73.2 | 105.7 | 73.3 | 105.9 | 73.4 | 75.4 |
| | 105.7 | 73.8 | 105.3 | 73.6 | 105.4 | 73.8 | 76.3 |
| | 154.8 | 27.8 | 154.1 | 27.9 | 154.5 | 27.7 | 29.3 |
| | 78.0 | 99.9 | 77.6 | 99.5 | 77.8 | 99.8 | 100.5 |
| | -48.3 | 218.4 | -49.0 | 218.1 | -47.9 | 218.0 | 213.7 |
| | 152.2 | 30.3 | 151.4 | 30.4 | 151.8 | 30.2 | 32.7 |
| | 111.0 | 68.9 | 110.5 | 68.7 | 110.6 | 69.0 | 70.9 |
| | 152.5 | 29.9 | 152.1 | 29.8 | 152.1 | 29.9 | 34.3 |
| | 115.2 | 65.0 | 114.8 | 64.7 | 115.0 | 64.9 | 63.4 |
| | 170.9 | 12.7 | 170.3 | 12.7 | 170.5 | 12.7 | 13.1 |
| | 8.6 | 165.0 | 7.9 | 164.9 | 8.9 | 164.6 | 167.8 |
| | 46.7 | 129.2 | 45.5 | 129.6 | 46.0 | 129.8 | 132.1 |
| | 45.6 | 130.3 | 44.6 | 130.5 | 45.2 | 130.4 | 130.8 |
| | 47.6 | 128.4 | 46.5 | 128.6 | 47.0 | 128.7 | 129.9 |
| | 41.6 | 134.1 | 40.6 | 134.2 | 41.1 | 134.3 | 134.4 |
| | 47.7 | 128.3 | 46.7 | 128.5 | 47.2 | 128.6 | 129.9 |
| | 45.9 | 130.0 | 45.0 | 130.1 | 45.5 | 130.2 | 130.8 |
| | 117.0 | 63.3 | 116.5 | 63.1 | 116.7 | 63.3 | 62.0 |
| | 120.5 | 60.0 | 120.1 | 59.8 | 120.2 | 60.0 | 62.3 |
| **18** | 23.2 | 151.3 | 22.5 | 151.2 | 23.0 | 151.3 | 142.8 |

| | | | | | | | |
|---|---|---|---|---|---|---|---|
| | 145.5 | 36.6 | 144.7 | 36.7 | 145.1 | 36.5 | 35.1 |
| | 102.1 | 77.2 | 101.6 | 77.1 | 101.8 | 77.2 | 76.3 |
| | 124.4 | 56.3 | 123.5 | 56.5 | 123.9 | 56.5 | 53.4 |
| | -40.2 | 210.8 | -41.0 | 210.6 | -40.0 | 210.6 | 204.3 |
| | 47.5 | 128.4 | 46.6 | 128.6 | 47.3 | 128.5 | 127.1 |
| | 105.1 | 74.5 | 104.6 | 74.2 | 105.0 | 74.2 | 72.3 |
| | 155.6 | 27.0 | 154.9 | 27.1 | 155.3 | 27.0 | 26.6 |
| | 146.9 | 35.2 | 146.0 | 35.5 | 146.4 | 35.3 | 38.2 |
| | 150.7 | 31.7 | 149.9 | 31.9 | 150.1 | 31.8 | 29.8 |
| | 73.4 | 104.2 | 73.1 | 103.8 | 73.3 | 104.1 | 106.8 |
| | -34.3 | 205.2 | -35.0 | 205.0 | -34.0 | 204.9 | 209.8 |
| | 122.7 | 57.9 | 121.9 | 58.1 | 122.1 | 58.2 | 53.8 |
| | 96.7 | 82.3 | 96.2 | 82.1 | 96.3 | 82.4 | 80.4 |
| | 98.7 | 80.4 | 98.1 | 80.3 | 98.4 | 80.4 | 78.5 |
| | 157.9 | 24.9 | 157.1 | 25.1 | 157.3 | 25.0 | 25.7 |
| | 157.6 | 25.2 | 156.9 | 25.3 | 157.1 | 25.2 | 24.7 |
| | 150.5 | 31.8 | 149.7 | 32.0 | 150.0 | 32.0 | 30.4 |
| | 128.8 | 52.2 | 128.2 | 52.2 | 128.3 | 52.4 | 49.3 |
| | 119.9 | 60.6 | 119.5 | 60.3 | 119.7 | 60.5 | 60.4 |
| | 146.0 | 36.0 | 145.3 | 36.2 | 145.5 | 36.2 | 31.2 |
| | 101.8 | 77.5 | 101.3 | 77.3 | 101.5 | 77.5 | 76.3 |
| | 95.8 | 83.1 | 95.3 | 83.0 | 95.6 | 83.1 | 80.6 |
| | 0.0 | 173.1 | -0.7 | 172.9 | 0.5 | 172.5 | 171.8 |
| | 1.3 | 171.9 | 0.5 | 171.8 | 1.7 | 171.3 | 167.3 |
| | 164.8 | 18.4 | 164.1 | 18.5 | 164.5 | 18.3 | 21.6 |
| | 153.5 | 29.0 | 152.8 | 29.1 | 153.2 | 29.0 | 24.4 |
| | 168.8 | 14.7 | 168.1 | 14.8 | 168.3 | 14.7 | 13.2 |
| **19** | 18.4 | 155.8 | 17.8 | 155.6 | 18.4 | 155.7 | 157.4 |
| | 85.4 | 92.9 | 84.3 | 93.3 | 84.8 | 93.2 | 96.5 |
| | 17.3 | 156.8 | 16.6 | 156.7 | 17.1 | 156.9 | 155.5 |
| | 79.8 | 98.2 | 78.5 | 98.7 | 79.3 | 98.4 | 100.3 |
| | 19.0 | 155.2 | 18.5 | 154.9 | 19.1 | 155.0 | 153.8 |
| | 79.1 | 98.9 | 78.0 | 99.2 | 78.6 | 99.1 | 106.7 |
| | 154.6 | 28.0 | 153.9 | 28.1 | 154.2 | 28.0 | 28.6 |
| | 119.7 | 60.7 | 119.3 | 60.5 | 119.5 | 60.6 | 66.1 |
| | 103.2 | 76.2 | 102.7 | 76.1 | 103.1 | 76.1 | 79.0 |
| | 46.6 | 129.4 | 45.5 | 129.6 | 46.1 | 129.7 | 130.4 |
| | 64.0 | 113.0 | 63.0 | 113.3 | 63.6 | 113.2 | 115.8 |
| | 53.6 | 122.8 | 52.5 | 123.0 | 53.2 | 123.0 | 120.3 |
| | 63.9 | 113.1 | 62.8 | 113.4 | 63.4 | 113.4 | 115.1 |
| | 33.9 | 141.3 | 32.9 | 141.4 | 33.5 | 141.4 | 145.1 |
| | 32.4 | 142.6 | 31.5 | 142.7 | 32.1 | 142.8 | 145.1 |
| | 149.5 | 32.8 | 148.6 | 33.1 | 149.1 | 32.8 | 36.5 |
| | 111.9 | 68.0 | 111.5 | 67.8 | 111.7 | 67.9 | 72.6 |
| | 76.1 | 101.7 | 74.9 | 102.1 | 75.7 | 101.8 | 100.3 |
| | 17.6 | 156.6 | 17.1 | 156.2 | 17.6 | 156.4 | 155.6 |
| | 100.7 | 78.6 | 100.2 | 78.4 | 100.5 | 78.5 | 76.6 |
| | 16.4 | 157.6 | 15.8 | 157.4 | 16.4 | 157.6 | 158.0 |
| | 85.4 | 92.9 | 84.3 | 93.3 | 84.8 | 93.2 | 96.3 |
| | 17.2 | 156.9 | 16.5 | 156.7 | 17.0 | 157.0 | 157.2 |

| | | | | | | | |
|---|---|---|---|---|---|---|---|
| | 85.1 | 93.2 | 84.0 | 93.6 | 84.6 | 93.4 | 95.9 |
| | 45.6 | 130.3 | 44.5 | 130.6 | 45.1 | 130.6 | 132.0 |
| | 60.6 | 116.2 | 59.5 | 116.5 | 60.0 | 116.5 | 119.0 |
| | 64.5 | 112.5 | 63.5 | 112.7 | 64.1 | 112.7 | 115.1 |
| | 31.6 | 143.4 | 30.7 | 143.5 | 31.2 | 143.6 | 145.1 |
| | 64.2 | 112.8 | 63.2 | 113.1 | 63.7 | 113.0 | 115.3 |
| | 33.4 | 141.7 | 32.5 | 141.8 | 33.1 | 141.9 | 145.1 |
| **20** | 119.1 | 61.4 | 118.7 | 61.0 | 118.8 | 61.2 | 60.1 |
| | 156.1 | 26.6 | 155.5 | 26.6 | 155.7 | 26.6 | 26.9 |
| | 149.4 | 32.8 | 148.7 | 32.9 | 149.1 | 32.8 | 31.7 |
| | 133.2 | 48.1 | 132.4 | 48.3 | 132.7 | 48.2 | 48.2 |
| | 118.4 | 61.9 | 117.9 | 61.8 | 118.2 | 61.9 | 60.1 |
| | 30.9 | 144.0 | 30.3 | 143.9 | 30.8 | 144.0 | 142.2 |
| | 59.2 | 117.5 | 58.2 | 117.7 | 58.8 | 117.7 | 116.3 |
| | 46.8 | 129.1 | 45.9 | 129.3 | 46.4 | 129.3 | 128.8 |
| | 52.0 | 124.3 | 51.0 | 124.5 | 51.6 | 124.5 | 124.4 |
| | 52.7 | 123.6 | 51.8 | 123.7 | 52.4 | 123.7 | 122.8 |
| | 39.6 | 135.9 | 38.6 | 136.1 | 39.2 | 136.1 | 132.9 |
| | 128.7 | 52.3 | 127.9 | 52.4 | 128.1 | 52.5 | 52.3 |
| | 138.7 | 42.9 | 138.3 | 42.7 | 138.5 | 42.8 | 42.7 |
| | 131.0 | 50.1 | 130.8 | 49.7 | 130.9 | 49.9 | 50.3 |
| | 129.0 | 52.0 | 128.6 | 51.8 | 128.8 | 51.8 | 52.9 |
| | 25.3 | 149.3 | 24.4 | 149.3 | 25.1 | 149.4 | 140.0 |
| | 44.5 | 131.3 | 43.9 | 131.1 | 44.5 | 131.1 | 127.9 |
| | 116.2 | 64.0 | 115.8 | 63.8 | 116.0 | 63.9 | 64.8 |
| | 101.3 | 78.0 | 100.9 | 77.8 | 101.1 | 77.9 | 77.6 |
| | 138.6 | 43.0 | 138.0 | 43.0 | 138.3 | 42.9 | 42.8 |
| | 1.2 | 171.9 | 0.6 | 171.7 | 1.6 | 171.5 | 170.3 |
| **21** | 48.8 | 127.2 | 47.9 | 127.4 | 48.4 | 127.4 | 128.0 |
| | 51.5 | 124.8 | 50.5 | 124.9 | 51.1 | 124.9 | 125.3 |
| | 53.8 | 122.6 | 52.9 | 122.7 | 53.5 | 122.6 | 122.3 |
| | 31.3 | 143.7 | 30.5 | 143.7 | 31.0 | 143.8 | 137.4 |
| | 34.8 | 140.4 | 34.2 | 140.2 | 34.7 | 140.3 | 141.4 |
| | 60.4 | 116.4 | 59.4 | 116.6 | 60.1 | 116.5 | 117.9 |
| | 126.8 | 54.1 | 126.1 | 54.1 | 126.2 | 54.3 | 52.8 |
| | 119.2 | 61.2 | 118.6 | 61.1 | 118.9 | 61.2 | 63.7 |
| | 2.3 | 170.9 | 1.9 | 170.5 | 2.9 | 170.3 | 167.7 |
| | 135.3 | 46.1 | 134.4 | 46.3 | 134.8 | 46.2 | 47.8 |
| | 127.1 | 53.8 | 126.6 | 53.6 | 126.8 | 53.7 | 57.5 |
| | 135.3 | 46.1 | 134.5 | 46.2 | 134.9 | 46.1 | 39.0 |
| | 137.9 | 43.6 | 137.6 | 43.3 | 137.8 | 43.4 | 36.5 |
| | 122.3 | 58.3 | 122.1 | 57.9 | 122.2 | 58.1 | 54.1 |
| | 112.7 | 67.3 | 112.5 | 66.9 | 112.5 | 67.1 | 66.3 |
| | 162.2 | 20.9 | 161.6 | 20.9 | 161.6 | 21.0 | 23.8 |
| | 152.5 | 30.0 | 151.8 | 30.0 | 152.1 | 30.0 | 29.3 |
| | 169.3 | 14.2 | 168.7 | 14.3 | 169.0 | 14.1 | 13.7 |
| | 54.8 | 121.7 | 54.1 | 121.6 | 54.7 | 121.5 | 117.7 |
| | 27.1 | 147.6 | 26.3 | 147.6 | 26.9 | 147.7 | 140.5 |
| | 123.0 | 57.7 | 122.5 | 57.5 | 122.7 | 57.6 | 52.3 |
| | 113.2 | 66.8 | 112.7 | 66.7 | 113.0 | 66.7 | 69.5 |

| | | | | | | | |
|---|---|---|---|---|---|---|---|
| | 131.7 | 49.4 | 131.1 | 49.5 | 131.2 | 49.6 | 47.9 |
| | 111.3 | 68.6 | 110.8 | 68.5 | 111.1 | 68.5 | 69.4 |
| | 22.7 | 151.7 | 22.1 | 151.5 | 22.7 | 151.6 | 150.0 |
| | 127.9 | 53.0 | 127.1 | 53.2 | 127.4 | 53.2 | 51.8 |
| | 39.8 | 135.7 | 38.9 | 135.8 | 39.5 | 135.8 | 134.3 |
| | 71.1 | 106.4 | 70.2 | 106.5 | 70.8 | 106.4 | 108.3 |
| | 48.9 | 127.2 | 48.0 | 127.3 | 48.5 | 127.3 | 128.2 |
| | 59.1 | 117.6 | 58.2 | 117.7 | 58.8 | 117.7 | 119.8 |
| | 53.5 | 122.9 | 52.6 | 122.9 | 53.3 | 122.9 | 122.2 |
| | 151.9 | 30.6 | 151.1 | 30.7 | 151.5 | 30.5 | 27.7 |
| | 152.9 | 29.6 | 152.3 | 29.6 | 152.5 | 29.5 | 28.5 |
| | 117.9 | 62.5 | 117.3 | 62.3 | 117.7 | 62.3 | 61.7 |
| | 127.3 | 53.6 | 127.2 | 53.1 | 127.2 | 53.4 | 53.7 |
| | 140.3 | 41.4 | 139.8 | 41.3 | 140.1 | 41.3 | 41.2 |
| | 122.3 | 58.3 | 122.0 | 58.0 | 122.1 | 58.2 | 58.1 |
| | 35.5 | 139.7 | 34.6 | 139.8 | 35.3 | 139.7 | 133.8 |
| | 53.4 | 122.9 | 52.7 | 122.9 | 53.3 | 122.9 | 122.2 |
| | 170.7 | 12.9 | 169.9 | 13.1 | 170.2 | 12.9 | 13.0 |
| **22** | 141.5 | 40.3 | 140.6 | 40.5 | 140.9 | 40.5 | 40.0 |
| | 31.0 | 144.0 | 30.4 | 143.8 | 30.9 | 143.9 | 143.4 |
| | 76.4 | 101.3 | 75.4 | 101.7 | 76.0 | 101.5 | 102.7 |
| | 48.7 | 127.4 | 47.7 | 127.6 | 48.3 | 127.5 | 129.9 |
| | 61.5 | 115.4 | 60.3 | 115.7 | 61.2 | 115.4 | 118.0 |
| | 41.5 | 134.1 | 40.7 | 134.2 | 41.1 | 134.3 | 136.5 |
| | 159.4 | 23.5 | 158.8 | 23.5 | 158.9 | 23.5 | 27.9 |
| | 154.6 | 28.0 | 153.9 | 28.1 | 154.2 | 28.0 | 27.3 |
| | 25.8 | 148.8 | 25.2 | 148.7 | 25.7 | 148.8 | 146.8 |
| | -14.8 | 187.0 | -15.7 | 187.0 | -14.8 | 186.9 | 188.3 |
| | 20.9 | 153.4 | 19.9 | 153.6 | 20.5 | 153.7 | 147.1 |
| | 39.5 | 136.0 | 38.4 | 136.3 | 39.2 | 136.1 | 135.2 |
| | 24.6 | 150.0 | 23.7 | 150.0 | 24.4 | 150.0 | 153.8 |
| | 57.8 | 118.8 | 57.0 | 118.9 | 57.6 | 118.8 | 119.7 |
| | 67.0 | 110.2 | 66.0 | 110.5 | 66.6 | 110.3 | 111.5 |
| | 68.4 | 108.9 | 67.4 | 109.1 | 68.0 | 109.1 | 108.3 |
| | -11.2 | 183.5 | -12.1 | 183.6 | -11.2 | 183.5 | 184.4 |
| | 154.6 | 28.0 | 153.8 | 28.2 | 153.9 | 28.2 | 28.8 |
| | 48.1 | 128.0 | 47.1 | 128.1 | 47.7 | 128.2 | 128.0 |
| | 58.1 | 118.6 | 57.1 | 118.8 | 57.6 | 118.8 | 119.6 |
| | 55.9 | 120.7 | 54.9 | 120.8 | 55.5 | 120.8 | 121.8 |
| | 40.1 | 135.4 | 39.3 | 135.4 | 39.9 | 135.5 | 137.3 |
| | 53.7 | 122.7 | 53.1 | 122.5 | 53.7 | 122.5 | 127.6 |
| | 62.6 | 114.3 | 61.8 | 114.4 | 62.4 | 114.3 | 111.6 |
| | 55.6 | 120.9 | 54.8 | 120.9 | 55.3 | 120.9 | 122.5 |
| | 56.3 | 120.2 | 55.5 | 120.3 | 56.1 | 120.2 | 120.8 |
| | 66.1 | 111.0 | 65.1 | 111.3 | 65.7 | 111.2 | 112.6 |
| | 36.1 | 139.2 | 35.2 | 139.2 | 35.7 | 139.4 | 133.4 |
| | 57.6 | 119.0 | 56.7 | 119.2 | 57.2 | 119.2 | 120.5 |
| | 55.0 | 121.5 | 54.1 | 121.6 | 54.6 | 121.7 | 122.6 |
| | 158.3 | 24.5 | 157.5 | 24.7 | 157.8 | 24.6 | 25.8 |
| | 167.9 | 15.5 | 167.2 | 15.6 | 167.4 | 15.5 | 17.8 |

| | | | | | | | |
|---|---|---|---|---|---|---|---|
| **23** | 135.6 | 45.9 | 135.1 | 45.7 | 135.3 | 45.7 | 46.9 |
| | 120.1 | 60.3 | 119.7 | 60.1 | 119.9 | 60.2 | 55.1 |
| | 150.0 | 32.3 | 149.5 | 32.2 | 149.7 | 32.2 | 30.2 |
| | 106.7 | 72.9 | 106.5 | 72.5 | 106.6 | 72.8 | 71.1 |
| | 4.2 | 169.1 | 3.5 | 169.0 | 4.5 | 168.8 | 171.6 |
| | 129.3 | 51.7 | 128.8 | 51.6 | 128.9 | 51.8 | 52.9 |
| | 143.2 | 38.7 | 142.6 | 38.6 | 142.8 | 38.7 | 41.4 |
| | 27.1 | 147.6 | 26.5 | 147.4 | 27.3 | 147.3 | 154.6 |
| | 128.7 | 52.3 | 128.3 | 52.0 | 128.4 | 52.2 | 49.3 |
| | 136.1 | 45.3 | 135.6 | 45.3 | 135.9 | 45.2 | 37.3 |
| | 148.4 | 33.8 | 147.9 | 33.7 | 148.2 | 33.7 | 23.7 |
| | -0.5 | 173.5 | -1.0 | 173.2 | -0.1 | 173.0 | 172.0 |
| | 9.0 | 164.6 | 8.4 | 164.4 | 9.2 | 164.3 | 168.1 |
| | 3.0 | 170.2 | 2.4 | 170.0 | 3.4 | 169.8 | 172.4 |
| | 128.5 | 52.5 | 127.9 | 52.5 | 128.1 | 52.6 | 55.4 |
| | 151.3 | 31.1 | 150.9 | 30.9 | 151.1 | 30.9 | 29.8 |
| | 68.8 | 108.5 | 67.8 | 108.7 | 68.4 | 108.7 | 105.9 |
| | 123.6 | 57.1 | 123.1 | 56.9 | 123.3 | 57.1 | 57.0 |
| | -1.9 | 174.8 | -2.5 | 174.6 | -1.5 | 174.4 | 173.6 |
| | 137.3 | 44.2 | 137.0 | 43.9 | 137.1 | 44.1 | 40.0 |
| | 120.5 | 60.0 | 120.0 | 59.8 | 120.1 | 60.0 | 63.3 |
| | 1.3 | 171.8 | 0.7 | 171.6 | 1.6 | 171.4 | 173.1 |
| | 115.7 | 64.5 | 115.2 | 64.3 | 115.4 | 64.5 | 60.0 |
| | 48.3 | 127.7 | 47.6 | 127.6 | 48.1 | 127.8 | 135.9 |
| | 24.8 | 149.7 | 24.1 | 149.7 | 24.9 | 149.5 | 157.5 |

[a] Parameters of linear regression models are presented in Table 2 in main text.
[b] For the references to the experimental works, please, see the main text.
